# Synthesis and Reactivity of an Iron–Tin Complex with Adjacent Stannylidyne and Ferriostannylene Units

**DOI:** 10.1021/jacs.4c18423

**Published:** 2025-02-11

**Authors:** Yang Liu, Franz F. Westermair, Isabelle Becker, Sebastian Hauer, Michael Bodensteiner, Christoph Hennig, Gábor Balázs, Franc Meyer, Ruth M. Gschwind, Robert Wolf

**Affiliations:** † Institute of Inorganic Chemistry, University of Regensburg, Regensburg 93040, Germany; ‡ Institute of Organic Chemistry, University of Regensburg, Regensburg 93040, Germany; § Institute of Inorganic Chemistry, University of Göttingen, Göttingen 37077, Germany; ∥ European Synchrotron Radiation Facility, Rossendorf Beamline (BM20-CRG), Grenoble 38043, France; ⊥ Institute of Resource Ecology, Helmholtz-Zentrum Dresden-Rossendorf, Dresden 01314, Germany

## Abstract

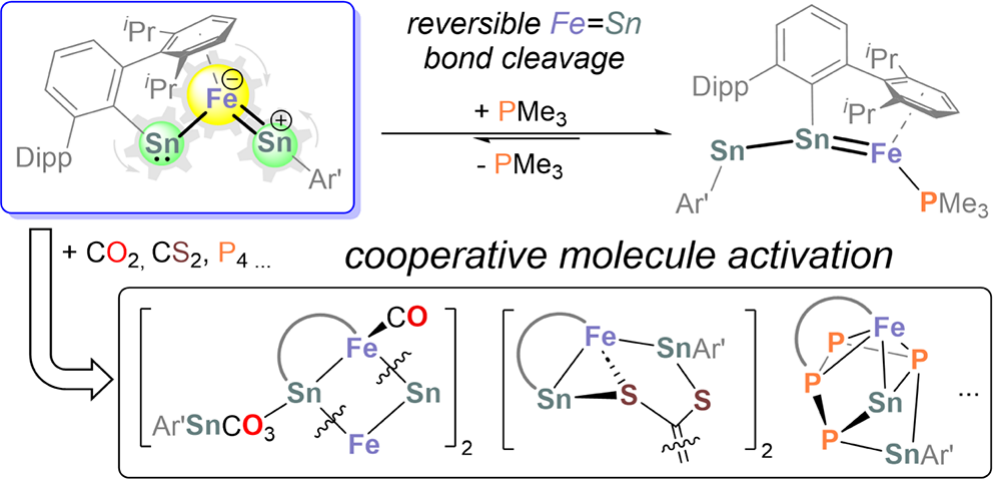

Heavier transition metal carbyne analogs hold significant potential for cooperative activation of small molecules. However, complexes containing more than one heavier tetrylidyne ligand RE (E = Si, Ge, Sn, Pb) are rare due to the high oligomerization tendency of RE ligands. In this study, we describe the complex [Fe(SnAr’)_2_] (**1**; Ar’ = 2,6-Dipp_2_-C_6_H_3_, Dipp = 2,6-*^i^*Pr_2_–C_6_H_3_), which features adjacent Fe–Sn single and double bonds. Complex **1** exhibits versatile reactivity with transition metal and main group compounds. Treatment of complex **1** with Ni(COD)_2_ (COD = 1,5-cyclooctadiene) yields the tetranuclear complex [Fe(μ-SnAr’)_2_Ni] (**2**), characterized by an unusual “push–pull” interaction between nickel(0) and the two coordinating Sn atoms, as revealed by quantum chemical studies. The reaction of complex **1** with AlBr_3_ results in Al–Br bond cleavage and Ar’ migration to aluminum. CH_3_I adds oxidatively to the Sn atom that is singly bonded to Fe, while PMe_3_ coordinates to Fe, inducing reversible cleavage of the Fe=Sn double bond. In addition, complex **1** activates inorganic molecules. CO_2_ undergoes disproportionation to produce a carbonate-bridged Ar’Sn(μ–OCO_2_)SnAr’ ligand, whereas CS_2_ is reductively coupled to form an ethylene tetrathiolate ligand ([C_2_S_4_]^4–^). The reaction with white phosphorus (P_4_) generates an unusual Ar’P_4_Sn_2_Ar’ ligand. This multifaceted reactivity illustrates the behavior of the Fe and Sn sites in complex **1**, suggesting that complexes of this type are promising reagents for small molecule activation.

## Introduction

**Figure 1:**
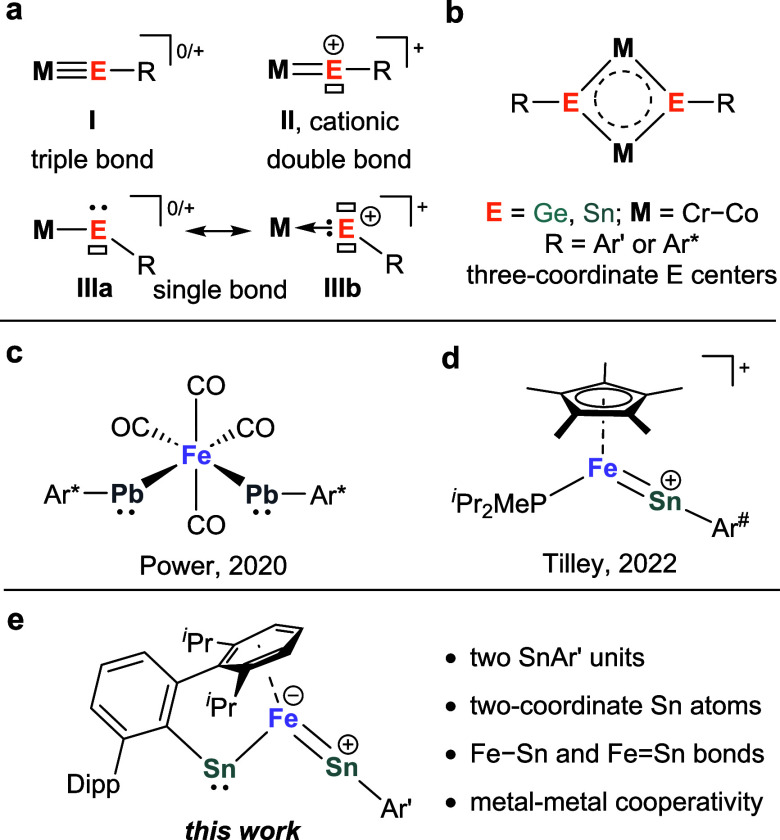
(a) Bonding modes between transition metal and monosubstituted tetryl element ligand. (b) M_2_E_2_ clusters. (c) Ferriobis(plumbylene) with two PbAr^*^ units. (d) Iron–stannylidyne complex featuring Fe=Sn double bond. (e) The FeSn_2_ cluster **1**.

Terminal carbyne complexes typically possessing a metal–carbon triple bond M≡CR (Figure 1a, **I**), have been widely investigated ever since the pioneering works by Fischer and Schrock in the 1970s,^1,2^ unfolding their broad applications in materials science and organic transformations, e.g., alkyne metathesis.^3^ In contrast, metal complexes with monosubstituted heavier tetrylidyne ligands RE (E = Si, Ge, Sn, Pb) are much less explored. These heavier carbyne homologues exhibit M–ER bonding interactions and reactivities that differ significantly from their carbon counterparts.^4^

The heavier group 14 elements, Si–Pb, show a strong reluctance to engage in s/p valence orbital hybridization, resulting in a decreased tendency for heavier tetrylidynes RE to form multiple bonds.^5^ Nevertheless, a few examples of M≡ER triple bonds, similar to metal carbyne complexes (Figure 1a, **I**), and metallotetrylenes showing M–ER single bonds (Figure 1a, **III**) have been reported.^4,6,7,8^ The singly bonded RE ligands (M–ER) are usually amphiphilic due to a chemically active electron lone pair and vacant p orbital(s) on the p-block element (Figure 1a, **III**).^9^ Additionally, the Wesemann and Tilley groups reported several cationic metal complexes [M=ER]^+^ (M = Ti, Ir, Co; E = Ge, Sn) bearing M=E double bonds recently.^10,11^ In these complexes, the positive charge is largely localized on the p-block atom which also possesses an empty p-orbital to avoid the otherwise reactive RE radical (Figure 1a, **II**). Noteworthily, [Ir=ER]^+^ (E = Ge, Sn) complexes have shown pronounced reactivity in cooperative X–H bond activation (X = N, O, H).^10b^

Overall, the unique bonding characteristics, coupled with the cooperativity between the metal and p-block centers, endow [M(ER)] complexes with distinctive reactivity, positioning them at the forefront of synthetic chemistry in the quest for innovative molecular activation methodologies. The application of [M(ER)] (E = Si–Pb) complexes in cooperative small molecule activation, such as H_2_,^10b,12^ NH_3_,^10b,13^ CO_2_^10c^ and aldehydes/ketones,^14^ as well as in catalysis^9c^ has been demonstrated, although the number of successful examples remains relatively limited.

Inspired by the rapidly growing family of multimetallic systems that exhibit enhanced reactivity due to cooperative effects,^15^ we sought to further develop this strategy by incorporating two heavier RE units into a transition metal complex. Specifically, we targeted a rare heterotrimetallic ME_2_ core with two-coordinate p-block metal centers. Compared to the ubiquitous heterobimetallic ME core, such a system would be of fundamental interest not only in general synthetic chemistry but also in molecular activation, as it offers potentially greater flexibility for substrate binding in terms of available reactive sites from multiple M/E centers, and hence greater scope of reactivity.

However, the synthesis of type [M(ER)_2_] (E = Si–Pb) complexes with adjacent RE ligands is challenging due to the inherent propensity of RE moieties toward dimerization to form a ditetrylyne unit REER.^16^ Power and coworkers have described heterotetrametallic complexes [M(ER)]_2_ (M = Cr, Mo, W, Fe, Co; E = Ge, Sn; Figure 1b) featuring rhomboid M_2_E_2_ cores.^17^ Each RE ligand bridges two metal atoms in those complexes, resulting in three-coordinate E centers with suppressed electrophilicity and nucleophilicity due to the additional M → E/E → M interactions, reducing their reactivity. Nevertheless, the Co_2_Sn_2_Ar’_2_ (Ar’ = 2,6-Dipp_2_-C_6_H_3_, Dipp = 2,6-*^i^*Pr_2_-C_6_H_3_) cluster activated white phosphorus (P_4_), affording an unusual ternary complex with a Co_2_Sn_2_P_4_ core.^17c^ To our knowledge, the only well-defined complex containing more than one coordinating RE unit with two-coordinate group 14 atoms is [(CO)_4_Fe(PbAr*)_2_] (Ar* = 2,6-Trip_2_-C_6_H_3_, Trip = 2,4,6-*^i^*Pr_3_-C_6_H_2_; Figure 1c), synthesized by the reaction of a diplumbyne with Fe_2_(CO)_9_.^18^ The two Fe–Pb–C*_ipso_* linkages are bent and feature Fe–Pb single bonds (type **IIIa**). However, its reactivity was not reported. Additionally, complexes featuring Fe–Sn multiple bonds are rare, with most known examples being iron-stannylene complexes [Fe(SnR_2_)].^11,19^ Tilley’s cationic complex [Cp*(*^i^*Pr_2_MeP)Fe(SnAr^#^)]^+^ (Figure 1d) and the related hydride [Cp*(*^i^*Pr_2_MeP)Fe(H)(SnAr^#^)]^+^ (Ar^#^ = 2,6-Mes_2_-C_6_H_3_, Mes = 2,4,6-Me_3_-C_6_H_2_) appear to be the only iron-stannylidyne complexes reported so far. According to structural and quantum chemical studies, these complexes show a type **II** Fe=Sn double bond.^11^ Remarkably, the reduction of this complex by a single electron led to intramolecular C–H bond activation, which was proposed to proceed through a neutral iron-stannylyne intermediate featuring an Fe=Sn double bond. However, isolable neutral complexes featuring M=ER double bonds are still unknown to date.

Here, we report the synthesis and characterization of the neutral FeSn_2_ cluster **1** bearing two adjacent Ar’Sn ligands (Figure 1e). The bonding situation and electronic structure of **1** have been established spectroscopically and computationally, revealing an Fe–Sn single bond and an Fe=Sn double bond within the same molecule. The reactivity of this heterotrimetallic system toward Lewis acid (AlBr_3_), Lewis base (PMe_3_), and small inorganic molecules such as P_4_, CS_2_ and CO_2_ was investigated. These studies illustrate a highly versatile and cooperative reactivity of the amphiphilic FeSn_2_ core of **1**.

## Results and Discussion

### Synthesis and Characterization

**Scheme 1:**
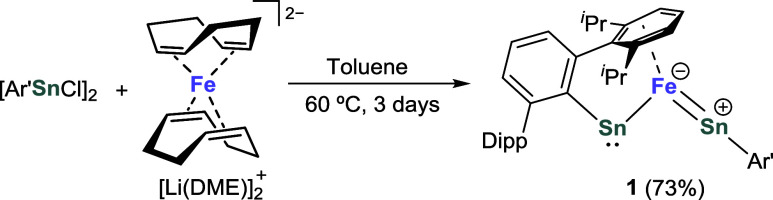
Synthesis of Complex **1**

Treatment of [Ar’SnCl]_2_ with one equivalent of the Fe^2–^ synthon [Li(DME)]_2_[Fe(COD)_2_] (COD = 1,5-cyclooctadiene, DME = 1,2-dimethoxyethane) in toluene at 60 °C (3 days) leads to the formation of the title complex [Fe(SnAr’)_2_] (**1**) in 73% yield as dark brown crystals after workup (Scheme 1). The reaction of [Ar’SnCl]_2_ with two equivalents of the Fe^–^ synthon [K(18-c-6)][Fe(η^4^-anthracene)_2_] (18-c-6 = 18-crown-6) still yields **1**, showing the preference for a single Fe site of this system. Complex **1** is stable at room temperature (r.t.) in an inert atmosphere, both in the solid state and in common organic solvents (e.g., benzene, toluene and THF). Heating a solution of **1** in C_6_D_6_ to 80 °C for several days does not result in any noticeable decomposition as evidenced by NMR monitoring experiments. However, **1** decomposes rapidly in air to give intractable mixtures.

**Figure 2:**
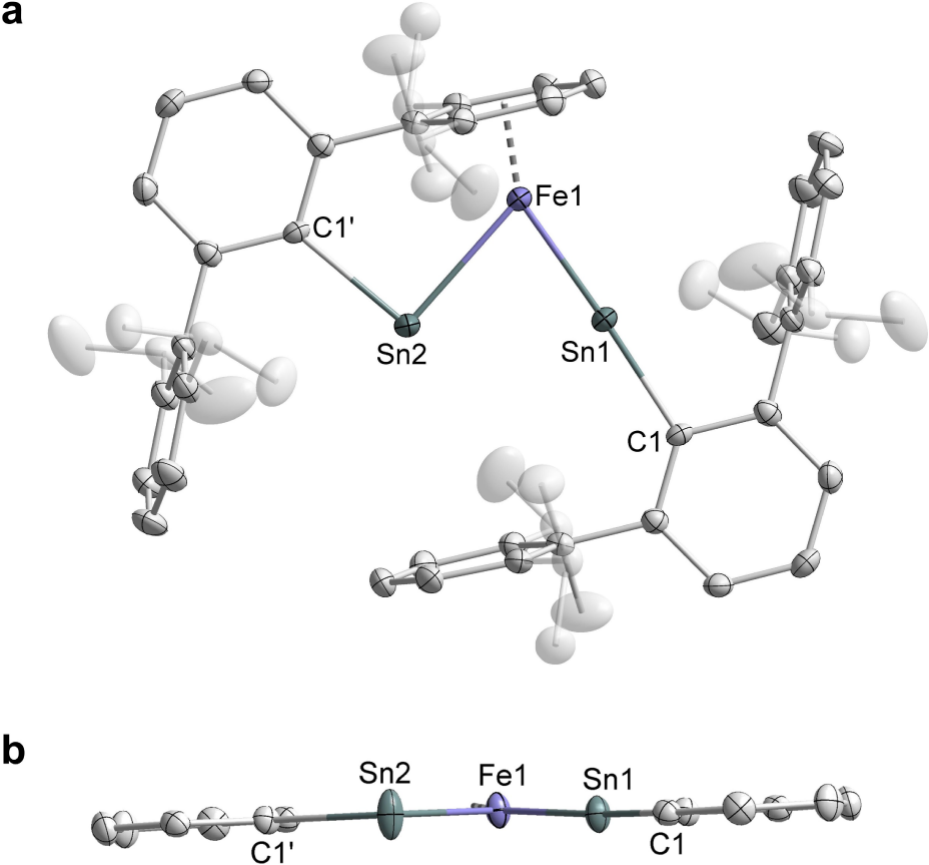
Molecular structure of complex **1** with 50% probability ellipsoids: front view (a) and side view without Dipp groups (b). Hydrogen atoms are omitted for clarity. Selected bond lengths [Å] and angles [°]: Fe1–Sn1 2.2954(7), Fe1–Sn2 2.6207(7), Sn1–C1 2.135(2), Sn2–C1’ 2.243(2), Sn1···Sn2 3.0595(6); Sn1–Fe1–Sn2 76.66(2), Fe1–Sn1–C1 176.60(6), Fe1–Sn2–C1’ 91.10(5).

The molecular structure of **1** was determined by single-crystal X-ray crystallography (Figure 2) and further supported by multinuclear NMR spectroscopy, HR-LIFDI-mass spectrometry, inductively coupled plasma-optical emission spectroscopy (ICP-OES) and elemental analysis. The molecular structure of **1** features a unique FeSn_2_ core where the iron atom bridges the two Ar’Sn moieties in an asymmetric fashion and is η^6^-coordinated by a flanking Dipp-aryl ring of one Ar’ substituent (Figure 2a). The overall structure of compound **1** resembles an unknown metal η^2^-distannyne complex. Notably, in metal *η*^2^-disilyne^20^ and η^2^-digermyne^16c,21^ complexes, [M(η^2^-R’EER’)] (M = Ni, Pd, Pt, Ag; E = Si, Ge; R’ = alkyl, aryl or silylene substituent), the ME_2_ rings are perpendicular to the C–E–E–C arrays so that the metal centers can interact with the E≡E π_out_ orbitals in a classic way that is analogous to metal η^2^-alkyne complexes. However, the FeSn_2_ plane in **1** is coplanar with the C1’–Sn1···Sn2–C1 array (Figure 2b). Given that the slipped Sn–Sn π_in_ orbital in the distannyne compound [Ar’SnSnAr’] is reluctant to engage in bonding, a distinct Sn–Fe–Sn bonding mode is expected for **1** (*vide infra*).^22^

The Fe1–Sn2 bond length of 2.6207(7) Å and the bent geometry at Sn2 (∠Fe1–Sn2–C1’: 91.10(5)°) in **1** signify a metallostannylene structural motif of the Fe1Sn2Ar’ unit with a type **III** Fe1–Sn2 single bond (Figure 1a). Compared to the structures of known ferriostannylene complexes (Fe–Sn: 2.56–2.61 Å and ∠Fe–Sn–C: 106.7–118.2°),^11,17b,23^ the Fe1–Sn2 bond length is slightly longer, and the Fe1–Sn2–C1’ angle is more acute, probably due to the strain induced by the coordination of the arene moiety to iron. In distinct contrast, the Fe1–Sn1 bond length of 2.2954(7) Å is significantly shorter than the Fe1–Sn2 bond, and the geometry at Sn1 is nearly linear (∠Fe1–Sn1–C1: 176.60(6)°). These metrical parameters are comparable with those in [Cp*(*^i^*Pr_2_MeP)Fe(SnAr^#^)]^+^ (2.2889(6) Å and 169.85(9)°; Figure 1d)^11^ and complex **4** (2.2960(4) Å and 175.45(8)°; *vide infra*), indicating the Fe1=Sn1 double bond character in **1**. The Sn1···Sn2 distance of 3.0595(6) Å is substantially longer than those of the relevant distannyne compound [Ar’SnSnAr’] (2.667(5) Å)^16a^ and complex [Co(SnAr’)]_2_ (2.8700(5) Å),^17c^ as well as a regular Sn–Sn single bond (ca. 2.80 Å),^24^ but shorter than the sum of the van der Waals radii of tin (∑*r*_vdW_ = 4.84 Å).^25^

The ^1^H NMR spectrum of **1** (C_6_D_6_, 298 K) shows three sets of signals attributed to the Dipp flanking groups with an integral ratio of 1:1:2, agreeing with the asymmetric coordination pattern (Figure S1). Neither broadening nor coalescence of these signals was observed at 341 K, indicating the absence of dynamic behavior, i.e., exchange of the coordinating aryl groups on the NMR time scale (Figure S4). The ^119^Sn{^1^H} NMR spectrum of **1** shows two singlets with remarkably different chemical shifts at +466 ppm and +3229 ppm, which are assigned to Sn1 and Sn2, respectively. This assignment is supported by DFT calculations (Table S19). The downfield-shifted signal of Sn2 lies within the range of 2515–3762 ppm, characteristic of ferriostannylenes (type **IIIa**)^11,23^ and other metallostannylenes with two-coordinate Sn centers.^10b,12a^ The signal of Sn1 is markedly upfield shifted relative to Sn2, which indicates a strong Fe → Sn back-donation, viz. the formation of a multiple bond, and is similar to that of the doubly bonded Sn1 in **4** (+768 ppm; *vide infra*). By contrast, the cationic complex [Cp*(*^i^*Pr_2_MeP)Fe(SnAr^#^)]^+^ shows a ^119^Sn{^1^H} NMR resonance at +1024 ppm.^11^ The zero-field ^57^Fe Mössbauer spectrum of **1**, recorded at 80 K for a solid sample, displays a doublet with the isomer shift value of δ = 0.61 mm s^–1^ and quadrupole splitting value of |Δ*E*_Q_| = 0.66 mm s^–1^. Although the relationship between the δ/Δ*E*_Q_ values and oxidation states of iron complexes featuring low-coordinate and low-valent Fe centers remains ambiguous,^26^ the isomer shift of **1** is positively shifted compared with known low-valent, η^6^-arene-coordinate Fe^0^ and Fe^–I^ systems, such as [bis(NHC)Fe^0^(η^6^-arene)] (0.43 mm s^–1^; NHC = *N*-heterocyclic carbene),^27^ [(η^6^-arene)Fe^0^Ge(N’’’)(NHC)]^+^ (0.47 mm s^–1^; N’’’ = {[Ph_2_PCH_2_Si(*^i^*Pr)_2_](Dipp)N},^12b^ and [(η^6^-arene)Fe^–I^Sn(N’’’)(NHC)] (0.52 mm s^–1^).^12b^ The higher isomer shift of **1** may therefore indicate a comparatively high d-electron density at the Fe atom, which shields the s-electrons from the nucleus. The UV–vis spectrum of **1** (toluene, r.t.) shows a prominent band at 450 nm and two weak, broad bands at 590 and 730 nm. These absorption features are assigned to transitions primarily from the highest occupied molecular orbitals (HOMO and HOMO–1) to the lowest unoccupied molecular orbitals (LUMO, LUMO+1, and LUMO+2), based on time-dependent DFT calculations (Figure S61).

### Electronic Structure and Bonding

**Figure 3:**
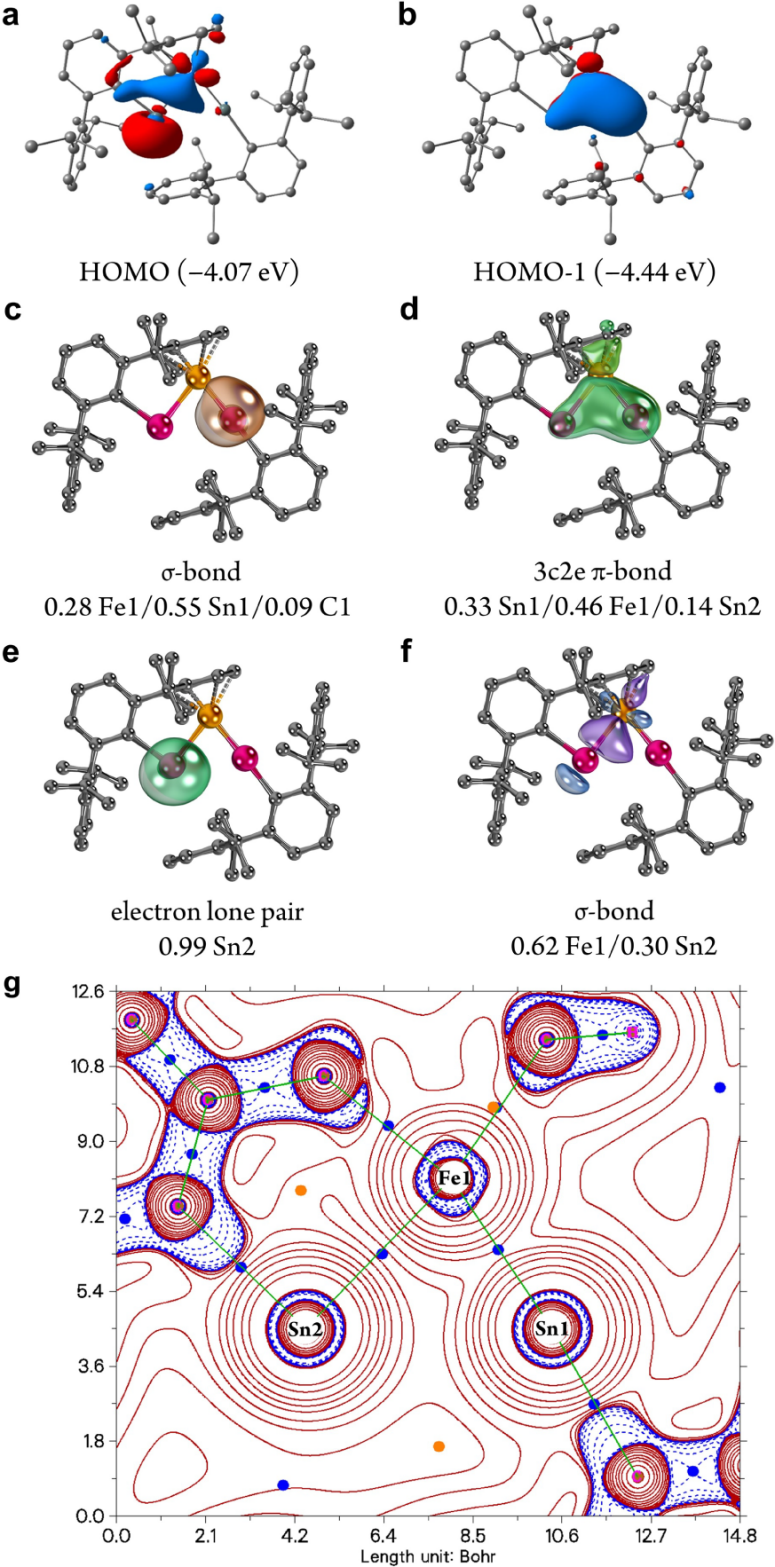
(a,b) Selected MOs of **1** (Isovalue = 0.04). (c–f) Selected IBOs of **1**. The bond type and percentage of the electron density on Fe and Sn are given. (g) Plot of the Laplacian of the electron density on the FeSn_2_ plane of **1** (BCP: blue dots, RCP: brown dots, bond paths: green lines).

To gain more insight into the electronic structure of **1**, quantum chemical calculations were performed at different levels of theory (see Supporting Information for details). The optimized structure agrees very well with the X-ray crystallographic data (Figure S60 and Table S7). The HOMO (−4.07 eV) represents the Fe1–Sn2 σ-bond with significant contribution from the electron lone pair on Sn2 (31% Fe, 46% Sn2; Figure 3a). HOMO–1 (−4.44 eV) corresponds to the Fe1–Sn1 out-of-plane π-bond with minor delocalization toward Sn2, forming a 3-center-2-electron (3c2e) π-interaction over the FeSn_2_ core (37% Fe1, 30% Sn1, 13% Sn2; Figure 3b). The Fe1–Sn1 σ-bond is much lower in energy (HOMO–16, −7.27 eV; Figure S62). The LUMO and LUMO+2 mainly consist of the Sn-centered p orbitals, specifically the orthogonally oriented Sn2 p_out_ and Sn1 p_in_ orbitals, while the LUMO+1 is mainly contributed by the Fe 3d orbitals (Figure S62). The bonding modes between Fe and Sn atoms in **1** are further illustrated by electron localization function (ELF)^28^ analysis, which exhibits the electron distribution patterns consistent with an Fe1–Sn1 double bond, an Fe1–Sn2 single bond with weak π-interaction, and relatively low ELF value between the two Sn atoms (Figure S64). The Mayer bond order (MBO) of Fe1–Sn1 (1.50) and Fe1–Sn2 (0.94) bonds suggests their double and single bond character, respectively, which is in line with the canonical MO and ELF analyses. The MBO of the Sn1···Sn2 (0.59) unit is relatively low, suggesting a rather weak Sn···Sn interaction in **1**. An independent gradient model based on Hirshfeld partition (IGMH)^29^ analysis also reveals that the Sn···Sn interaction is much weaker compared to the Fe–Sn interactions (Figure S65).

Intrinsic bond orbital (IBO)^30^ analysis provides further insights into the bonding situation of **1**. The Fe1–Sn1 σ-bonding interaction consists mainly of the Sn1-based lone pair, thus representing a dative bond (Figure 3c). The out-of-plane Fe1–Sn1 π-bond extends to the adjacent Sn2 atom, which aligns with the HOMO–1 of **1** (Figure 3d). A nonbonding electron lone pair resides exclusively at Sn2 and lies in the FeSn_2_ plane (Figure 3e). The Fe1–Sn2 σ-bond shows strong polarization toward Fe1 (Figure 3f). The natural charge distribution (Fe1: −0.65, Sn1: +1.04, Sn2: +0.92; Table S8) reveals an electron-rich iron center, which is consistent with the high positive isomer shift value obtained from the ^57^Fe Mössbauer measurement of **1** (*vide supra*), and the positive charge accumulation of Sn centers. Furthermore, substantial net electron transfer from the Ar’Sn1 moiety (+0.52 au) to the Ar’Sn2Fe unit is calculated, implying a zwitterionic character of **1** (Table S8). Quantum theory of atoms in molecules (QTAIM)^31^ analysis reveals two Fe–Sn bond critical points (BCP; Figure 3g). However, neither a BCP between Sn1 and Sn2 atoms nor a ring critical point (RCP) within FeSn_2_ unit could be located, indicating the absence of a Sn1–Sn2 bond. The energy density (*H*(r)) and ellipticity of electron density (ε(r)) at the BCP of Fe1–Sn1 bond (*H*(r): −0.031 e.a_0_^–3^; ε(r) = 0.15) are larger than those of the Fe1–Sn2 bond (*H*(r): −0.015 e.a_0_^–3^; ε(r) = 0.09), providing additional evidence for the multiple bond character of the Fe1–Sn1 bond.^32^ The delocalization index (DI)^33^ values of the basins between Fe1 and Sn1 (1.41)/Sn2 (0.94), and Sn1 and Sn2 (0.46) agree well with the MBO.

An energy decomposition analysis coupled with natural orbitals for chemical valence (EDA-NOCV)^34^ of the Fe1=Sn1 bond provides a consistent bonding scenario with the above analyses. The smallest orbital interaction energy |Δ*E*_orb_| (139.62 kcal mol^–1^) was found between the [Ar’Sn2Fe]^−^ anion and the [Ar’Sn1]^+^ cation fragments, both in the singlet state, which is significantly smaller than those of other states (e.g., *S* = 1/2–4; 198.59–331.16 kcal mol^–1^; Table S17 and Figure S81 and see SI for details). These data further corroborate the zwitterionic character of **1** and indicate the presence of a σ-donor-π-acceptor type double bond between Fe1 and Sn1.

Overall, the bonding of the FeSn_2_ core in **1** can be best described as containing an Fe1=Sn1 double bond, comprising one σ- and one π-bond, and a type **IIIa** Fe1–Sn2 single bond, with little or negligible Sn1···Sn2 interaction. It is worth noting that the ligand constraint might play a crucial role in the formation of such a bonding situation. For example, the complex [(η^2^-RSiSiR)Fe(η^6^-C_6_H_6_)] (*R* = *κ*^*2*^-amidinate),^35^ shows a three-membered FeSi_2_ metallacyclic structure. Additionally, complex **1** has a zwitterionic character with the positive charge residing primarily at the doubly bonded Sn1 atom. Noteworthily, a few cationic transition metal complexes with M=ER double bonds have been reported (e.g., [(PMe_3_)_3_(H)M(EAr*)]^+^ (M = Ir, Co; E = Ge, Sn)^10b,10c^ and [Cp*(*^i^*Pr_2_MeP)Fe(SnAr^#^)]^+^,^11^Figure 1d). To our knowledge, **1** represents the first isolable neutral iron-stannylidyne complex featuring an Fe=Sn double bond.

### Reactivity Study

**Scheme 2:**
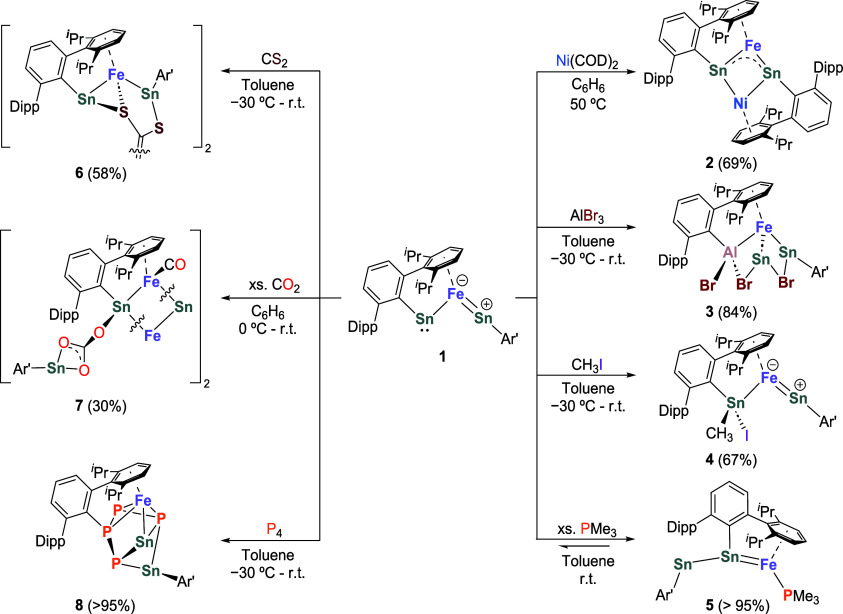
Reactivity Study of Complex **1**

**Figure 4:**
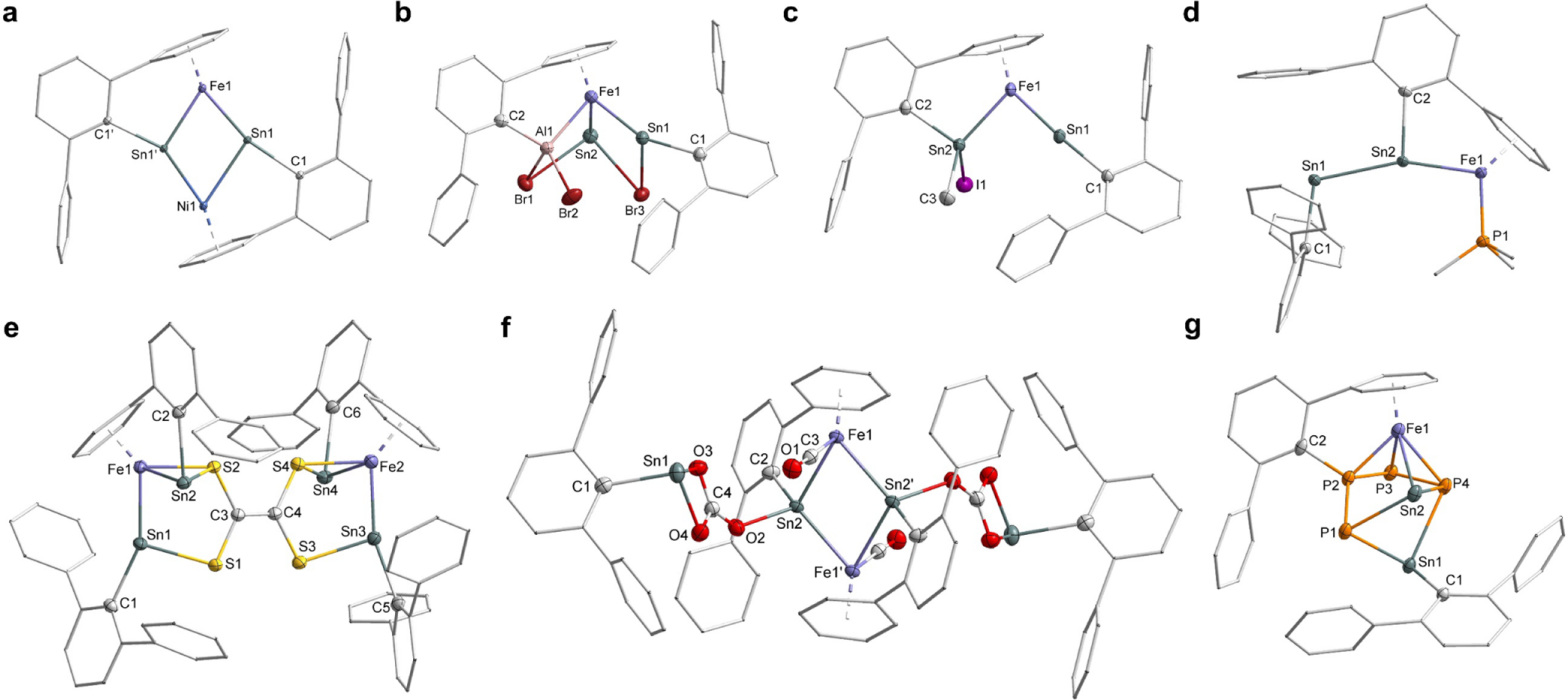
Molecular structures of **2** (a), **3** (b), **4** (c), **5** (d), **6** (e), **7** (f), and **8** (g), showing 50% probability thermal ellipsoids. Hydrogen atoms, solvent molecules, and the *^i^*Pr substituents are omitted for clarity.

To test if the lone pair on Sn2 and the p-vacant orbital on Sn1 could potentially form “push-pull” interactions, we performed the reaction of **1** with Ni(COD)_2_. This led to the formation of complex **2** as dark green crystals in 69% yield (Scheme 2). The solid-state molecular structure of **2** (Figure 4a) reveals a planar, quasi-rhomboid FeNiSn_2_ core structurally similar to the Co_2_Sn_2_ core in complex [Co(SnAr’)]_2_.^17c^ The Ni1–Sn1 (2.7018(24) Å) and Ni1–Sn1’ (2.4328(22) Å) bond lengths are comparable to the bis(stannylene)nickel complexes reported by Wesemann and coworkers, where two stannylene (SnR_2_) ligands serve as L- and Z-type ligands.^36^ The Fe1–Sn1 (2.3775(22) Å) and Fe1–Sn1’ (2.4431(23) Å) bonds are elongated and shortened, respectively, compared to those in **1** (Table 1).

**Table 1:** Selected Bond Lengths (Å) and Angles (Deg) and Experimental ^119^Sn NMR and ^57^Fe Mössbauer Data of Complexes **1**–**8**

	Fe–Sn1 (Å)	Fe–E (Å)	Fe1–Sn1–C, Fe1–E–C (deg)	^119^Sn NMR δ_Sn1_, δ_E_ (ppm)^a^	^57^Fe Mössbauer δ, |Δ*E*_Q_| (mm s^–1^)
**1**, E=Sn2	2.2954(7)	2.6207(7)	176.60(6), 91.10(5)	466, 3229	0.61, 0.66
**2**, E=Sn1’	2.3775(22)	2.4431(23)	165.29(7), 95.98(7)	369, 1701	0.55, 0.76
**3**, E=Sn2	2.3768(9)	2.5249(6)	158.02(9)	15, 2393	
**4**, E=Sn2	2.2960(4)	2.5188(5)	175.45(8), 97.45(8)	768, 107^b^	
**5**, E=Sn2		2.4864(7)	99.78(2)	2403, 1456	0.50, 1.17
**6**, E=Sn2	2.4136(8)	2.7925(8)	154.92(5), 86.14(5)	–124, 655^c^	
**7**, E=Sn2/Sn2’		2.5421(5)/2.5874(6)	96.50(7), 132.50(7)	264, 343^c^	
**8**, E=Sn2		2.7892(7)		–455, −366^d^	

a Signals were assigned based on DFT calculations (Table S19).

b Signals were assigned based on a comparison with literature data.

c Signals were not assigned.

d Multiplets due to the Sn–P coupling.

Albeit isoelectronic, complex **2** shows a distinct electronic structure from [Co(SnAr’)]_2_.^17c^ The IBO analysis of **2** reveals that the electron lone pair from Sn1’ coordinates to Ni forming a highly polarized Ni1–Sn1’ σ-bond (0.29 Ni1/0.53 Sn1’), while the Ni1–Sn1 bond is predominantly contributed by Ni 3d orbitals (0.90 Ni1/0.13 Sn1; Figure S68). This suggests a Sn1’ → Ni → Sn1 “push-pull” interaction, showcasing the electron donating and accepting properties of Sn2 and Sn1 in **1**, respectively. In addition, IBO and MBO analyses of the Fe–Sn bonds in **2** (IBOπ_Sn1–Fe1–Sn1’_: 0.29 Sn1/0.44 Fe1/0.20 Sn1’; MBO_Fe1–Sn1_: 1.26, MBO_Fe1–Sn1’_: 1.22; Figure S68 and Table S10), indicate a π-electron flow from Sn1 to Sn2 (*via* the central Fe) upon the addition of Ni. Despite the contraction of the Sn1···Sn1’ distance to 2.8860(3) Å in **2** (vs 3.0595(6) Å in **1**), QTAIM analysis suggests no Sn1–Sn1’ bond (Figure S69). The ^1^H NMR spectra of **2** recorded at 298 and 341 K are nearly identical, displaying four sets of signals corresponding to the Dipp groups. This observation is consistent with the asymmetric structure observed in the solid state and indicates no dynamic behavior in solution on the NMR time scale (Figure S8). Due to the increase in the coordination number of Sn1’, a considerable upfield shift of its ^119^Sn{^1^H} NMR resonance signal (+1701 ppm vs +3229 ppm in **1**; Table 1) was observed,^37^ while the signal at +369 ppm was assigned to Sn1 (Table S19). The zero-field ^57^Fe Mössbauer spectrum (80 K, solid state) shows a doublet with δ = 0.55 mm s^–1^ and |Δ*E*_Q_| = 0.76 mm s^–1^. The smaller δ value of **2** compared to **1** (0.66 mm s^–1^; Table 1) suggests a decrease of electron density on Fe1, which is consistent with the change of its natural charge (Fe1: −0.46 in **2** vs −0.65 in **1**; Table S8).

To probe the potential amphiphilic reactivity of the Sn centers in **1**, as predicted by the canonical MO analysis, we investigated the reactivity of **1** toward the Lewis acid AlBr_3_, the electrophile CH_3_I, and the nucleophile Me_3_P. At −30 °C, **1** reacts readily with AlBr_3_, affording complex **3** as maroon crystals in 84% yield (Scheme 2). The solid-state molecular structure of **3** (Figure 4b) shows that, instead of a donor–acceptor adduct, Al–Br bond cleavage along with Ar’ migration occurred. The Ar’ substituent migrates from Sn2 to Al, while one Br migrates from Al to bridge the two Sn centers with 2.6875(5) Å (Sn1–Br3) and 2.8765(8) Å (Sn2–Br3) bond lengths. The Sn1 center adopts a nearly T-shaped geometry with Fe1–Sn1–C1 angle of 158.02(9)° and with the Fe1–Sn1 bond length of 2.3768(9) Å, which lies within the range of the reported iron-stannylene complexes (2.37–2.48 Å).^11,38^ The Sn2 center has a pyramidal environment, which is typical for three-coordinate Sn(II) centers with a stereochemically active electron lone pair. The Fe1–Sn2 (2.5249(6) Å) bond is considerably shorter than those of ferriostannylene complexes (2.56–2.60 Å).^11,17b,23^ IBO analysis corroborates the presence of Fe1–Sn1 and Fe1–Sn2 single bonds and the nonbonding electron lone pair on Sn2 (Figure S71). The ^119^Sn{^1^H} NMR spectrum of **3** displays two singlets at +15 ppm and +2393 ppm, which were assigned to Sn1 and Sn2, respectively, based on DFT calculations (Table S19). The high-frequency chemical shift for Sn2 indicates a low coordination number and is in agreement with the ^119^Sn{^1^H} NMR chemical shifts reported for ferriostannylene complexes.^11,23^ These structural features allow several possible Lewis-type descriptions of **3** (Scheme S1).

The reaction of **1** with CH_3_I afforded complex **4**, resulting from an oxidative addition of CH_3_I to Sn2 (Scheme 2). Complex **4** was isolated as dark red crystals in 67% yield. The solid-state molecular structure of **4** reveals a Sn(IV) center in tetrahedral geometry (Figure 4c). The Fe1–Sn2 bond length of 2.5188(5) Å lies in the expected range for a Fe–Sn(IV) single bond.^39^ The Sn1···Sn2 distance of 3.3804(5) Å is much longer than that in **1** (3.0596(6) Å), indicating no interaction between the Sn atoms (Figure S75). Notably, the Fe1–Sn1 bond length of 2.2960(4) Å and the Fe1–Sn1–C1 bond angle of 175.45(8)° are almost identical to those in **1** (2.2954(7) Å and 176.60(6)°; Table 1), indicating a similar bonding scenario. Indeed, the presence of an Fe1=Sn1 double bond and the zwitterionic character of **4**, which includes an [Ar’Sn1]^+^ moiety, are supported by the canonical MO, IBO, NPA and EDA-NOCV analyses (Figures S73, S74, and S82, Tables S8 and S18), as well as the MBO (MBO_Fe1–Sn1_: 1.68; Table S13). Hence, the Fe1–Sn1 bond is essentially unaffected by the oxidative addition of CH_3_I to Sn2. The ^119^Sn{^1^H} NMR resonances of **4** were detected at +768 ppm (Sn1) and +107 ppm (Sn2), which is consistent with an Fe=SnAr’ moiety and a tetrahedral Sn(IV) center, respectively.^11,39^ This reaction highlights the amphiphilic character of Sn2 in **1** and demonstrates the potential of **1** to form other complexes with Fe–Sn multiple bonds via the derivatization of the ferriostannylene moiety.

Treatment of **1** with excess PMe_3_ resulted in the clean conversion into complex **5**, which was isolated as dark red crystals in quantitative yield (Scheme 2). Intriguingly, PMe_3_ was found to coordinate to the Fe center, inducing the cleavage of the Fe1=Sn1 double bond and the formation of a Sn1–Sn2 bond, giving rise to a distannyne (Ar’SnSnAr’) ligand (*vide infra*). Monitoring the stoichiometric reaction of **1** with PMe_3_ by ^1^H NMR spectroscopy uncovered that the reaction is reversible. A van ‘t Hoff analysis based on ^1^H NMR data at various temperatures provides the thermodynamic parameters: Δ*H*° = −14.77 kcal mol^–1^, Δ*S*° = −32.53 cal mol^–1^ and Δ*G*° (298 K) = −5.08 kcal mol^–1^ (; Figure S77 and see SI for details). The *K*_eq_(298 K) was calculated to be 5.3 × 10^3^ M^–1^, indicating that the formation of **5** is favored.

The solid-state molecular structure of **5** shows the Ar’SnSnAr’ ligand coordinating to the iron center via one η^1^-Sn atom (Sn2) and one η^6^-Dipp group (Figure 4d). The three-coordinate Sn2 center adopts a planar geometry with a large Fe1–Sn2–Sn1 angle of 160.12(1)° and a Fe1–Sn2 bond length of 2.4864(7) Å. This Fe1–Sn2 bond is considerably shorter than the Fe1–Sn2 single bond in **1** (2.6207(7) Å; Table 1) and lies slightly above the range expected for iron-stannylene complexes (2.37–2.48 Å).^11,38^ In line with this relatively short Fe1–Sn2 bond, IBO and MBO (MBO_Fe–Sn1_: 1.34) analyses suggest double bond character, comprising both σ and π components (Figure S78 and Table S15). The zero-field ^57^Fe Mössbauer spectrum (80 K, solid state) of **5** reveals a doublet with δ = 0.50 mm s^–1^ and |Δ*E*_Q_| = 1.17 mm s^–1^, which is slightly lower than those of **2** (0.55 mm s^–1^) and **1** (0.66 mm s^–1^; Table 1). The ^119^Sn{^1^H} NMR spectrum of **5** exhibits two singlets at +2403 ppm and +1456 ppm, for Sn1 and Sn2, respectively, which is consistent with the presence of two-coordinate stannylene (Sn1) and metal-stannylene (Sn2) units.

The coordination of Lewis basic ligands, such as isocyanides,^40^ amines,^41^ imines^42^ and NHCs^43^ to distannynes, forming L → Sn σ-bonds, has been well established. By contrast, the coordination of distannynes to transition metals is underexplored. So far, only two distannyne-iron carbonyl complexes were reported, where the distannynes are intramolecularly stabilized by amine ligands and act as electron donors, forming Sn → Fe σ-bonds.^44^ Notably, the bonding of the distannyne in **5** is distinct, featuring an Fe1=Sn2 double bond. Moreover, the reversible cleavage and formation of an M–E multiple bond, as observed for **5** in solution, represents a new reaction pattern for heavier tetrylidyne complexes.

The reactivity of **1** toward small molecules, such as carbon disulfide (CS_2_), carbon dioxide (CO_2_), and white phosphorus (P_4_), was also investigated. The addition of one equivalent of CS_2_ to **1** in toluene at −30 °C immediately gave a red solution, from which complex **6** was isolated as red crystals in 58% yield (Scheme 2). The solid-state molecular structure of **6** shows the reductive coupling of two CS_2_ molecules, resulting in a C_2_S_4_ unit which bridges two FeSn_2_ clusters in a μ–κ^2^(S^1^,S^1’^):κ^2^(S^2^,S^2’^) fashion (Figure 4e). One pair of the S atoms (S1 and S3) is each bonded to a Sn atom, while the other pair (S2 and S4) bridges between Sn and Fe atoms. The C_2_S_4_ unit is planar, with a C3–C4 bond length of 1.355(3) Å, which is close to a typical C=C double bond.^24b^ The C–S bond lengths (1.762(2) Å to 1.786(2) Å) are characteristic of C–S single bonds.^24^ These structural features suggest the presence of an ethylene tetrathiolate (C_2_S_4_)^4–^ ligand in **6**. The ^1^H NMR spectrum (C_6_D_6_, r.t.) displays only one set of signals that can be assigned to two Ar’ groups. Additionally, its ^119^Sn{^1^H} NMR spectrum (C_6_D_6_, r.t.) exhibits only two singlets at +655 ppm and −124 ppm, supporting a *C*_2_ symmetry of **6** in solution on the NMR time scale. A few p-block element-mediated CS_2_ reductive coupling reactions have been reported,^45^ including three examples with stannylenes.^45b−45d^ In these cases, the (C_2_S_4_)^4–^ ligands were proposed to be formed via carbene intermediates and favor a μ–κ^2^(S^1^,S^2^):κ^2^(S^1’^,S^2’^) coordination mode (Figure S59). The formation of **6** is proposed to proceed through an initial [3 + 2] cycloaddition of CS_2_ to the Fe1=Sn1 double bond of **1**, assisted by Sn2, generating a carbene species, which then dimerizes to afford **6** (Scheme S2). This mechanism is underpinned by the reactivity of a disilene with CS_2_, where a [3 + 2]-cycloadduct with a bridging (C_2_S_4_)^4–^ unit was obtained.^46^

The ability of **1** to reduce CS_2_ motivated us to explore its reactivity toward CO_2_, a much less reactive inorganic molecule. Exposure of a benzene solution of **1** (dark brown) to a CO_2_ atmosphere (4 bar) led to an orange solution, from which complex **7** was isolated as orange crystals in 30% yield (Scheme 2). According to the solid-state molecular structure of this complex, **1** reacts with two equivalents of CO_2_, forming a CO and a CO_3_^2–^ ligand. The CO ligand coordinates to Fe, while the carbonate ligand bridges the two Sn atoms in a μ–κ^1^(O):κ^2^(O’,O’’) fashion. Two of these units are held together via a planar, centrosymmetric Fe_2_Sn_2_ ring (Figure 4f). Recently, Wesemann and coworkers reported the CO_2_ reduction with a cobalt-stannylidyne complex [(Me_3_P)_3_CoSnTbb] (Tbb = 4-*^t^*Bu-2,6-(CH(SiMe_3_)_2_)_2_-C_6_H_2_),^10c^ which is proposed to proceed via an initial [2 + 2]-cycloaddition of CO_2_ to the Co≡Sn triple bond. The Aldridge group has reported distannyne-mediated reductions of CO_2_, giving [Ar^R^Sn(μ–OCO_2_)SnAr^R^] (Ar^R^ = NCN pincer ligand) as products, where a bis(stannylene)oxide [(Ar^Et^Sn)_2_O] intermediate was identified.^47^ Given the simultaneous existence of a metal–tin multiple bond, the electron-rich nature of the Fe center, the electrophilic Sn centers, and the closely positioned Ar’Sn moieties in **1**, we propose that the first step in the reduction of CO_2_ by **1** occurs through a [2 + 2]-cycloaddition of CO_2_ to the Fe=Sn double bond, assisted by the second Sn atom (Scheme S3). This is followed by the splitting of the C=O bond, resulting in the formation of a carbonyl iron intermediate with a (Ar’Sn)_2_O ligand which then inserts a second equivalent of CO_2_ to yield the carbonate-bridged ligand Ar’Sn(μ–OCO_2_)SnAr’. Dimerization yields the final product **7**.

The distinct reactivity of **1** toward CO_2_ and CS_2_ is likely caused by the different polarity of C=O and C=S bonds; the more polarized C=O bond favors a [2 + 2]-cycloaddition with the Fe=Sn double bond of **1**, while CS_2_ favors [3 + 2]-cycloaddition. By contrast, the digermyne compound [(Ar(Me_3_Si)N)Ge]_2_ (Ar = 4-Me-2,6-(Ph_2_CH)_2_-C_6_H_2_) has been shown to reduce both CO_2_ and CS_2_ in the same manner.^48^

Complex **1** also activates the unpolar bonds of white phosphorus. The reaction of **1** with P_4_ proceeds smoothly in toluene at room temperature and furnishes complex **8**, which was isolated as orange crystals in quantitative yield (Scheme 2). The solid-state molecular structure of **8** reveals a P_4_Sn five-membered ring in an envelope conformation, coordinating η^4^ to iron via three P and one Sn atoms (Figure 4g). The (η^6^-arene)Ar’ substituent migrates from Sn2 to P2, and the Ar’Sn1 unit bridges two P atoms (P1 and P4) with bond lengths of 2.6657(5) Å (P1–Sn1) and 2.8070(6) Å (P4–Sn1). The P–P bond lengths in **8** are very similar and range from 2.1313(7) to 2.1666(7) Å, being typical for P–P single bonds.^24^ Both Sn centers adopt a trigonal pyramidal geometry with a Sn1···Sn2 distance of 3.1262(2) Å. The ^31^P{^1^H} NMR spectrum of **8** shows four multiplets of an AEMX spin system, with *J*_PP_ coupling constants ranging from 490 to 3.0 Hz. ^117/119^Sn satellites were detected for these multiplets with coupling constants ranging from 753 to 107 Hz (Figures S31 and S33, Table S1). In the ^119^Sn{^1^H} NMR spectrum, two multiplets due to the Sn–P coupling were detected at −454 ppm (Sn1) and −366 ppm (Sn2; Figures S32 and S34, Table S19). P_4_ activation by heavier tetryl elements remains limited.^49^ To our knowledge, only two examples of low-valent tin compounds, the distannyne [(DippNC(CH_3_))_2_C_6_H_3_Sn]_2_^42^ and the stannylene [Ar^#^Sn(Si*^t^*Bu_3_)],^50^ have been reported to react with P_4_ to give butterfly like P_4_ units via cleavage of one P–P bond. By contrast, the reaction of the Co_2_Sn_2_ cluster [Co(SnAr’)]_2_ with P_4_ results in a P_4_ chain,^17c^ while a heterobimetallic CoSn cluster reacting with P_4_ gave the triple-decker cobalt complex [(^*t*^Bu_2_C_2_P_2_)_2_Co_2_(μ,η^5^:η^5^-SnP_4_)] with a planar P_4_Sn unit.^51^

## Conclusion

In summary, we synthesized and characterized [Fe(SnAr’)_2_] (**1**), the first complex to feature both ferriostannylene and stannylidyne functionalities. The combination of iron and two monosubstituted tin centers, along with the constrained ligand structure, forms an Fe–Sn single bond and an Fe=Sn double bond (σ+π). This arrangement results in a positively charged Sn center and an electron-rich Fe center. The unusual coordination environment effectively reduces the tendency of the two aryl-tin moieties to dimerize, thereby maintaining the high reactivity of the two-coordinate Sn centers. Additionally, the proximity of the metal centers enhances cooperative reactivity.

Comprehensive spectroscopic characterization and theoretical calculations support the existence of an amphiphilic FeSn_2_ core with multiple reactive sites. This was demonstrated in the reactions of **1** with Ni(COD)_2_, AlBr_3_, CH_3_I, and PMe_3_. The lone-pair electrons and vacant p-orbital of the two Sn atoms in **1** create “push-pull” interactions with nickel due to their optimal distance and orientation. In the reaction with AlBr_3_, the cleavage of the Al–Br bonds was observed, accompanied by the migration of Ar’. The formation of a **1**·AlBr_3_ adduct may initiate this reaction. The oxidative addition of CH_3_I leads to the formation of a Sn(IV) center, illustrating the amphiphilic nature of the ferriostannylene site. Interestingly, PMe_3_ binds to the iron center, resulting in the cleavage of the Fe=Sn double bond and the formation of a Sn–Sn single bond. Notably, this process is reversible. Furthermore, compound **1** activates CS_2_ and CO_2_ through different reaction pathways, and P_4_, which leads to the cleavage of multiple P–P bonds.

Our results emphasize the significant synthetic potential of complexes that feature multiple coordinatively unsaturated sites from group 14 elements. Complexes similar to **1** should be easily obtainable using comparable strategies with other low-valent transition metalates^52^ and appropriate main group precursors. This bodes well for future investigation pursuing further challenging synergistic bond activation reactions. Studies in this direction are in hand.

## Supporting Information Available

Experimental details and synthetic procedures, further spectroscopic and analytical data, crystallographic information, and details of DFT calculation (PDF)
