# Supplementary material for: Synthesis and Reactivity of an Iron–Tin Complex with Adjacent Stannylidyne and Ferriostannylene Units
Source: J Am Chem Soc. 2025 Feb 11;147(8):7083–93. doi: 10.1021/jacs.4c18423 (PMC11869287; doi:10.1021/jacs.4c18423)
Supplement: Supplementary file 1 — ja4c18423_si_001.pdf [file ja4c18423_si_001.pdf]

Supporting Information

Synthesis and Reactivity of an Iron-Tin Complex with Adjacent Stannylidyne and Ferriostannylene Units

Yang Liu,[a] Franz F. Westermair,[b] Isabelle Becker,[c] Sebastian Hauer,[a] Michael Bodensteiner,[a] Christoph Hennig,[d,e] Gábor Balázs,[a] Franc Meyer,[c] Ruth M. Gschwind,[b] and Robert Wolf[a]*

[a]Institute of Inorganic Chemistry, University of Regensburg, 93040 Regensburg, Germany

[b]Institute of Organic Chemistry, University of Regensburg, 93040 Regensburg, Germany

[c]Institute of Inorganic Chemistry, University of Göttingen, Tammannstraße 4, 37077 Göttingen, Germany

[d]European Synchrotron Radiation Facility, Rossendorf Beamline (BM20-CRG), 38043 Grenoble, France

[e]Institute of Resource Ecology, Helmholtz-Zentrum Dresden-Rossendorf, 01314 Dresden, Germany

robert.wolf@ur.de

Table of Contents

Experimental Procedures 3
General Considerations 3
Synthesis of Complexes **1**-**8** 3
NMR Spectra 10
NMR Simulations 26
^{1}H NMR Spectroscopy and van't Hoff Analysis of the Reaction of **1** with PMe_3 28
UV-vis Spectra 30
LIFDI-Mass Spectra 34
IR Spectrum of **7** 36
ICP-OES Spectroscopy of **1** and **2** 37
Zero-field ^{57}Fe Mössbauer Measurements 38
Crystallographic Data 39
Proposed Mechanism for the Formation of **3**, **6** and **7** 46
DFT Calculations 47
Natural Population Analysis (NPA) 50
Energy Decomposition Analysis with Natural Orbitals for Chemical Valency (EDA-NOCV) 58
Calculation of the ^{119}Sn NMR Chemical Shifts 60
References 74

Experimental Procedures

General Considerations

All manipulations of air and moisture sensitive materials were performed under an atmosphere of dry dinitrogen with rigorous exclusion of air and moisture using standard Schlenk techniques, or in an argon-filled glovebox. Solvents were purified, dried, and degassed with an MBraun SPS800 solvent purification system. All dry solvents except *n*-hexane and *n*-pentane were stored under argon over activated 3 Å molecular sieves in gas-tight Schlenk tubes. *n*-Hexane and *n*-pentane were stored over a potassium mirror. d_6-Benzene (C_6D_6) and d_8-tetrahydrofuran (d_8-THF) were dried over activated 3 Å molecular sieves. [Ar'SnCl]$_2$ (Ar' = 2,6-Dipp$_2$-C_6H_3, Dipp = 2,6-^{i}Pr$_2$-C_6H_3)[1], [K(18-c-6)][Fe(η^4-anthracene)$_2$] (18-c-6 = 18-crown-6)[2] and [Li(DME)]$_2$[Fe(COD)$_2$] (DME = 1,2-Dimethoxyethane, COD = 1,5-cyclooctadiene)[3] were synthesized according to literature procedures. All other chemicals were purchased from Merck, and solid chemicals ([Ni(COD)$_2$] and ArBr$_3$) were used as received and liquid chemicals (CH_3I, PMe_3 and CS_2) were further dried over activated 3 Å molecular sieves. CO_2 gas (grade 4.8) was purchased from Linde-Gas and used as received. ^{1}H, ^{13}C{^{1}H}, ^{31}P{^{1}H} and ^{119}Sn{^{1}H} NMR spectra were recorded with Bruker Avance 400 MHz, 500 MHz, or 600 MHz spectrometer. All chemical shifts are reported in units of ppm and referenced to the residual ^{1}H NMR signals of the deuterated solvents for proton chemical shifts, the ^{13}C{^{1}H} NMR signal of deuterated solvents for carbon chemical shifts, the ^{31}P{^{1}H} NMR signal of 85% phosphorous acid (external standard) for phosphorous chemical shifts, and the ^{119}Sn{^{1}H} NMR signal of $SnMe_4$ (external standard) for tin chemical shifts. The assignments of the ^{1}H and ^{13}C{^{1}H} NMR signals were confirmed by two-dimensional (COSY, NOESY, HSQC, HMBC) and DEPT experiments. UV-vis spectra were recorded on an Ocean Optics DH-2000-BAL Spectrometer. Elemental analyses were determined by the analytical department of the University of Regensburg with the vario MICRO cube instrument. HR-LIFDI-mass spectra were recorded on an Agilent Q-TOF 6540 UHD device by the analytical department of the University of Regensburg. IR spectra were recorded with a Bruker ALPHA spectrometer equipped with a diamond ATR unit.

Synthesis of Complexes 1-8

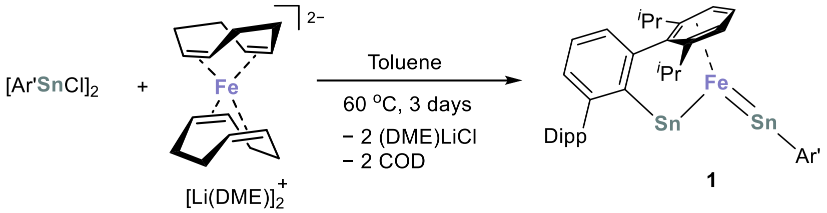

Synthesis of [Fe(SnAr')$_2$] (1). To a toluene (20 mL) solution of [Ar'SnCl]$_2$ (110 mg, 0.1 mmol) in a Schlenk tube (with high vacuum valve plug) was added [Li(DME)]$_2$[Fe(COD)$_2$] (60 mg, 0.13 mmol) at room temperature. The resulting dark green solution was warmed up to 60 °C and stirred for 3 days. During this time the color of the solution changed slowly from dark green to dark brown. After removing the solvent under vacuo, the black solid was washed with *n*-hexane (3 × 2 mL) and then extracted into benzene (3 × 3 mL). Slow evaporation of this benzene solution at room temperature afforded the product **1** as dark brown crystals. Yield: 80 mg, 73%. **^{1}H NMR** (400 MHz, C_6D_6, 298 K): δ (ppm) 0.63 (d, *J* = 6.8 Hz, 6H, −CH(C*H*$_3$)$_2$ of η^6-Dipp), 1.02 (d, *J* = 6.8 Hz, 6H, −CH(C*H*$_3$)$_2$ of Dipp of Ar'(Sn−Fe)), 1.06 (d, *J* = 6.8 Hz, 6H, −CH(C*H*$_3$)$_2$ of η^6-Dipp), 1.11 (d, *J* = 6.8 Hz, 12H, −CH(C*H*$_3$)$_2$ of Dipp-Ar'(Sn=Fe)), 1.23 (d, *J* = 6.8 Hz, 6H, −CH(C*H*$_3$)$_2$ of Dipp of Ar'(Sn−Fe)), 1.33 (d, *J* = 6.8 Hz, 12H, −CH(C*H*$_3$)$_2$ of Dipp-Ar'(Sn=Fe)), 2.37 (sept, *J* = 6.8 Hz, 2H, −C*H*(CH$_3$)$_2$ of η^6-Dipp), 3.03 (sept, *J* = 6.8 Hz, 4H, −C*H*(CH$_3$)$_2$ of Dipp-Ar'(Sn=Fe)), 3.07 (sept, *J* = 6.8 Hz, 2H, −C*H*(CH$_3$)$_2$ of Dipp of Ar'(Sn−Fe)), 4.75 (d, *J* = 6.0 Hz, 2H, *m*-C*H*-η^6-Dipp), 5.62 (t, *J* = 6.0 Hz, 1H, *p*-C*H*-η^6-Dipp), 7.09–7.15 (m, 6H, *m, p*-C*H*-Dipp of Ar'(Sn=Fe)), 7.20 (d, *J* = 7.6 Hz, 2H, *m*-C*H*-Dipp of Ar'(Sn=Fe)), 7.28

(d, *J* = 7.6 Hz, 2H, *m*-C*H*-Dipp of Ar’(Sn–Fe)), 7.33 (dd, *J* = 7.2, 8.0 Hz, 1H, *p*-C*H*-Dipp of Ar’(Sn=Fe)), 7.43 (t, *J* = 8.0 Hz, 1H, *p*-C*H*-Dipp of Ar’(Sn–Fe)), 7.46 (t, *J* = 7.2 Hz, 1H, *p*-C*H*-Ar’(Sn–Fe)), 7.53 (dd, *J* = 7.6, 1.2 Hz, 1H, *m*-C*H*-Ar’(Sn–Fe)), 7.74 (dd, *J* = 7.2, 1.2 Hz, 1H, *m*-C*H*-Ar’(Sn–Fe)). **^{13}C{^{1}H} NMR** (100 MHz, C_6D_6, 298 K): *δ* (ppm) 174.8 (C_{ipso}–Sn–Fe), 173.0 (C_{ipso}–Sn=Fe), 150.4 (*o*-*C*-Ar’(Sn–Fe)), 147.4 (*o*-*C*-Dipp of Ar’(Sn=Fe)), 146.8 (*o*-*C*-Dipp of Ar’(Sn–Fe)), 145.3 (*o*-*C*-Ar’(Sn=Fe)), 145.0 (*o*-*C*-Ar’(Sn–Fe)), 143.7 (Dipp-*C*–Ar’(Sn–Fe)), 139.3 (Dipp-*C*–Ar’(Sn=Fe)), 131.9 (*m*-*C*-Ar’(Sn–Fe)), 130.0 (*p*-*C*-Dipp of Ar’(Sn=Fe)), 129.2 (*m*-*C*-Ar’(Sn=Fe)), 128.0 (*p*-*C*-Ar’(Sn=Fe)), 127.9 (*p*-*C*-Dipp of Ar’(Sn–Fe)), 127.6 (*m*-*C*-Ar’(Sn–Fe)), 126.2 (*p*-*C*-Ar’(Sn–Fe)), 124.2 (*m*-*C*-Dipp of Ar’(Sn=Fe)), 122.7 (*m*-*C*-Dipp of Ar’(Sn–Fe)), 112.0 (*o*-*C*- η^6-Dipp), 110.4 (η^6-Dipp-*C*–Ar’(Sn–Fe)), 80.9 (*p*-*C*-η^6-Dipp), 76.9 (*m*-*C*- η^6-Dipp), 31.1 (–*C*H(CH_3)$_2$ of Dipp-Ar’(Sn=Fe)), 31.0 (–*C*H(CH_3)$_2$ of Dipp-Ar’(Sn–Fe)), 29.1 (–*C*H(CH_3)$_2$ of η^6-Dipp), 25.7 (–CH(*C*H$_3$)$_2$ of Dipp-Ar’(Sn=Fe)), 25.4 (–CH(*C*H$_3$)$_2$ of η^6-Dipp), 24.9 (–CH(*C*H$_3$)$_2$ of Dipp-Ar’(Sn–Fe)), 24.4 (–CH(*C*H$_3$)$_2$ of η^6-Dipp), 23.8 (–CH(*C*H$_3$)$_2$ of Dipp-Ar’(Sn–Fe)), 23.6 (–CH(*C*H$_3$)$_2$ of Dipp-Ar’(Sn=Fe)). **^{119}Sn{^{1}H} NMR** (224 MHz, *d*$_8$-THF, 298 K): *δ* (ppm) 3229 (s, Ar’*Sn*–Fe), 466 (s, Ar’*Sn*=Fe). **Anal.** Calcd. for $C_{60}H_{74}Sn_2Fe{\cdot}C_6H_6$: C 67.95, H 6.91; Found: C 67.82, H 7.28. **HR-LIFDI-MS** (toluene) m/z (%): 1088.2841 (100) for $[C_{60}H_{74}Sn_2Fe]^+$. **Absorption spectrum** (toluene, 298 K): λ_{max}, nm (*ε*, M^{-1} cm^{-1}) 450 (3600), 590 (shoulder, 1280), 730 (broad, 390). **^{57}Fe Mössbauer** (solid state, 80 K): δ = 0.61 mm s^{-1}, $|\Delta E_Q|$ = 0.66 mm s^{-1}.

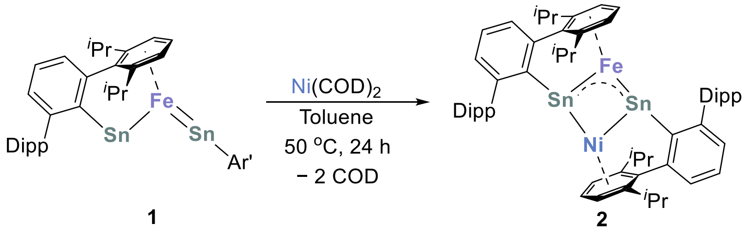

Synthesis of [(Ar’Sn)$_2$FeNi] (2). To a dark brown benzene (10 mL) solution of **1** (55 mg, 0.05 mmol) in a Schlenk tube (with high vacuum valve plug) was added [Ni(COD)$_2$] (60 mg, 0.22 mmol) at room temperature. The reaction mixture was warmed up to 50 °C and then let to stay without stirring in the oil bath for 24 h under exclusion of light. During this time, the color of the solution changed slowly from dark brown to dark green, along with the formation of a nickel mirror on the wall of the Schlenk tube (due to the thermal decomposition of [Ni(COD)$_2$]). The reaction mixture was cooled to room temperature and let to stay without stirring for two days, which afforded dark green crystals of **2** at the bottom of the Schlenk tube. The crystals were collected by decanting the supernatant. After removing the solvent from the supernatant, the resulting green residue was extracted into toluene (3 × 1 mL) which was then stored at −30 °C for 2 days to give the second batch of crystals. Total yield: 40 mg, 69%. **^{1}H NMR** (400 MHz, *d*$_8$-THF, 298 K): *δ* (ppm) 0.59 (d, *J* = 7.2 Hz, 6H, –CH(C*H*$_3$)$_2$ of η^6-Dipp-Fe/Ni), 0.93 (d, *J* = 6.8 Hz, 6H, –CH(C*H*$_3$)$_2$ of η^6-Dipp-Fe/Ni), 1.00 (d, *J* = 6.8 Hz, 6H, –CH(C*H*$_3$)$_2$ of Dipp-Ar’), 1.03 (d, *J* = 6.8 Hz, 6H, –CH(C*H*$_3$)$_2$ of η^6-Dipp-Fe/Ni), 1.14 (d, *J* = 6.8 Hz, 6H, –CH(C*H*$_3$)$_2$ of Dipp-Ar’), 1.15 (d, *J* = 6.8 Hz, 6H, –CH(C*H*$_3$)$_2$ of Dipp-Ar’), 1.19 (d, *J* = 6.8 Hz, 6H, –CH(C*H*$_3$)$_2$ of η^6-Dipp-Fe/Ni), 1.37 (d, *J* = 6.8 Hz, 6H, –CH(C*H*$_3$)$_2$ of Dipp-Ar’), 2.06 (sept, *J* = 6.8 Hz, 2H, –C*H*(CH_3)$_2$ of η^6-Dipp-Fe/Ni), 2.40 (sept, *J* = 6.8 Hz, 2H, –C*H*(CH_3)$_2$ of η^6-Dipp-Fe/Ni), 2.81 (sept, *J* = 6.8 Hz, 2H, –C*H*(CH_3)$_2$ of Dipp-Ar’), 2.82 (sept, *J* = 6.8 Hz, 2H, –C*H*(CH_3)$_2$ of Dipp-Ar’), 4.05 (d, *J* = 6.0 Hz, 2H, *m*-C*H*-η^6-Dipp-Fe/Ni), 4.96 (t, *J* = 6.0 Hz, 1H, *p*-C*H*-η^6-Dipp-Fe/Ni), 5.01 (t, *J* = 7.2 Hz, 1H, *p*-C*H*-η^6-Dipp-Fe/Ni), 5.94 (d, *J* = 7.2 Hz, 2H, *m*-C*H*-η^6-Dipp-Fe/Ni), 7.21 (d, *J* = 7.6 Hz, 2H, *m*-C*H*-Dipp-Ar’), 7.24 (d, *J* = 8.0 Hz, 1H, *m*-C*H*-Ar’), 7.32–7.39 (m, 3H, *m*-C*H*-Ar’, *p*- C*H*-Dipp-Ar’), 7.44 (d, *J* = 7.6 Hz, 2H, *m*-C*H*-Dipp-Ar’), 7.48–7.59 (m, 3H, *p*-C*H*-Ar’, *p*-C*H*-Dipp-Ar’), 7.93 (dd, *J* = 7.6, 1.2 Hz, 1H, *m*-C*H*-Ar’). **^{13}C{^{1}H} NMR** (100 MHz, *d*$_8$-THF, 298 K): *δ* (ppm) 166.2 (*o*-*C*-Ar’), 162.2 (*o*-*C*-Ar’), 151.4 (Ar’-*C*-Sn), 148.1 (*o*-*C*-Dipp), 147.4 (*o*-*C*-Dipp), 145.6 (*o*-*C*-Ar’), 144.7 (Dipp-*C*-Ar’), 143.3 (*o*-*C*-Ar’), 142.6 (Ar’-*C*-Sn), 139.3 (Dipp-*C*-Ar’), 129.3 (*p*-*C*H-Ar’ or *p*-*C*H-Dipp-Ar’), 128.9 (*p*-*C*H-Ar’ or *p*-*C*H-Dipp-

Ar'), 128.6 (*m*-*C*H-Ar'), 128.3 (*m*-*C*H-Ar'), 127.8 (*m*-*C*H-Ar' or *p*-*C*H-Dipp-Ar'), 127.2 (*p*-*C*H-Ar' or *p*-*C*H-Dipp-Ar'), 127.1 (*m*-*C*H-Ar' or *p*-*C*H-Dipp-Ar'), 126.5 (*m*-*C*H-Ar' or *p*-*C*H-Dipp-Ar'), 123.7 (*m*-*C*H-Dipp-Ar'), 122.9 (*m*-*C*H-Dipp-Ar'), 120.6 (*o*-*C*-η^6-Dipp), 116.7 (η^6-Dipp-*C*-Ar'), 116.2 (η^6-Dipp-*C*-Ar'), 107.6 (*o*-*C*-η^6-Dipp), 102.2 (*p*-*C*H-η^6-Dipp-Fe/Ni), 99.7 (*m*-*C*H-η^6-Dipp-Fe/Ni), 79.5 (*p*-*C*H-η^6-Dipp-Fe/Ni), 74.0 (*m*-*C*H-η^6-Dipp-Fe/Ni), 31.5 (−*C*H(CH$_3$)$_2$ of Dipp-Ar'), 31.2 (−*C*H(CH$_3$)$_2$ of Dipp-Ar'), 29.8 (−*C*H(CH$_3$)$_2$ of η^6-Dipp-Fe/Ni), 29.7 (−*C*H(CH$_3$)$_2$ of η^6-Dipp-Fe/Ni), 25.5 (−CH(*C*H$_3$)$_2$ of η^6-Dipp-Fe/Ni), 25.3 (−CH(*C*H$_3$)$_2$ of Dipp-Ar'), 24.7 (−CH(*C*H$_3$)$_2$ of Dipp-Ar'), 24.4 (−CH(*C*H$_3$)$_2$ of η^6-Dipp-Fe/Ni), 24.4 (−CH(*C*H$_3$)$_2$ of η^6-Dipp-Fe/Ni), 24.3 (−CH(*C*H$_3$)$_2$ of η^6-Dipp-Fe/Ni), 24.0 (−CH(*C*H$_3$)$_2$ of Dipp-Ar'), 23.9 (−CH(*C*H$_3$)$_2$ of Dipp-Ar'). **^{119}Sn{^{1}H} NMR** (224 MHz, d_8-THF, 298 K): δ (ppm) 1701 (s, (η^6-Dipp-Fe)Ar'*Sn*), 369 (s, (η^6-Dipp-Ni)Ar'*Sn*). **Anal.** Calcd. for $C_{60}H_{74}Sn_2FeNi{\cdot}0.5C_6H_6$: C 63.79, H 6.54; Found: C 63.48, H 6.65. HR-LIFDI-MS (toluene) m/z (%): 1146.2473 (100) for $[C_{60}H_{74}Sn_2FeNi]^+$. **Absorption spectrum** (toluene, 298 K): λ_{max}, nm (ε, M^{-1} cm^{-1}) 400 (12700), 565 (3360), 590 (3200), 660 (2400). **^{57}Fe Mössbauer** (solid state, 80 K): δ = 0.55 mm s^{-1}, $|\Delta E_Q|$ = 0.76 mm s^{-1}.

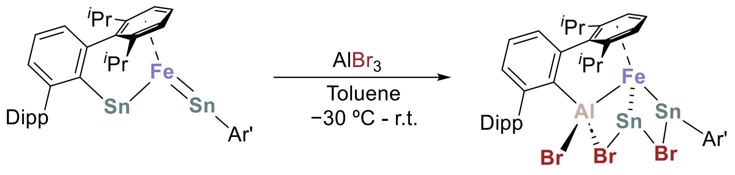

1 3

Synthesis of [(Ar'AlBr$_2$)(Ar'SnBr)FeSn] (3). To a dark brown toluene (10 ml) solution of **1** (55 mg, 0.05 mmol) at −30 °C was added solid AlBr$_3$ (13.3 mg, 0.05 mmol). The color of the solution turned maroon immediately. The reaction mixture was allowed to warm to room temperature and was stirred for 30 min. The maroon solution was concentrated under vacuum until incipient precipitation (*ca.* 5 ml) and then filtered. The solution was stored at −30 °C for 1 week to give the maroon crystals of **3**. Yield: 57 mg, 84%. **^{1}H NMR** (500 MHz, C_6D_6, 298 K): δ (ppm) 0.75 (d, *J* = 6.7 Hz, 3H, −CH(C*H*$_3$)$_2$ of η^6-Dipp-Fe), 0.92 (d, *J* = 6.9 Hz, 6H, −CH(C*H*$_3$)$_2$ of Dipp-Ar'Sn), 0.93 (d, *J* = 6.5 Hz, 3H, −CH(C*H*$_3$)$_2$ of η^6-Dipp-Fe), 0.99 (d, *J* = 6.7 Hz, 6H, −CH(C*H*$_3$)$_2$ of Dipp-Ar'Sn), 1.10 (d, *J* = 6.9 Hz, 3H, −CH(C*H*$_3$)$_2$ of η^6-Dipp-Fe), 1.13 (d, *J* = 6.7 Hz, 3H, −CH(C*H*$_3$)$_2$ of Dipp-Ar'Al), 1.17 (d, *J* = 6.7 Hz, 3H, −CH(C*H*$_3$)$_2$ of Dipp-Ar'Al), 1.22 (d, *J* = 7.1 Hz, 3H, −CH(C*H*$_3$)$_2$ of η^6-Dipp-Fe), 1.38 (d, *J* = 6.8 Hz, 6H, −CH(C*H*$_3$)$_2$ of Dipp-Ar'Sn), 1.46 (d, *J* = 6.7 Hz, 6H, −CH(C*H*$_3$)$_2$ of Dipp-Ar'Sn), 1.53 (d, *J* = 6.8 Hz, 3H, −CH(C*H*$_3$)$_2$ of Dipp-Ar'Al), 1.60 (d, *J* = 6.8 Hz, 3H, −CH(C*H*$_3$)$_2$ of Dipp-Ar'Al), 2.27 (sept, *J* = 7.0 Hz, 1H, −C*H*(CH$_3$)$_2$ of η^6-Dipp-Fe), 2.51 (sept, *J* = 7.0 Hz, 1H, −C*H*(CH$_3$)$_2$ of η^6-Dipp-Fe), 2.95 (sept, *J* = 7.0 Hz, 1H, −C*H*(CH$_3$)$_2$ of Dipp-Ar'Al), 3.04 (sept, *J* = 7.0 Hz, 1H, −C*H*(CH$_3$)$_2$ of Dipp-Ar'Al), 3.06 (broad, 2H, −C*H*(CH$_3$)$_2$ of Dipp-Ar'Sn), 3.21 (broad, 2H, −C*H*(CH$_3$)$_2$ of Dipp-Ar'Sn), 4.55 (d, *J* = 5.9 Hz, 1H, *m*-C*H*-η^6-Dipp-Fe), 4.64 (d, *J* = 6.1 Hz, 1H, *m*-C*H*-η^6-Dipp-Fe), 5.20 (t, *J* = 6.0 Hz, 1H, *p*-C*H*-η^6-Dipp-Fe), 7.04 (t, *J* = 7.5 Hz, 1H), 7.13 – 7.15 (m, 1H), 7.17 – 7.21 (m, 3H), 7.21 – 7.28 (m, 4H), 7.28 – 7.31 (m, 1H), 7.33 (dd, *J* = 7.5, 1.5 Hz, 1H, *m*-C*H*-Dipp-Ar'Al), 7.36 (t, *J* = 8.0 Hz, 1H). Due to overlapping signals in the aromatic region, the full assignment of the signals of the aryl rings is difficult. Signals of two H atoms on aryl rings overlap with the residual solvent peak of C_6D_6. **^{13}C{^{1}H} NMR** (125 MHz, C_6D_6, 298 K): δ (ppm) 165.1, 148.4 (*o*-*C*-Dipp-Ar'Sn), 148.1 (Dipp-*C*-Ar'Sn), 146.6 (*o*-*C*-Dipp-Ar'Al), 146.5 (*o*-*C*-Dipp-Ar'Al), 144.2, 143.6, 140.7 (Dipp-*C*-Ar'Al), 139.3, 132.6, 131.0, 130.1 (*m*-*C*H-Ar'), (*p*-*C*H-Ar'), 128.5, 128.4, 127.8 (*p*-*C*-Dipp-Ar'Al), 127.6, 126.2, 125.3, 124.4, 123.4, 122.8 (*m*-*C*-Dipp-Ar'Al), 122.4 (*m*-*C*-Dipp-Ar'Al), 114.3 (η^6-Dipp-*C*-Ar'), 114.1 (*o*-*C*-η^6-Dipp-Fe), 103.9 (*o*-*C*-η^6-Dipp-Fe), 80.61 (*m*-*C*H-η^6-Dipp-Fe), 80.58 (*m*-*C*H-η^6-Dipp-Fe), 78.1 (*p*-*C*H-η^6-Dipp-Fe), 31.4 (−C*H*(CH$_3$)$_2$ of η^6-Dipp-Fe), 31.2 (−*C*H(CH$_3$)$_2$ of Dipp-Ar'Sn), 31.1 (2C, −*C*H(CH$_3$)$_2$ of Dipp-Ar'Sn/−*C*H(CH$_3$)$_2$ of Dipp-Ar'Sn), 31.0 (−*C*H(CH$_3$)$_2$ of Dipp-Ar'Al), 29.2 (−*C*H(CH$_3$)$_2$ of η^6-Dipp-Fe), 27.8 (−CH(*C*H$_3$)$_2$ of η^6-Dipp-Fe), 27.2 (−CH(*C*H$_3$)$_2$ of η^6-Dipp-Fe), 26.9 (−CH(*C*H$_3$)$_2$ of Dipp-Ar'Al), 26.6 (−CH(*C*H$_3$)$_2$ of Dipp-Ar'Al), 26.4 (broad, −CH(C*H*$_3$)$_2$ of Dipp-Ar'Sn), 25.9 (broad, −CH(*C*H$_3$)$_2$ of Dipp-Ar'Sn), 25.0

(−CH(*C*H$_3$)$_2$ of η^6-Dipp-Fe), 23.9 (−CH(*C*H$_3$)$_2$ of Dipp-Ar'Sn), 23.5 (−CH(*C*H$_3$)$_2$ of Dipp-Ar'Sn), 23.4 (−CH(*C*H$_3$)$_2$ of Dipp-Ar'Al), 23.2 (−CH(*C*H$_3$)$_2$ of Dipp-Ar'Al), 22.7 (−CH(*C*H$_3$)$_2$ of η^6-Dipp-Fe). **^{119}Sn{^{1}H} NMR** (224 MHz, C_6D_6, 298 K): δ (ppm) 2393 (s, Fe*Sn*), 14.8 (s, Fe*Sn*Ar'(Br)). **Anal.** Calcd. for $C_{60}H_{74}Sn_2FeAlBr_3{\cdot}0.5C_6H_7$: C 54.43, H 5.61; Found: C 54.28, H 5.93. **Absorption spectrum** (toluene, 298 K): λ_{max}, nm (ε, M^{-1} cm^{-1}) 390 (shoulder, 4150), 480 (shoulder, 2480).

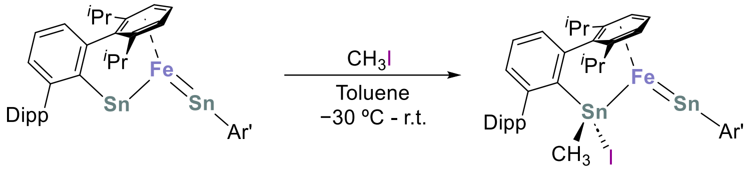

1 4

Synthesis of [(Ar'(CH$_3$)SnI)Fe(SnAr')] (4). To a dark brown toluene (10 ml) solution of **1** (55 mg, 0.05 mmol) at −30 °C was added pre-cooled CH_3I (7.1 mg, 0.05 mmol). The color of the solution turned dark red immediately. The reaction solution was allowed to warm to room temperature and was stirred for 2 h. After removing the solvent under vacuo, the resulting red solid was washed by *n*-pentane (3 × 1 mL) and then dissolved in toluene (*ca.* 2 ml). Storage at −30 °C for 2 days gave dark red crystals of **4**. Yield: 42 mg, 67%. **^{1}H NMR** (500 MHz, C_6D_6, 298 K): δ (ppm) 0.31 (s + satellites, $^2J_{SnH}$ = 38.5 Hz, 3H, C*H*$_3$SnI), 0.98 (d, *J* = 6.6 Hz, 6H, −CH(C*H*$_3$)$_2$ of η^6-Dipp-Fe), 1.01 (d, *J* = 6.8 Hz, 12H, −CH(C*H*$_3$)$_2$ of Dipp-Ar'Sn), 1.07 (d, *J* = 6.7 Hz, 6H, −CH(C*H*$_3$)$_2$ of Dipp-Ar'(CH$_3$)SnI), 1.12 (d, *J* = 6.9 Hz, 6H, −CH(C*H*$_3$)$_2$ of η^6-Dipp-Fe), 1.44 (d, *J* = 6.9 Hz, 12H, −CH(C*H*$_3$)$_2$ of Dipp-Ar'Sn), 1.51 (d, *J* = 6.7 Hz, 6H, −CH(C*H*$_3$)$_2$ of Dipp-Ar'(CH$_3$)SnI), 2.30 (sept, *J* = 6.5 Hz, 2H, −C*H*(CH$_3$)$_2$ of η^6-Dipp-Fe), 2.93 (sept, *J* = 6.5 Hz, 4H, −CH(C*H*$_3$)$_2$ of Dipp-Ar'Sn), 3.12 (sept, *J* = 6.5 Hz, 2H, −C*H*(CH$_3$)$_2$ of of Dipp-Ar'(CH$_3$)SnI), 5.46 (s, 3H, *m*-C*H*-η^6-Dipp-Fe/*p*-C*H*-η^6-Dipp-Fe), 6.89 (d, *J* = 7.5 Hz, 2H, *m*-C*H*-Ar'Sn), 7.17 – 7.22 (m, 5H, *m*-C*H*-Dipp-Ar'Sn/*p*-C*H*-Ar'Sn), 7.23 (t, *J* = 7.5 Hz, 1H, *p*-C*H*-Ar'(CH$_3$)SnI), 7.28 (t, 2H, *J* = 7.5 Hz, *p*-C*H*-Dipp-Ar'Sn), 7.32 (d, *J* = 7.5 Hz, 2H, *m*-C*H*-Dipp-Ar'(CH$_3$)SnI), 7.35–7.42 (m, 3H, *p*-C*H*-Dipp-Ar'(CH$_3$)SnI/*m*-C*H*-Ar'(CH$_3$)SnI). **^{13}C{^{1}H} NMR** (100 MHz, C_6D_6, 298 K): δ (ppm) 178.4 (*o*-*C*-Ar'Sn), 156.1 (*o*-*C*-Ar'(CH$_3$)SnI), 148.1 (*o*-*C*-Dipp-Ar'Sn), 147.2 (*o*-*C*-Dipp-Ar'(CH$_3$)SnI), 145.6 (Ar'-*C*-Sn(CH$_3$)I), 143.0 (*o*-*C*-Ar'(CH$_3$)SnI), 142.6 (Ar'-*C*-Sn), 140.6 (Dipp-*C*-Ar'(CH$_3$)SnI), 137.0 (Dipp-*C*-Ar'Sn), 131.1 (*p*-*C*-Ar'(CH$_3$)SnI), 130.7 (*p*-C*H*-Dipp-Ar'Sn), 130.4 (*m*-*C*H-Ar'Sn), 128.5 (*p*-*C*H-Dipp-Ar'(CH$_3$)SnI), 127.9 (*p*-*C*H-Ar'Sn), 126.74/126.66 (*m*-*C*H-Ar'(CH$_3$)SnI), 124.4 (*m*-*C*H-Dipp-Ar'Sn), 123.0 (*m*-*C*H-Dipp-Ar'(CH$_3$)SnI), 116.1 (η^6-Dipp-*C*-Ar'Sn), 109.9 (*o*-*C*-η^6-Dipp-Fe), 76.6 (*m*-*C*H-η^6-Dipp-Fe), 75.9 (*p*-*C*H-η^6-Dipp-Fe), 31.1 (−*C*H(CH$_3$)$_2$ of Dipp-Ar'Sn), 30.9 (−*C*H(CH$_3$)$_2$ of Dipp-Ar'(CH$_3$)SnI), 28.9 (−*C*H(CH$_3$)$_2$ of η^6-Dipp-Fe), 26.4 (−CH(*C*H$_3$)$_2$ of Dipp-Ar'(CH$_3$)SnI), 26.0 (−CH(*C*H$_3$)$_2$ of η^6-Dipp-Fe), 25.1 (−CH(*C*H$_3$)$_2$ of Dipp-Ar'Sn), 24.0 (−CH(*C*H$_3$)$_2$ of Dipp-Ar'Sn), 23.9 (−CH(*C*H$_3$)$_2$ of η^6-Dipp-Fe), 23.8 (−CH(*C*H$_3$)$_2$ of Dipp-Ar'(CH$_3$)SnI), 10.3 (*C*H$_3$SnI). **^{119}Sn{^{1}H} NMR** (224 MHz, C_6D_6, 298 K): δ (ppm) 768 (s + satellites, $^2J_{Sn\text{-}Sn}$ = 5448 Hz, Fe*Sn*Ar'), 107 (s + satellites, $^2J_{Sn\text{-}Sn}$ = 5466 Hz, CH$_3$*Sn*I). **Anal.** Calcd. for $C_{61}H_{77}Sn_2FeI$: C 59.54, H 6.31; Found: C 59.56, H 6.61. **Absorption spectrum** (toluene, 298 K): λ_{max}, nm (ε, M^{-1} cm^{-1}) 370 (10700), 475 (6190), 570 (shoulder, 1260), 660 (broad, 720).

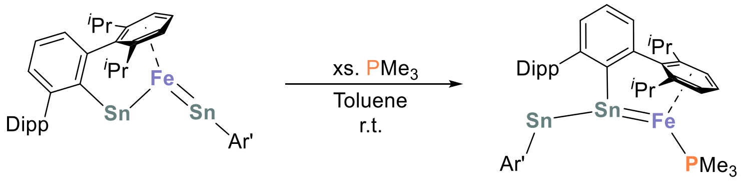

1 5

Synthesis of [(Ar'SnSnAr')Fe(PMe$_3$)] (5). To a dark brown toluene (10 ml) solution of **1** (55 mg, 0.05 mmol) was added PMe$_3$ (19.0 mg, 0.25 mmol) at room temperature. The color of the solution slowly turned dark red. The reaction mixture was stirred at room temperature overnight. After removing the solvent under vacuo, dark red powder of **5** was obtained in a quantitative yield. Dark red crystals suitable for X-ray crystallography were obtained by slow evaporation of the red solution of **5** in *n*-pentane at room temperature. ^{1}**H NMR** (400 MHz, C_6D_6, 298 K): δ (ppm) 0.62 (d, J = 7.2 Hz, 6H, −CH(CH_3)$_2$ of Dipp-Ar'SnFe), 0.65 (d, J = 6.8 Hz, 6H, −CH(CH_3)$_2$ of η^6-Dipp-Fe), 1.06 (d, J = 7.2 Hz, 12H, −CH(CH_3)$_2$ of Dipp-Ar'Sn), 1.08 (d, J = 6.8 Hz, 6H, −CH(CH_3)$_2$ of η^6-Dipp-Fe), 1.18 (d, J = 6.8 Hz, 6H, −CH(CH_3)$_2$ of Dipp-Ar'SnFe), 1.51 (d, $J^{\mathrm{P\text{-}H}}$ = 6.8 Hz, 9H, (*Me*$_3$P), 3.07 (broad, 4H, −C*H*(CH$_3$)$_2$ of Dipp-Ar'Sn), 3.25 (sept, J = 6.8 Hz, 2H, −C*H*(CH$_3$)$_2$ of η^6-Dipp-Fe), 3.29 (sept, J = 6.8 Hz, 2H, −C*H*(CH$_3$)$_2$ of Dipp-Ar'SnFe), 4.03 (dd, $J^{\mathrm{H\text{-}H}}$ = 6.0 Hz, $J^{P\text{-}H}$ = 2.8 Hz, 2H, *m*-C*H*-η^6-Dipp-Fe), 6.04 (dd, $J^{\mathrm{H\text{-}H}}$ = 6.0 Hz, $J^{P\text{-}H}$ = 12.0 Hz, 1H, *p*-C*H*-η^6-Dipp-Fe), 7.08–7.15 (m, 10H, *m* and *p*-C*H*-Ar'Sn/*m* and *p*-C*H*-Dipp-Ar'SnFe/*m*-C*H*-DippAr'Sn), 7.21 (t, 2H, J = 7.6 Hz, *p*-C*H*-Dipp-Ar'Sn), 7.41 (dd, J = 7.2, 1.2 Hz, 1H, *m*-C*H*-Ar'SnFe), 7.79 (t, 1H, J = 7.2 Hz, *p*-C*H*-Ar'SnFe), 7.90 (dd, J = 7.6, 1.6 Hz, 1H, *m*-C*H*-Ar'SnFe). Signals of 12 protons corresponding to the −CH(CH_3)$_2$ group of Dipp-Ar'Sn moiety were not observed due to the dynamic behavior of **5** in solution. This dynamic process could be attributed to the rotation of the Ar' substituent around the Sn−C$_{ipso}$ axis. These signals can be detected in the ^{1}H NMR spectrum recorded at 233 K (Figures S16 and S17). ^{13}**C{^{1}H} NMR** (100 MHz, C_6D_6, 298 K): δ (ppm) 192.9 (Ar'-*C*-Sn), 185.6 (Ar'-*C*-SnFe), 149.1 (*o*-*C*-Ar'SnFe), 128.3 (*m*-*C*H-Ar'), 146.7 (*o*-*C*-Dipp-Ar'Sn), 146.5 (*o*-*C*-Dipp-Ar'SnFe), 145.8 (Dipp-*C*-Ar'SnFe), 143.9 (*o*-*C*-Ar'SnFe), 143.2 (*o*-*C*-Ar'Sn), 137.0 (Dipp-*C*-Ar'Sn), 131.0 (*m*-*C*H-Ar'Sn), 130.4 (*m*-*C*H-Ar'SnFe), 129.2 (*m*-*C*H-Ar'SnFe), 128.8 (*p*-*C*H-Ar'Sn), 128.6 (*p*-*C*H-Dipp-Ar'Sn), 125.3 (*p*-*C*H-Dipp-Ar'SnFe), 124.3 (*p*-*C*H-Ar'SnFe), 123.6 (*m*-*C*H-Dipp-Ar'Sn), 123.5 (*m*-*C*H-Dipp-Ar'SnFe), 111.1 (η^6-Dipp-*C*-Ar'), 111.0 (*o*-*C*-η^6-Dipp-Fe), 90.1 (*p*-*C*H-η^6-Dipp-Fe), 72.2 (*m*-*C*H-η^6-Dipp-Fe), 31.9 (−*C*H(CH$_3$)$_2$ of η^6-Dipp-Fe), 30.4 (−*C*H(CH$_3$)$_2$ of Dipp-Ar'SnFe), 30.3 (−*C*H(CH$_3$)$_2$ of Dipp-Ar'Sn), 26.7 (−CH(*C*H$_3$)$_2$ of Dipp-Ar'Sn), 26.4 (−CH(*C*H$_3$)$_2$ of η^6-Dipp-Fe), 25.1 (d, $J^{\mathrm{P\text{-}C}}$ = 24.1 Hz, *Me*$_3$P), 24.7 (−CH(*C*H$_3$)$_2$ of Dipp-Ar'SnFe), 24.5 (−CH(*C*H$_3$)$_2$ of η^6-Dipp-Fe), 23.4 (−CH(*C*H$_3$)$_2$ of Dipp-Ar'Sn), 21.3 (−CH(*C*H$_3$)$_2$ of Dipp-Ar'SnFe). ^{119}**Sn{^{1}H} NMR** (224 MHz, C_6D_6, 298 K): δ (ppm) 2403 (s, Ar'*Sn*Sn), 1456 (s, Ar'*Sn*Fe). ^{31}**P{^{1}H} NMR** (162 MHz, C_6D_6, 298 K): δ (ppm) −44.7. **Anal.** Calcd. for $C_{63}H_{83}Sn_2FeP{\cdot}2C_5H_{12}$: C 66.99, H 8.24; Found: C 66.66, H 8.01. **Absorption spectrum** (toluene, 298 K): λ_{max}, nm (ε, M^{-1} cm^{-1}) 310 (13400), 360 (shoulder, 5440), 450 (3960), 685 (1370). ^{57}**Fe Mössbauer** (solid state, 80 K): δ = 0.50 mm s^{-1}, |ΔE$_Q$| = 1.17 mm s^{-1}.

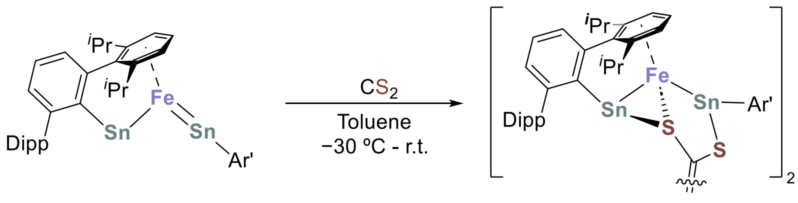

1 6

Synthesis of [((Ar'Sn)$_2$Fe)$_2$C$_2$S$_4$] (6). To a dark brown toluene (10 ml) solution of **1** (55 mg, 0.05 mmol) at −30 °C was added a pre-cooled CS$_2$ solution in THF (10 µL, 0.05 mmol, 5 M). The reaction mixture was allowed to warm to room temperature and during this time, the color of the solution turned dark red. After stirring at room temperature for 2 h, the solvent was removed under vacuo, and the resulting red solid was dissolved in *n*-pentane (*ca.* 2 mL). Slow evaporation of this *n*-pentane solution yielded dark red crystals of **6**. Yield: 34 mg, 58%. ^{1}**H NMR** (400 MHz, C_6D_6, 298 K): δ (ppm) 0.77 (d, J = 6.8 Hz, 3H, −CH(CH_3)$_2$ of Dipp-Ar'(η^6-Dipp)Sn), 1.01–1.17 (*m*, 18H, −CH(CH_3)$_2$), 1.20 (d, J = 6.8 Hz, 3H, −CH(CH_3)$_2$ of Dipp-Ar'(η^6-Dipp)), 1.29 (broad, 6H, −CH(CH_3)$_2$ of Dipp-Ar'Sn), 1.37 (d, J = 6.4 Hz, 6H, −CH(CH_3)$_2$ of Dipp-Ar'Sn), 1.42 (d, J = 7.2 Hz, 3H, −CH(CH_3)$_2$ of Dipp-Ar'(η^6-Dipp)), 1.46 (d, J = 7.2 Hz, 3H, −CH(CH_3)$_2$ of η^6-Dipp-Fe), 1.51 (d, J = 6.8 Hz, 3H, −CH(CH_3)$_2$ of η^6-Dipp-Fe), 1.79

(d, J = 6.8 Hz, 3H, –CH(CH_3)$_2$ of η^6-Dipp-Fe), 2.35–2.56 (m, 2H, –CH(CH$_3$)$_2$ of η^6-Dipp-Fe and Dipp-Ar'(η^6-Dipp)), 2.70 (sept, J = 6.8 Hz, 1H, –CH(CH$_3$)$_2$ of Dipp-Ar'(η^6-Dipp)), 2.80–3.14 (m, broad, 4H, –CH(CH$_3$)$_2$ of Dipp-Ar'Sn), 3.24 (sept, J = 6.8 Hz, 1H, –CH(CH$_3$)$_2$ of η^6-Dipp-Fe), 4.32 (d, J = 5.6 Hz, 1H, *m*-C*H*-η^6-Dipp-Fe), 5.76 (t, J = 5.6 Hz, 1H, *p*-C*H*-η^6-Dipp-Fe), 6.20 (d, J = 5.6 Hz, 1H, *m*-C*H*-η^6-Dipp-Fe), 7.06 – 7.13 (m, 2H, *p*-C*H*-Ar'(η^6-Dipp) and *p*-C*H*-Ar'Sn), 7.22 (d, J = 8.0 Hz, 2H, *m*-C*H*-Ar'Sn), 7.24 (dd, J = 7.6, 0.8 Hz, 1H, *m*-C*H*-Ar'(η^6-Dipp)), 7.32 (dd, J = 7.6, 0.8 Hz, 1H, *m*-C*H*-Ar'(η^6-Dipp)), 7.39 (dd, J = 8.0, 1.2 Hz, 1H, *m*-C*H*-Dipp-Ar'(η^6-Dipp)), 7.51 (t, J = 7.6 Hz, 1H, *p*-C*H*-Dipp-Ar'(η^6-Dipp)), 7.62 (dd, J = 7.6, 1.2 Hz, 1H, *m*-C*H*-Dipp-Ar'(η^6-Dipp)). The signals of the –CH(CH_3)$_2$ (6H) group of the Dipp-Ar'Sn unit are broad and cannot be clearly discerned because of the dynamic behavior of **6** in solution, but they have been detected in the ^{1}H NMR spectrum recorded at 233 K (Figures S22 and S23). **^{13}C{^{1}H} NMR** (100 MHz, C_6D_6, 298 K): δ (ppm) 171.9, 163.9, 149.2, 147.6, 147.5, 147.4, 146.1, 145.4, 141.7, 141.5, 140.2, 138.5, 130.7, 129.6, 129.1, 128.6, 128.0, 127.3, 125.8, 124.0, 123.0, 122.9, 111.5, 111.0, 100.6, 79.5, 78.8, 65.5, 31.05, 31.03, 31.0, 30.9, 29.8, 29.7, 27.7, 26.6, 26.4, 26.2, 25.6, 25.5, 24.7, 24.1, 23.8, 22.3. Signals at *ca.* 24.0 and 26.3 corresponding to the –CH(CH$_3$)$_2$ of Dipp-Ar'Sn are very broad because of the dynamic behavior of **6** in solution (Figure S23) **^{119}Sn{^{1}H} NMR** (224 MHz, C_6D_6, 298 K): δ (ppm) 655 (s), –124 (s). **Anal.** Calcd. for $C_{122}H_{148}Sn_4Fe_2S_4 \cdot C_5H_{12}$: C 64.09, H 7.01; Found: C 64.01, H 7.08. **Absorption spectrum** (toluene, 298 K): λ_{max}, nm (ε, M^{-1} cm^{-1}) 360 (shoulder, 23680), 430 (16570), 505 (broad, 9900), 530 (broad, 9700), 700 (shoulder, 2140).

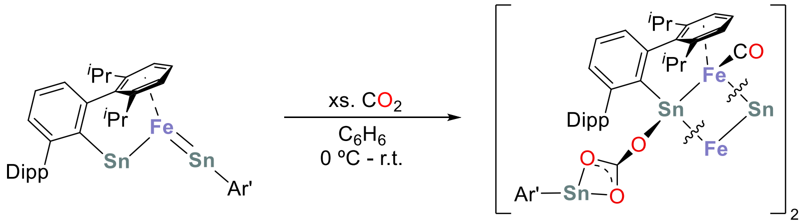

1 7

Synthesis of [(Ar'SnCO$_3$SnAr')Fe(CO)]$_2$ (7). To a dark brown benzene (10 ml) solution of **1** (55 mg, 0.05 mmol) in a Schlenk tube (with high vacuum valve plug) at 0 °C (ice bath) was injected CO_2 gas (4 bar). The reaction was kept at 0 °C for 30 min and then was slowly warmed to room temperature. During this time, the color of the solution turned dark red slowly. The reaction mixture was further stirred at room temperature overnight. After removing the solvent under vacuo, the resulting red solid was washed by *n*-hexane (3 × 2 mL) and then extracted with Et_2O (2 × 2 mL). Slow evaporation of the combined Et_2O solution at room temperature yielded dark red crystals of **7**. Yield: 18 mg, 30%. **^{1}H NMR** (400 MHz, C_6D_6, 298 K): δ (ppm) 0.56 (d, J = 7.2 Hz, 3H), 0.58 (d, J = 6.8 Hz, 3H), 0.97 (d, J = 6.4 Hz, 3H), 1.03 (d, J = 6.8 Hz, 3H), 1.12 (d, J = 6.8 Hz, 6H), 1.13 – 1.19 (m, 15H), 1.20 (d, J = 6.6 Hz, 6H), 1.40 (d, J = 6.6 Hz, 3H), 1.43 (d, J = 6.8 Hz, 3H), 2.46 (sept, J = 6.8 Hz, 1H), 2.90 (sept, J = 6.8 Hz, 1H), 3.05 (sept, J = 6.8 Hz, 1H), 3.12 – 3.23 (m, 5H), 4.64 (d, J = 6.4 Hz, 1H, *m*-C*H*-η^6-Dipp-Fe), 4.73 (t, J = 6.4 Hz, 1H, *p*-C*H*-η^6-Dipp-Fe), 5.47 (d, J = 6.4 Hz, 1H, *m*-C*H*-η^6-Dipp-Fe), 7.09 – 7.14 (m, 1H), 7.18 – 7.31 (m, 10H), 7.33 – 7.40 (m, 3H), 7.59 (dd, J = 7.6, 0.8 Hz, 1H). **^{13}C{^{1}H} NMR** (125 MHz, C_6D_6, 298 K): δ (ppm) 176.9, 164.2, 161.6, 149.0, 148.2, 147.6, 147.5, 146.0, 145.6, 142.3, 140.6, 138.8, 132.1, 130.3, 129.5, 128.5, 126.8, 126.7, 123.7, 123.4, 123.2, 122.0, 121.6, 119.5, 110.3, 90.4, 86.0, 84.0, 32.1, 31.1, 30.9, 30.7, 30.7, 29.3, 27.6, 27.5, 27.3, 26.7, 26.6, 26.0, 23.9, 23.6, 23.5, 23.2, 23.1, 22.1. **^{119}Sn{^{1}H} NMR** (224 MHz, C_6D_6, 298 K): δ (ppm) 343 (s), 264 (s). **Anal.** Calcd. for $C_{124}H_{148}Sn_4Fe_2O_4 \cdot 2Et_2O$: C 63.46, H 7.15; Found: C 63.83, H 6.74. **Absorption spectrum** (toluene, 298 K): λ_{max}, nm (ε, M^{-1} cm^{-1}) 320 (shoulder, 16400), 380 (shoulder, 7350), 470 (2500), 665 (broad, 155). **ATR–IR** (powder, cm^{-1}): ν = 1909 (Fe*CO*).

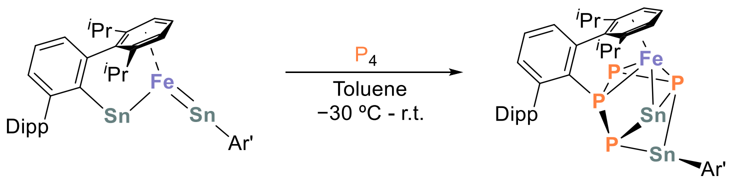

Synthesis of [(Ar'P_4Sn_2Ar')Fe] (8). To the dark brown toluene (10 ml) solution of **1** (80 mg, 0.07 mmol) at −30 °C was added solid P_4 (9.8 mg, 0.08 mmol). The reaction was allowed to warm to room temperature and was stirred overnight. During this time, the color of the solution turned dark red slowly. After removing the solvent under vacuo, dark red powder of **8** was obtained in a quantitative yield. Dark red crystals suitable for X-ray crystallography were obtained by slow evaporation of the red solution of **8** in *n*-pentane at room temperature. **^{1}H NMR** (400 MHz, C_6D_6, 298 K): δ (ppm) 0.75 (d, J = 6.8 Hz, 3H), 0.81 (d, J = 6.8 Hz, 3H), 0.86 (d, J = 6.4 Hz, 3H), 0.93 (d, J = 6.8 Hz, 3H), 1.10 (d, J = 6.8 Hz, 6H), 1.15 (d, J = 6.8 Hz, 6H), 1.19 (d, J = 6.8 Hz, 3H), 1.22 (d, J = 6.8 Hz, 3H), 1.23 (d, J = 6.8 Hz, 3H), 1.61 (d, J = 6.6 Hz, 3H), 1.63 (d, J = 6.8 Hz, 6H), 1.65 (d, J = 6.8 Hz, 6H), 2.03 (sept, J = 6.8 Hz, 1H), 2.15 (sept, J = 6.8 Hz, 1H), 2.47 (sept, J = 6.8 Hz, 1H), 2.81 (sept, J = 6.8 Hz, 1H), 2.99 (sept, J = 6.8 Hz, 2H), 3.20 (sept, J = 6.8 Hz, 2H), 4.80 (d, J = 6.0 Hz, 1H, *m*-C*H*-η^6-Dipp-Fe), 4.98 (t, J = 6.0 Hz, 1H, *p*-C*H*-η^6-Dipp-Fe), 5.10 (d, J = 6.0 Hz, 1H, *m*-C*H*-η^6-Dipp-Fe), 6.99 – 7.12 (m, 3H), 7.12 – 7.15 (m, 3H), 7.24 – 7.32 (m, 4H), 7.32 – 7.39 (m, 3H), 7.42 (t, J = 7.6 Hz, 2H). **^{13}C{^{1}H} NMR** (100 MHz, C_6D_6, 298 K): δ (ppm) 160.0, 152.8, 149.0, 147.0, 146.8, 146.7, 146.1, 145.9 (d, $J_{P\text{-}C}$ = 5.9 Hz), 144.6 (d, $J_{P\text{-}C}$ = 13.4 Hz), 142.4, 136.7 (d, $J_{P\text{-}C}$ = 2.9 Hz), 130.3 (d, $J_{P\text{-}C}$ = 6.5 Hz), 130.0, 129.3, 129.1, 128.4, 128.2 (d, $J_{P\text{-}C}$ = 1.1 Hz), 125.1, 124.4, 124.3, 124.0, 122.6, 122.5, 110.3, 95.0 (d, $J_{P\text{-}C}$ = 2.5 Hz), 85.2, 84.0, 82.0 (d, $J_{P\text{-}C}$ = 1.7 Hz), 31.6, 31.1 (2C), 31.0 (d, $J_{P\text{-}C}$ = 1.2 Hz), 30.6 (d, $J_{P\text{-}C}$ = 6.0 Hz), 29.1, 27.6, 27.1, 27.06, 26.6, 26.4, 26.3, 24.1, 24.06 (d, $J_{P\text{-}C}$ = 7.8 Hz), 23.8 (d, $J_{P\text{-}C}$ = 2.9 Hz), 22.9 (d, $J_{P\text{-}C}$ = 4.5 Hz), 21.3, 21.0 (d, $J_{P\text{-}C}$ = 14.3 Hz). **^{31}P{^{1}H} NMR** (162 MHz, C_6D_6, 298 K): δ (ppm) 38.5 (ddd, $J_{P\text{-}P}$ = 494, 459, 35 Hz), −17.0 (ddd, $J_{P\text{-}P}$ = 364, 35, 27 Hz), −162.9 (ddd, $J_{P\text{-}P}$ = 486, 28, 4.6 Hz), −196.7 (ddd, $J_{P\text{-}P}$ = 459, 364, 3.4 Hz). $^{117/119}$Sn satellites were detected for these multiplets (see Figures S31 and S33 and Table S1 for the assignment and simulation). **^{119}Sn{^{1}H} NMR** (224 MHz, C_6D_6, 298 K): δ (ppm) −366 (ddd, $J_{Sn\text{-}P}$ = 492.2, 456.8, 143.6 Hz), −455 (dtd, $J_{Sn\text{-}P}$ = 753.1, 321.1, 321.1, 107.3 Hz) (see Figure S34 and Table S2 for the simulation). **Anal.** Calcd. for $C_{60}H_{74}Sn_2FeP_4{\cdot}C_5H_{12}$: C 60.78, H 6.75; Found: C 60.45, H 6.57. **Absorption spectrum** (toluene, 298 K): λ_{max}, nm (ε, M^{-1} cm^{-1}) 320 (shoulder, 17800), 450 (shoulder, 3250).

NMR Spectra

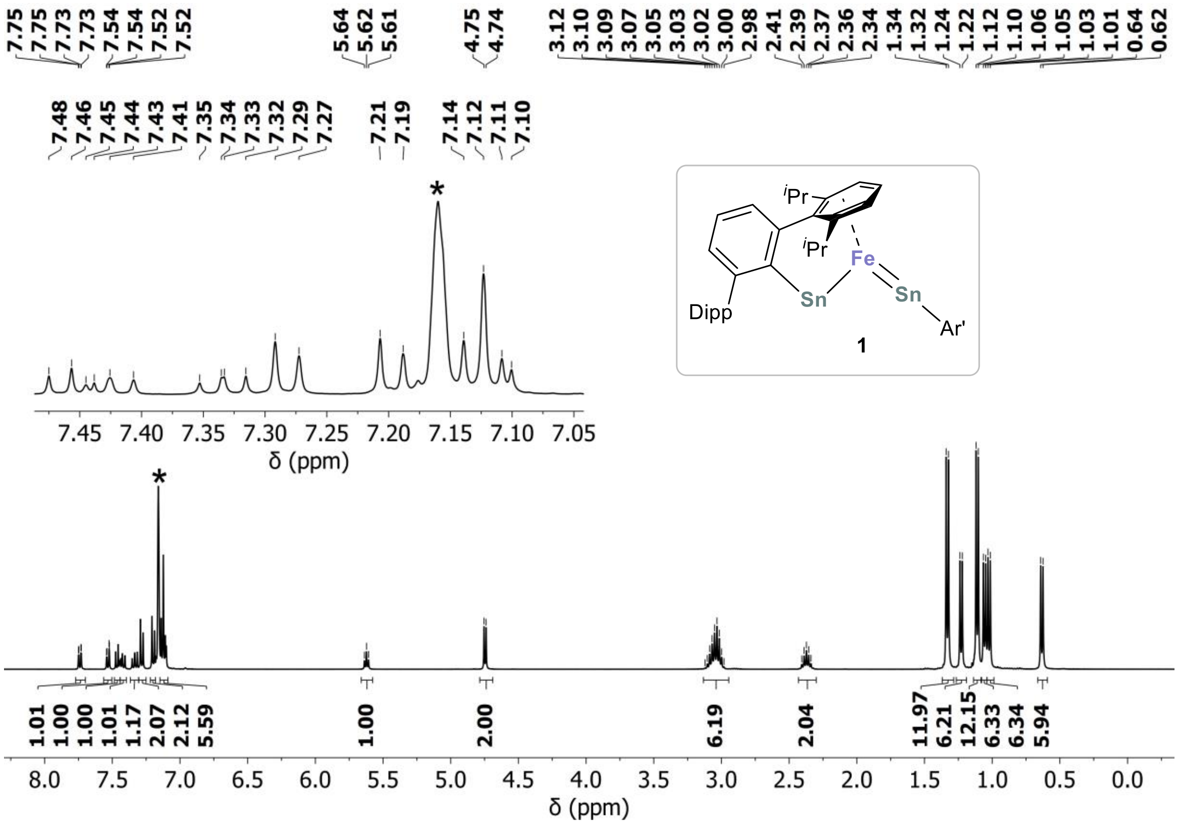

Figure S1. ^{1}H NMR (400 MHz, 298 K) spectrum of $[Fe(SnAr')_2]$ (**1**) in C_6D_6. *C_6D_6.

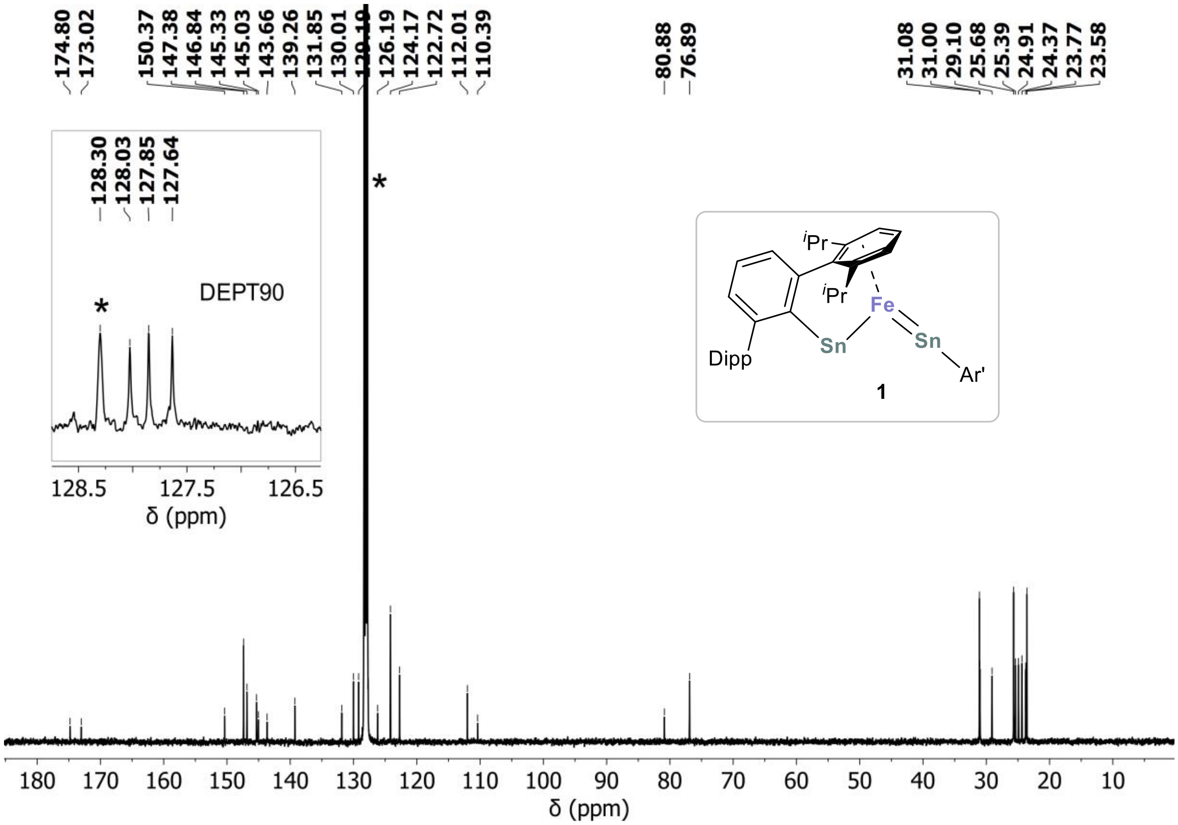

Figure S2. $^{13}C\{^{1}H\}$ NMR (100 MHz, 298 K) spectrum of $[Fe(SnAr')_2]$ (**1**) in C_6D_6. Inset: DEPT90 $^{13}C\{^{1}H\}$ NMR spectrum of **1** in C_6D_6, showing the signals that overlap with the residual signal of C_6D_6 in the standard $^{13}C\{^{1}H\}$ NMR spectrum. *C_6D_6.

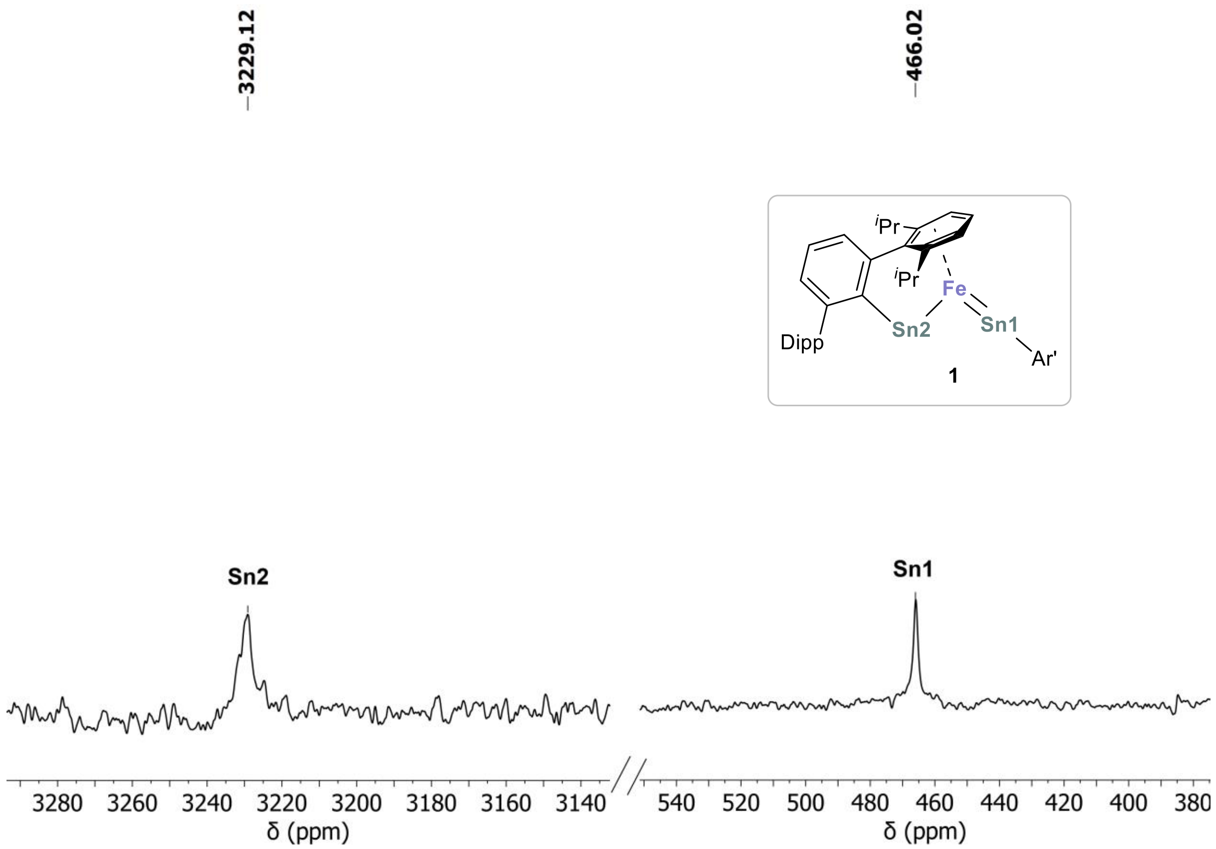

Figure S3. $^{119}Sn\{^{1}H\}$ NMR (224 MHz, 298 K) spectrum of $[Fe(SnAr')_2]$ (**1**) in d_8-THF.

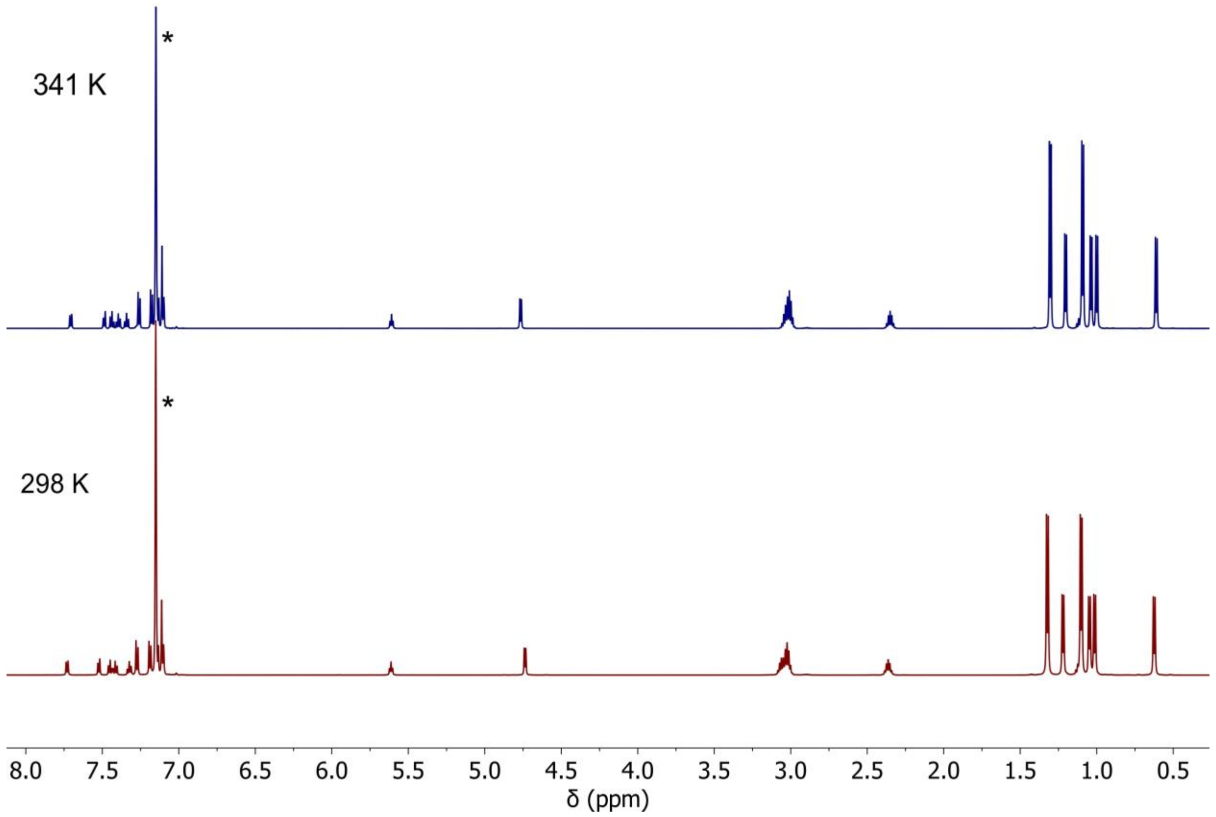

Figure S4. Comparison of ^{1}H NMR (600 MHz) spectra of $[Fe(SnAr')_2]$ (**1**) recorded at 298 K and 341 K in C_6D_6. $^{*}C_6D_6$.

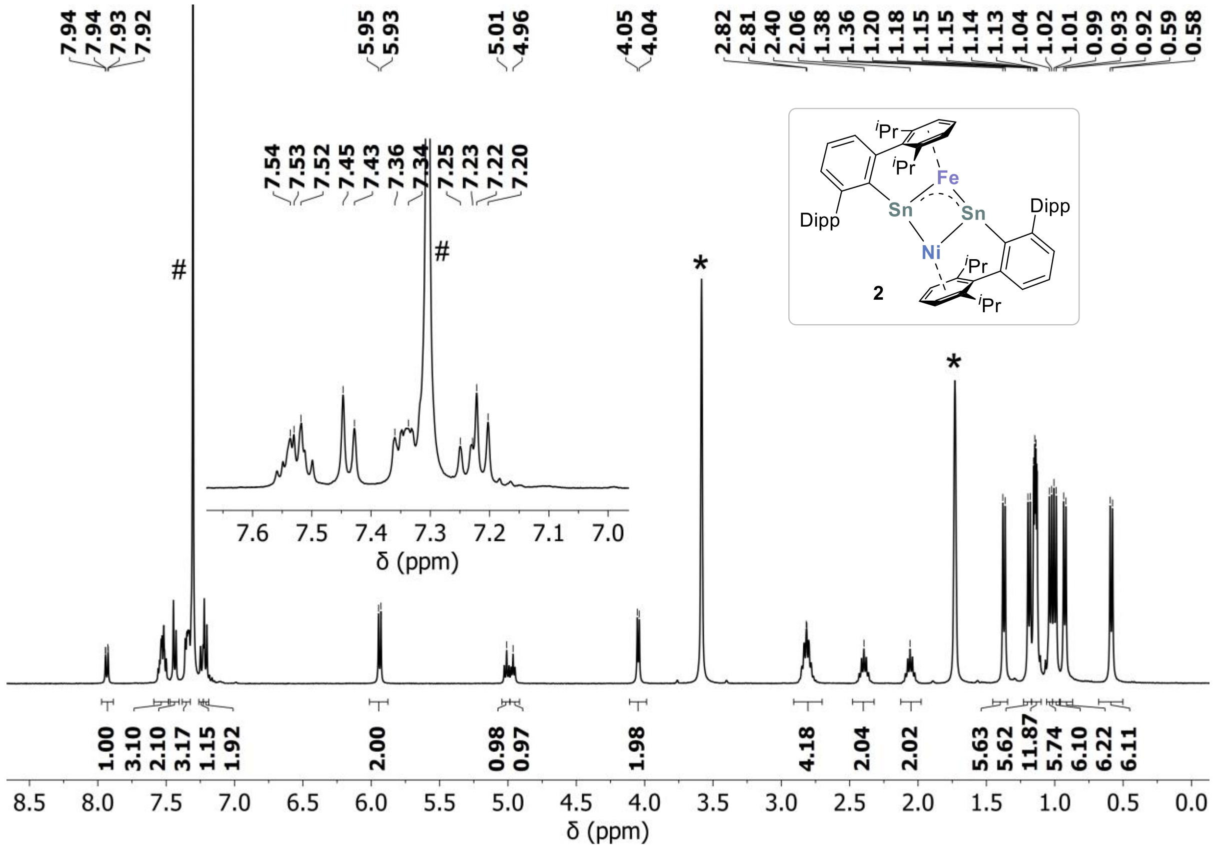

Figure S5. ^{1}H NMR (400 MHz, 298 K) spectrum of [(Ar'Sn)$_2$FeNi] (**2**) in d_8-THF. *d_8-THF, #C_6H_6.

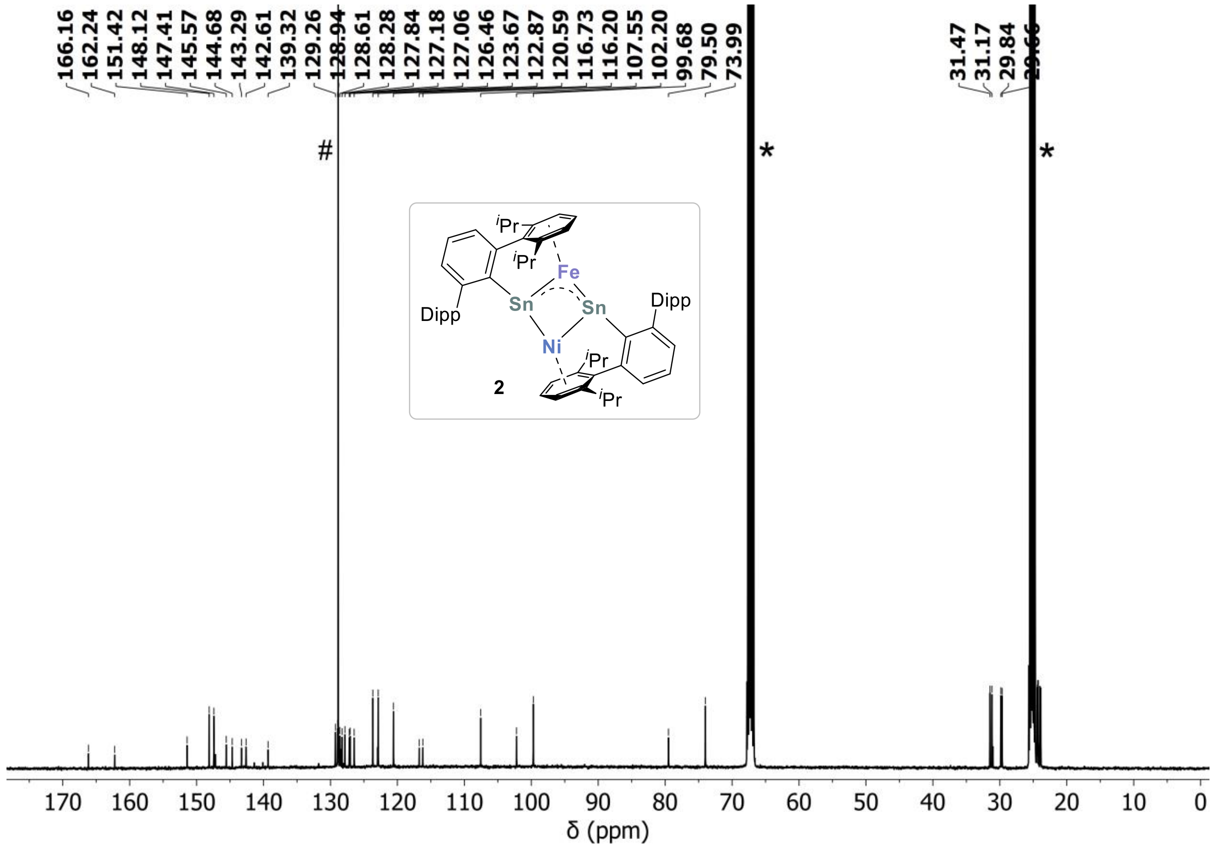

Figure S6. ^{13}C{^{1}H} NMR (100 MHz, 298 K) spectrum of [(Ar'Sn)$_2$FeNi] (**2**) in d_8-THF. *d_8-THF, #C_6H_6.

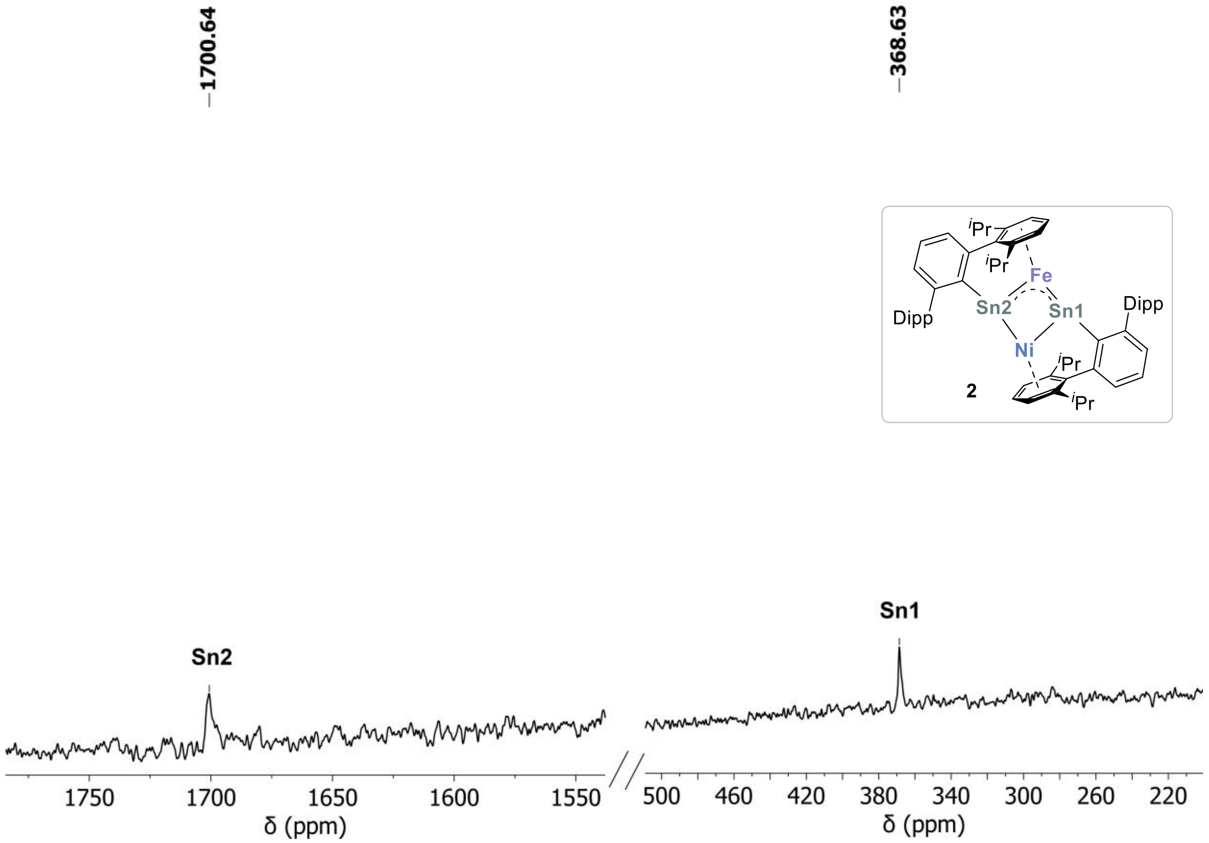

Figure S7. $^{119}Sn\{^1H\}$ NMR (224 MHz, 298 K) spectrum of [(Ar’Sn)$_2$FeNi] (**2**) in d_8-THF.

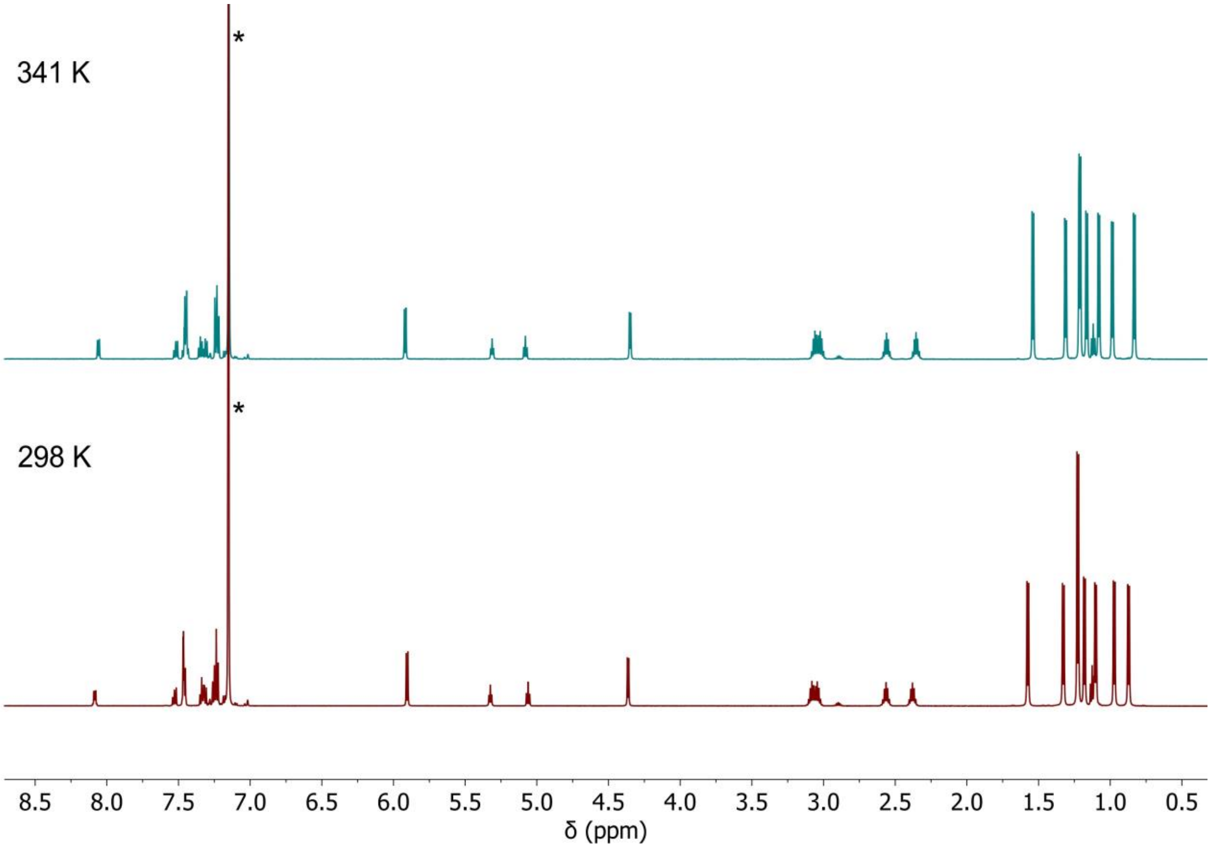

Figure S8. Comparison of ^1H NMR (600 MHz) spectra of [(Ar’Sn)$_2$FeNi] (**2**) recorded at 298 K and 341 K in C_6D_6. *C_6D_6.

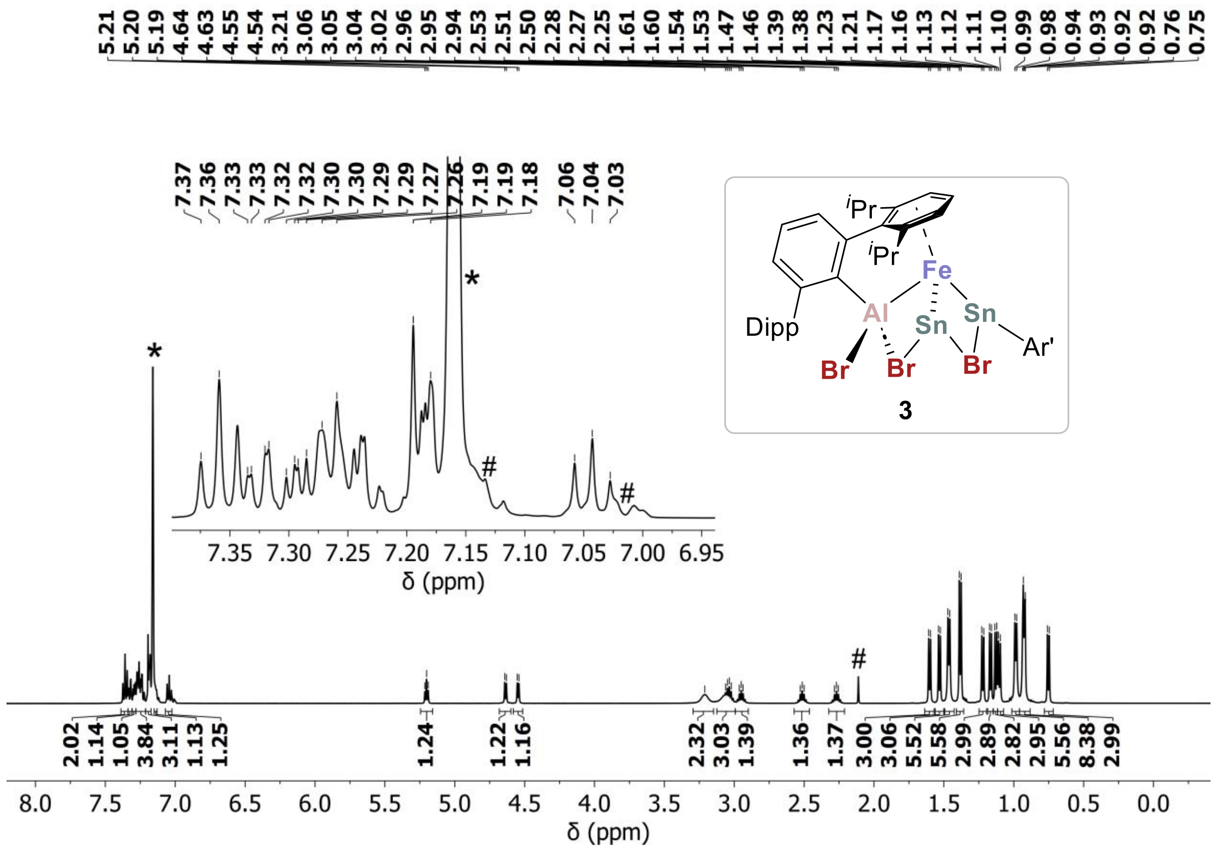

Figure S9. ^{1}H NMR (500 MHz, 298 K) spectrum of [(Ar'AlBr$_2$)(Ar'SnBr)FeSn] **(3)** in C_6D_6. *C_6D_6, #Toluene.

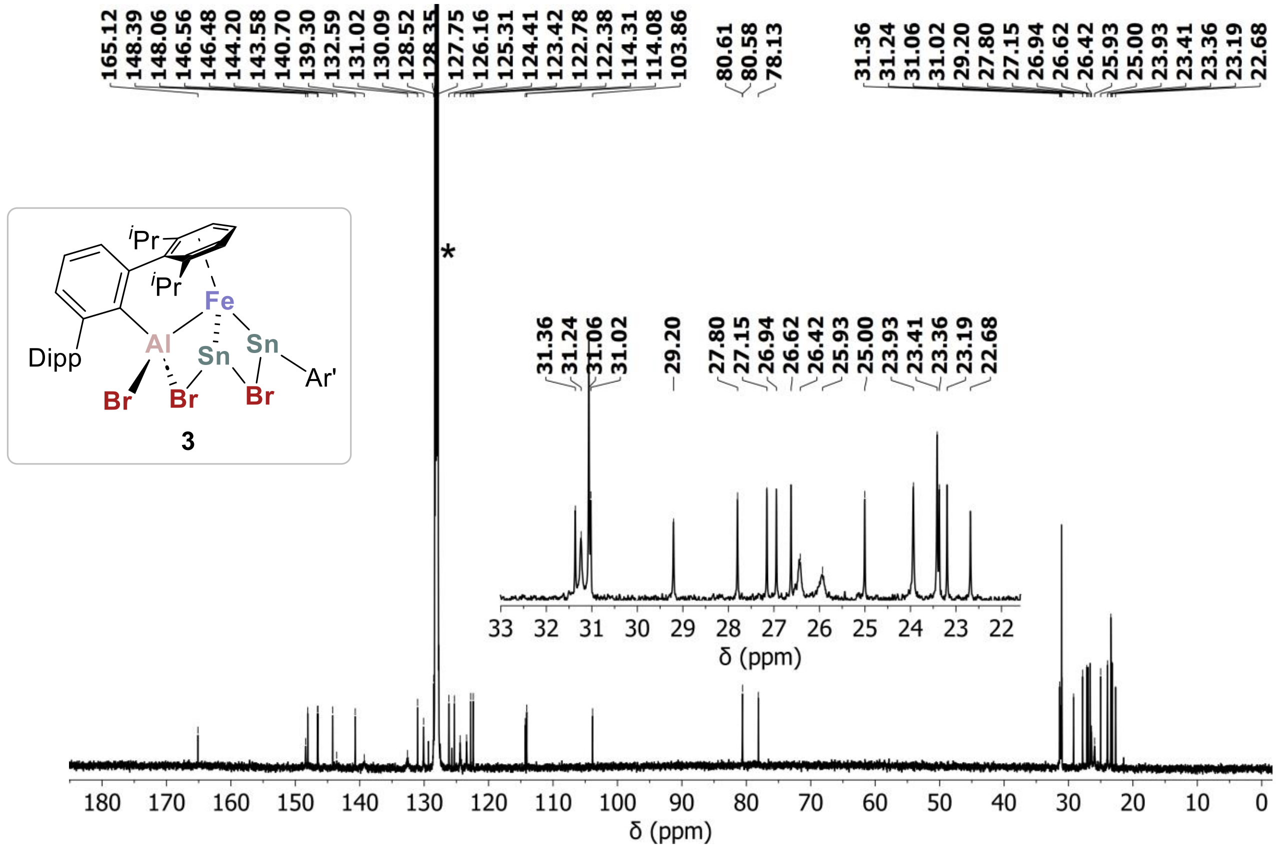

Figure S10. $^{13}C\{^{1}H\}$ NMR (125 MHz, 298 K) spectrum of [(Ar'AlBr$_2$)(Ar'SnBr)FeSn] (**3**) in C_6D_6. *C_6D_6.

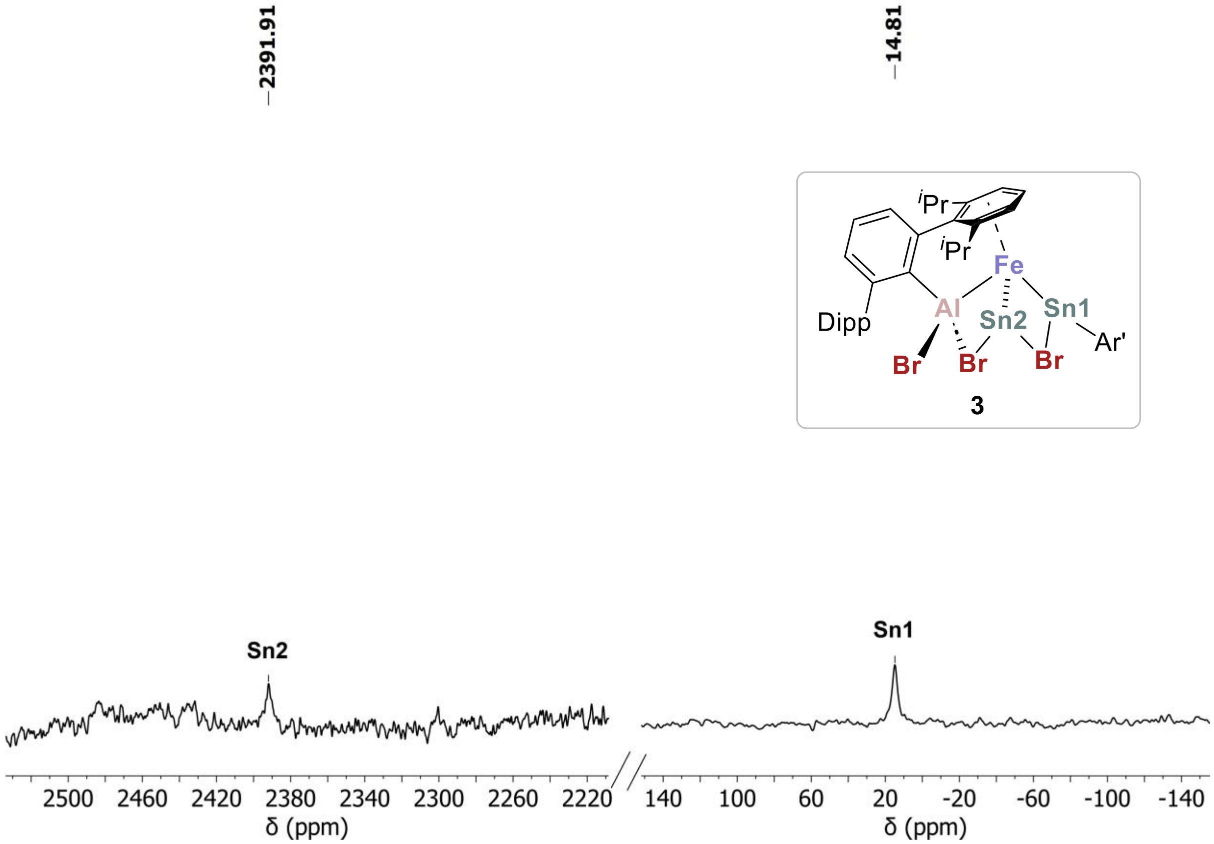

Figure S11. $^{119}Sn\{^{1}H\}$ NMR (224 MHz, 298 K) spectrum of [(Ar’AlBr₂)(Ar’SnBr)FeSn] (**3**) in *d*₈-THF.

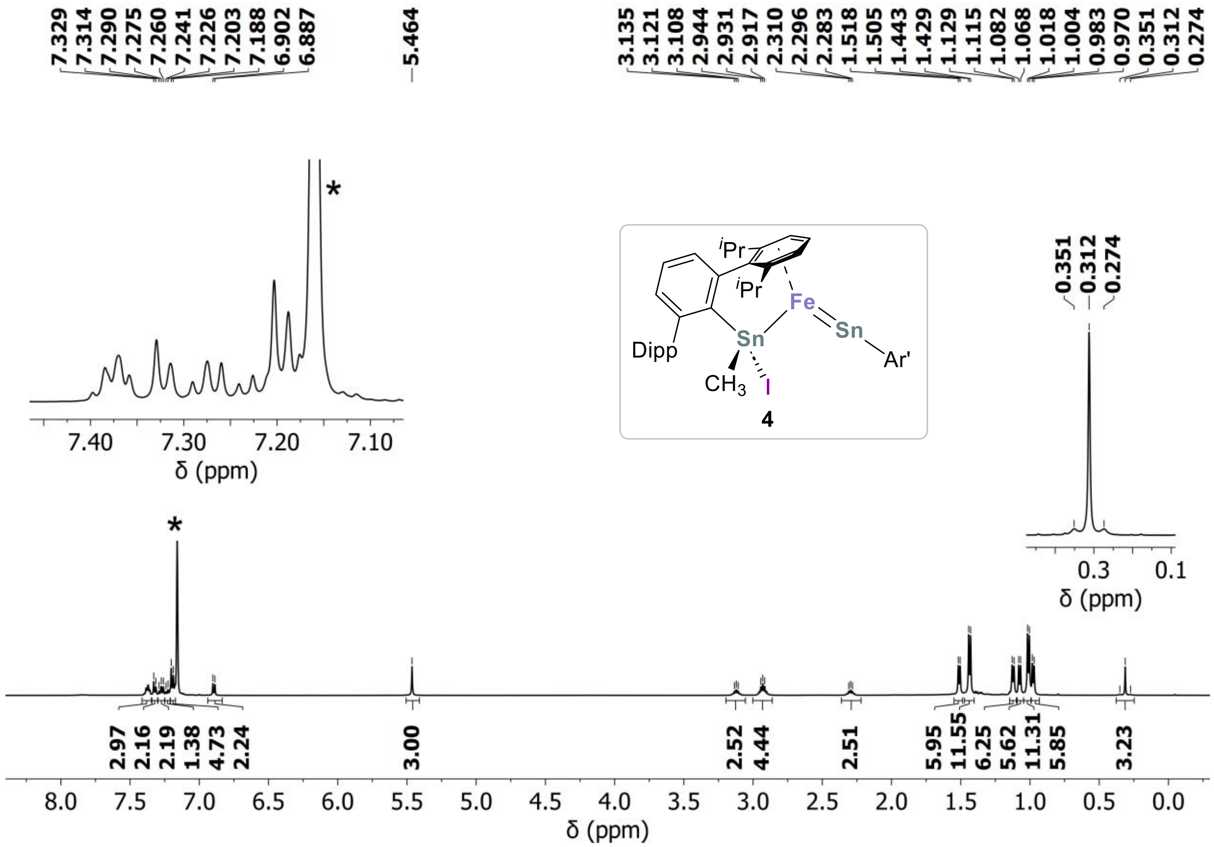

Figure S12. ^{1}H NMR (500 MHz, 298 K) spectrum of [(Ar’(CH₃)SnI)Fe(SnAr’)] (**4**) in C_6D_6. *C_6D_6.

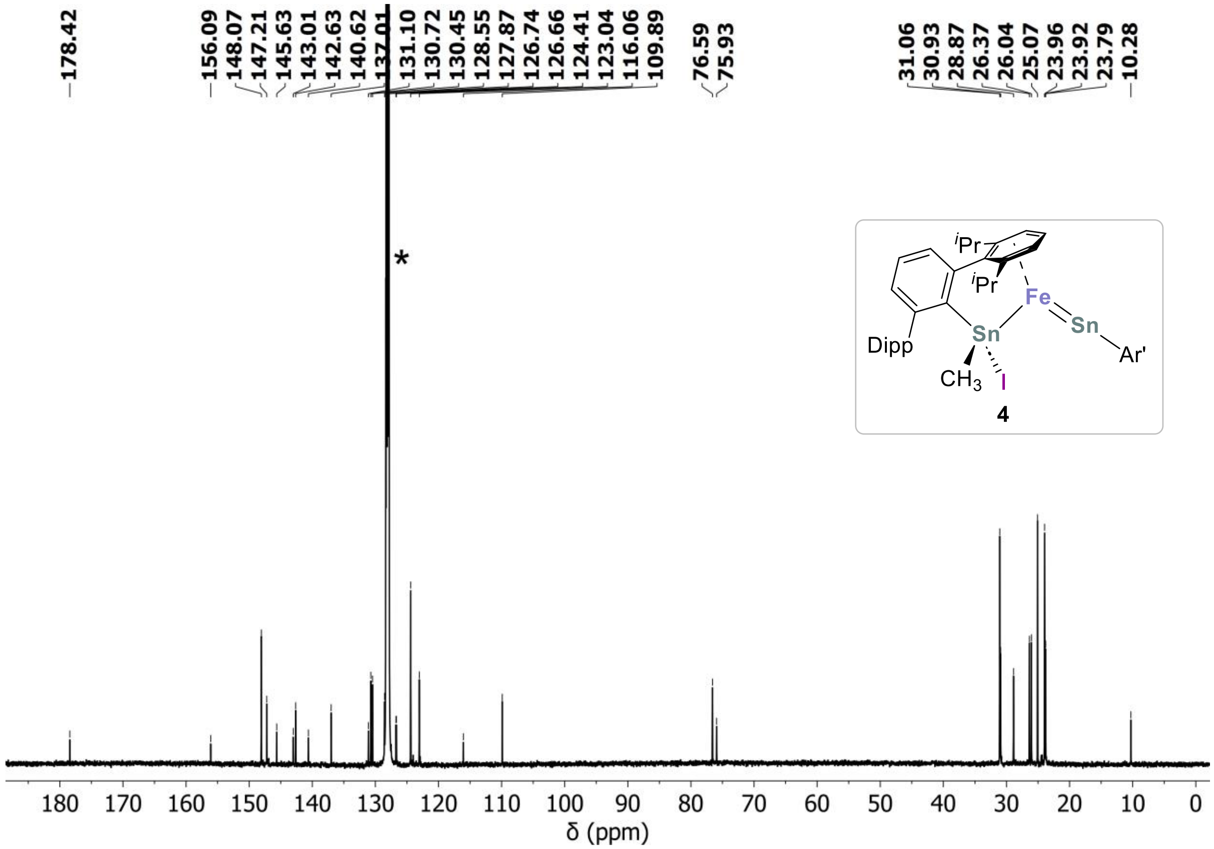

Figure S13. $^{13}C\{^{1}H\}$ NMR (125 MHz, 298 K) spectrum of [(Ar'(CH$_3$)SnI)Fe(SnAr')] (**4**) in C_6D_6. *C_6D_6.

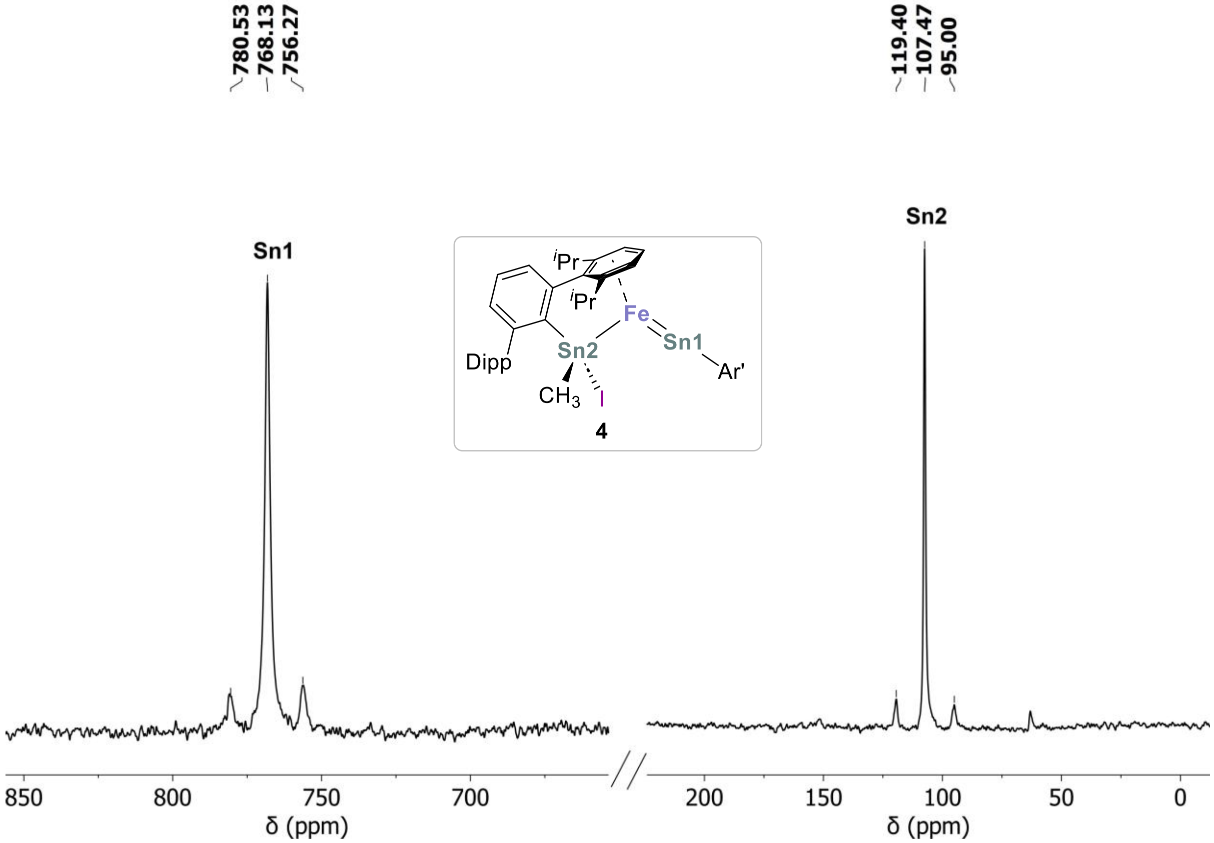

Figure S14. $^{119}Sn\{^{1}H\}$ NMR (224 MHz, 298 K) spectrum of [(Ar'(CH$_3$)SnI)Fe(SnAr')] (**4**) in C_6D_6.

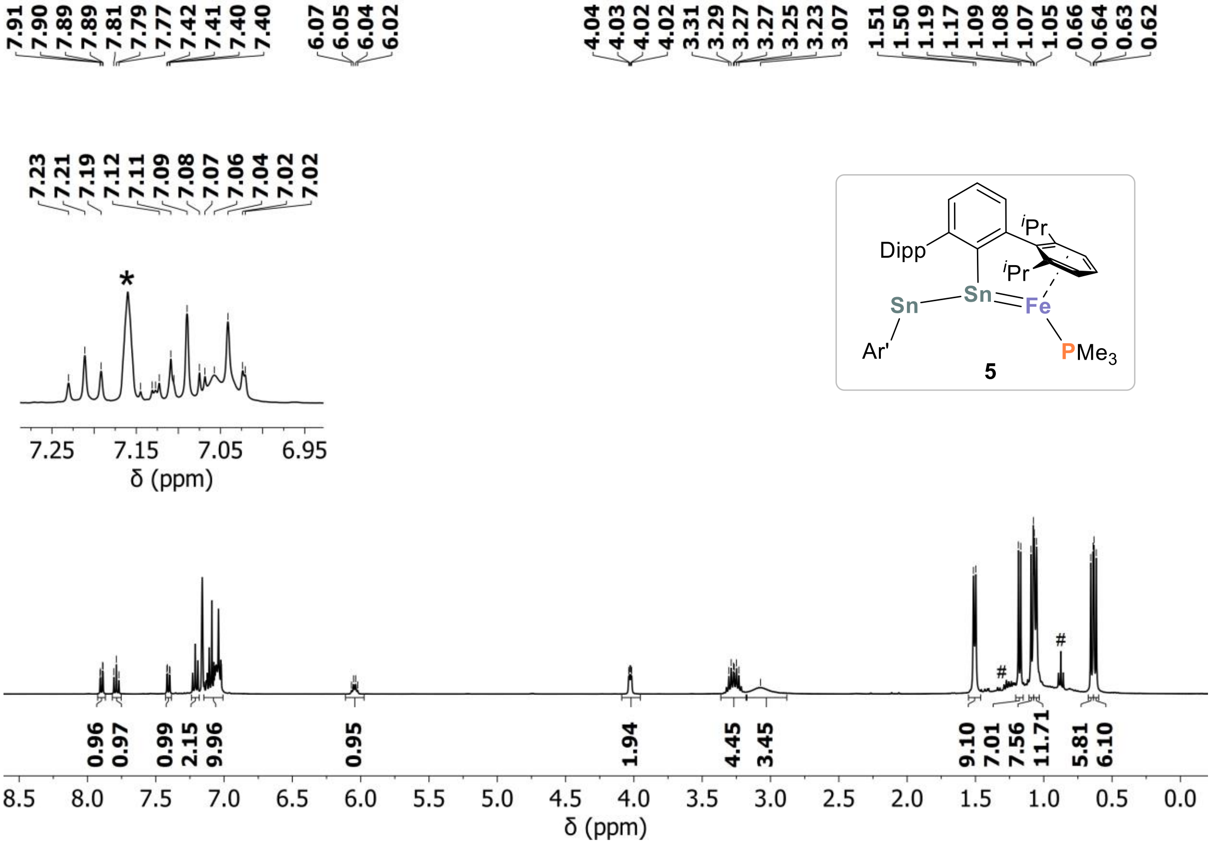

Figure S15. ^{1}H NMR (400 MHz, 298 K) spectrum of [(Ar'SnSnAr')Fe(PMe$_3$)] (**5**) in C_6D_6. *C_6D_6, #*n*-pentane.

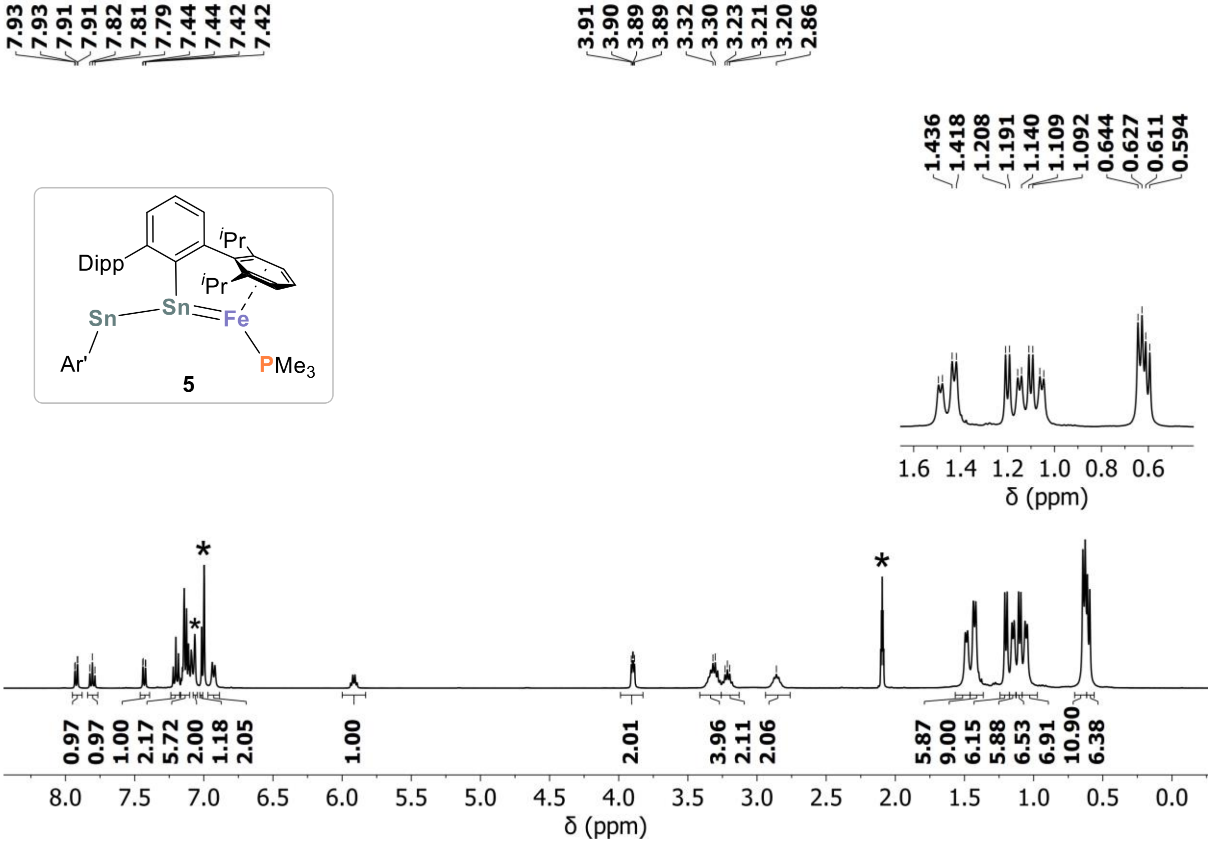

Figure S16. ^{1}H NMR (400 MHz, 233 K) spectrum of [(Ar'SnSnAr')Fe(PMe$_3$)] (**5**) in *d*$_8$-toluene. **d*$_8$-toluene.

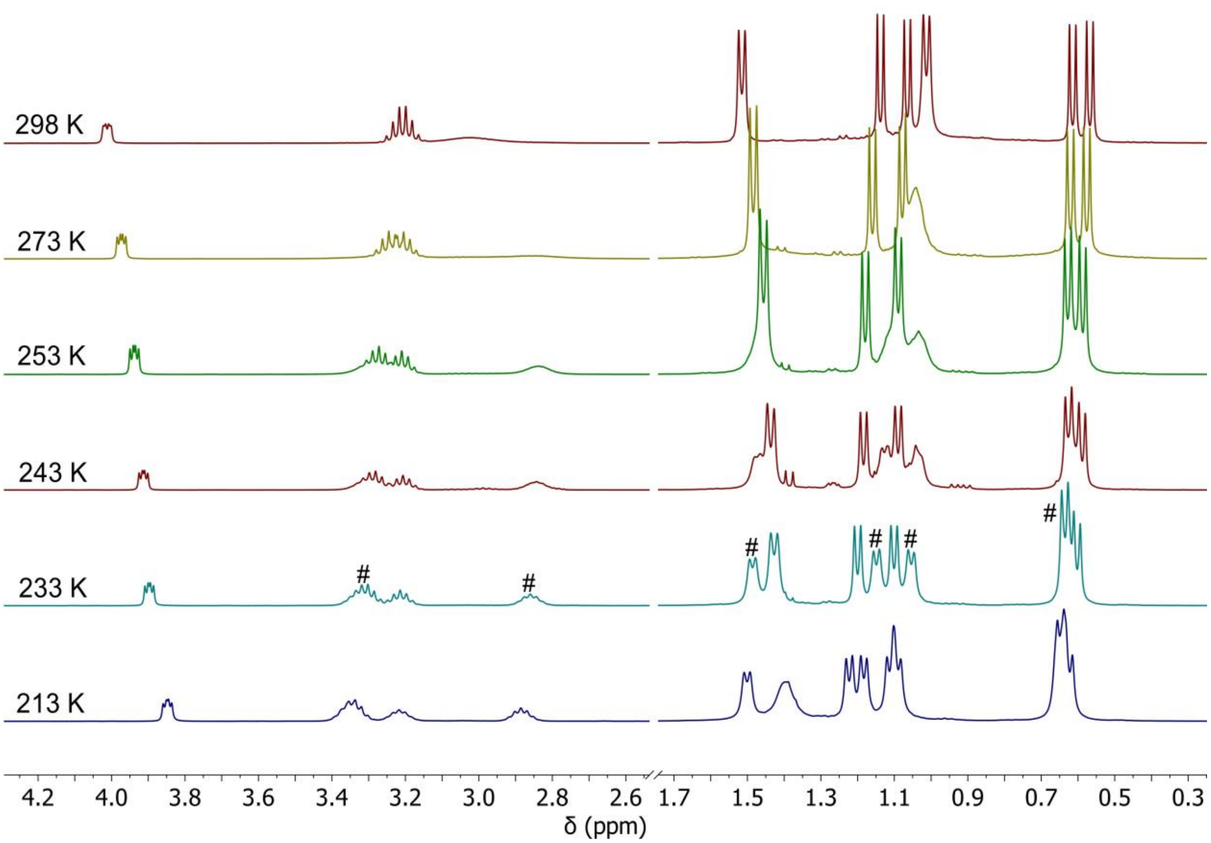

Figure S17. Variable-temperature (298 K–213 K) ^{1}H NMR spectra (0.3–1.7 ppm and 2.6–4.2 ppm) of [(Ar’SnSnAr’)Fe(PMe$_3$)] (**5**) in d_8-toluene (400 MHz). # Marks the new signals at 233 K.

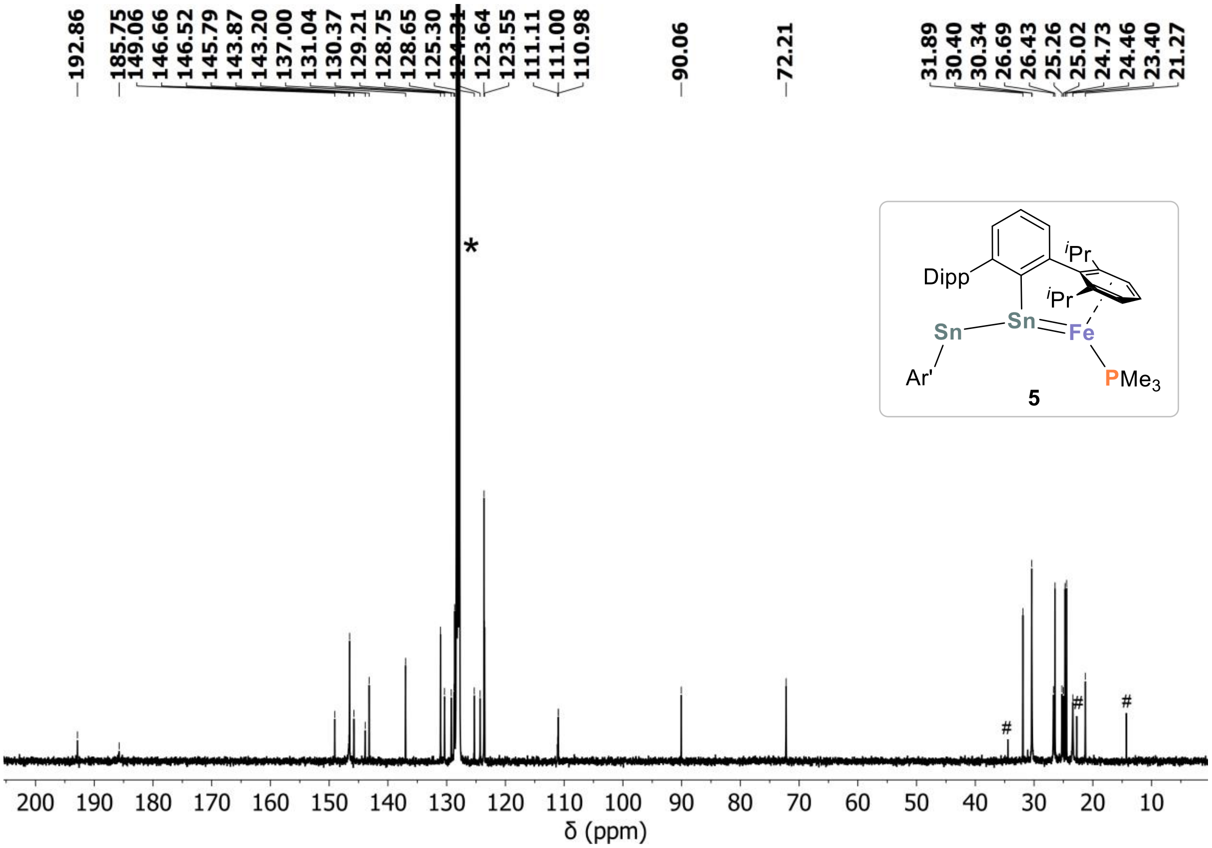

Figure S18. $^{13}C\{^{1}H\}$ NMR (100 MHz, 298 K) spectrum of [(Ar’SnSnAr’)Fe(PMe$_3$)] (**5**) in C_6D_6. *C_6D_6, #*n*-pentane.

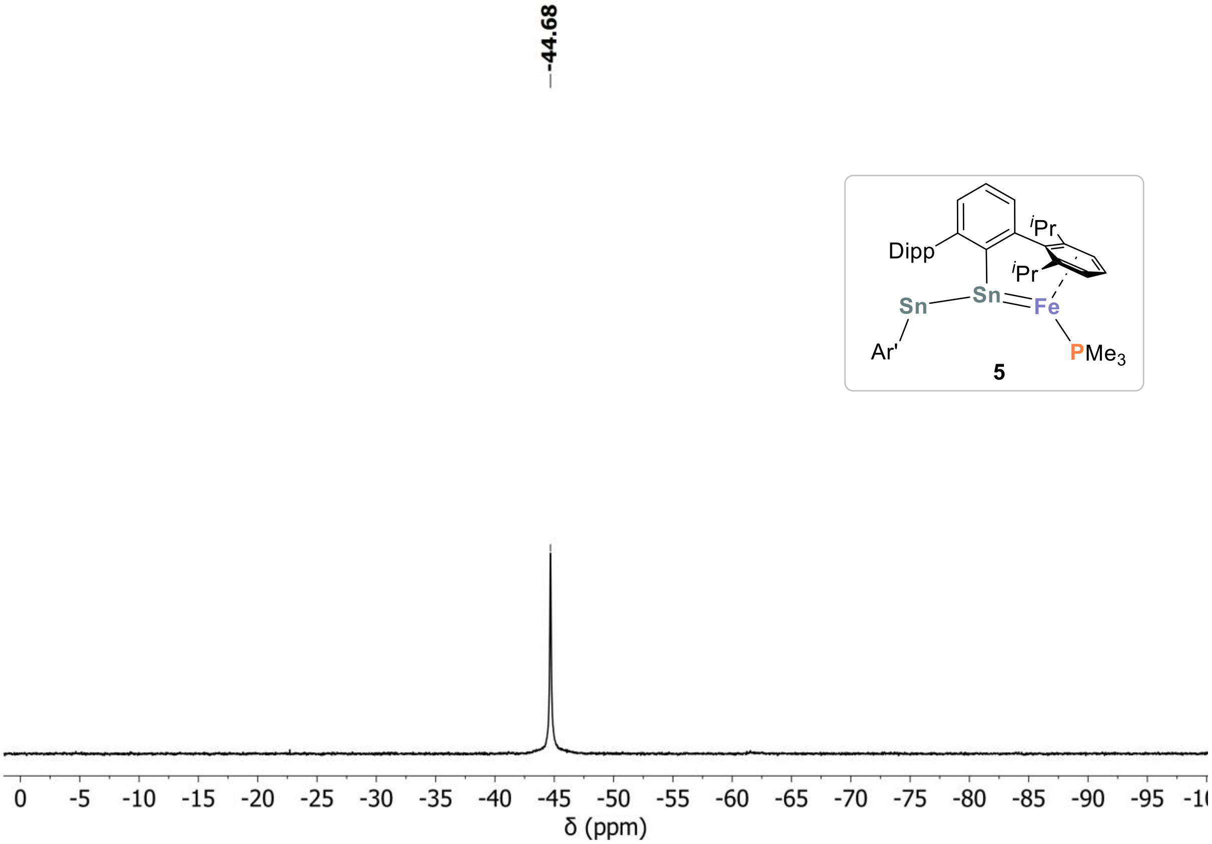

Figure S19. $^{31}P\{^{1}H\}$ NMR (162 MHz, 298 K) spectrum of [(Ar'SnSnAr')Fe(PMe$_3$)] (**5**) in C_6D_6.

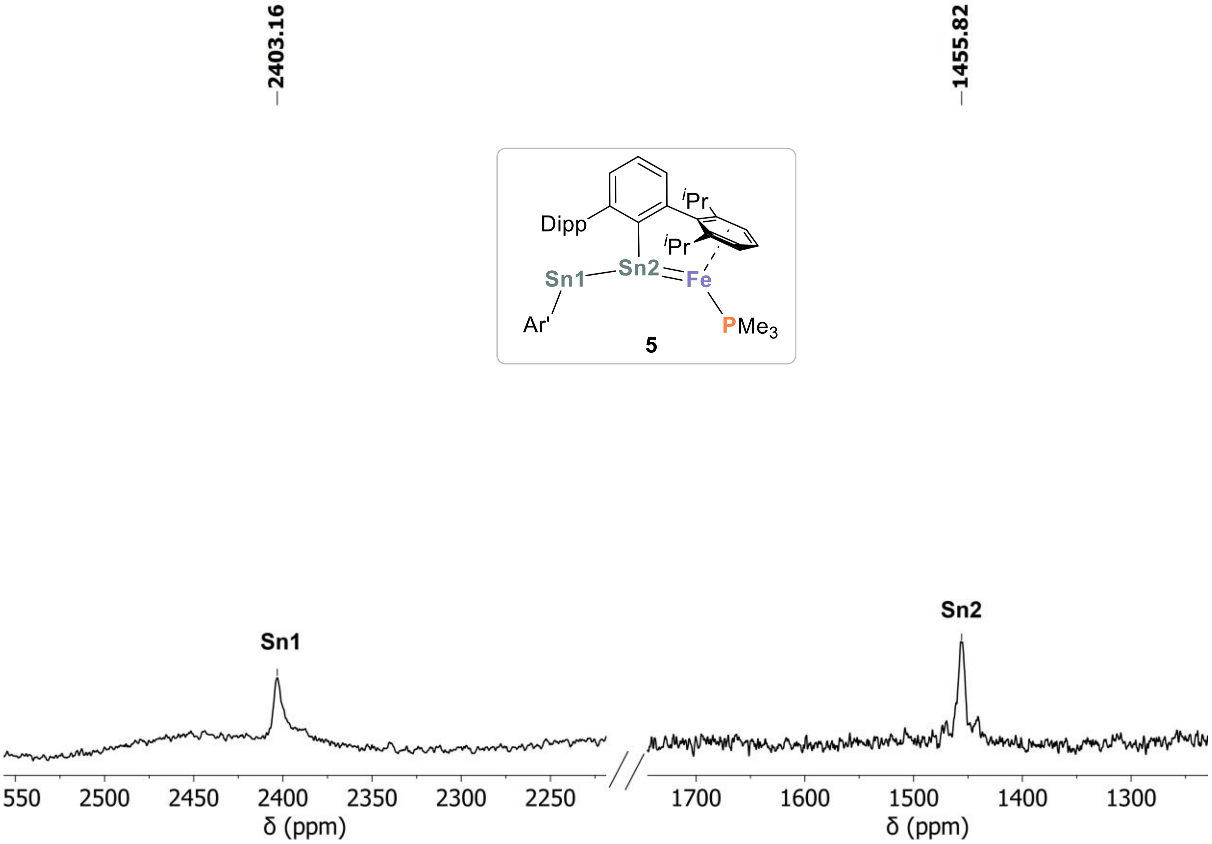

Figure S20. $^{119}Sn\{^{1}H\}$ NMR (224 MHz, 298 K) spectrum of [(Ar'SnSnAr')Fe(PMe$_3$)] (**5**) in C_6D_6.

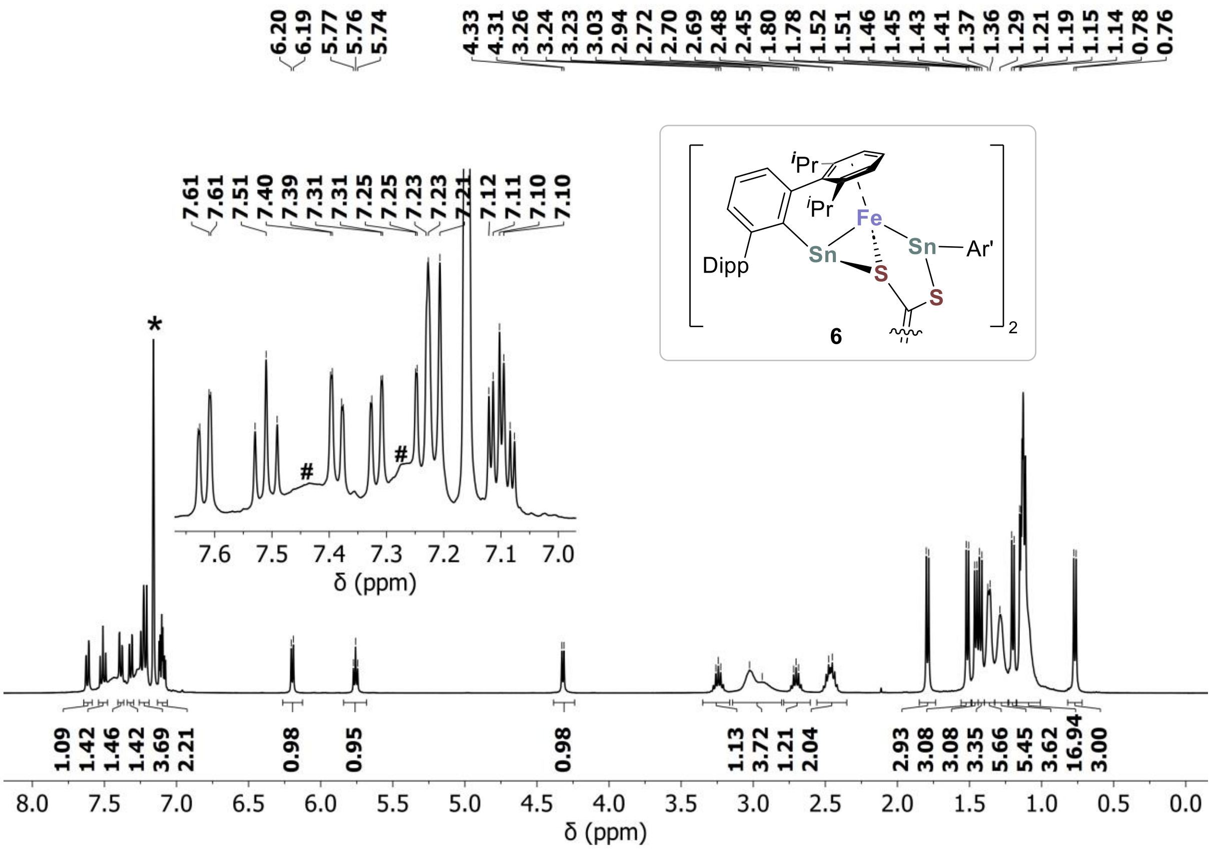

Figure S21. ^{1}H NMR (400 MHz, 298 K) spectrum of [((Ar'Sn)$_2$Fe)$_2$C$_2$S$_4$] (**6**) in C_6D_6. #Signal broadening due to the dynamic behavior of **6**. *C_6D_6.

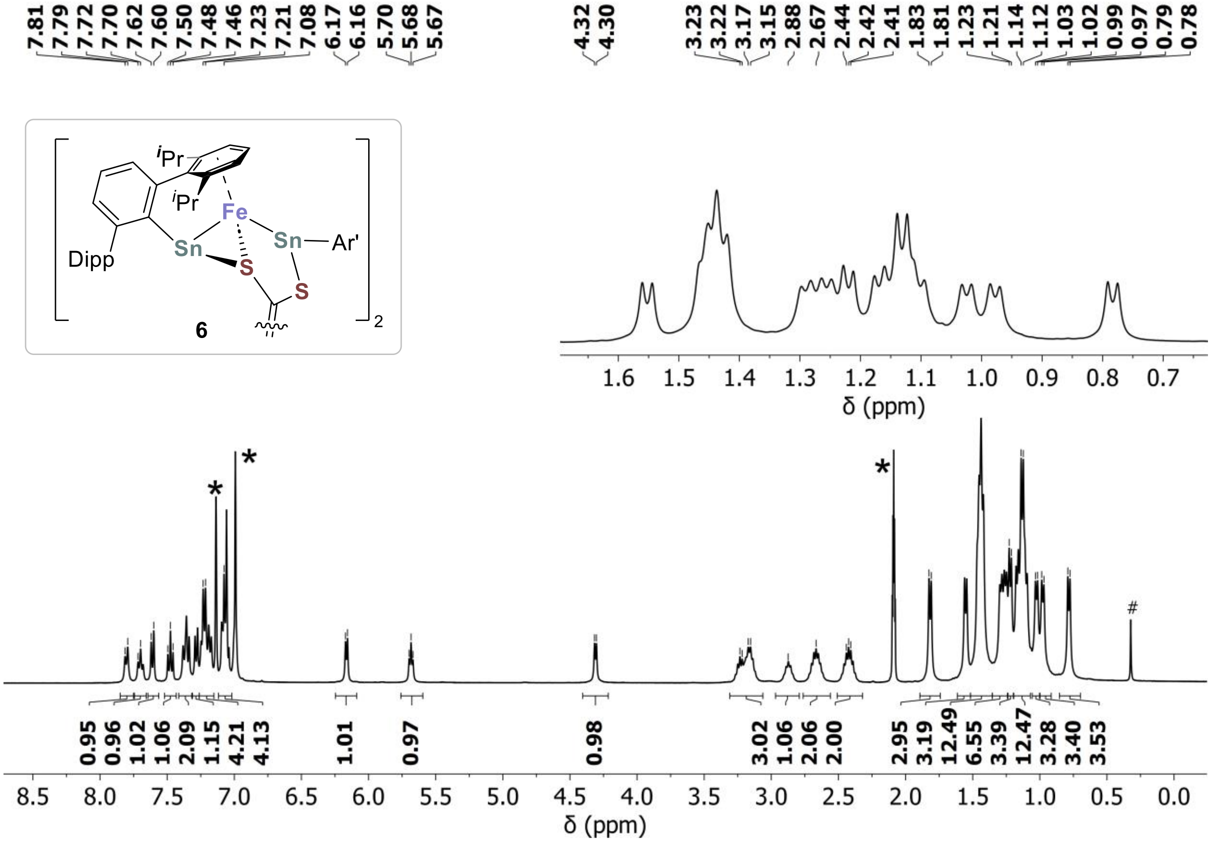

Figure S22. ^{1}H NMR (400 MHz, 233 K) spectrum of [((Ar'Sn)$_2$Fe)$_2$C$_2$S$_4$] (**6**) in d_8-toluene. *d_8-toluene, #grease.

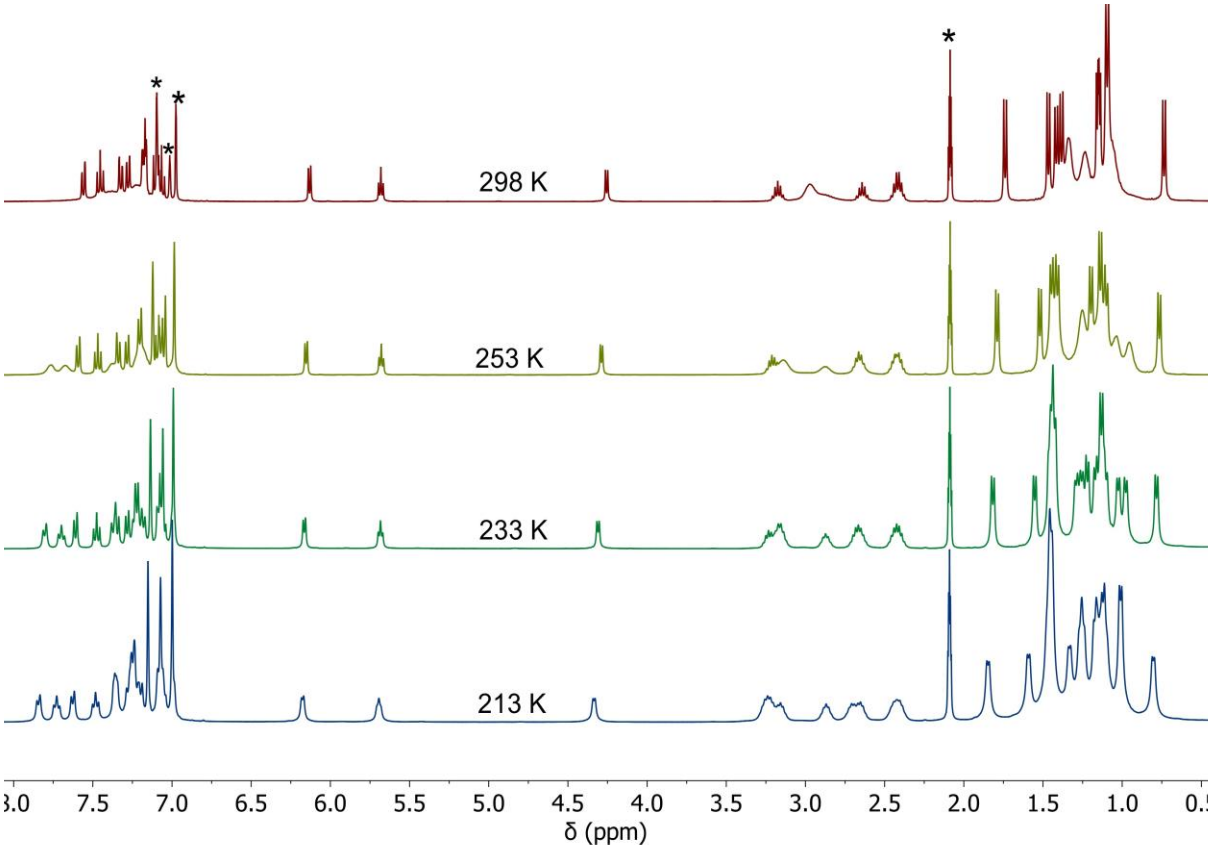

Figure S23. Variable-temperature (298 K–213 K) ^{1}H NMR spectra of [((Ar'Sn)$_2$Fe)$_2$C$_2$S$_4$] (**6**) in d_8-toluene (400 MHz). *d_8-toluene.

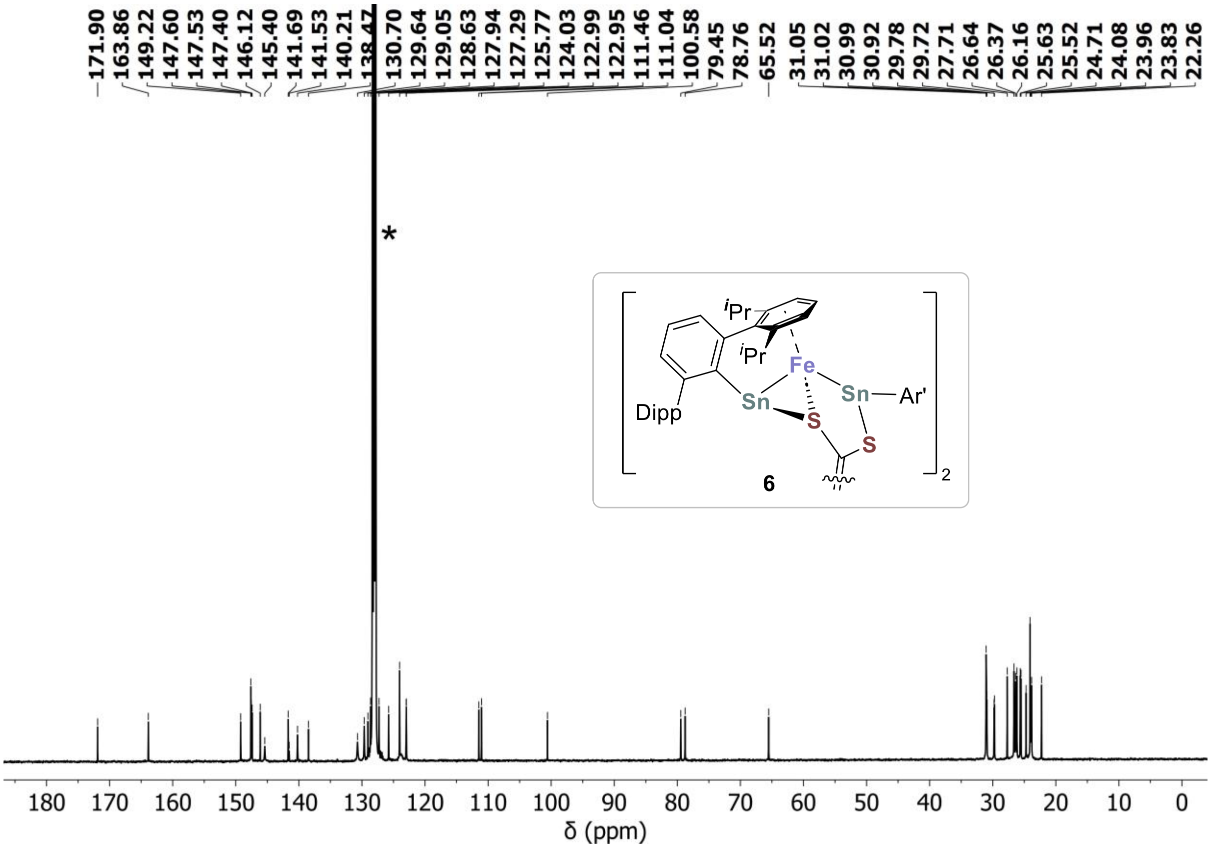

Figure S24 $^{13}C\{^{1}H\}$ NMR (100 MHz, 298 K) spectrum of [((Ar'Sn)$_2$Fe)$_2$C$_2$S$_4$] (**6**) in C_6D_6. *C_6D_6.

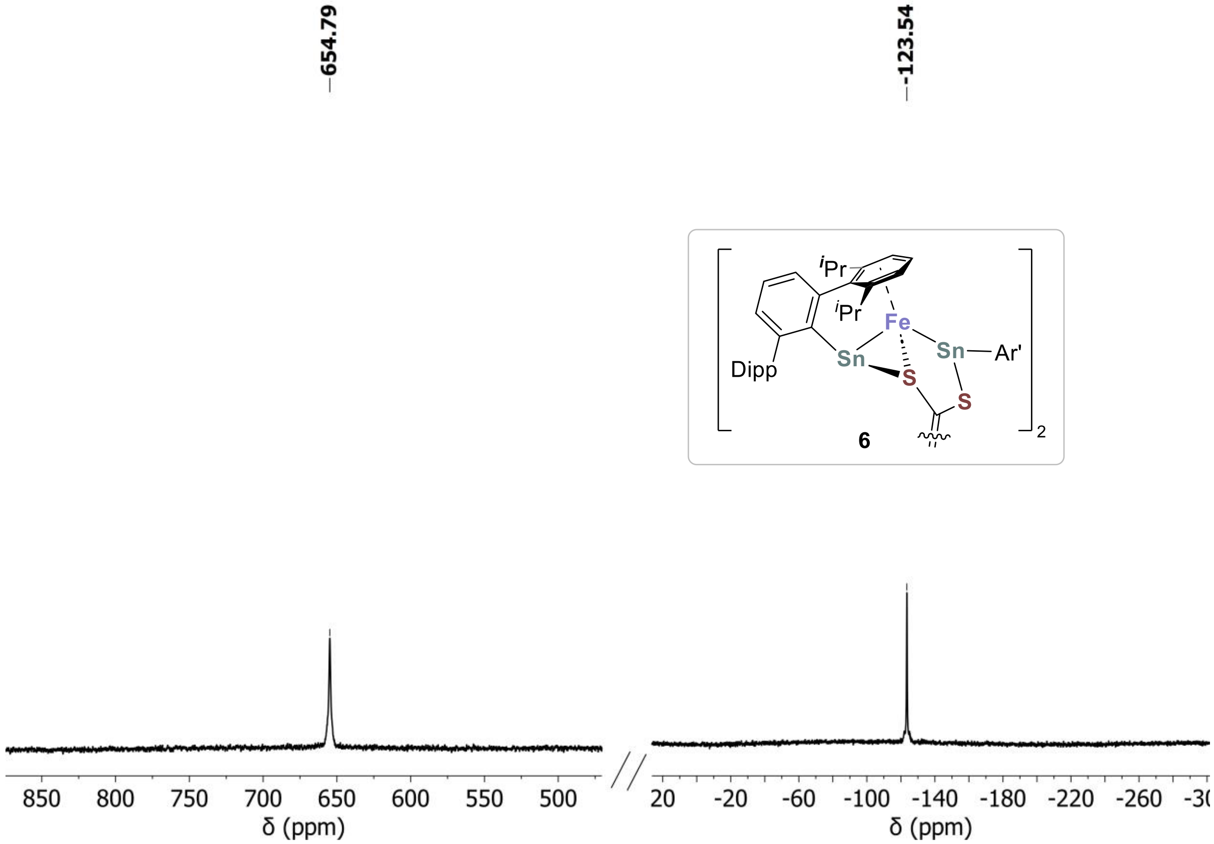

Figure S25. $^{119}Sn\{^{1}H\}$ NMR (224 MHz, 298 K) spectrum of $[((Ar'Sn)_2Fe)_2C_2S_4]$ (**6**) in C_6D_6.

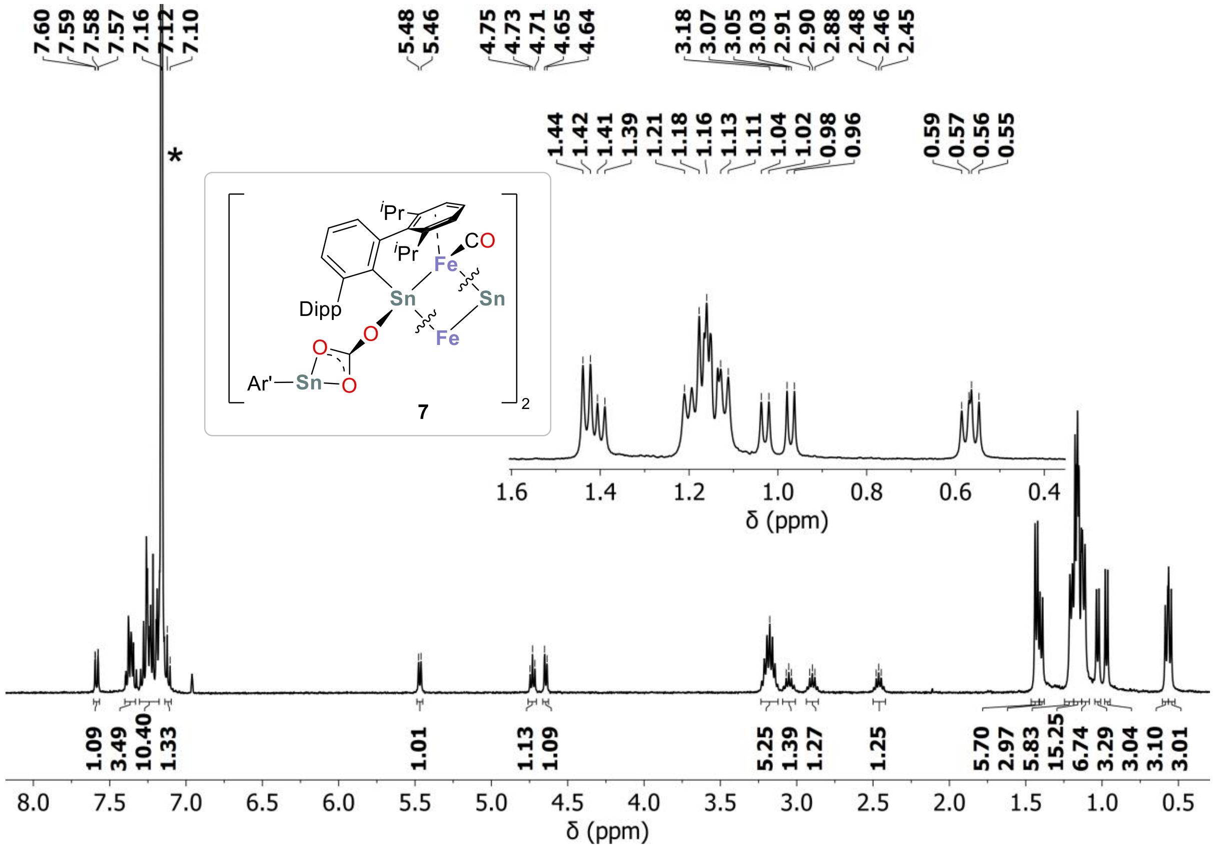

Figure S26. ^{1}H NMR (500 MHz, 298 K) spectrum of $[(Ar'SnCO_3SnAr')Fe(CO)]_2$ (**7**) in C_6D_6. *C_6D_6.

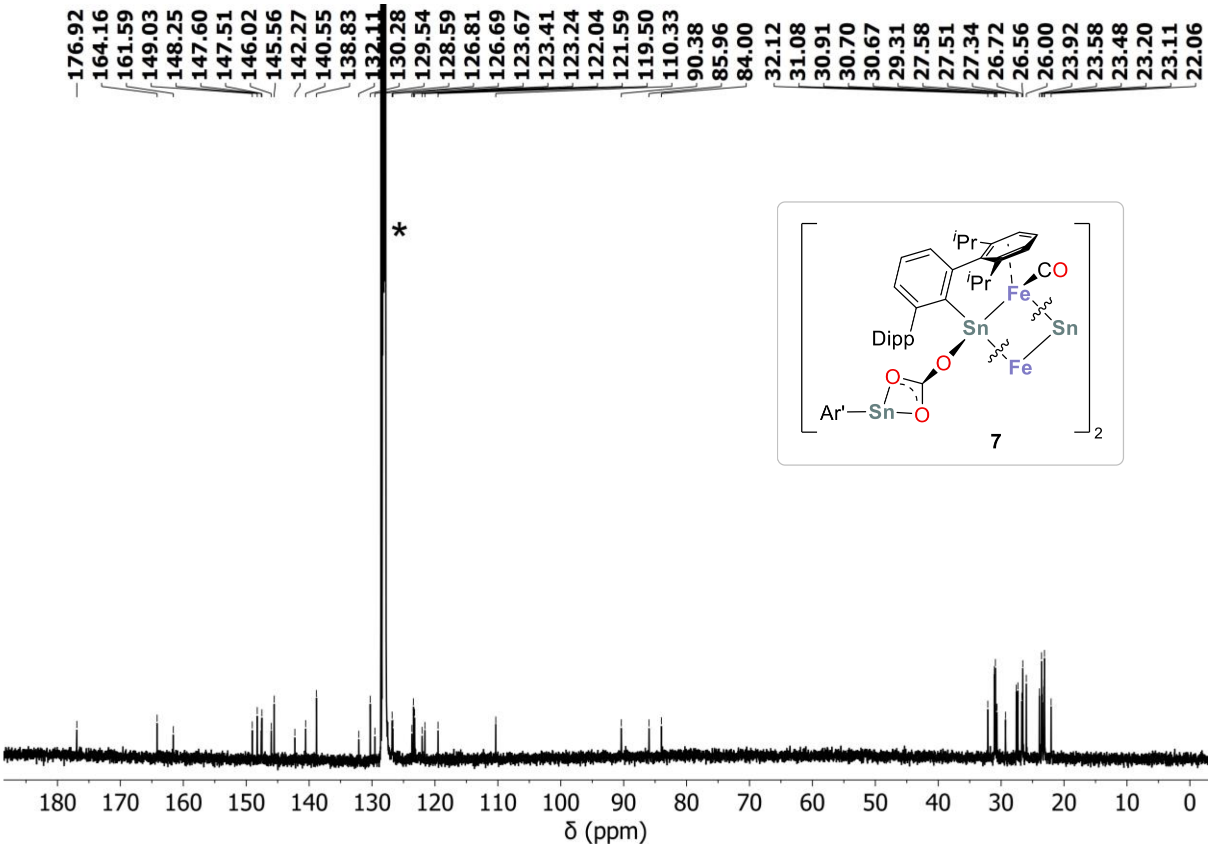

Figure S27. $^{13}C\{^1H\}$ NMR (125 MHz, 298 K) spectrum of $[(Ar'SnCO_3SnAr')Fe(CO)]_2$ (**7**) in C_6D_6. $*C_6D_6$.

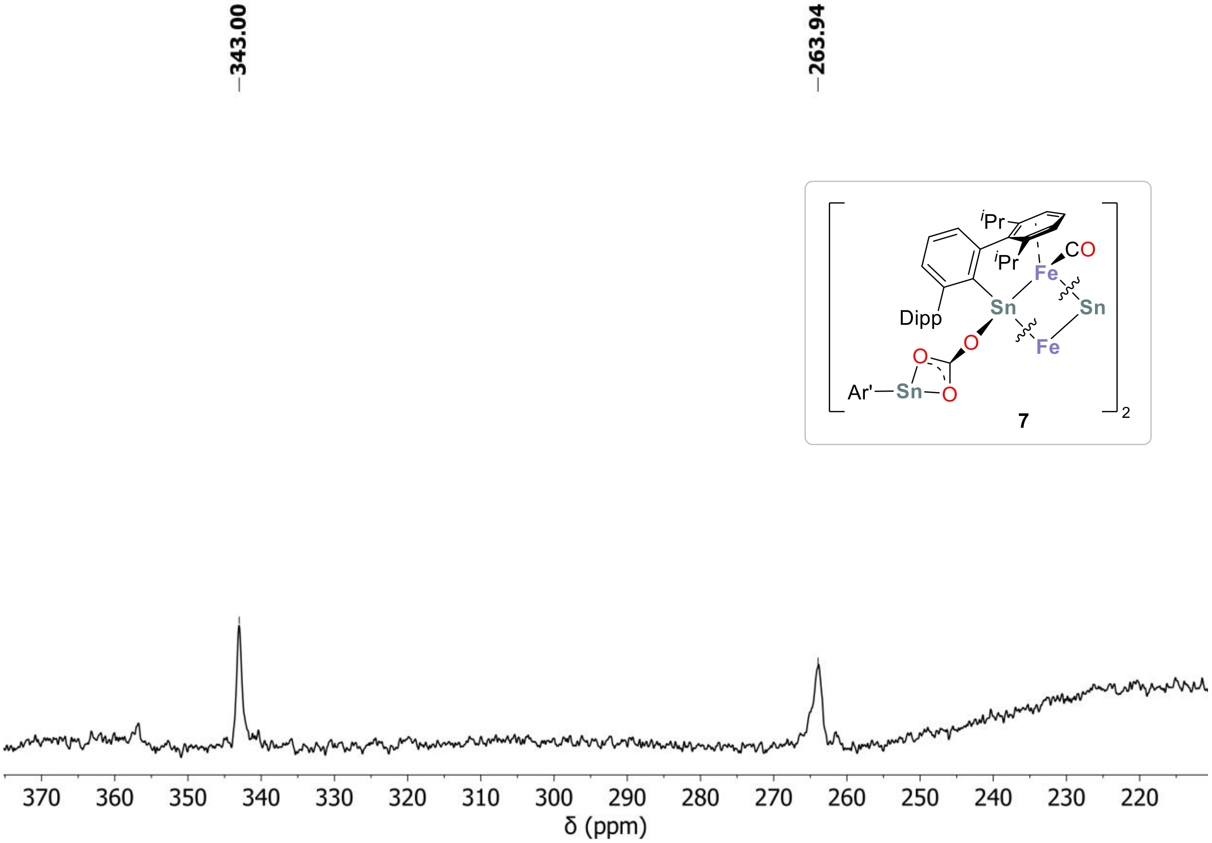

Figure S28. $^{119}Sn\{^1H\}$ NMR (224 MHz, 298 K) spectrum of $[(Ar'SnCO_3SnAr')Fe(CO)]_2$ (**7**) in C_6D_6.

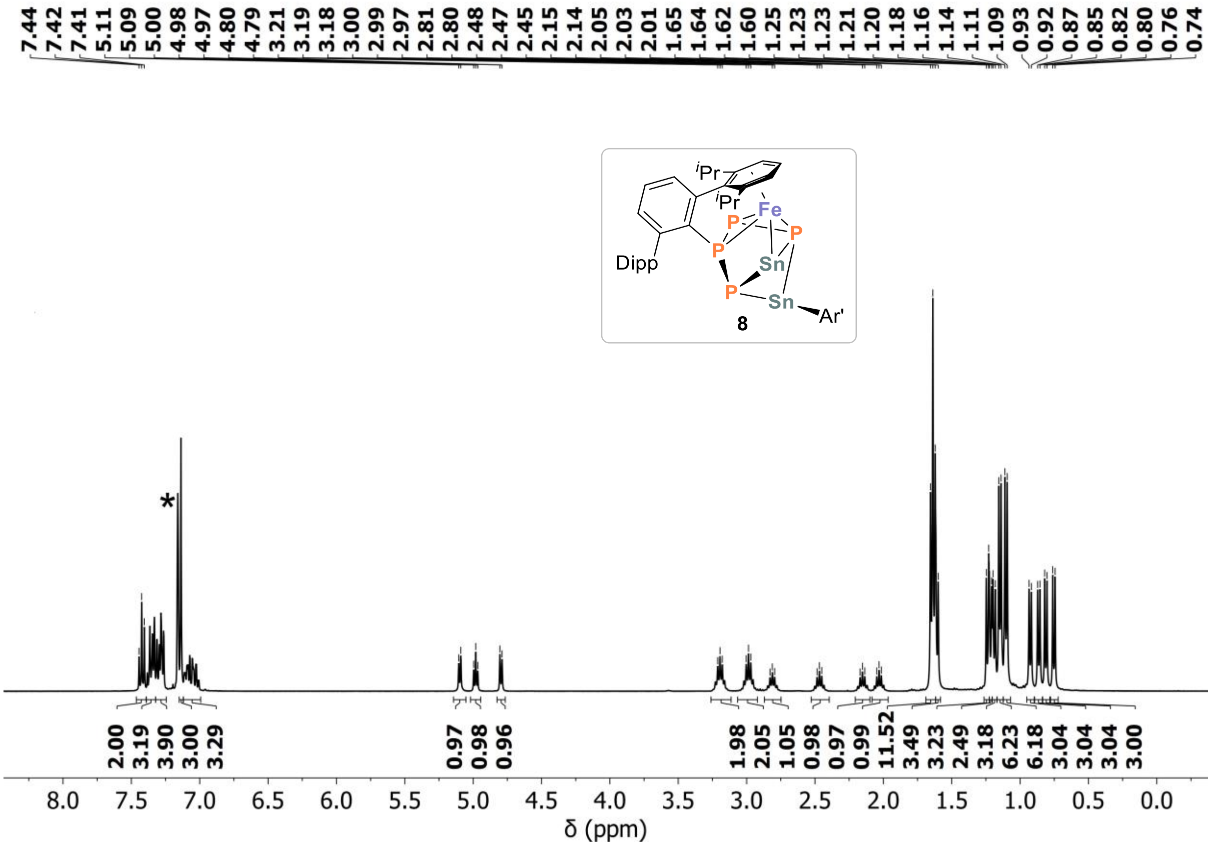

Figure S29. ^{1}H NMR (400 MHz, 298 K) spectrum of [(Ar'P_4Sn_2Ar')Fe] (**8**) in C_6D_6. *C_6D_6.

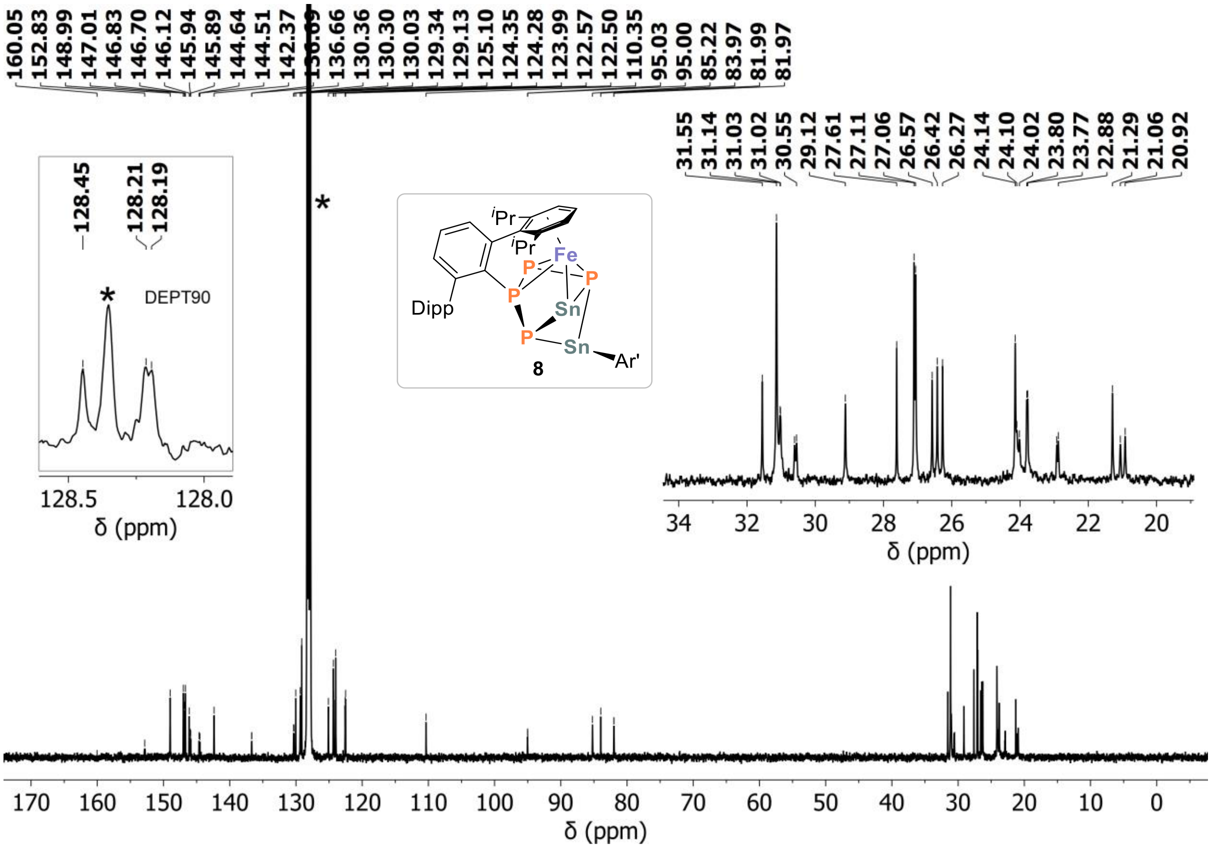

Figure S30. $^{13}C\{^{1}H\}$ NMR (125 MHz, 298 K) spectrum of [(Ar'P_4Sn_2Ar')Fe] (**8**) in C_6D_6. Inset: DEPT90 ^{13}C NMR spectrum of **8** in C_6D_6, showing the signals that overlap with the residual signal of C_6D_6 in the standard $^{13}C\{^{1}H\}$ NMR spectrum. *C_6D_6.

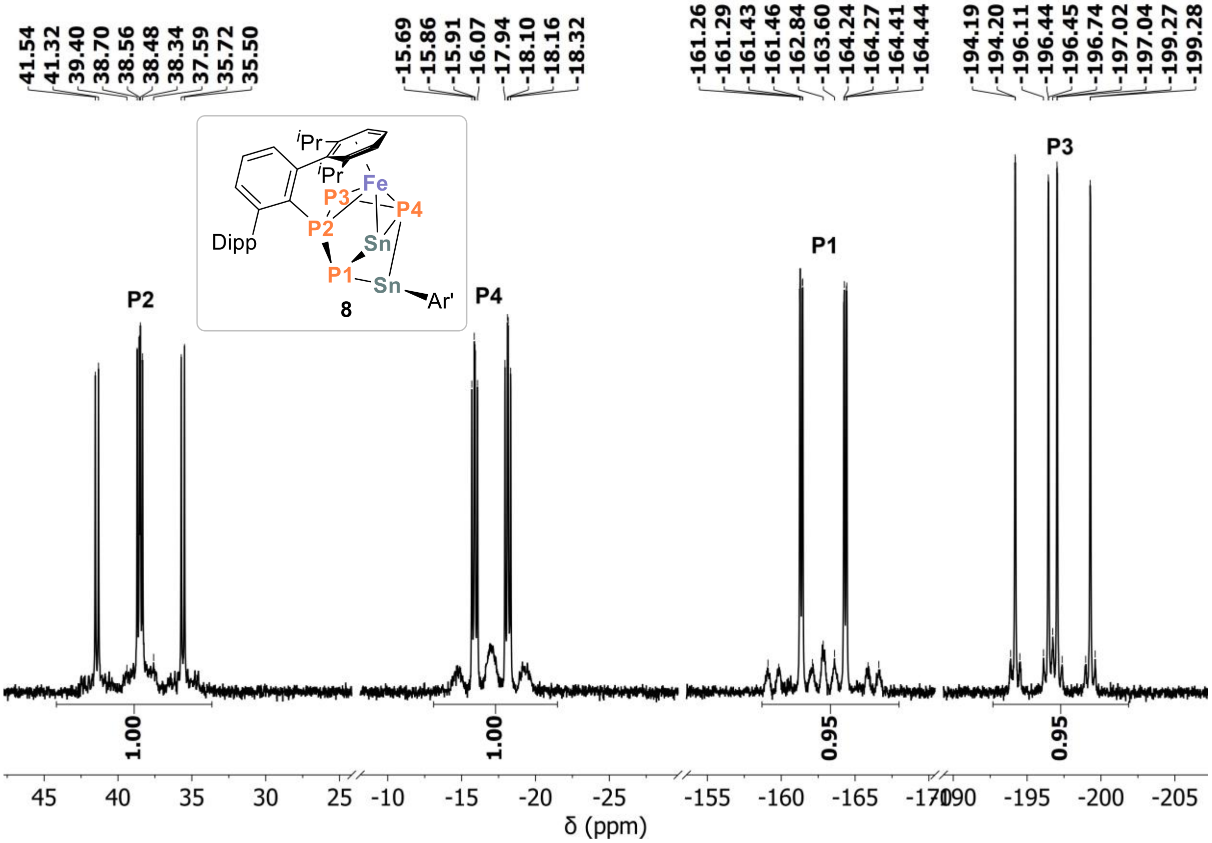

Figure S31. $^{31}P\{^1H\}$ NMR (162 MHz, 298 K) spectrum of [(Ar'P$_4$Sn$_2$Ar')Fe] (**8**) in C_6D_6.

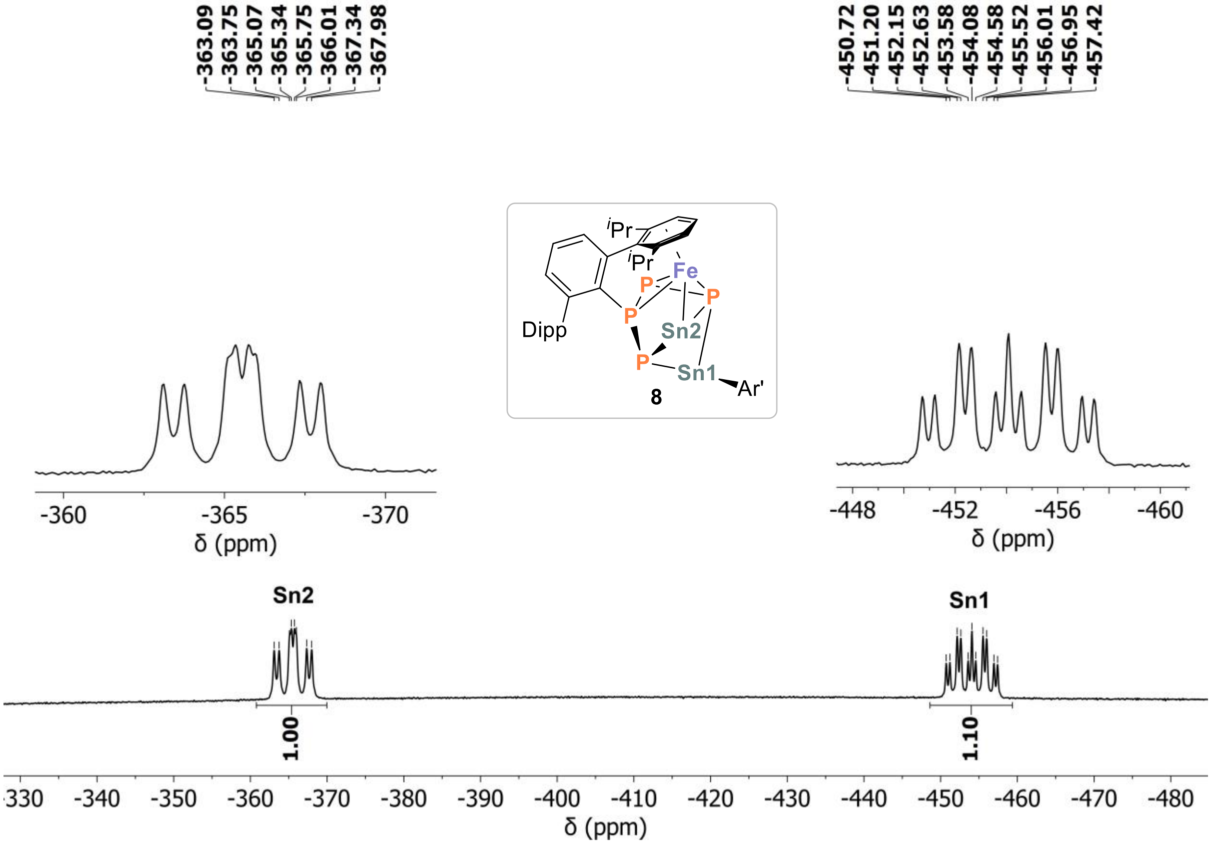

Figure S32. $^{119}Sn\{^1H\}$ NMR (224 MHz, 298 K) spectrum of [(Ar'P$_4$Sn$_2$Ar')Fe] (**8**) in C_6D_6.

NMR Simulations

The $^{31}P\{^1H\}$ and $^{119}Sn\{^1H\}$ NMR spectrum of compound **8** was fitted using the full lineshape iteration of gNMR (version 5.0.6, by Cherwell Scientific) to determine the ^{31}P-^{31}P and ^{31}P-$^{119/117}Sn$ coupling constants.[4] Initial coupling constants were extracted from the experimental data (Figures S31 and S32) and were all set to positive values. Natural abundance isotopomer mixture of tin were used for the simulation, but interpret all couplings as given for ^{119}Sn.

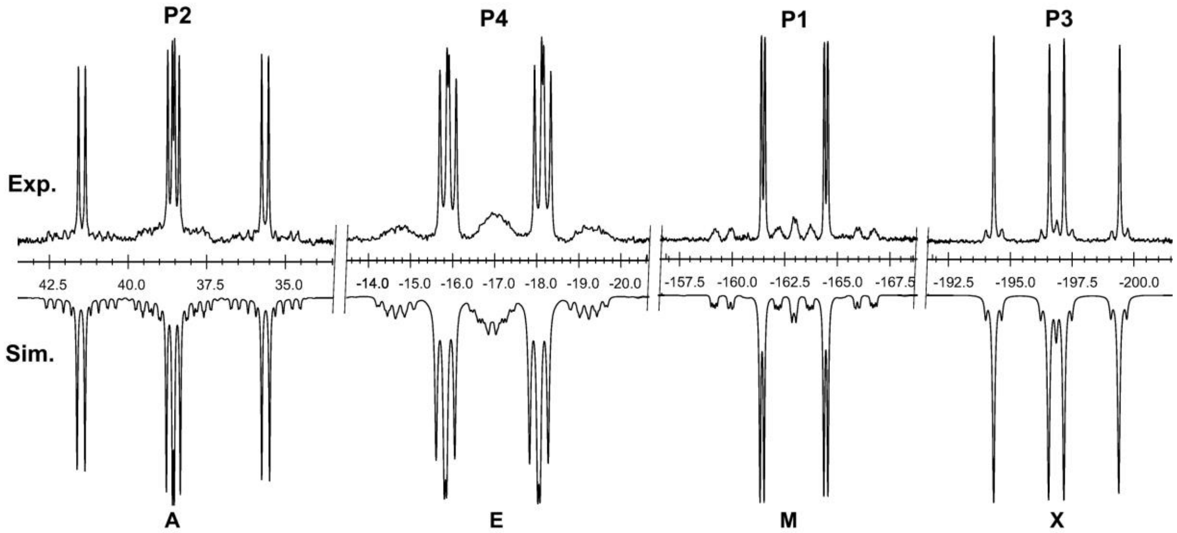

Figure S33. Measured (upward) and simulated (downward) $^{31}P\{^1H\}$ NMR spectra of **8** in C_6D_6.

Table S1. Chemical shifts (ppm) and $J_{P\text{-}P}$ and $J_{Sn\text{-}P}$ coupling constants (Hz) derived from the iterative fit of AEMX spin system for the $^{31}P\{^1H\}$ NMR spectra of **8**.

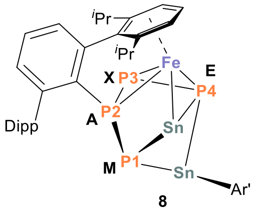 8	δ(A) = 38.5	J_{AM} = 490.9, J_{AX} = 459.6, J_{AE} = 40.6	$J_{A\text{-}Sn}$ = 321.1, 143.6
	δ(E) = −162.9	J_{EM} = 31.1, J_{EX} = 359.5	$J_{E\text{-}Sn}$ = 753.1, 492.2
	δ(M) = −16.9	J_{MX} = 2.9	$J_{M\text{-}Sn}$ = 456.8, 321.1
	δ(X) = −196.8		$J_{X\text{-}Sn}$ = 107.3

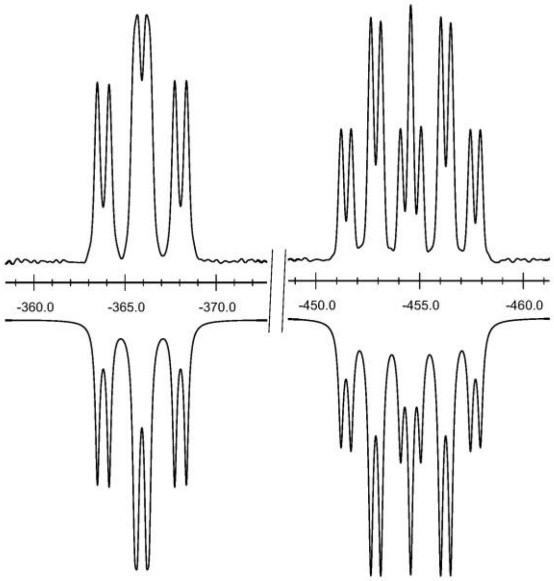

Figure S34. Measured (upward) and simulated (downward) ^{119}Sn{^{1}H} NMR spectra of **8** in C_6D_6.

Table S2. Chemical shifts (ppm) and $J_{Sn\text{-}P}$ coupling constants (Hz) derived from the iterative fit of AB spin system for the ^{119}Sn{^{1}H} NMR spectra of **8**.

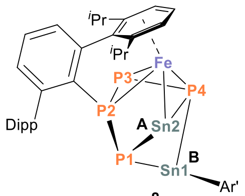 **8**	δ(A) = −365.9	$J_{A\text{-}P}$ = 492.2, 456.8, 143.6
	δ(B) = −454.6	$J_{B\text{-}P}$ = 753.1, 321.1, 321.1, 107.3

^{1}H NMR Spectroscopy and van't Hoff Analysis of the Reaction of 1 with PMe_3

To a C_6D_6 (0.5 mL) solution of **1** (6.2 mg, 5.7 µmol, 11.4 mM) in a *J. Young* NMR tube was added PMe_3 (*ca.* 0.57 M in C_6D_6, 5.2 µmol, 0.91 eq) at room temperature. The exact amount of PMe_3 added was calculated based on the ^{1}H NMR data collected immediately after the preparation of the sample. The reaction solution was heated to 308 K in an oil bath and the reaction progress was monitored by ^{1}H NMR spectroscopy. The integral of the signal of the para-hydrogen atom of the Dipp-ring coordinating to iron was used for determining the equilibrium constant. The equilibrium was considered to be reached when there was no obvious change of the integral ratio in the reference signals of **1** and **5** (product) in two consecutive NMR measurements with ≥6 h time interval. Due to the long time needed to reach the equilibrium (> 6 h at 343 K), all the ^{1}H NMR data were collected at room temperature (298 K) within 10 min after the NMR tube was taken out of the oil bath. **Note:** Although complex **1** is stable at 80 °C for several days, weak signals attributed to the distannyne Ar'SnSnAr' could be observed after heating the reaction mixture at 343 K for 20 h, which might be due to the presence of PMe_3.

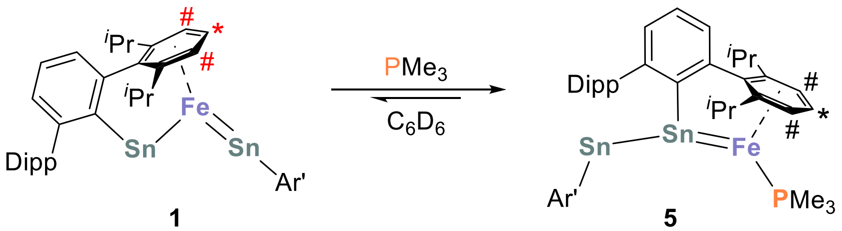

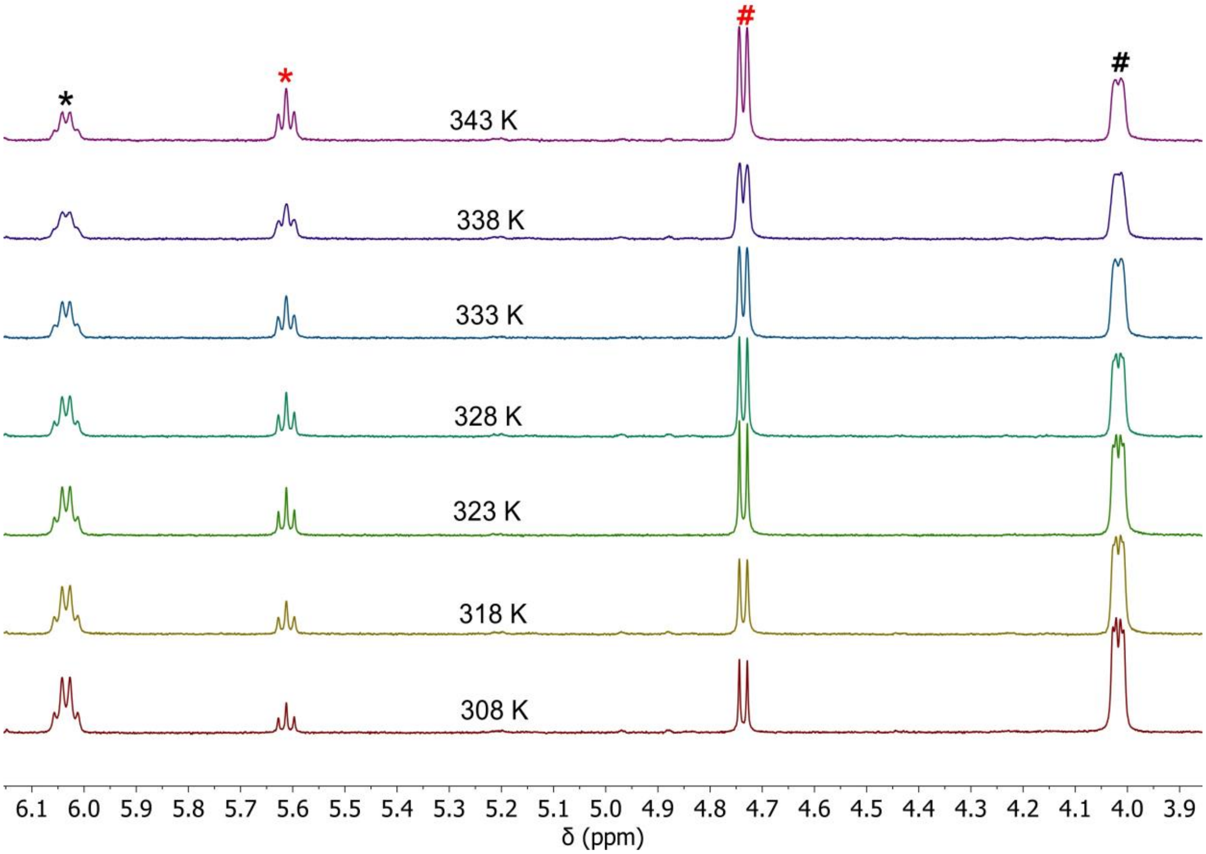

Figure S35. Stacked ^{1}H NMR spectra (3.9–6.1 ppm) for the reaction of **1** with PMe_3 at chemical equilibrium (308 K–343 K). The para- and meta-hydrogen atoms of the coordinating Dipp-ring from **1**/**5** are marked by red/black * and #, respectively.

Table S3. Calculated values for K_{eq} at different temperatures.

**[1]**$_0$ (mM)[a]	**[1]**$_e$(mM)[b]	T (K)	1/T (K^{-1})	K_{eq} (M^{-1})	InK_{eq}(M^{-1})
11.4	2.5612	308	0.00324675	2247.883	7.719
11.4	3.2258	318	0.00314465	1151.920	7.050
11.4	3.7353	323	0.00309598	757.404	6.631
11.4	4.2537	328	0.00304878	520.489	6.255
11.4	4.6760	333	0.00300300	393.976	5.977
11.4	5.2949	338	0.00295858	270.091	5.599
11.4	5.8612	343	0.00291545	195.442	5.276

[a]Initial concentration. [b]Equilibrium concentration.

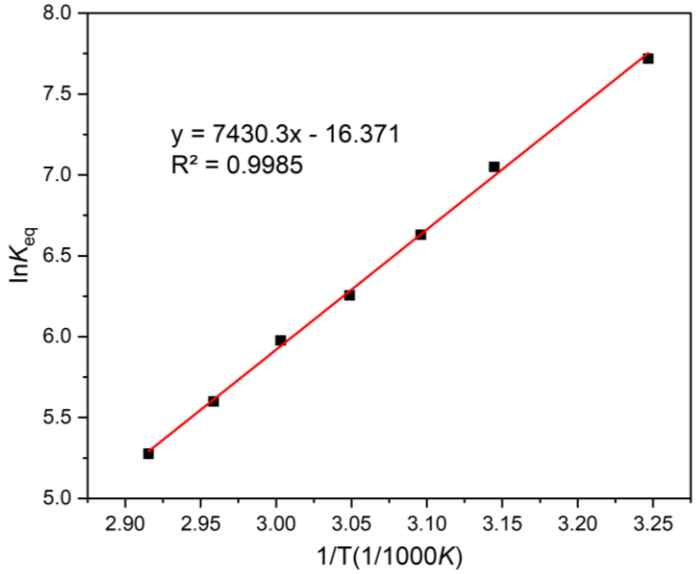

Figure S36. van't Hoff plot for the determination the ΔH° and ΔS° of reaction of **1** with PMe_3. The following values were determined: ΔH° = −14.8 ± 0.3 kcal mol^{-1}, ΔS° = −32.5 ± 0.8 cal mol^{-1} K^{-1} and ΔG°(298 K) = −5.08 ± 0.02 kcal mol^{-1}.

UV-vis Spectra

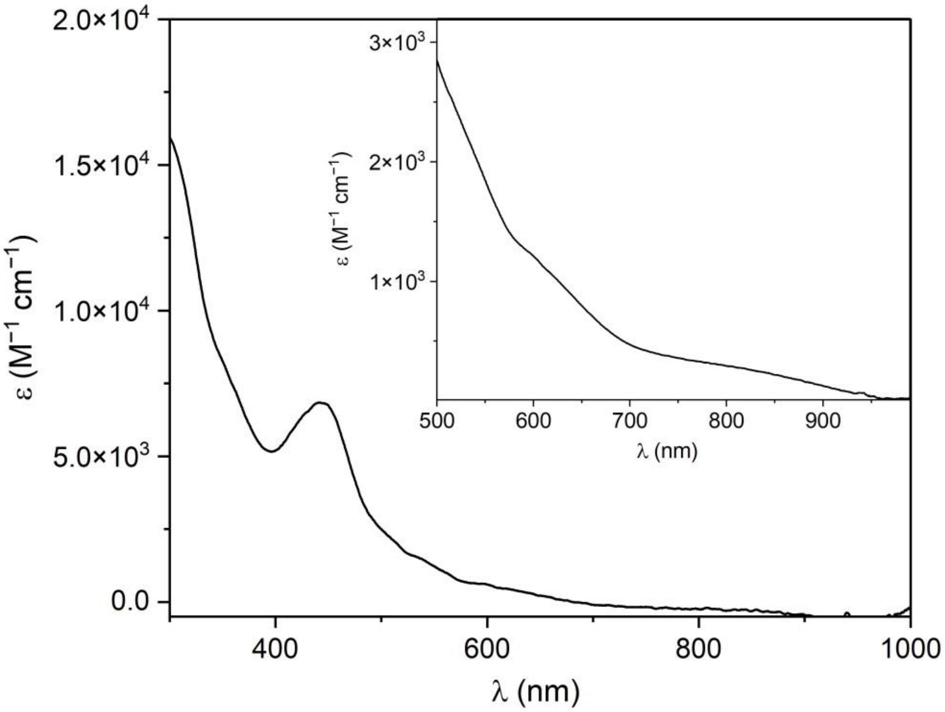

Figure S37. UV-vis spectra of **1** recorded in toluene (*ca.* 0.05 mM, inset: *ca.* 0.5 mM) at 25 °C.

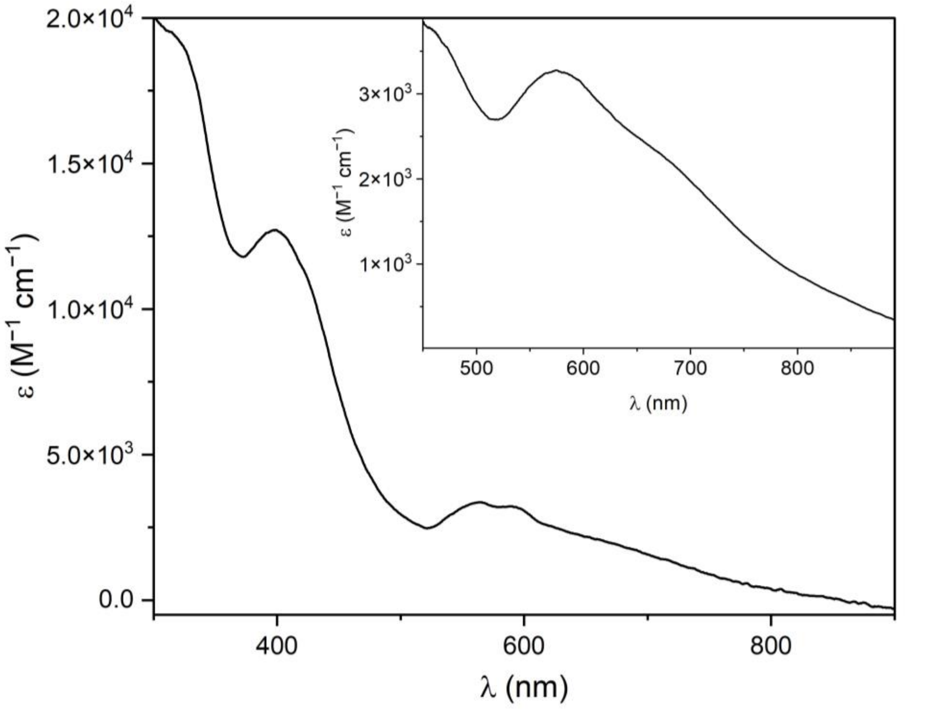

Figure S38. UV-vis spectra of **2** recorded in toluene (*ca.* 0.05 mM, inset: *ca.* 0.5 mM) at 25 °C.

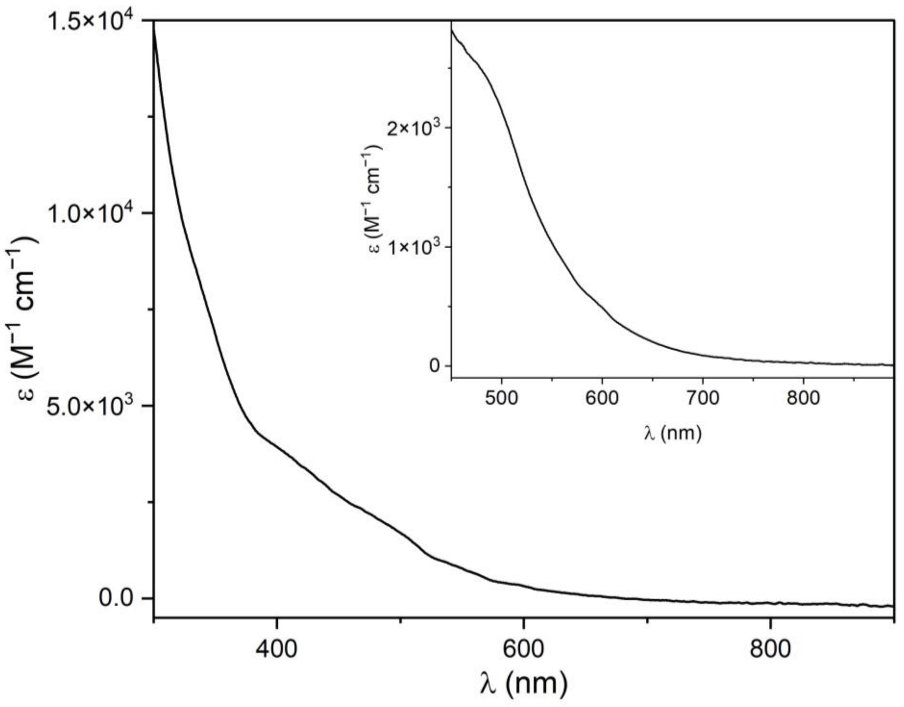

Figure S39. UV-vis spectra of **3** recorded in toluene (*ca.* 0.1 mM, inset: *ca.* 0.5 mM) at 25 °C.

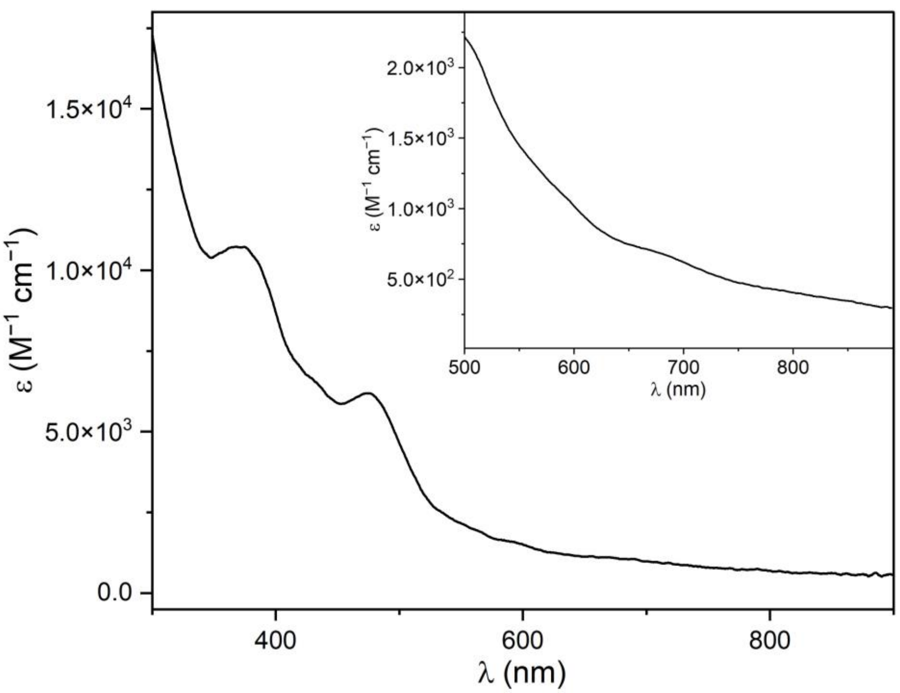

Figure S40. UV-vis spectra of **4** recorded in toluene (*ca.* 0.05 mM, inset: *ca.* 0.5 mM) at 25 °C.

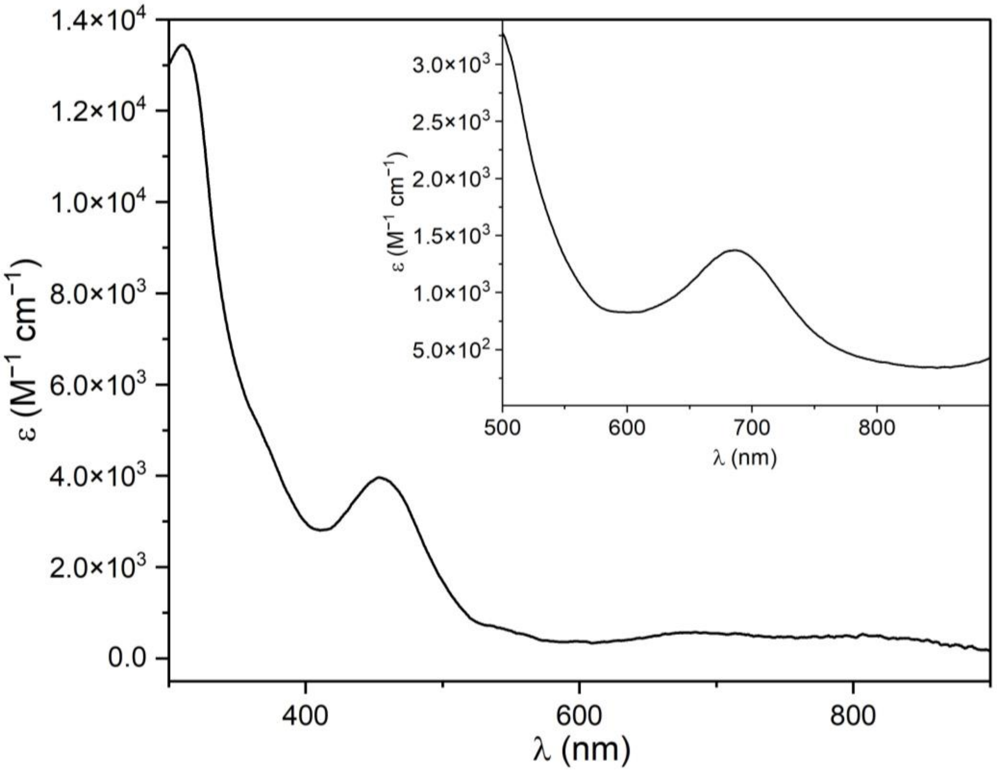

Figure S41. UV-vis spectra of **5** recorded in toluene (*ca.* 0.05 mM, inset: *ca.* 0.5 mM) at 25 °C.

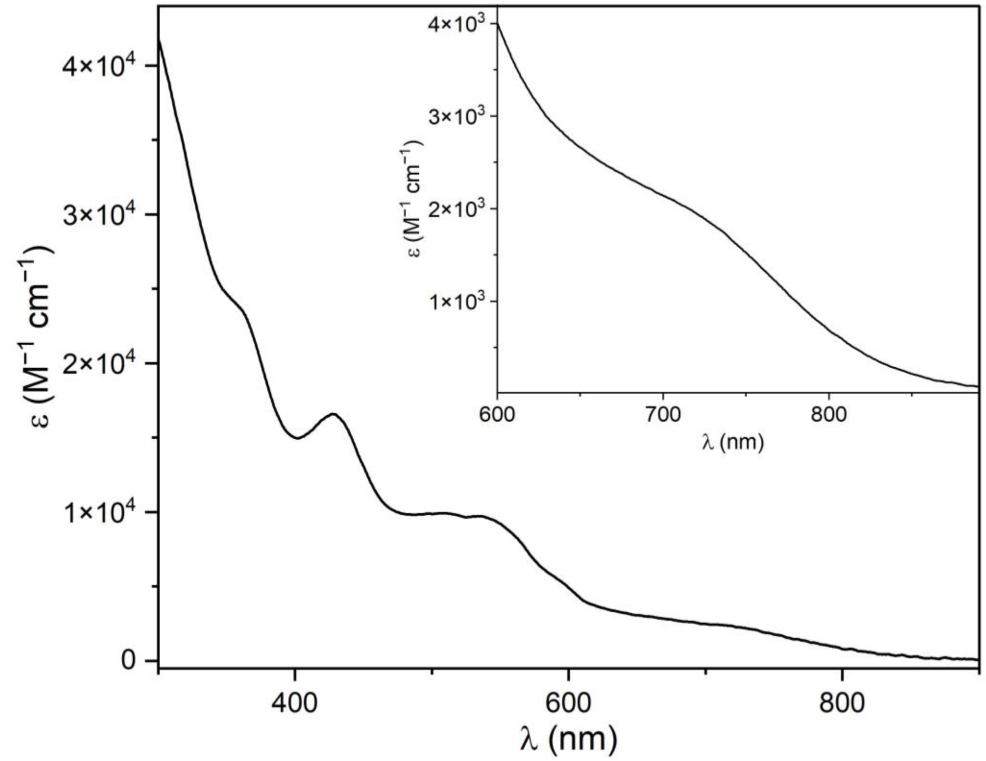

Figure S42. UV-vis spectra of **6** recorded in toluene (*ca.* 0.03 mM, inset: *ca.* 0.3 mM) at 25 °C.

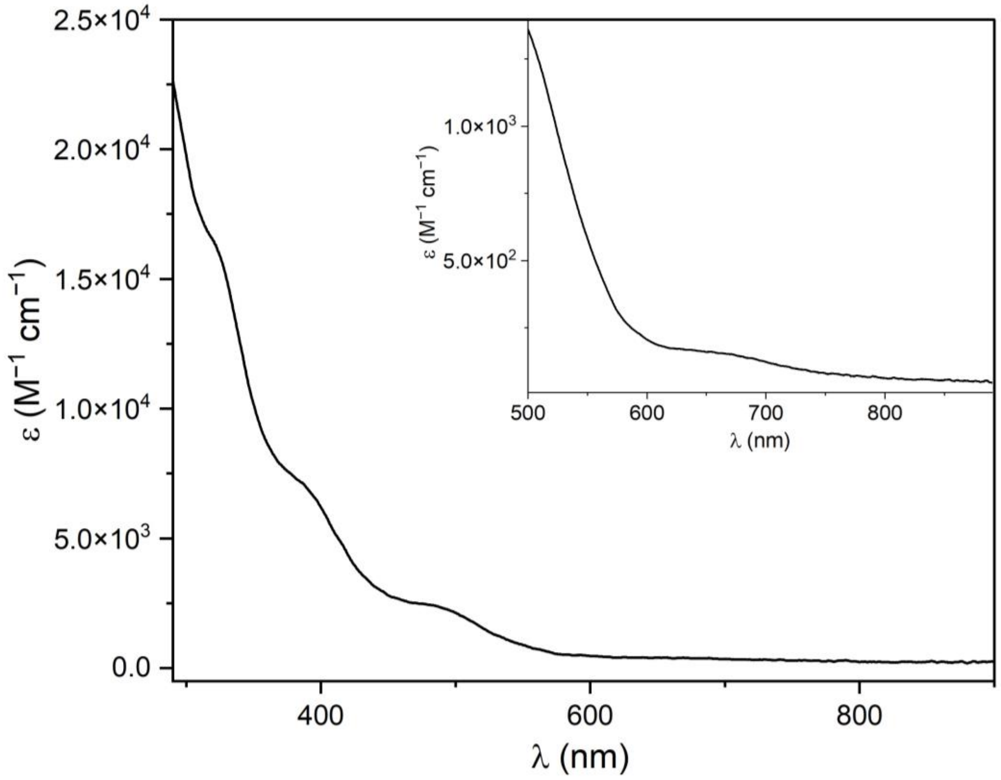

Figure S43. UV-vis spectra of **7** recorded in toluene (*ca.* 0.05 mM, inset: *ca.* 0.5 mM) at 25 °C.

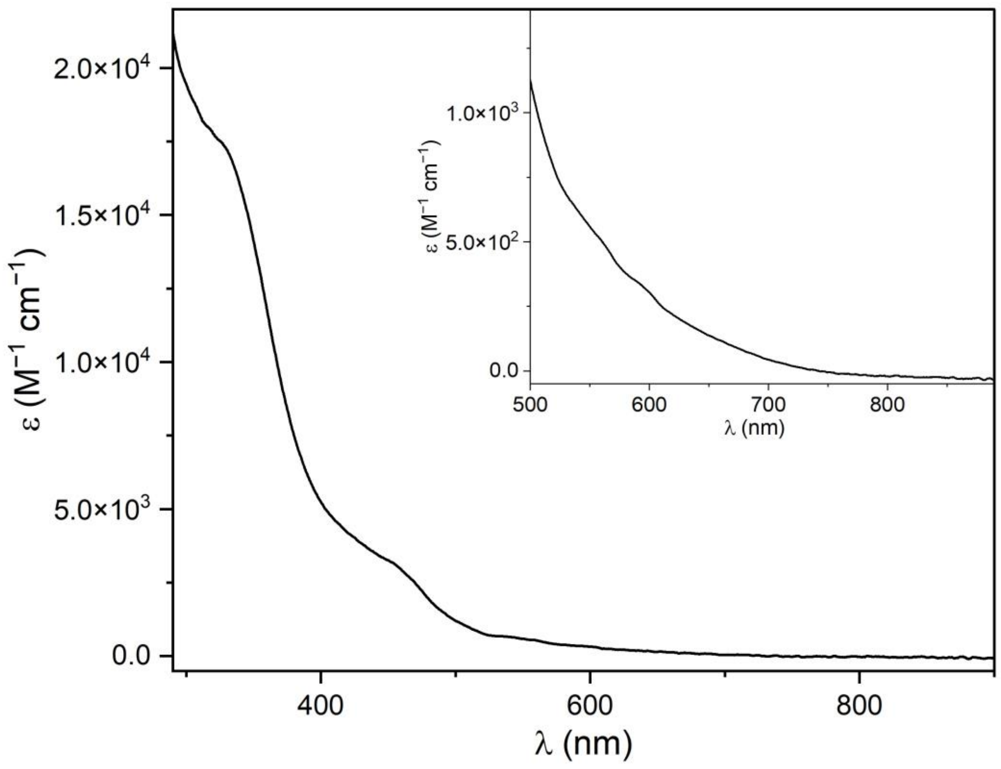

Figure S44. UV-vis spectra of **8** recorded in toluene (*ca.* 0.05 mM, inset: *ca.* 0.5 mM) at 25 °C.

LIFDI-Mass Spectra

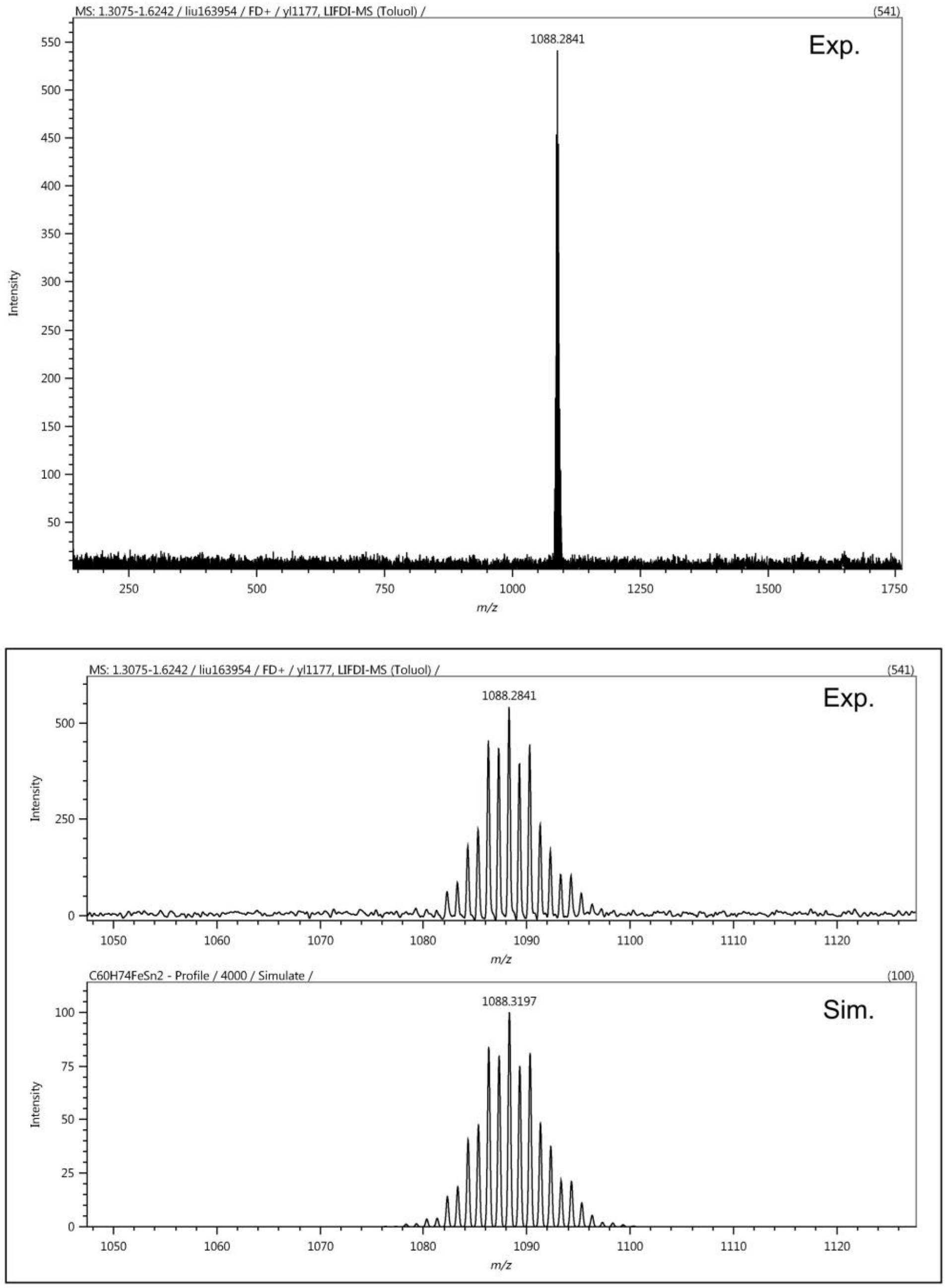

Figure S45. LIFDI–MS spectrum (toluene) of **1** (top) and the isotope pattern and a simulation for the peak around m/z = 1088 $[M]^+$ (bottom).

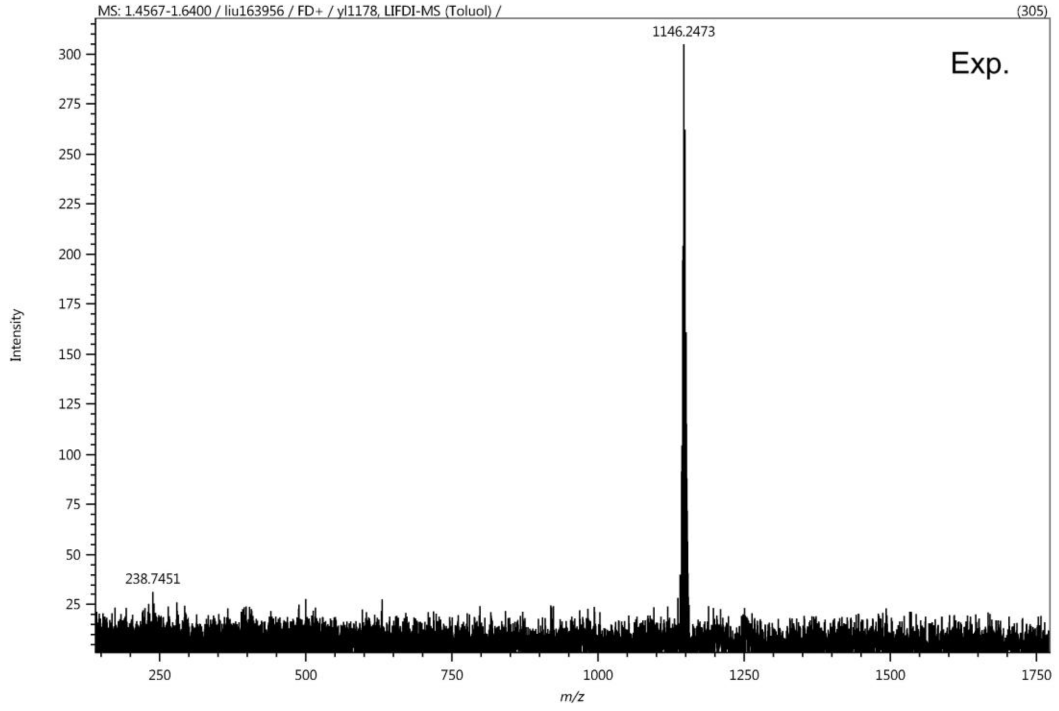

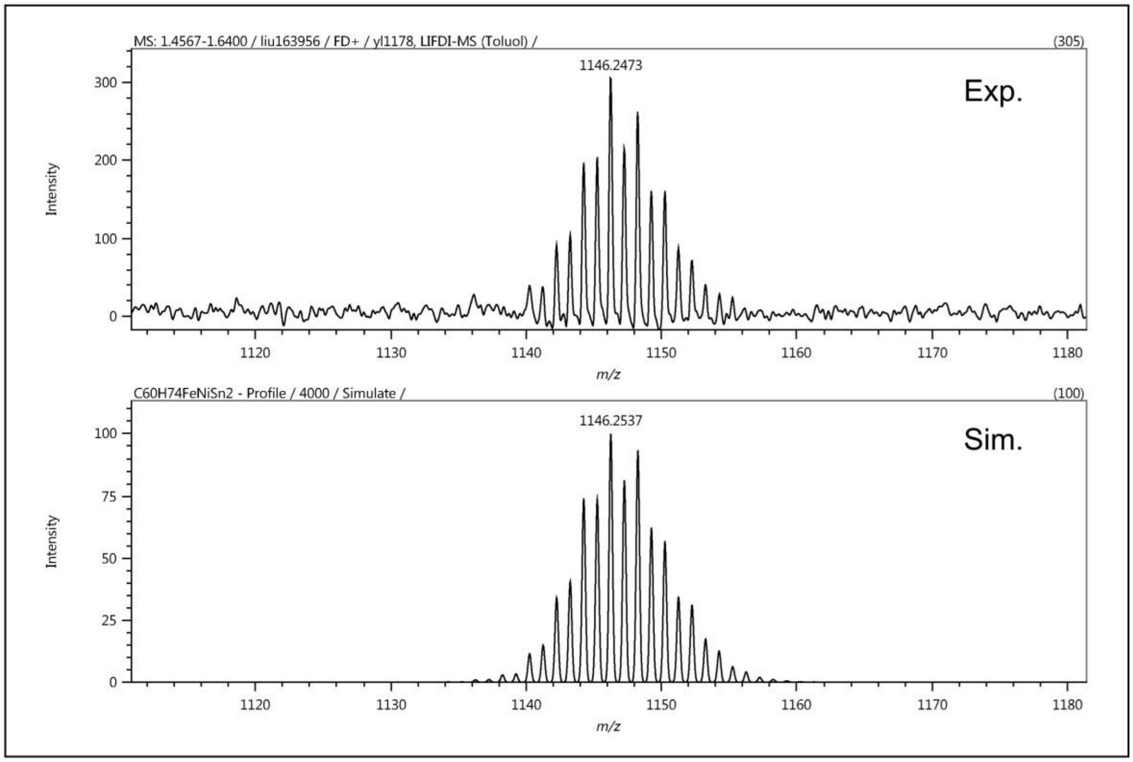

Figure S46. LIFDI–MS spectrum (toluene) of **2** (top) and the isotope pattern and a simulation for the peak around m/z = 1146 $[M]^+$ (bottom).

IR Spectrum of 7

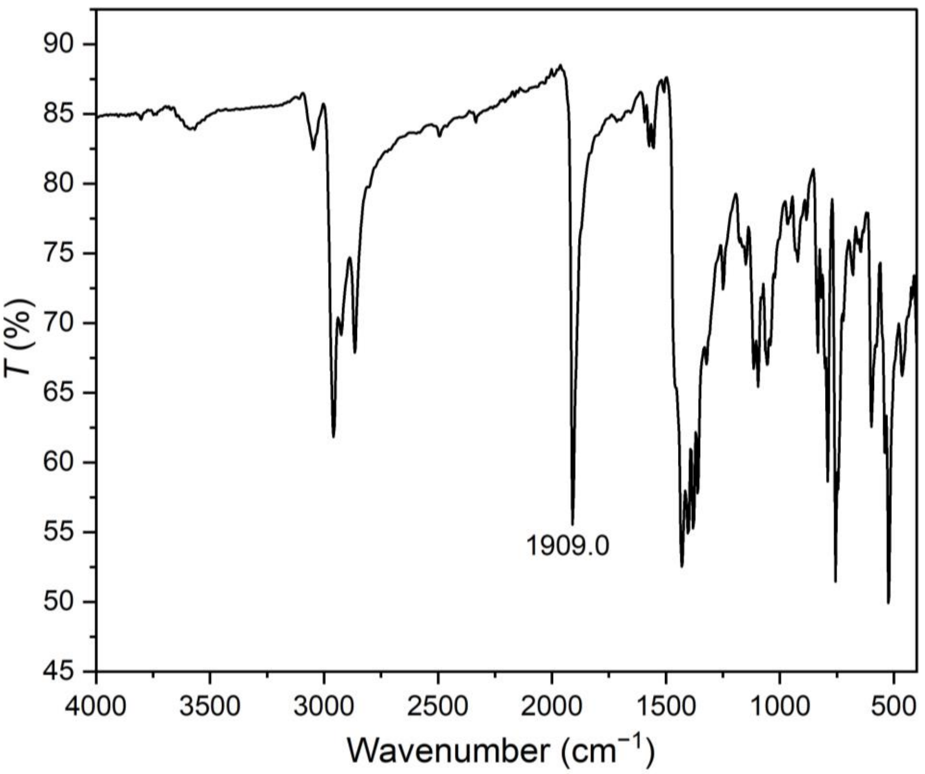

Figure S47. ATR–IR spectra (solid) of **7**.

ICP-OES Spectroscopy of 1 and 2

Inductively coupled plasma optical emission spectroscopy (ICP-OES) was recorded with the spectrometer from SPECTRO. Calibration curves created by measuring a series of standard samples were used to determine the concentrations of the elements of interest. Samples were prepared as 1.0 mM solutions in 65% HNO_3 by the following procedure. Table S4 summarizes the results from the ICP-OES measurements for complexes **1** and **2**.

65% HNO_3 (10 mL) was added to a glass vial containing fine powder of **1** or **2** (0.01 mmol, 1.0 mM) at room temperature. The mixture was subjected to sonication for 10 – 20 min until no black powder of **1** or **2** could be observed at the bottom of the vial. During this time grey precipitate appeared in the solution. The resulting yellowish solution was used for the ICP-OEC analysis after filtration.

Table S4. The concentrations of Fe, Ni and Sn detected in the ICP-OES analyses.

Sample **1**			Sample **2**		
No.	Conc. of Fe and Sn (μM)		Conc. of Fe, Ni and Sn (μM)		
	Fe	Sn	Fe	Ni	Sn
1	854.9	291.8	836.3	859.9	56.19
2	836.8	281.3	831.0	833.3	53.87
3	828.7	275.1	810.1	821.4	52.11
Average	840.2	282.7	825.8	838.2	54.06

Note: The concentrations of Fe and Ni detected for both **1** and **2** are close to the expected value (1000 μM). The ratio of Fe to Ni in **2** (1 : 1) is consistent with the metal composition in the solid-state structure. The detected concentrations of Sn in **1** and **2** deviate largely from the expected value. This may be due to the formation of insoluble organotin species during the sample preparation, as indicated by the appearance of grey precipitate.

Zero-field ^{57}Fe Mössbauer Measurements

Mössbauer spectra were recorded with a ^{57}Co source in a Rh matrix using an alternating constant acceleration *Wissel* Mössbauer spectrometer operated in transmission mode and equipped with a *Janis* closed-cycle helium cryostat (Göttingen University, Germany). Isomer shifts are given in mm s^{-1} relative to iron metal at ambient temperature. Simulation of the experimental data was performed with the *mf2.SL* program using *Lorentzian* line doublets: E. Bill, Max-Planck Institute for Chemical Energy Conversion, Mülheim/Ruhr, Germany.

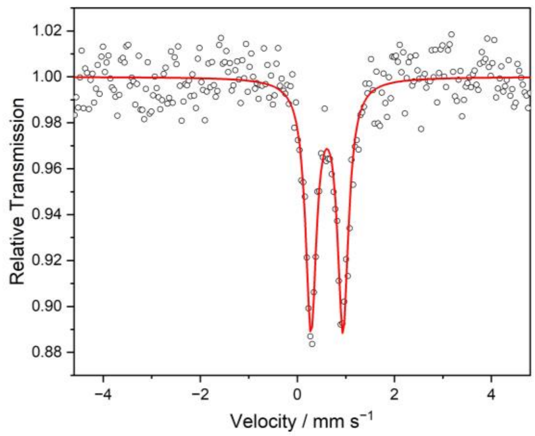

Figure S48. Zero-field ^{57}Fe Mössbauer spectrum for solid sample of complexes **1** (δ = 0.61 mm s^{-1}, $|\Delta E_Q|$ = 0.66 mm s^{-1}) at 80 K.

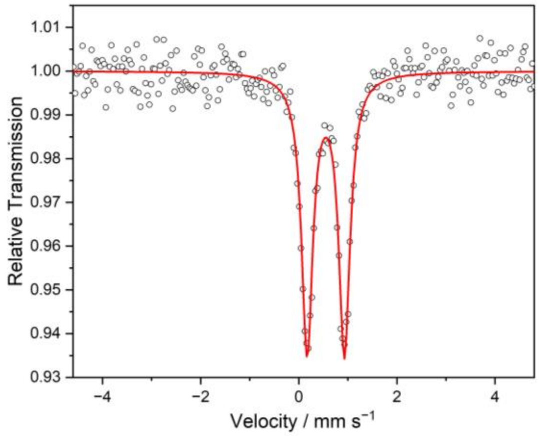

Figure S49. Zero-field ^{57}Fe Mössbauer spectrum for solid sample of complexes **2** **(**δ = 0.55 mm s^{-1}, $|\Delta E_Q|$ = 0.76 mm s^{-1}**)** at 80 K.

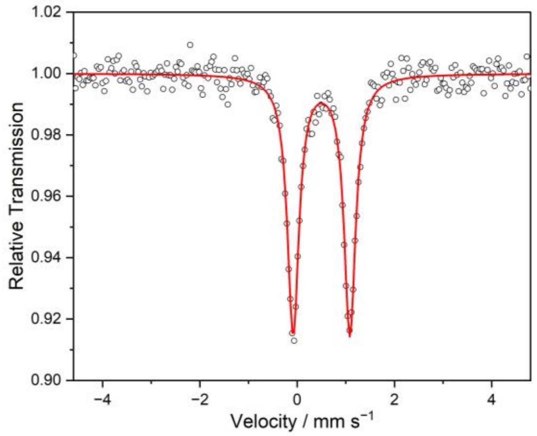

Figure S50. Zero-field ^{57}Fe Mössbauer spectrum for solid sample of complexes **5** **(**δ = 0.50 mm s^{-1}, $|\Delta E_Q|$ = 1.17 mm s^{-1}**)** at 80 K.

Crystallographic Data

X-Ray Structure Determination. Crystal data and details of the data collections are given in Tables S5 and S6, molecular structures are shown in Figures S51–S58. Single-crystal X-ray diffraction data were recorded on Rigaku XtaLAB Synergy DW (HyPix-Arc 150) diffractometers with Cu-K$_\alpha$ radiation (λ = 1.54184 Å; for **1**, **3** – **7**) and Mo-K$_\alpha$ radiation (λ = 0.71073 Å; for **2**). The solid-state structures for **8** were measured at the European Synchrotron Radiation Facility, beamline BM20 (λ = 0.61996 Å).[5] Crystals were selected under mineral oil, mounted on micro mount loops and quench-cooled using an open flow N_2 cooling device. The structures were solved with SHELXT[6] solution program using dual methods and by using Olex2 as the graphical interface.[7] The models were refined with ShelXL[8] (for **1**, **3**-**8**) or olex2.refine[9] (for **2**) using full matrix least squares minimization on F^2. The hydrogen atoms were located in idealized positions and refined isotropically with a riding model. Either semi-empirical multi-scan absorption corrections[10] or analytical ones[11] were applied to the data by using CrysAlisPro (171.43.58a) program.[12]

In **1**, only half of the formula unit is present in the asymmetric unit, with the other half consisting of symmetry equivalent atoms. The Sn1, Sn2 and Fe1 atoms are disordered with respect to a crystallographic inversion center and each atom was refined at half-occupancy at the two disordered sites. SADI (Fe−Sn and Sn−C) restraints were applied.

In **2**, only half of the formula unit is present in the asymmetric unit, with the other half consisting of symmetry equivalent atoms. The Sn1, Sn2, Fe1 and Ni1 atoms are disordered with respect to a crystallographic inversion center and each atom was refined at half-occupancy at the two disordered sites. SADI (Fe−Sn, Ni−Sn and Sn−C) restraints and EADP constraints were applied.

In **3**, two toluene are disordered. One toluene was found to be disordered around two positions (occupancy factors: 0.57 / 0.43). It was refined with SADI (C−C), SAME, SIMU and restraints and EADP constraint. The other toluene was found disordered around a special position and was refined at half-occupancy with SADI (C−C), DFIX (C^{Me}−C^{Ar} 1.51 Å, C^{Ar}−C^{Ar} 1.39 Å), DANG (C^{Me}−C^{Ar} 2.52 Å, C^{Ar}−C^{Ar} 2.40 Å), FLAT, SIMU and RIGU restraints.

In **5**, two independent molecules with similar conformations, bond distances and angles are in one asymmetric unit.

In **6**, one ^{i}Pr group was found to be disordered over two positions (occupancy factors: 0.53 / 0.47). 2.25 *n*-pentane molecules per asymmetric unit are severely disordered. They were refined by using a solvent mask which was calculated by Olex2, and 381 electrons were found in a volume of 2364 Å^3 in one void per unit cell. This is consistent with the presence of 9 *n*-pentane

molecules which account for 378 electrons per void. Although these solvent atoms were not included in the refinement model they are included in the formula and in the calculation of the formula weight, density, μ, $F(000)$, etc. As **6** was crystallized from *n*-pentane this serves as evidence for the presence of *n*-pentane in these positions, though they proved to be severely disordered.

In **7**, one Et_2O was found disordered around a special position and was refined at half-occupancy. It was refined with SADI, DANG (O−C 2.37 Å), DFIX (C−C 1.48 Å, C−O 1.42 Å), SIMU and RIGU restraints.

In **8**, two independent molecules with similar conformations, bond distances and angles are in one asymmetric unit. 0.5 *n*-pentane molecules per asymmetric unit were found severely disordered. They were refined using the solvent mask which was calculated by Olex2, and 75 electrons were found in a volume of 272 Å^3 in one void per unit cell. This is consistent with the presence of approximately 2 *n*-pentane molecules per void which account for 84 electrons. Although these solvent atoms were not included in the refinement model they are included in the formula and in the calculation of the formula weight, density, μ, $F(000)$, etc. As **8** was crystallized from *n*-pentane this serves as evidence for the presence of *n*-pentane in these positions, though they proved to be severely disordered.

Table S5. Crystallographic data and structure refinement details for compounds **1 – 4**.

**Compound**	**1**	**2**	**3**	**4**
CCDC	2406557	2410942	2406598	2406570
Empirical formula	$C_{60}H_{74}FeSn_2$	$C_{72}H_{86}FeNiSn_2$	$C_{77.5}H_{94}AlBr_3FeSn_2$	$C_{61}H_{77}FeISn_2$
Formula weight	1088.42	1303.44	1585.46	1230.35
Temperature/K	123.00(10)	100.00(10)	123.00(10)	123.00(10)
Crystal system	orthorhombic	triclinic	monoclinic	orthorhombic
Space group	*P*ccn	*P*-1	$P2_1/n$	*P*bca
a/Å	15.54750(10)	10.4878(3)	10.52340(10)	17.07659(8)
b/Å	20.5763(2)	13.1780(3)	19.38400(10)	18.77190(8)
c/Å	16.55260(10)	13.3517(3)	34.5194(2)	34.86238(18)
α/°	90	65.579(2)	90	90
β/°	90	77.441(2)	94.1140(10)	90
γ/°	90	69.499(2)	90	90
Volume/Å^3	5295.34(7)	1568.08(7)	7023.32(9)	11175.49(9)
Z	4	1	4	8
ρ_{calc} g/cm^3	1.365	1.380	1.499	1.463
μ/mm^{-1}	9.868	1.350	9.673	13.704
F(000)	2240	671.8	3212	4976
Crystal size/mm^3	0.17 × 0.147 × 0.091	0.36 × 0.29 × 0.14	0.169 × 0.149 × 0.026	0.157 × 0.118 × 0.052
Radiation	Cu Kα (λ = 1.54184 Å)	Mo Kα (λ = 0.71073 Å)	Cu Kα (λ = 1.54184 Å)	Cu Kα (λ = 1.54184 Å)
2Θ range for data collection/°	8.594 to 150.706	2.08 to 43.53	5.134 to 150.684	5.07 to 150.446
Index ranges	−19 ≤ h ≤ 19, −25 ≤ k ≤ 25, −20 ≤ l ≤ 14	−17 ≤ h ≤ 14, −25 ≤ k ≤ 25, −25 ≤ l ≤ 25	−12 ≤ h ≤ 12, −23 ≤ k ≤ 23, −43 ≤ l ≤ 42	−20 ≤ h ≤ 21, −22 ≤ k ≤ 23, −43 ≤ l ≤ 43
Reflections collected	29211	111014	80074	101547
Independent reflections	5380 [R_{int} = 0.0243, R_{sigma} = 0.0155]	20296 [R_{int} = 0.0226, R_{sigma} = 0.0146]	14275 [R_{int} = 0.0370, R_{sigma} = 0.0219]	11391 [R_{int} = 0.0290, R_{sigma} = 0.0154]
Data/restraints/parameters	5380/2/306	20296/6/357	14275/325/865	11391/0/603
Goodness-of-fit on F^2	1.137	1.050	1.072	1.070
Final R indexes [I>=2σ (I)]	R1 = 0.0335, wR2 = 0.0821	R1 = 0.0198 , wR2 = 0.0516	R1 = 0.0354, wR2 = 0.0947	R1 = 0.0299, wR2 = 0.0783
Final R indexes [all data]	R1 = 0.0358, wR2 = 0.0830	R1 = 0.0225, wR2 = 0.0525	R1 = 0.0393, wR2 = 0.0966	R1 = 0.0314, wR2 = 0.0791
Largest diff. peak/hole / e Å^{-3}	0.31/−0.43	0.75/−0.59	1.04/−1.05	2.65/−0.89

Table S6. Crystallographic data and structure refinement details for compounds **5 – 8**.

**Compound**	**5**	**6**	**7**	**8**
CCDC	2406571	2406573	2406574	2407837
Empirical formula	$C_{63}H_{83}FePSn_2$	$C_{133.25}H_{175}Fe_2S_4Sn_4$	$C_{128}H_{158}Fe_2O_9Sn_4$	$C_{65}H_{86}FeP_4Sn_2$
Formula weight	1164.49	2491.42	2426.99	1284.44
Temperature/K	123.00(10)	123.00(10)	123.00(10)	100.15
Crystal system	monoclinic	monoclinic	triclinic	triclinic
Space group	$P2_1$/c	$P2_1$/c	*P*-1	*P*-1
a/Å	19.14402(6)	18.66358(6)	11.7790(2)	12.48710(10)
b/Å	25.90377(8)	27.75355(7)	13.6942(2)	18.7238(2)
c/Å	23.61732(7)	25.54582(8)	19.4337(4)	28.4843(2)
α/°	90	90	88.181(2)	90.8290(10)
β/°	91.4294(3)	110.2089(4)	74.209(2)	102.5690(10)
γ/°	90	90	73.4160(10)	101.5510(10)
Volume/$Å^3$	11708.24(6)	12417.65(7)	2887.37(9)	6356.80(10)
Z	8	4	1	4
ρ_{calc}g/cm^3	1.321	1.333	1.396	1.342
μ/mm^{-1}	9.21	9.096	9.169	0.792
F(000)	4816	5162	1250	2648
Crystal size/mm^3	0.15 × 0.11 × 0.08	0.279 × 0.268 × 0.268	0.19 × 0.15 × 0.05	0.6 × 0.4 × 0.3
Radiation	Cu Kα (λ = 1.54184 Å)	Cu Kα (λ = 1.54184 Å)	Cu Kα (λ = 1.54184 Å)	synchrotron (λ = 0.61996 Å)
2Θ range for data collection/°	4.618 to 140.15	4.87 to 150.388	4.732 to 149.336	2.966 to 62.212
Index ranges	−23 ≤ h ≤ 22, −31 ≤ k ≤ 31, −28 ≤ l ≤ 28	−22 ≤ h ≤ 23, −34 ≤ k ≤ 34, −31 ≤ l ≤ 31	−13 ≤ h ≤ 14, −16 ≤ k ≤ 16, −23 ≤ l ≤ 23	−20 ≤ h ≤ 20, −31 ≤ k ≤ 31, −47 ≤ l ≤ 47
Reflections collected	143452	158834	61684	211759
Independent reflections	23868 [R_{int} = 0.0302, R_{sigma} = 0.0184]	25275 [R_{int} = 0.0280, R_{sigma} = 0.0157]	11473 [R_{int} = 0.0226, R_{sigma} = 0.0153]	59489 [R_{int} = 0.0603, R_{sigma} = 0.0600]
Data/restraints/parameters	22231/0/1245	25275/0/1251	11473/54/685	59489/0/1286
Goodness-of-fit on F^2	1.100	1.035	1.086	1.032
Final R indexes [I>=2σ (I)]	R1 = 0.0274, wR2 = 0.0683	R1 = 0.0249, wR2 = 0.0640	R1 = 0.0281, wR2 = 0.0722	R1 = 0.0447, wR2 = 0.1013
Final R indexes [all data]	R1 = 0.0294, wR2 = 0.0691	R1 = 0.0262, wR2 = 0.0646	R1 = 0.0296, wR2 = 0.0731	R1 = 0.0686, wR2 = 0.1086
Largest diff. peak/hole / e $Å^{-3}$	2.34/−0.76	1.74/−0.85	0.73/−1.26	1.85/−1.06

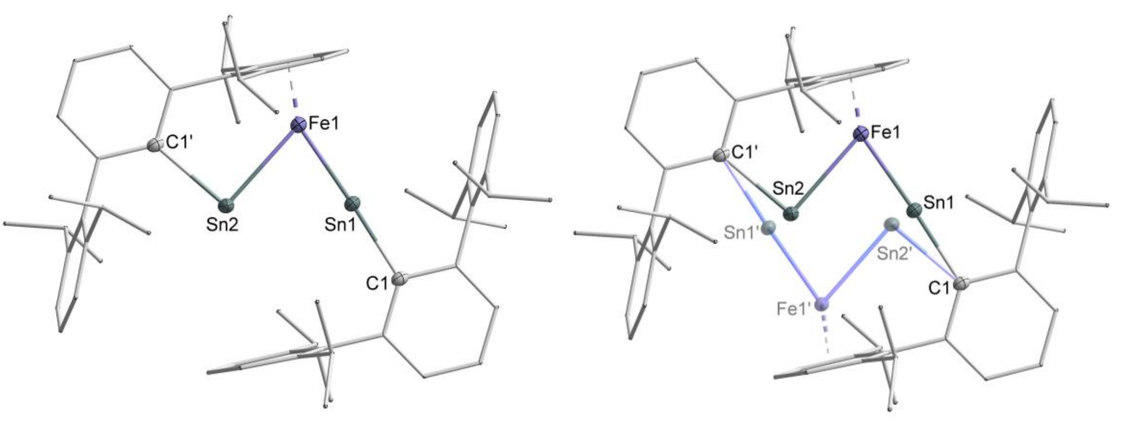

Figure S51. Plot (50% probability thermal ellipsoids, hydrogen atoms omitted) of the solid-state molecular structure of **1** without (left) and with (right) the disordered Fe and Sn atoms. Selected bond lengths [Å] and angles [°]: Fe1–Sn1 2.2954(7), Fe1–Sn2 2.6207(7), Sn1–C1 2.1345(19), Sn2–C1' 2.2428(19), Sn1···Sn2 3.0596(6); Fe1–Sn1–C1 176.60(6), Fe1–Sn2–C1' 91.10(5), Sn1–Fe1–Sn2 76.66(2), C1–Sn1···Sn2 121.14(5), C1'–Sn2···Sn1 137.89(5).

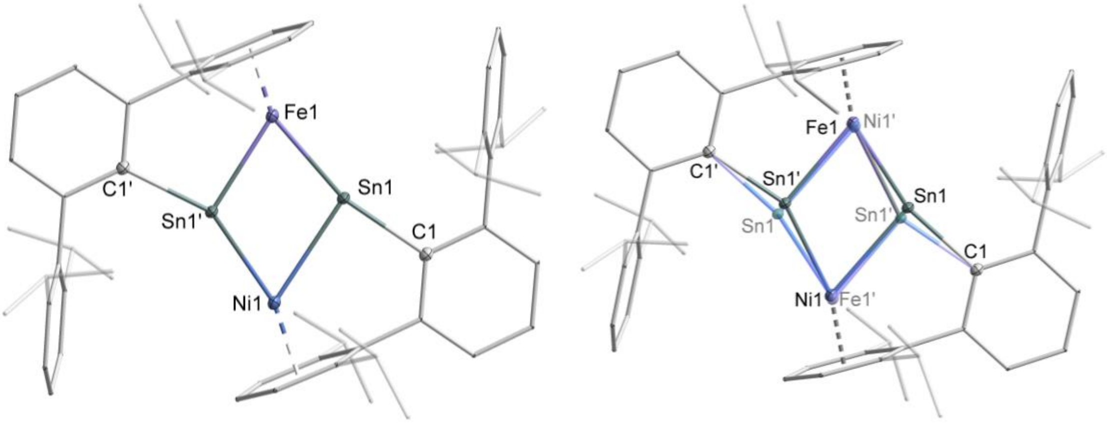

Figure S52. Plot (50% probability thermal ellipsoids, hydrogen atoms and solvent molecule (C_6H_6) omitted) of the solid-state molecular structure of **2** without (left) and with (right) the disordered Fe, Ni and Sn atoms. Selected bond lengths [Å] and angles [°]: Fe1–Sn1 2.3775(22), Fe1–Sn1' 2.4431(23), Sn1–C1 2.1875(5), Sn1'–C1' 2.4431(24), Sn1···Sn1' 2.8860(3), Ni1–Sn1 2.7018(24), Ni1–Sn1' 2.4328(22); Sn1–Fe1–Sn1' 74.54(7), Fe1–Sn1–C1 165.29(7), Fe1–Sn1–Ni 105.71(8), Fe1–Sn1'–C1' 95.98(7), Fe1–Sn1'–Ni 112.47(8), Ni1–Sn1'–C1' 151.29(7), Ni1–Sn1–C1 89.00(6).

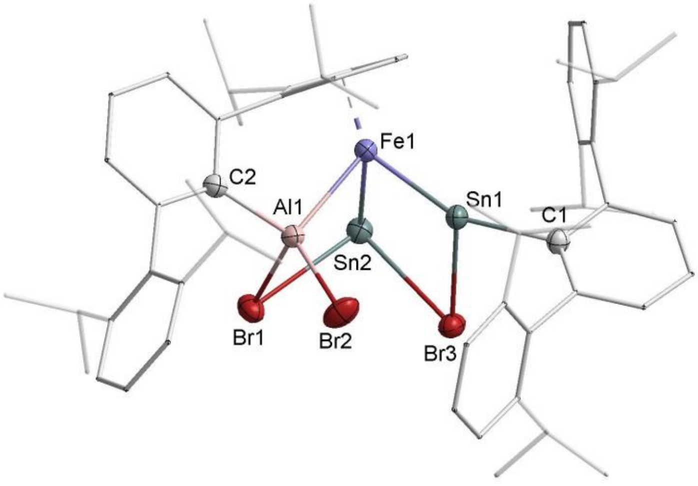

Figure S53. Plot (50% probability thermal ellipsoids, hydrogen atoms and solvent molecules (toluene) omitted) of the solid-state molecular structure of **3**. Selected bond lengths [Å] and angles [°]: Fe1–Sn1 2.3768(9), Fe1–Sn2 2.5249(6), Fe1–Al1 2.4605(12), Sn1–C1 2.193(3), Sn1–Br3 2.6875(5), Sn2–Br3 2.8765(8), Sn2–Br2 2.9139(8), Sn1···Sn2 3.2654(5), Al1–Br1 2.4579(11), Al1–Br2 2.3331(11), Al1–C2 1.987(4); Fe1–Sn1–C1 158.02(9), Fe1–Sn1–Br3 105.95(2), C1–Sn1–Br3 90.78(9), Fe1–Sn2–Br3 96.85(2), Fe1–Sn2–Br1 95.93(2), Br1–Sn2–Br3 89.76(1), Sn2–Fe1–Al1 78.75(3), Sn1–Fe1–Sn2 83.49(2), Fe1–Al1–C2 103.81(10).

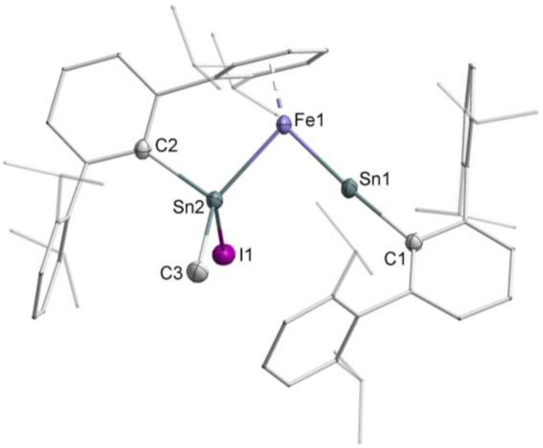

Figure S54. Plot (50% probability thermal ellipsoids, hydrogen atoms omitted) of the solid-state molecular structure of **4**. Selected bond lengths [Å] and angles [°]: Fe1–Sn1 2.2960(4), Fe1–Sn2 2.5188(5), Sn1–C1 2.143(3), Sn2–C2 2.183(3), Sn2–C3 2.178(3), Sn2–I1 2.8086(3), Sn1···Sn2 3.3804(5); Fe1–Sn1–C1 175.45(8), Fe1–Sn2–C2 97.45(8), Sn1–Fe1–Sn2 89.07(2), Fe1–Sn2–I1 110.00(1), Fe1–Sn2–C3 134.01(9), C3–Sn2–I1 97.05(10).

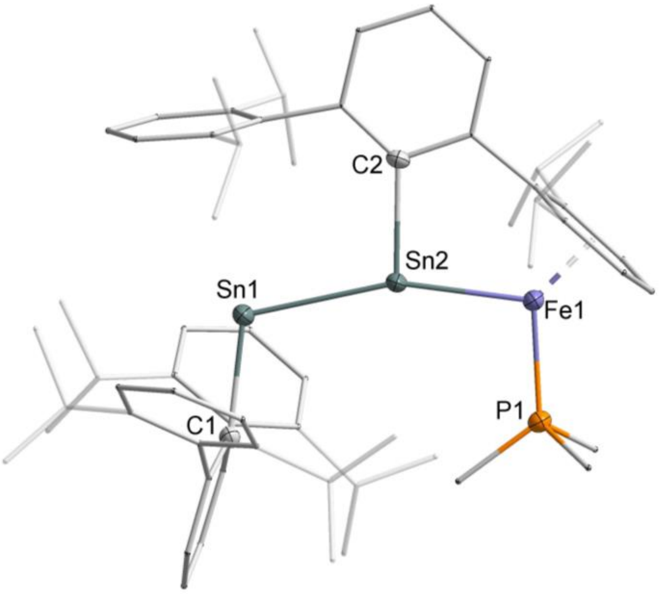

Figure S55. Plot (50% probability thermal ellipsoids, hydrogen atoms omitted) of the solid-state molecular structure of **5**. Selected bond lengths [Å] and angles [°]: Fe1–Sn2 2.4864(7), Fe1–P1 2.2053(7), Sn1–Sn2 2.8614(7), Sn2–C2 2.2385(24), Sn1–C1 2.2182(24); Sn2–Fe1–P1 99.78(2), Fe1–Sn2–C2 96.59(6), Fe1–Sn2–Sn1 160.123(11), C2–Sn2–Sn1 102.42(6), Sn2–Sn1–C1 105.82(6).

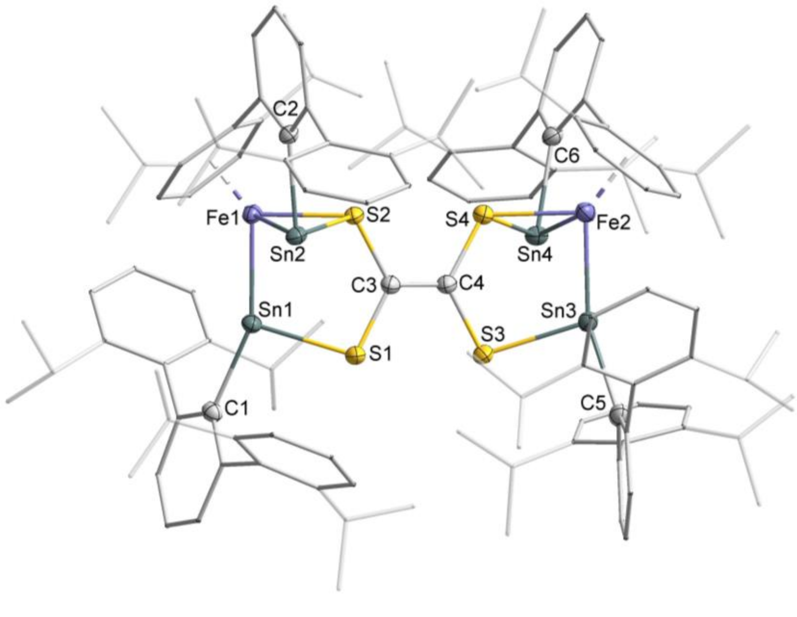

Figure S56. Plot (50% probability thermal ellipsoids, hydrogen atoms and solvent molecules (*n*-pentane) omitted) of the solid-state molecular structure of **6**. Selected bond lengths [Å] and angles [°]: C3–C4 1.356(3), C3–S1 1.765(2), C3–S2 1.784(2), C4–S3 1.762(2), C4–S4 1.786(2), Fe1–Sn1 2.4136(8), Fe1–S2 2.2781(6), Fe1–Sn2 2.7925(8), Sn2–S2 2.5556(7), Sn2–C2 2.2537(22), Sn1···Sn2 3.5029(8), Sn1–C1 2.1850(24), Sn1–S1 2.4267(6), Fe2–Sn3 2.4062(8), Fe2–S4 2.2786(6), Fe2–Sn4 2.8246(8), Sn3–S3 2.4336(7), Sn3–C5 2.1776(21), Sn3···Sn4 2.4295(8), Sn4–C6 2.2574(24), Sn4–S4 2.5655(7); Fe1–Sn1–C1 154.92(5), Fe1–Sn1–S1 105.62(6), C1–Sn1–S1 99.12(5), Fe1–Sn2–C2 86.14(5), Fe1–Sn2–S2 50.182(12), C2–Sn2–S2 88.95(5), Fe1–S2–Sn2 70.32(2), Sn2–Fe1–S2 59.50(1), Sn2–Fe1–Sn1 84.24(1), S2–Fe1–Sn1 90.24(2), S1–C3–S2 120.32(11), S3–C4–S4 120.15(11), Fe2–Sn3–C5 162.03(5), Fe2–Sn3–S3 105.79(1), C5–Sn3–S3 99.70(5), Fe2–Sn4–C6 86.29(5), Fe2–Sn4–S4 49.73(1), C6–Sn4–S4 93.54(5), Fe2–S4–Sn4 71.05(2), Sn4–Fe2–S4 59.22(1), Sn4–Fe2–Sn3 81.51(1), S4–Fe2–Sn3 90.14(2).

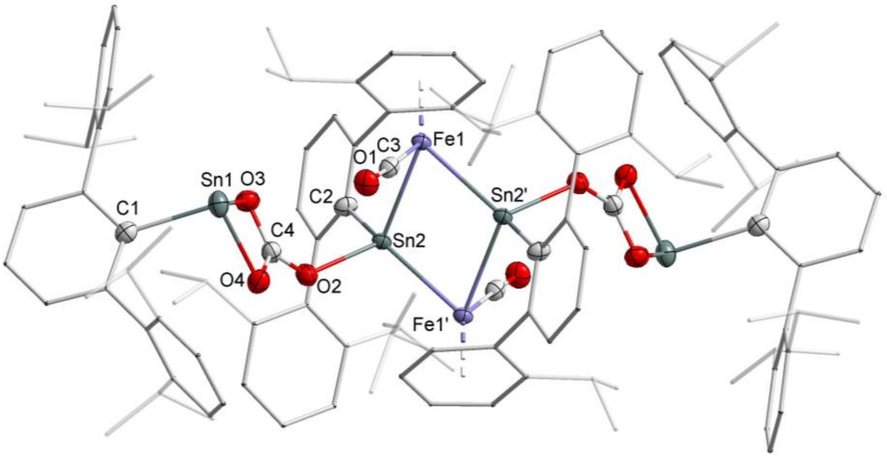

Figure S57. Plot (50% probability thermal ellipsoids, hydrogen atoms and solvent molecules (Et_2O) omitted) of the solid-state molecular structure of **7**. Selected bond lengths [Å] and angles [°]: Fe1–Sn2' 2.5421(5), Fe1–Sn2 2.5874(6), Fe1–C3 1.750(3), C3–O1 1.156(3), Sn2···Sn2' 3.1228(6), Sn2–C2 2.166(2), Sn2–O2 2.102(2), C4–O2 1.288(3), C4–O3 1.283(3), C4–O4 1.288(3), Sn1–O3 2.241(2), Sn1–O4 2.185(2), Sn1–C1 2.214(3); Sn2–Fe1–Sn2' 75.00(1), Fe1–Sn2–C2 96.50(7), Fe1–Sn2–Fe1' 105.00(1), Fe1–Sn2–O2 115.53(5), O2–Sn2–C2 102.73(9), Fe1'–Sn2–C2 132.50(7), Fe1–C3–O1 177.3(2), Sn2–O2–C4 132.1(2), O2–C4–O3 123.4(2), O2–C4–O4 119.4(2), O3–C4–O4 117.2(2), O3–Sn1–O4 59.41(7), O3–Sn1–C1 99.40(8), O4–Sn1–C1 96.07(9).

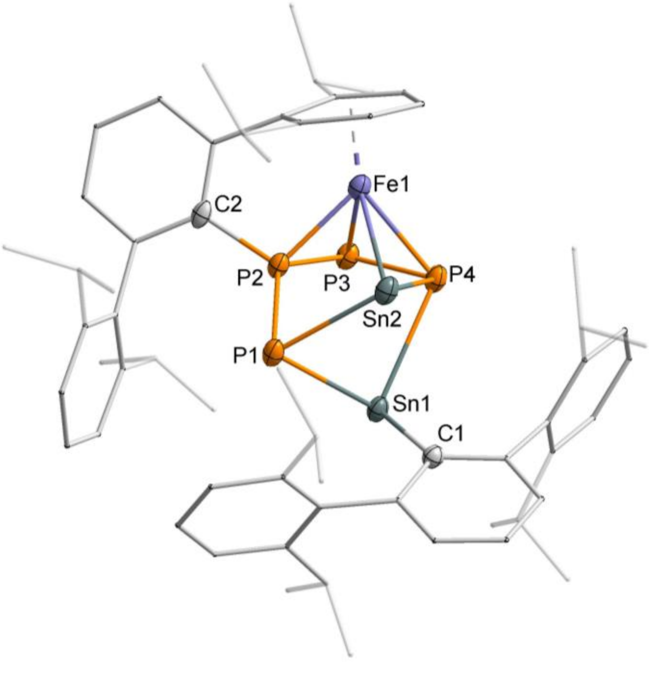

Figure S58. Plot (50% probability thermal ellipsoids, hydrogen atoms and solvent molecules (*n*-pentane) omitted) of the solid-state molecular structure of **8**. Selected bond lengths [Å] and angles [°]: Fe1–Sn2 2.7892(7), Fe1–P2 2.2016(5), Fe1–P3 2.3333(7), Fe1–P4 2.4154(8), C2–P2 1.8329(18), Sn2–P1 2.6272(6), Sn2–P4 2.7190(7), Sn1–P1 2.6657(5), Sn1–P4 2.8070(6), Sn1···Sn2 3.1262(2), C1–Sn1 2.2763(19), P1–P2 2.1313(8), P2–P3 2.1666(8), P3–P4 2.1523(8); Fe1–Sn2–P1 88.65(1), Fe1–Sn2–P4 52.00(1), P1–Sn2–P4 93.43(2), P1–Sn1–C1 97.75(5), P4–Sn1–C1 107.24(5).

Proposed Mechanism for the Formation of 3, 6 and 7

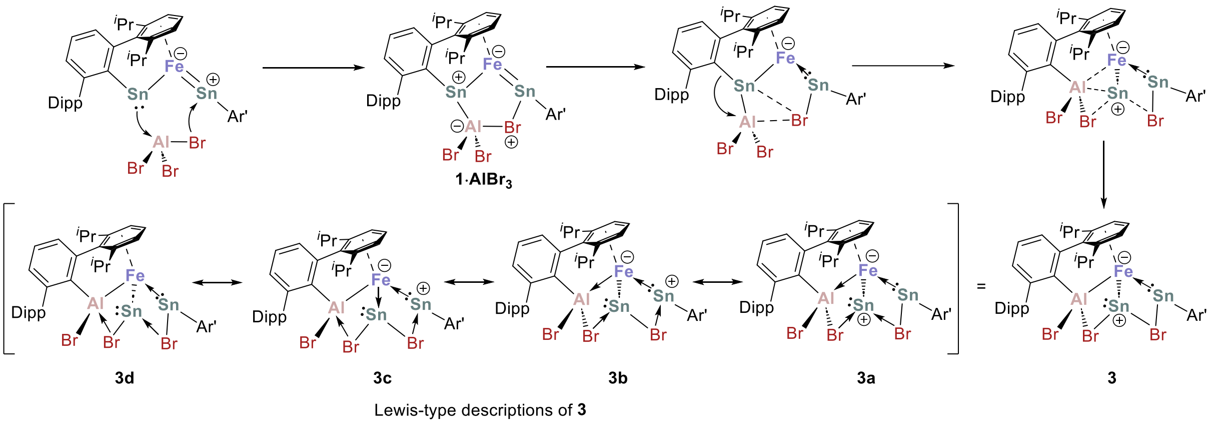

Scheme S1. Proposed mechanism for the formation of **3** through the reaction of **1** with $AlBr_3$.

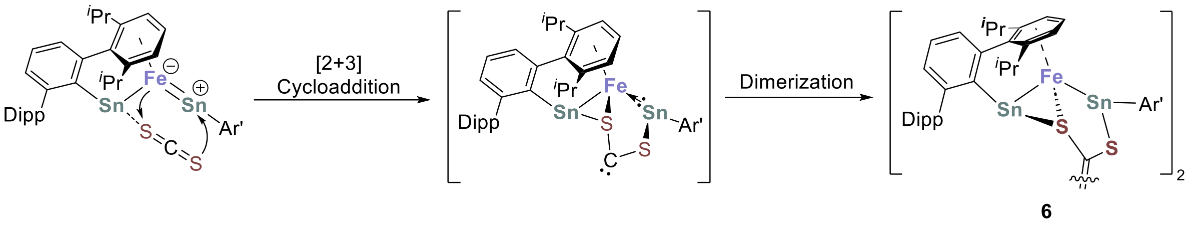

Scheme S2. Proposed mechanism for the formation of **6** through the reaction of **1** with CS_2.

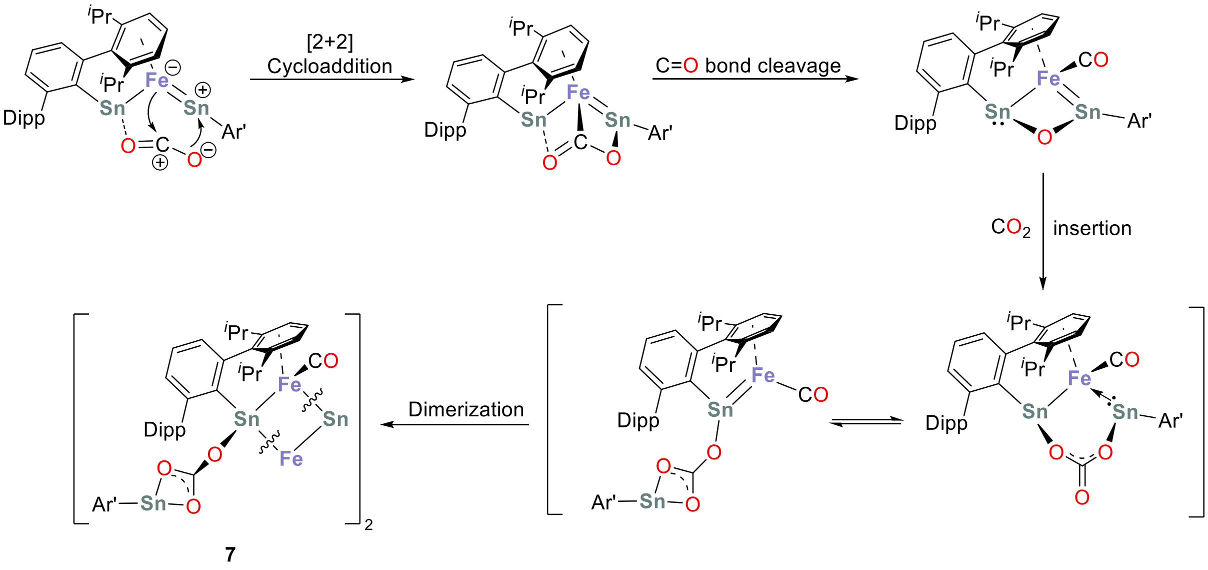

Scheme S3. Proposed mechanism for the formation of **7** through the reaction of **1** with CO_2.

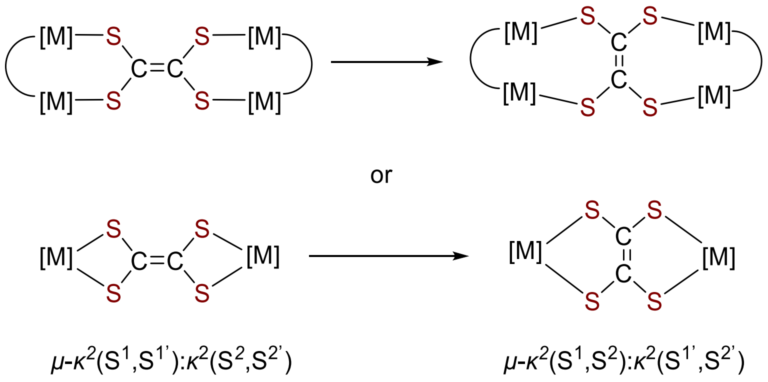

Figure S59. Coordination modes of the ethene-1,1,2,2-tetrathiolate ligand $[C_2S_4]^{4-}$.

DFT Calculations

Computational Details. All calculations were performed with the ORCA quantum chemical program package (version 5.0.4).[13,14] Geometry optimizations (OPT) were performed with the BP86 functional[14a,b] and the def2-TZVP basis set[15] in combination with the auxiliary basis set def2/J.[16] Effective core potentials (ECP) were used for Sn and I atoms.[17] The RI approximations was used to accelerate the calculations.[18] The noncovalent interactions were considered via atom-pairwise dispersion corrections with Becke-Johnson (D3BJ) damping.[19] Coordinates from X-ray structural analyses were used as starting coordinates and the optimized coordinates of **1–5** and **8** are given in Tables S20. Numerical frequency calculations at the same level of theory were performed to identify the number of imaginary frequencies (zero for local minimum) and provide the energy difference between Gibbs free energy and electronic energy (G−E(el)). Optimized structures were visualized by the IboView[20] or Chemcraft[21] program. Single point energy calculations presented in the manuscript were carried out with the B3LYP functional[14a-e] using the same basis set combination and using the RIJCOSX approximations.[22] The single point energy calculation for **1** was also carried out with ωB97X-D3 functional[14f] using the same basis set combination and using the RIJCOSX approximations, which yields similar molecular orbitals (MOs) to those calculated at the B3LYP/def2-TZVP level of theory, and selected MOs are presented in Figure S63. Time-dependent density functional theory (TD-DFT) calculations (80 roots) were carried out at the B3LYP level of theory and the CPCM method was applied to include solvent effects in the calculations.[23] The obtained excitation energies and simulated UV-vis spectrum are summarized in Figure S61. The Intrinsic bond orbital (IBO)[20] was calculated using ORCA program at the B3LYP level of theory. Natural bond orbital (NBO) calculations were carried out using NBO 6.0 program.[24] Energy decomposition analyses coupled with natural orbitals for chemical valence (EDA-NOCV),[25a,b] electron localization function (ELF),[25c] independent gradient model based on Hirshfeld partition (IGMH)[25d] analysis, intrinsic bond strength index (IBSI)[25e] and delocalization index (DI)[25f] values were carried out by using Multiwfn.[26]

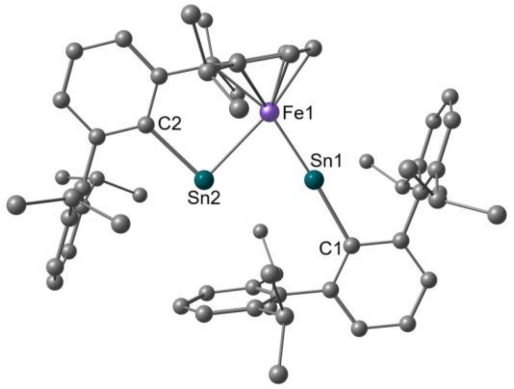

Figure S60. The optimized structure of **1** (BP86/def2-TZVP level). Hydrogen atoms are omitted for clarity.

Table S7. Comparison of the selected bond distances (Å) and bond angles (°) obtained from the crystal structure and the optimized geometries of **1**.

	**Exp.**	**Opt.**
Fe1–Sn1/Sn2	2.2954(7)/ 2.6207(7)	2.293/2.647
Sn1/Sn2–C_{ipso}	2.1345(19), 2.2428(19)	2.170/2.236
Sn1···Sn2	3.0596(6)	3.100
Fe1–Sn1/Sn2–C_{ipso}	176.60(6)/91.10(5)	175.80/89.73

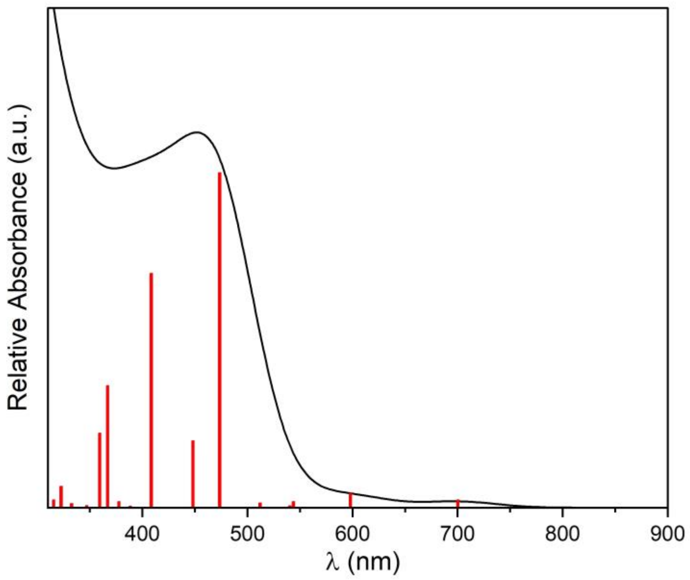

Figure S61. TD-DFT calculated UV-vis spectra for **1**. The calculated spectrum was convoluted using a Gaussian line shape function with a half-width of 85 nm and a shift of −70 nm was applied. The principal contributions involved in the electronic transitions at 410 nm, 470 nm, 590 nm and 690 nm are HOMO-2 → LUMO+2 (35%) and HOMO-1 → LUMO (14%), HOMO-1 → LUMO (68%), HOMO-1 → LUMO+1 (28%) and HOMO → LUMO+2 (66%) and HOMO → LUMO (95%), respectively.

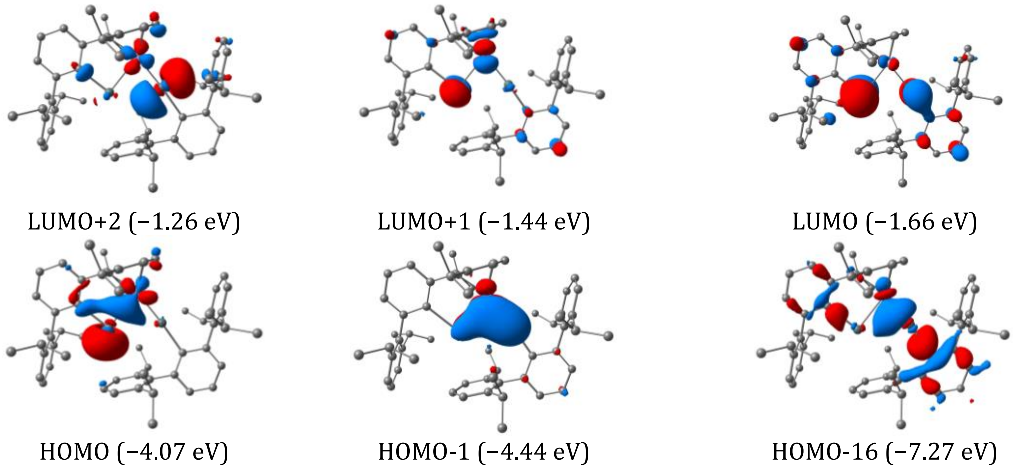

Figure S62. Selected molecular orbitals of **1** (Isovalue = 0.04; B3LYP/def2-TZVP level).

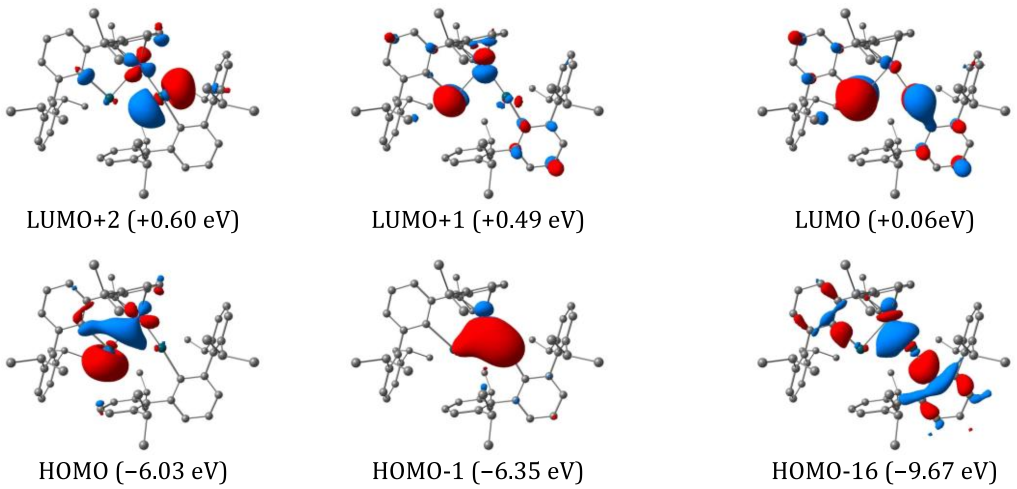

Figure S63. Selected molecular orbitals of **1** (Isovalue = 0.04; ωB97X-D3/def2-TZVP level).

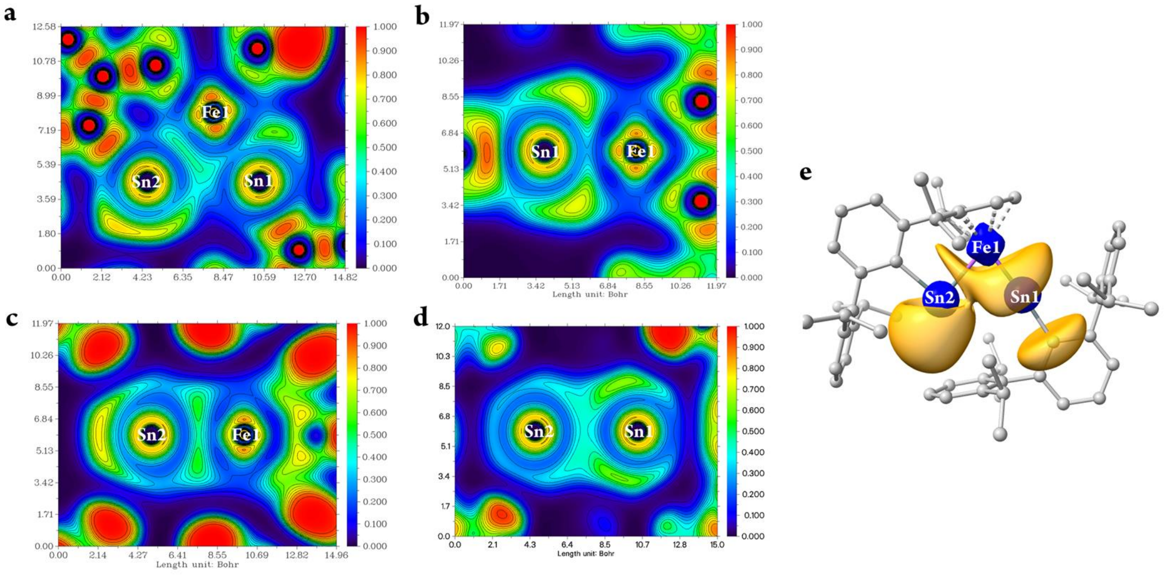

Figure S64. ELF plot of **1** in the Sn1–Fe1–Sn2 plane (a) and planes perpendicular to the Sn1–Fe1–Sn2 plane while going through the Fe1–Sn1 bond (b), Fe1–Sn2 bond (c) and Sn1···Sn2 axis (d), and 3D ELF plot of **1** (e; Only contributions from Fe and Sn were considered; Isovalue = 0.45).

Note: The presence of high electron density above and below the Fe1–Sn1 bond (yellow regions in view b) and relatively low electron density on the Fe1–Sn1 bond axis (view a), indicates an Fe1=Sn1 double bond.[25c] Similarly, the green regions above and below the Fe1–Sn2 bond (view c), along with the high electron density on the Fe1–Sn1 bond axis where a torus can be observed (view a), indicate an Fe1–Sn2 single bond with weak π-interaction. The cyan region between the two Sn atoms shows no torus, indicating a weak Sn1···Sn2 interaction (view d).

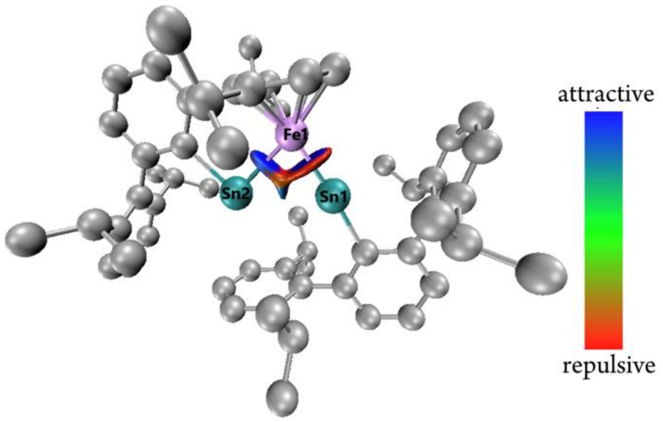

Figure S65. IGMH analysis of **1** (Isovalue = 0.03). Intrinsic bond strength index (IBSI) based on IGMH formulation was calculated for Fe1–Sn1 (0.39), Fe1–Sn2 (0.21) and Sn1···Sn2 (0.13).

Natural Population Analysis (NPA)

Table S8. Charges on selected atoms and fragments obtained from natural population analysis (NPA) of **1, 2, 4** and **5**.

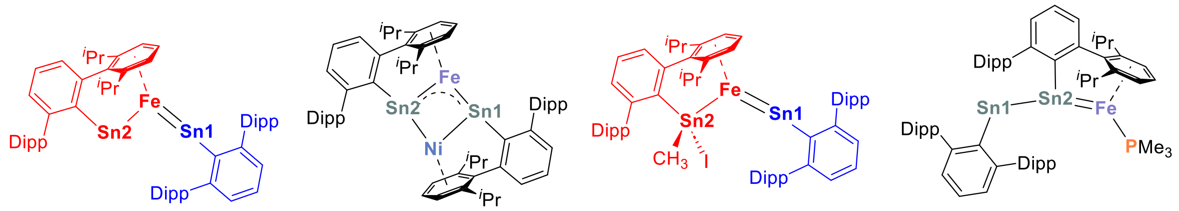

1 2 4 5

	NPA Charge/e			
Atom/Fragment	**1**	**2**	**4**	**5**
Fe	−0.65	−0.46	−0.61	−0.31
Sn1	+1.04	+0.91	+1.26	+0.94
Sn2	+0.92	+0.71	+1.36	+0.37
Fragment in red	−0.52		−0.79	
Fragment in blue	+0.52		+0.79	

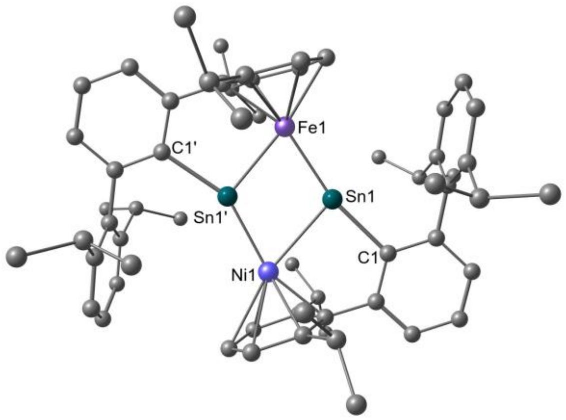

Figure S66. The optimized structure of **2** (BP86/def2-TZVP level). Hydrogen atoms are omitted for clarity.

Table S9. Comparison of the selected bond distances (Å) and bond angles (°) obtained from the crystal structure and the optimized geometries of **2**.

	**Exp.**	**Opt.**
Fe1–Sn1/Sn1’	2.3775(22)/2.4431(23)	2.374/2.477
Sn1/Sn1’–C_{ipso}	2.1874(5)/2.1592(5)	2.176/2.164
Sn1···Sn1’	2.8860(3)	2.892
Fe1–Sn1/Sn1’–C_{ipso}	165.29(7)/95.98(7)	167.66/95.19
Ni1–Sn1/Sn1’	2.702(2)/2.433(2)	2.693/2.390
Ni1–Sn1/Sn1’–C_{ipso}	151.29(7)/89.00(6)	152.48/86.81

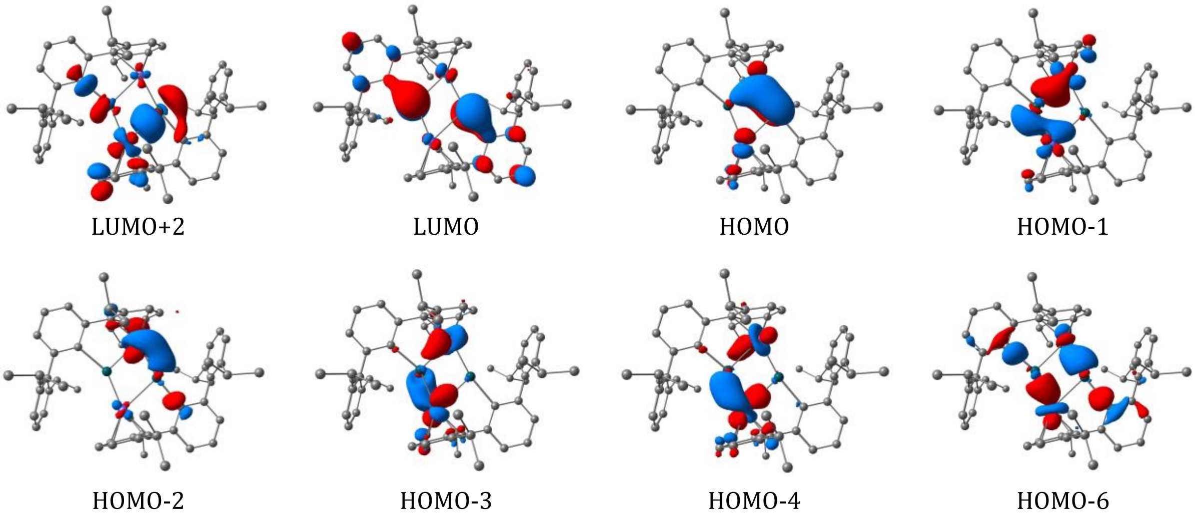

Figure S67. Selected molecular orbitals of **2** (Isovalue = 0.04).

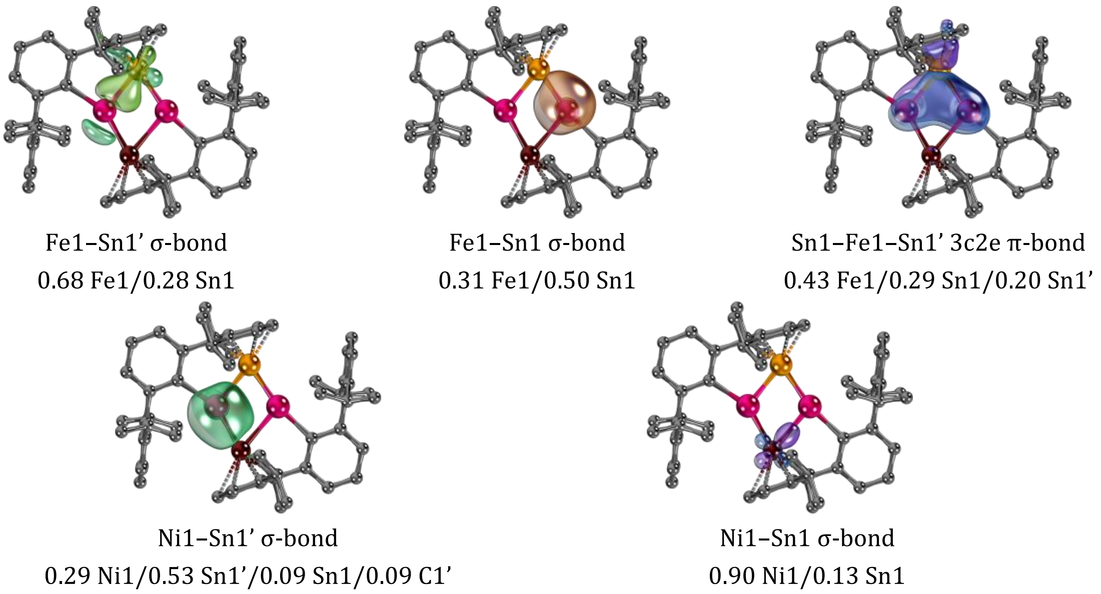

Figure S68. Selected IBOs of **2**. The bond type and percentage of the electron density on Fe, Ni and Sn are given.

Table S10. Mayer bond orders of selected bonds of **2**.

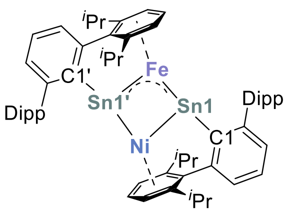	**Mayer Bond Order (MBO)**						
	Fe–Sn1	Fe–Sn1'	Ni –Sn1	Ni–Sn1'	Sn1–C1	Sn1'–C1'	Sn1···Sn1'
	1.26	1.22	0.62	0.90	0.83	0.85	0.53

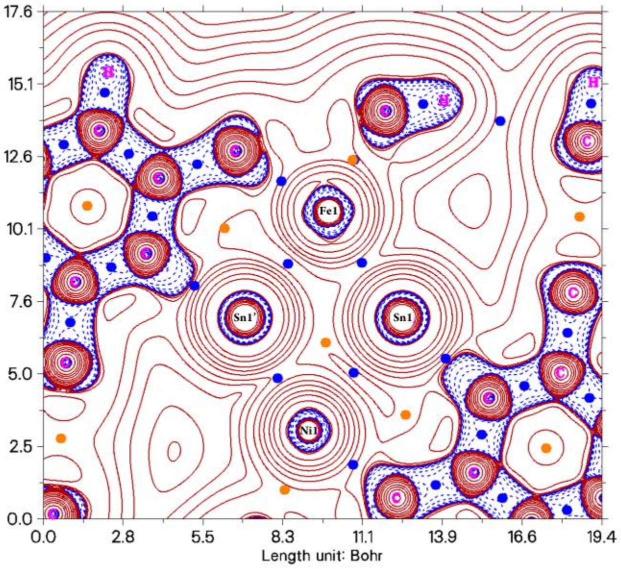

Figure S69. Plot of the Laplacian of the electron density on the $FeNiSn_2$ plane of **2** (BCP: blue dots, RCP: brown dots).

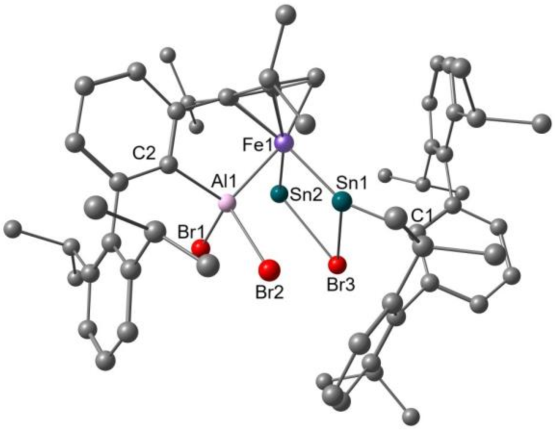

Figure S70. The optimized structure of **3** (BP86/def2-TZVP level). Hydrogen atoms are omitted for clarity.

Table S11. Comparison of the selected bond distances (Å) and bond angles (°) obtained from the crystal structure and the optimized geometries of **3**.

	**Exp.**	**Opt.**
Fe1–Sn1/Sn2	2.3768(9)/2.5249(6)	2.382/2.528
Sn1–C_{ipso}	2.193(3)	2.190
Sn1/Sn2–Br3	2.6875(5)/2.8765(8)	2.782/2.860
Sn2–Br1	2.9139(8)	2.994
Fe1–Al1	2.461(1)	2.395
Sn1···Sn2	3.2654(5)	3.297
Fe1–Sn1–C1	158.02(9)	157.86
Fe1–Al1–C2	103.81(10)	104.81
Fe1–Sn2–Br3	96.85(2)	99.03

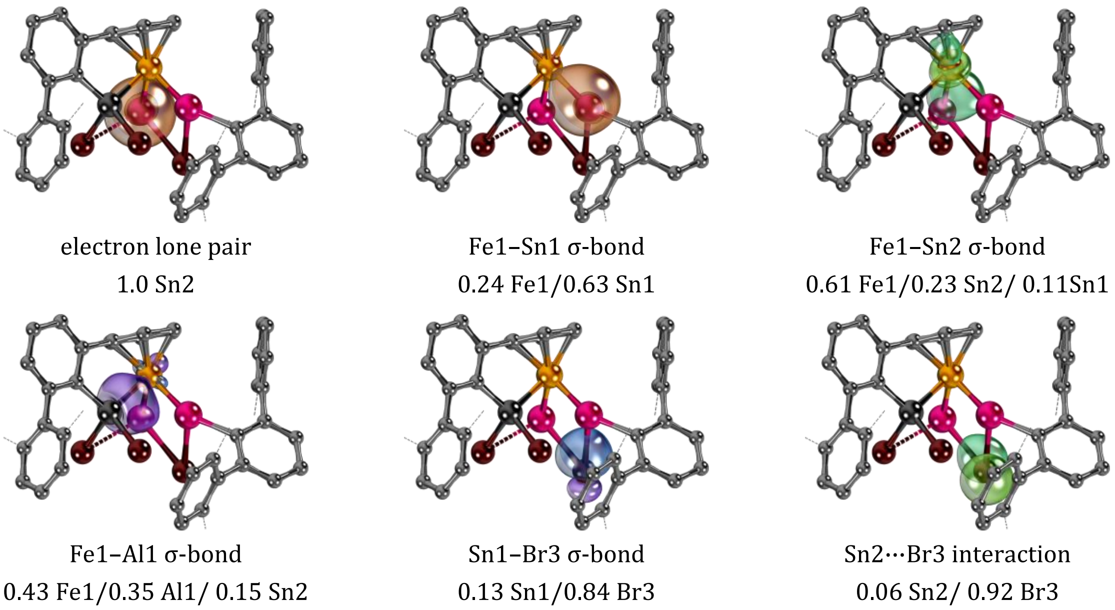

Figure S71. Selected IBOs of **3**. Dipp groups are omitted for clarity. The bond type and percentage of the electron density on Fe, Al, Sn and Br are given.

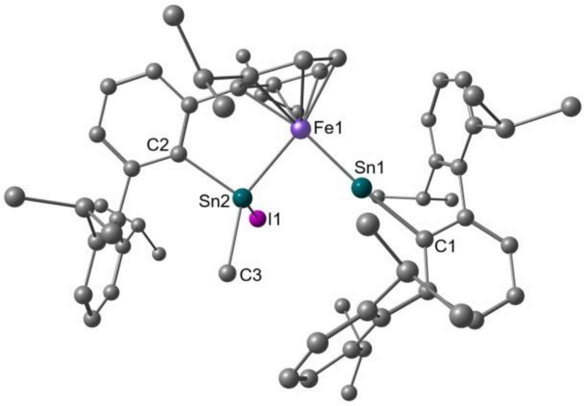

Figure S72. The optimized structure of **4** (BP86/def2-TZVP level). Hydrogen atoms are omitted for clarity.

Table S12. Comparison of the selected bond distances (Å) and bond angles (°) obtained from the crystal structure and the optimized geometries of **4**.

	**Exp.**	**Opt.**
Fe1–Sn1/Sn2	2.2960(4)/2.5188(5)	2.283/2.538
Sn1/Sn2–C_{ipso}	2.143(3)/2.183(3)	2.161/2.190
Sn2-C^{Me}	2.178(3)	2.160
Sn2-I	2.8086(3)	2.825
Sn1···Sn2	3.3804(5)	3.327
Fe1–Sn1/Sn2–C_{ipso}	175.45(8)/97.45(8)	175.00/96.55

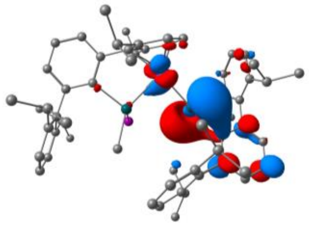

LUMO+1 (−1.47 eV)

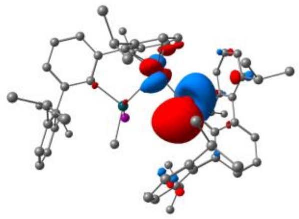

LUMO (−1.96 eV)

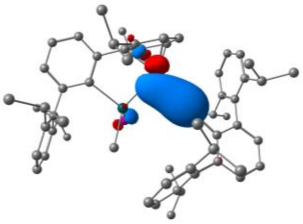

HOMO (−4.53)

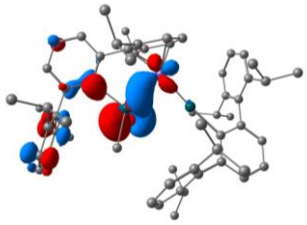

HOMO-6 (−6.21 eV)

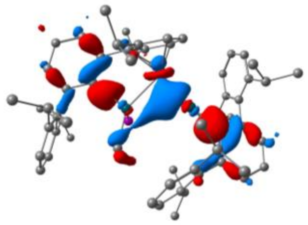

HOMO-19 (−7.56 eV)

Figure S73. Selected molecular orbitals of **4** (Isovalue = 0.04).

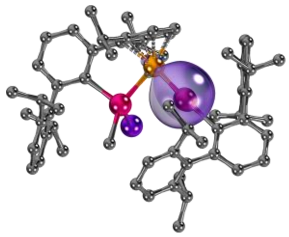

Fe1–Sn1 σ-bond
0.31 Fe1/0.50 Sn1

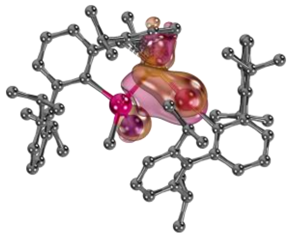

Fe1–Sn1 π-bond
0.58 Fe1/0.22 Sn1/ 0.08 Sn2

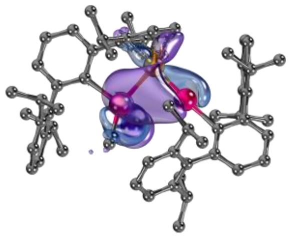

Fe1–Sn2 σ-bond
0.43 Fe1/0.27 Sn2/ 0.11 Sn1

Figure S74. Selected IBOs of **4**. The bond type and percentage of the electron density on Fe and Sn are given.

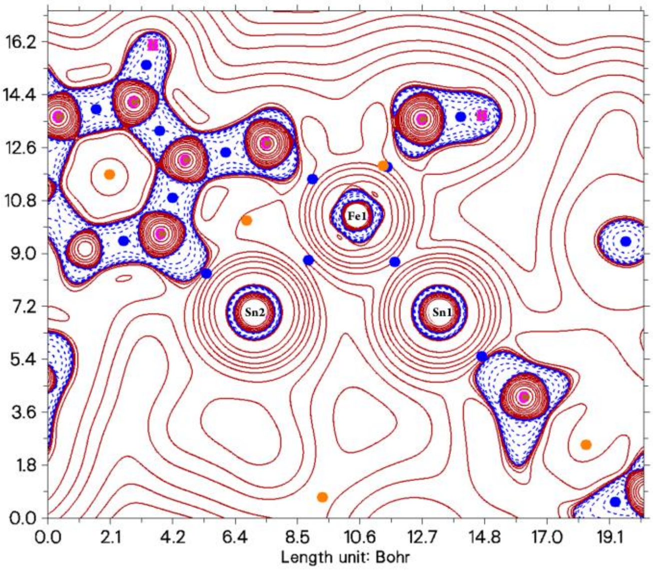

Figure S75. Plot of the Laplacian of the electron density on the $FeSn_2$ plane of **4** (BCP: blue dots, RCP: brown dots).

Table S13. Mayer bond orders of selected bonds of **4**.

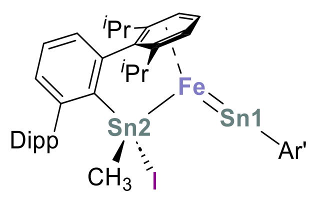	Mayer Bond Order (MBO)					
	Fe–Sn1	Fe–Sn2	Sn1–C*Me*	Sn1–C*ipso*	Sn2–C*ipso*	Sn1···Sn1’
	1.68	0.82	0.82	0.91	0.69	0.34

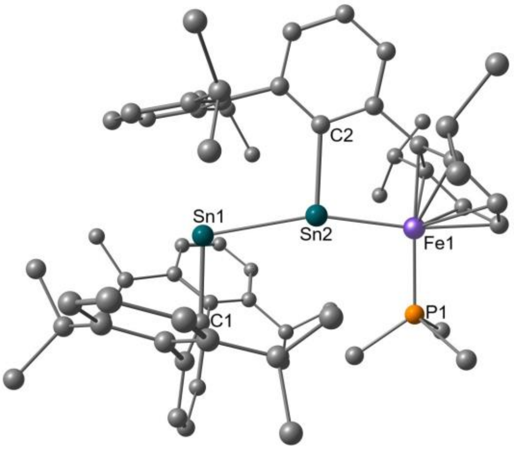

Figure S76. The optimized structure of **5** (BP86/def2-TZVP level). Hydrogen atoms are omitted for clarity.

Table S14. Comparison of the selected bond distances (Å) and bond angles (°) obtained from the crystal structure and the optimized geometries of **5**.

	**Exp.**	**Opt.**
Fe1–Sn2	2.4864(7)	2.464
Fe1–P1	2.2053(7)	2.184
Sn1/Sn2–C*ipso*	2.218(2)/2.239(2)	2.251/2.283
Sn1–Sn2	2.8614(7)	2.939
Fe1–Sn2–P1	100.20(2)	98.37
Fe1–Sn2–C*ipso*	99.78(2)	94.88
Fe1–Sn2–Sn1	160.123(11)	161.86
Sn2–Sn1–C*ipso*	105.82(6)	102.07

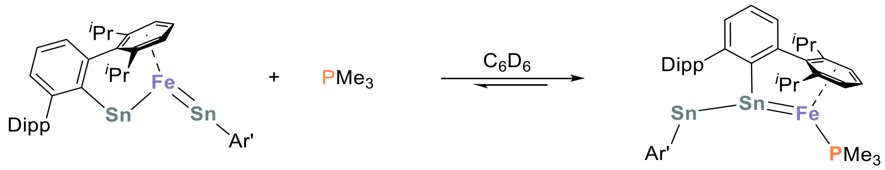

r²SCAN-3c	0.0 kcal mol⁻¹	−5.34 kcal mol⁻¹
B3LYP/def2-QZVP/D4	0.0 kcal mol⁻¹	−8.70 kcal mol⁻¹

Figure S77. Relative Gibbs free energies (ΔG°, 298 K) of **1** + PMe_3 and **5** (experimental value: Δ*G*°(298 K) = −5.08 kcal mol^{-1}).

Note: The CPCM (benzene) method has been applied to include the solvent effects.[23] The energy calculated at B3LYP/def2-QZVP/D4 level[27] of theory is in good agreement with the experimental value, considering the margin of error

associated with this theoretical approach.[28] While the energy calculated by using the r^{2}SCAN-3c composite method,[29] which has demonstrated superior accuracy in energy calculations, shows an even closer match to the experimental value.

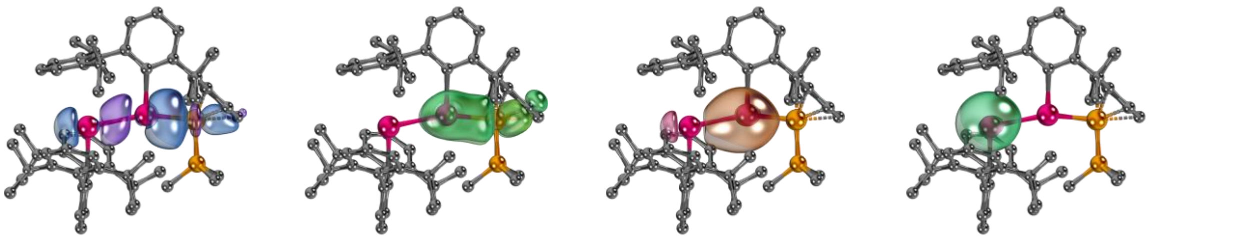

Fe1–Sn2 σ-bond	Fe1–Sn2 π-bond	Sn1–Sn2 σ-bond	electron lone pair
0.51 Fe1/0.35 Sn2	0.56 Fe1/0.26 Sn2	0.73 Sn2/0.28 Sn1	0.93 Sn1/ 0.05 Sn2

Figure S78. Selected IBOs of **5**. The bond type and percentage of the electron density on Fe and Sn are given.

Table S15. Mayer bond orders of selected bonds of **5**.

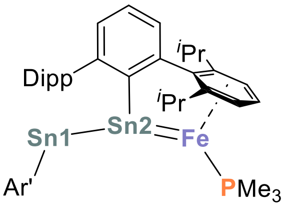	**Mayer Bond Order (MBO)**				
	Fe–Sn2	Sn2–C$_{ipso}$	Sn2–Sn1	Sn1–C$_{ipso}$	Fe–P
	1.34	0.68	1.10	0.90	1.05

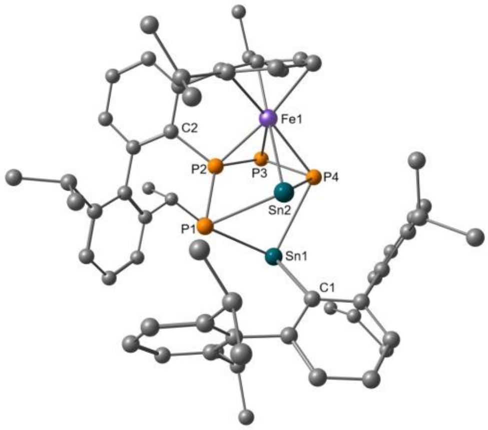

Figure S79. The optimized structure of **8** (BP86/def2-TZVP level). Hydrogen atoms are omitted for clarity.

Table S16. Comparison of the selected bond distances (Å) and bond angles (°) obtained from the crystal structure and the optimized geometries of **8**.

	**Exp.**	**Opt.**
Fe1–Sn2	2.7892(7)	2.808
Fe1–P2/P3/P4	2.2016(5)/2.3333(7)/ 2.4154(8)	2.185/2.317/2.401
Sn1–C1	2.2763(19)	2.282
P1–P2/P2–P3/P3–P4	2.1313(8)/2.1666(8)/2.1523(8)	2.132/2.167/2.150
P1–Sn1/Sn2	2.6658(8)/2.6272(6)	2.675/2.635
P4–Sn1/Sn2	2.8070(7)/2.7190(7)	2.796/2.733
Sn1···Sn2	3.1262(2)	3.116
P1–Sn1–C1	97.75(5)	97.03
P4–Sn1–C1	107.24(5)	102.99

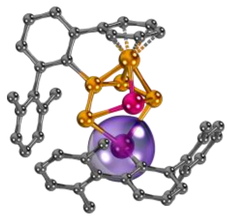

electron lone pair
1.0 Sn1

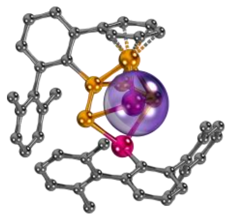

electron lone pair
1.0 Sn2

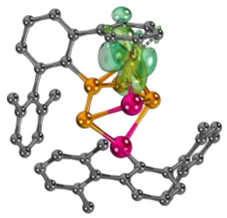

Fe1–Sn2 σ-bond
0.74 Fe1/0.08 Sn2

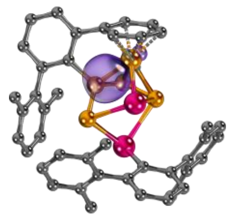

P2–Fe1 σ-bond
0.68 P2/0.28 Fe1

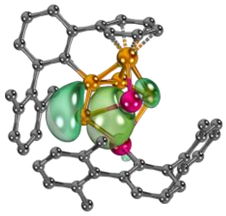

P1–Sn1–Sn2 3c2e bond
0.65 P1/0.10 Sn1/0.19 Sn2

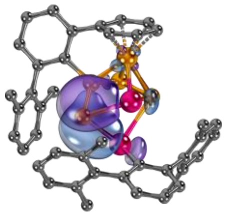

P1–Sn1–Sn2 3c2e bond
0.66 P1/0.12 Sn1/0.12 Sn2

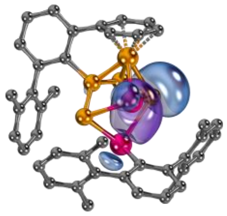

P4–Sn1–Sn2 3c2e bond
0.66 P1/0.15 Sn1/0.17 Sn2

Figure S80. Selected IBOs of **8**. The bond type and percentage of the electron density on Fe, Sn and P are given.

Energy Decomposition Analysis with Natural Orbitals for Chemical Valency (EDA-NOCV)

EDA-NOCV bonding analyses on transition metal-carbyne complexes have been reported by Frenking group.[30] In their study, the interacting fragments with several different electronic configurations were considered (Schrock *vs* Fischer-type), which accordingly led to different bonding interaction between those fragments, including the donor-acceptor dative bond, dative/electron-sharing bond and electron-sharing bond. In line with this, several different interaction modes of the corresponding fragments were considered to elucidate the nature of the Fe1–Sn1 bond in **1** and **4**. Tables S17 and S18 summarize the relevant ΔE_{orb} contributions to the total fragment interaction energy for **1** and **4**, respectively. The best description of the electronic structure of Fe1–Sn1 bond is suggested by the interacting fragments with the smallest $|\Delta E_{orb}|$ term.[25b, 30] Therefore, the donor-acceptor interactions, i.e., having dative bond, are both favored with the $|\Delta E_{orb}|$ of 139.62 and 140.05 kcal mol^{-1} in **1** and **4**, respectively. Deformation density plots associated with the σ and π components of the dative bonding interactions in **1** and **4** are shown in Figures S81 and S82, respectively.

Table S17. Summary of the relevant ΔE_{orb} contributions to the total fragment interaction energy resulted from the EDA analysis of complex **1** with the interacting fragments in different electronic states.

Bonding	**Dative bond**	**Electron-sharing bond**	**Dative/Electron-sharing bond**	**Electron-sharing bond**
Fragment state	[(κ^2-Ar'Sn2)Fe]$^-$<br>(S = 0)<br>+<br>[Ar'Sn1]$^+$<br>(S = 0)	[(κ^2-Ar'Sn2)Fe]$^-$<br>(S = 1)<br>+<br>[Ar'Sn1]$^+$<br>(S = 1)	[(κ^2-Ar'Sn2)Fe]<br>(S = 1/2)<br>+<br>[Ar'Sn1]<br>(S = 1/2)	[(κ^2-Ar'Sn2)Fe]<br>(S = 3/2)<br>+<br>[Ar'Sn1]<br>(S = 3/2)
ΔE_{orb} (kcal mol^{-1})	**−139.62**	−331.16	−198.59	−323.81

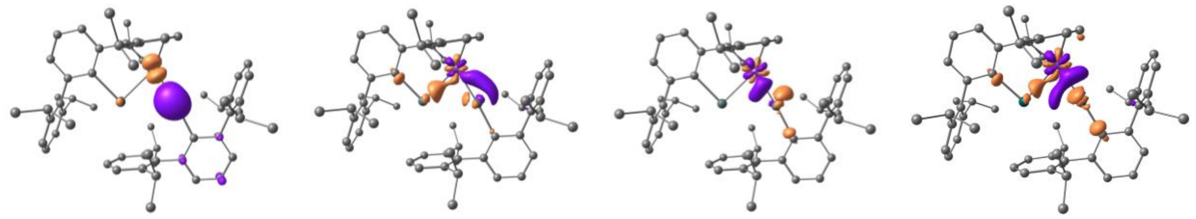

Δρ1, |ν| = 1.14	Δρ2, |ν| = 0.71	Δρ3, |ν| = 0.46	**Δρ2 + Δρ3**
$\Delta E\rho1$ = −53.0 kcal mol^{-1} (38%)	$\Delta E\rho2$ = −38.4 kcal mol^{-1} (26%)	$\Delta E\rho3$ = −21.1 kcal mol^{-1} (15%)	ΔE_{total} = −59.5 kcal mol^{-1} (41%)
Fe1 → Sn1 π-backdonation	Sn1 → Fe1 σ-donation	Sn1 → Fe1 σ-donation	Sn1 → Fe1 σ-donation

Figure S81. Deformation density Δρ plots of the pairwise orbital interactions between the [Ar'SnFe]$^-$ (S = 0) and [SnAr']$^+$ (S = 0) fragments in complex **1**. $\Delta E\rho$ = orbital interaction energies in kcal mol^{-1}. Isovalues = 0.005. |ν| = relative amount of charge transfer. Charge flow: Orange → Purple.

Note: The dominant orbital interactions demonstrate the strong out-of-plane Fe1 → Sn1 π-backdonation with stabilization energies of −53.0 kcal mol^{-1} ($\Delta E\rho1$). The second and third remarkable interactions appear to be mainly the Sn1 → Fe1 σ donation ($\Delta E\rho2$ = −38.4 kcal mol^{-1}, $\Delta E\rho3$ = −21.1 kcal mol^{-1}). Due to the presence of intrafragment charge redistribution in the latter two interactions, they have relatively low pair energies ($\Delta E\rho2$ and $\Delta E\rho3$). Nevertheless, the net σ-donation contribution ($\Delta E\rho1$ + $\Delta E\rho2$) is slightly higher than the π-backdonation contribution ($\Delta E\rho1$). Overall, the deformation densities illustrate the presence of σ-donor-π-acceptor type double bond between Fe1 and Sn1 in **1**.

Table S18. Summary of the relevant ΔE_{orb} contributions to the total fragment interaction energy resulted from the EDA analysis of complex **4** with the interacting fragments in different electronic states.

Bonding	**Dative bond**	**Electron-sharing bond**	**Dative/Electron-sharing bond**	**Electron-sharing bond**
Fragment state	[(κ^2-Ar'Sn2MeI)Fe]$^-$ (S = 0) + [Ar'Sn1]$^+$ (S = 0)	[(κ^2-Ar'Sn2MeI)Fe]$^-$ (S = 1) + [Ar'Sn1]$^+$ (S = 1)	[(κ^2-Ar'Sn2MeI)Fe] (S = 1/2) + [Ar'Sn1] (S = 1/2)	[(κ^2-Ar'Sn2MeI)Fe] (S = 3/2) + [Ar'Sn1] (S = 3/2)
ΔE_{orb} (kcal mol^{-1})	**−140.05**	−293.41	−148.12	−340.11

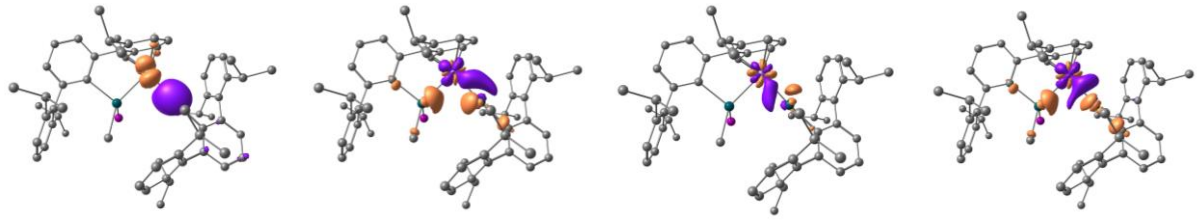

Δρ1, |ν| = 1.11	Δρ2, |ν| = 0.81	Δρ3, |ν| = 0.42	**Δρ2 + Δρ3**
ΔEρ1 = −49.3 kcal mol^{-1} (35%)	ΔEρ2 = −47.2 kcal mol^{-1} (33%)	ΔEρ3 = −16.2 kcal mol^{-1} (12%)	ΔE_{total} = −63.4 kcal mol^{-1} (45%)
Fe1 → Sn1 π-backdonation	Sn1 → Fe1 σ-donation	Sn1 → Fe1 σ-donation	Sn1 → Fe1 σ-donation

Figure S82. Deformation density Δρ plots of the pairwise orbital interactions between the [Ar'SnFeMeI]$^-$ (S = 0) and [Ar'Sn]$^+$ (S = 0) fragments in complex **4**. ΔEρ = orbital interaction energies in kcal mol^{-1}. Isovalues = 0.005. |ν| = relative amount of charge transfer. Charge flow: Orange → Purple.

Note: The dominant orbital interactions demonstrate the strong out-of-plane Fe1 → Sn1 π-backdonation with stabilization energies of −49.3 kcal mol^{-1} (ΔEρ1). The second and third remarkable interactions appears to be mainly the Sn1 → Fe1 σ donation (ΔEρ2 = −47.2 kcal mol^{-1}, ΔEρ3 = −16.2 kcal mol^{-1}). Due to the presence of substantial amount of intrafragment charge redistribution in the third interaction, it has relatively low pair energy (ΔEρ3). Overall, the deformation densities illustrate the presence of σ-donor-π-acceptor type double bond between Fe1 and Sn1 in **4**, which is similar with the Fe1−Sn1 bonding interaction in **1**. This result has been indicated by their almost identical Fe1−Sn1 bond distances and Fe1−Sn1−C_{ipso} bond angles.

Calculation of the ^{119}Sn NMR Chemical Shifts

The optimized geometries at the BP86/def2-tzvp level of theory were used for the calculations. The ^{119}Sn NMR chemical shifts were calculated using the GIAO formalism[31] as single point calculations with the TPSSH functional[32] and ZORA-def2-TZVP basis set[15b] for all nuclei except Sn, where the SARC-ZORA-TZVP basis set[33] was used. Solvent effects were considered by using the CPCM model.[23] The conversion of calculated ^{119}Sn absolute chemical shielding σ_{calc} to chemical shift δ_{calc} was done using $\delta_{calc} = \sigma_{Me4Sn} - \sigma_{calc}$ with σ_{Me4Sn} being the absolute chemical shielding of Me_4Sn calculated at the same level of theory (σ_{Me4Sn} = 2444 ppm with CPCM(benzene) and 2456 ppm with CPCM(THF)). The initial coordinate of Me_4Sn was modeled by Chemcraft[21] and was optimized at the same level of theory as used for the optimization of other complexes.

Table S19. Summary of experimental and calculated ^{119}Sn chemical shifts.

Complex	^{119}Sn chemical shifts (ppm)			
	**Sn1/Sn2**		**Sn1**	**Sn2**
	Experimental (δ)		Calculated (δ_{calc})	Calculated (δ_{calc})
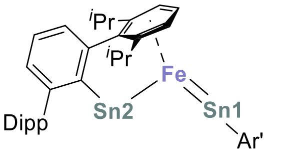 **1**	466	3229	168	3266
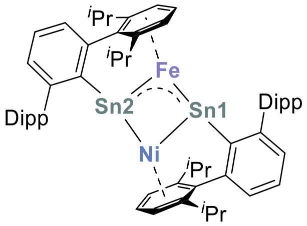 **2**	369	1701	−135	1854
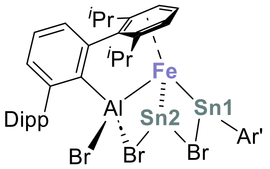 **3**	15	2393	−81	1991
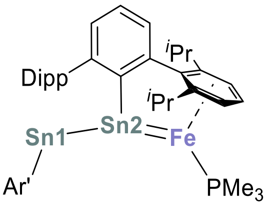 **5**	2403	1456	1626	1121
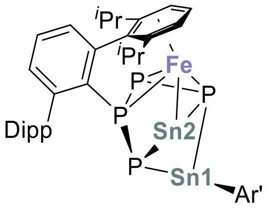 **8**	−455	−366	−815	−393

Note: The calculated δ_{calc} values are in reasonable agreement with the experimental data (the deviations are less than 500 ppm),[34] except the calculated value for Sn1 of complex **5** which has a large deviation of 777 ppm. This conspicuous deviation observed for complex **5** might be due to the presence of conformers in solution as indicated by its VT-^{1}H NMR spectra (Figure S17).

Table S20. Cartesian coordinates of the optimized geometries of **1 – 5** and **8**.

Complex 1

Sn	7.89541862238312	9.26134024342415	9.04066730993816
Sn	7.81331669374799	11.93160086202416	7.46796728668112
Fe	9.33681667961713	11.48004449609435	9.12134615172123
C	4.57270094528481	11.55432303300928	4.38626024211000
H	3.84549705464836	10.81013623496491	4.05644078179413
C	6.43455119390954	13.46157097742922	5.23857192136790
C	6.36621035331239	12.20287482738594	5.87390163437643
C	5.35893347130655	9.91287722586572	6.12750439019307
C	5.43166156196006	11.24227276355105	5.45118236351879
C	5.57387808081870	13.75320113392130	4.17658172935534
H	5.63035510522118	14.72731602654501	3.68736089247588
C	7.07702296181470	15.28912394534167	6.83928727353262
C	4.53471398032022	9.75022848990781	7.26370526870127
C	6.12340305901078	8.83403109106406	5.62857181799884
C	4.64496880434005	12.79718437668195	3.75274789661850
H	3.97147488839690	13.02360120128629	2.92485642656252
C	8.77579705876442	14.39253709851413	5.30008508457978
C	7.43938548113557	14.43710637602149	5.76773737671062
C	5.18858298938548	7.41102301638474	7.36361803868867
H	5.14478846158667	6.44355080700490	7.86229524650846
C	9.19043157515395	13.44612303365475	4.18413766734884
H	8.35962465827631	12.74302350608562	4.02250217158961
C	6.02027904603100	7.58878814639925	6.26107753589817
H	6.61156093462811	6.74755133458950	5.89547264607719
C	7.05540940252666	9.01178689929244	4.44001213548285
H	6.94681333183860	10.04746916482844	4.08608782540944
C	9.72208625001613	15.24231540395881	5.88763624639330
H	10.75481743936488	15.21991468278812	5.53674913913008
C	3.73877256818898	10.91268323543305	7.83684630826665
H	3.95280074383561	11.79790186689388	7.21994421514520
C	8.05609332022326	16.12047530391533	7.39773663904321
H	7.79144335749806	16.78233772712344	8.22389059199317
C	5.66230815391874	15.29931794042081	7.39712734084292
H	5.10982201338145	14.48405867062439	6.90783513027118
C	4.45874913862316	8.48647561308942	7.86224728288373
H	3.83209265533574	8.34455812691240	8.74412906939748
C	9.36634248368009	16.10267875850407	6.92414196267933
H	10.11707572068237	16.75550141955055	7.37270514590804
C	2.22703368866702	10.64984950157014	7.75252256612440
H	1.91687303317959	10.46237715856018	6.71477871604648
H	1.66202828170019	11.51482641983649	8.13064117546904
H	1.94141446022310	9.77245487205947	8.35162083019591
C	9.40160798629431	14.21751254701603	2.87098451686643
H	8.49286405731465	14.76538653883565	2.58447640444369
H	9.66042836720875	13.52834321085046	2.05366180968066
H	10.21789382015016	14.94886207418474	2.97124581376583
C	10.42926273162996	12.61691062715439	4.55421245950855
H	11.32236950587185	13.24929838114415	4.66768955379807
H	10.64376335957368	11.87941247593325	3.76748708510513
H	10.27774852586905	12.07451064514708	5.50009699693291
C	4.16883227346839	11.24023647440488	9.27507760271815
H	3.98670862634889	10.39243402653891	9.95123278666705
H	3.61025472786972	12.10683353963944	9.65822339492928
H	5.24248627796004	11.47674410685824	9.32263627452190
C	6.67363997075048	8.08278477672665	3.27795111013183
H	6.77226419762852	7.02563534487189	3.56595862862303
H	7.32793947366932	8.25726336367790	2.41093234807078
H	5.63337582867931	8.24943712629469	2.96462379445035
C	8.52401934803574	8.82075629629498	4.84919024127525
H	8.79278335776224	9.49739380372262	5.67415482756953
H	9.19530972336515	9.02747993277987	4.00261985895120
H	8.71392219151718	7.79283296820152	5.19014749711493
C	5.64004571625099	15.02575597386133	8.90854941992761

H	6.15489303640805	14.08382868409346	9.15073024367145
H	4.60354724920706	14.95010944730723	9.26766914068148
H	6.13134779725367	15.83147452104602	9.47408771778139
C	4.94588318758300	16.61471707755888	7.05178618843585
H	4.92199542690062	16.77729625379716	5.96499023836094
H	5.45681037533587	17.47509377050075	7.50959980841239
H	3.90986602701571	16.59963149850835	7.42084228697158
C	10.99737107754483	8.70202380730260	12.24183849759273
H	11.79681703805723	9.32644781148959	12.64635821798351
C	8.94430923843504	7.10117293910581	11.18025559162253
C	9.15782687781476	8.39466494163061	10.66998320168183
C	10.39572593337047	10.53561248407788	10.61900410824511
C	10.19014473205650	9.18286261646462	11.20399462658196
C	9.75288122141098	6.62328565882689	12.22383207017642
H	9.58326778269058	5.61792930561175	12.61551976480563
C	8.18766424072739	5.40744059472303	9.48893703908587
C	11.20878928773879	10.67680090220526	9.43863672021188
C	9.66862209836208	11.65654731171954	11.15378833911204
C	10.77120433249043	7.42070835512232	12.75272089928986
H	11.39417295197708	7.03883704444826	13.56314468056395
C	6.57557425650955	6.24566001043939	11.13629208136600
C	7.87894853346533	6.22946455379744	10.59493429580981
C	10.69602199685485	13.09680648063855	9.42591970409245
H	10.74381467093910	14.05955703618838	8.91833920208842
C	6.21088722027909	7.18848202359347	12.27220859792835
H	7.14126167204823	7.66673642855364	12.61174191697636
C	9.84257671260480	12.93214687231390	10.54330153327445
H	9.25378796552892	13.77945195662103	10.89183688998768
C	8.74097855493674	11.49022811177630	12.34409604787579
H	8.54184527351397	10.41257708435996	12.44163562903427
C	5.60238584480122	5.40489699935414	10.58178881677502
H	4.59118743372777	5.40590070960723	10.99314072280545
C	11.90485940338146	9.47665273307345	8.82186084802720
H	11.36724925406242	8.58351569377990	9.17446634690020
C	7.18785574507727	4.58182782860750	8.96012382997808
H	7.41472215914121	3.93902558318058	8.10750362127181
C	9.56603598729884	5.44230639118259	8.84642390642863
H	10.21116003689118	6.06180013762801	9.48597630434675
C	11.35531514235186	11.97457062448368	8.86869157949993
H	11.91989200942833	12.09419294939091	7.94522917776880
C	5.90617388140161	4.56914090369662	9.50824237398664
H	5.13911387827299	3.91437625529665	9.09081697210713
C	13.34986307672007	9.38662834047483	9.33829493998298
H	13.37134097096183	9.32224081117077	10.43515526949254
H	13.85444146085084	8.49725968581843	8.93293259390202
H	13.92839658172774	10.27414290574226	9.03907703967540
C	5.60449431702195	6.44970227576458	13.47354641329700
H	6.28257746998331	5.66533117351600	13.83838591856852
H	5.41272654898593	7.15046940835807	14.29953908187118
H	4.64751886874192	5.97266328043323	13.21416008027874
C	5.27856146239208	8.30255575418663	11.76996183391272
H	4.32142886609818	7.88636968489020	11.41983072231207
H	5.06561492413777	9.02989455085375	12.56727506289655
H	5.73108762607414	8.84141641788224	10.92200025582354
C	11.84833803057998	9.47070264115608	7.29097569572225
H	12.44850914356992	10.28149337875851	6.85169614997736
H	12.24303164562628	8.52233061305158	6.89934934982766
H	10.81178066741762	9.59071327180428	6.94649857468082
C	9.44646459479903	11.95730550791651	13.62747578574610
H	9.68807959371462	13.02981861667010	13.57118002989824
H	8.80484865116763	11.79829053839443	14.50660912985806
H	10.38545690166614	11.40841320371338	13.78538557975314
C	7.39098293156605	12.19052123620891	12.15455543440051
H	6.92685770467887	11.87236453522906	11.21082553177901
H	6.71424797510208	11.94378407700929	12.98523331334925
H	7.49347313120689	13.28567683673650	12.12747304935150
C	9.50373235849751	6.12000299416420	7.46928948632228

H	9.05426907523416	7.12386436080912	7.54173408701205
H	10.50882735876741	6.22530001552277	7.03537558195302
H	8.88535981347246	5.53793857869545	6.76886378142647
C	10.20531954246568	4.04953161494771	8.75637053345693
H	10.27033495222920	3.58130686419605	9.74854411940613
H	9.62504609071773	3.37891007447054	8.10520096975141
H	11.22161634776768	4.11859511169378	8.34098092463060

Complex 2

Sn	8.69709698514659	8.90066535520353	5.50863894641560
Sn	7.87362990149195	11.18523243001547	7.07902740139173
Fe	8.23138166089158	9.00196837727586	7.93971004709085
Ni	8.49798763562920	11.04063474382579	4.46363787837095
C	7.41653992385781	13.28320742557361	6.72914668951805
C	6.95362506990899	14.05201595039860	7.81162057760755
C	7.58344126944685	13.86470767185894	5.46237984467890
C	7.85431096319071	12.67729727560556	11.22427610788957
H	8.70755640292557	12.66053646927456	11.90476462724476
C	9.28761372457456	8.63805823419558	9.71226037860658
H	10.02130715660916	9.26805781105825	10.21379352740921
C	6.64130141152987	15.40276597246264	7.61473791955275
H	6.27794480580039	15.99968597203776	8.45347311328951
C	10.87138906986671	7.43362194045530	2.67093478047623
C	6.80291141487223	15.98194383026369	6.35199656408415
H	6.56089471111348	17.03516759386477	6.20262083379222
C	10.50115027691747	13.54523136493182	5.09387092108939
H	9.93066231400292	13.87634381377126	5.97417487933743
C	6.94211965895838	8.07059765357636	9.30169860996896
H	5.88728780835972	8.26564985475455	9.49266376960345
C	7.91814761158312	8.78063152756713	10.04102558130880
H	7.61050855982693	9.51926502500004	10.77975585099665
C	8.09443558563803	13.03666767934252	4.33279069415784
C	7.31145999809507	7.18946841090994	8.24263824291030
C	11.58759479013197	12.58276340838279	5.58549360412454
H	11.13458852424790	11.70121587619653	6.06150799302002
H	12.23418801390363	13.08332362843407	6.32011365074650
H	12.22804724695418	12.22986314558906	4.76424214319587
C	9.78270429484223	8.18830639205982	0.63331428572241
H	9.82789833610162	8.72302857832277	-0.31746970422505
C	7.94996488674086	13.37601042449538	10.01449285771081
C	9.43297130974788	4.83158816605818	6.88467992617642
H	9.36845604647560	4.34532543390136	7.86008969297826
C	9.71234326253059	7.77497170320243	8.65995031441768
C	12.08600033144027	7.44846085619212	3.58525157639694
H	11.84685432093082	6.82763983269882	4.46094599510573
C	5.77657191189441	5.21472716899395	8.22993190728400
H	5.30365949712624	5.51301657760213	9.17812026951188
H	6.61999492636017	4.55194761399234	8.47010284588351
H	5.04189211692258	4.63822962418081	7.64844757257280
C	9.67749685449062	6.78183894887305	3.04925982305391
C	9.51583995504300	12.87478710296337	4.15195807484517
C	6.83650154089434	13.38173434907990	9.14355698845070
C	7.16793516055342	12.37017226550267	3.45501618288296
C	5.66946116688494	12.49070555413327	3.67068527476102
H	5.52515798199878	12.96882544393678	4.65073234723010
C	12.36261209944194	8.87256715931882	4.09471846155185
H	13.16983483157620	8.87150368404971	4.84156023417182
H	11.46356548433344	9.31048773965078	4.55491058854479
H	12.66236330211745	9.53797940121976	3.27035723346389
C	7.27733424484558	15.22025776349654	5.28003024266338
H	7.40877444894678	15.67767837461865	4.29738202217938
C	11.86912174982949	6.66127633665930	9.26150043597559
H	12.92575909890322	6.52007605148075	8.98981227828124
H	11.37401397422721	5.68039229457262	9.23259722960175
H	11.82889261345308	7.02918482600760	10.29815712224442
C	9.25125960415690	14.07027233782390	9.64214959285684

H	9.09038005284808	14.58051002395213	8.68128428193846
C	13.32485072864821	6.84141352833310	2.91122493775019
H	13.62757100141858	7.42441379553149	2.02835378289878
H	13.12941945409152	5.81119253222646	2.58183130654923
H	14.17571051138165	6.82486589530246	3.60829170711983
C	8.71259311437914	7.02362415847887	7.93923103191782
C	9.91309668768391	4.71174747413753	4.50356029002571
H	10.22043147875898	4.13477366415987	3.62920728141952
C	5.60591741432370	11.98313227779362	10.69012568434625
H	4.70503216068540	11.42715262612848	10.95471667370477
C	6.69772359462118	5.20287002072478	1.69732970908946
H	6.48840603465648	5.64151834073807	0.71005518547853
H	5.76761762617061	4.74378317951158	2.06383431552671
H	7.44369939936370	4.40744174759552	1.55974327674247
C	8.52242753374898	6.87228598470016	2.24238956934530
C	4.47450386699512	12.62176270787603	8.51980294530667
H	4.78828716853055	13.09815697530991	7.57926409266666
C	5.04674799720231	13.40706735148101	2.60566894498428
H	5.52004788863292	14.39913520278379	2.61084042402689
H	3.97069256319147	13.54008612711690	2.78920119688723
H	5.16994471007913	12.98006647560686	1.59891809466028
C	11.91373154112083	8.99967792866518	8.27304529520971
H	11.98594330091620	9.45323062420206	9.27272210761174
H	11.38185961969505	9.70566534151390	7.61948289140081
H	12.93846458485078	8.86921673626216	7.89611723093145
C	11.18456095506961	7.65232778886423	8.30622342743618
H	11.23365674740296	7.22646147653875	7.29258143569557
C	10.90754429337265	8.12486853011032	1.45371769870187
H	11.82721355825199	8.62753188447028	1.14801308977395
C	9.82368337904772	4.09966068508043	5.75799198277903
H	10.06258142299276	3.03979378757780	5.85958419643610
C	7.19999978009149	6.26630975577407	2.68508994089503
H	7.37196967658641	5.76763983233819	3.64991399958793
C	9.97600031328365	12.11559919120945	3.04588548620596
H	11.04597956532896	11.96735819614802	2.90269548457381
C	9.06870888633667	11.50730734877813	2.15789111475282
H	9.44216893018063	10.87702078919992	1.35156158328492
C	10.38173447214537	13.04784474684810	9.43919369958505
H	11.30384123661828	13.54723676203777	9.10739214849647
H	10.10601307533906	12.29445576840418	8.68554331340519
H	10.60178741642831	12.51058053197809	10.37381156958153
C	6.14570520267712	7.36134773352858	2.91361944641842
H	5.89331572852310	7.87103045064296	1.97121036452725
H	6.51315757566440	8.12929516749513	3.61169401988142
H	5.22032949769095	6.93280752558296	3.32529766417757
C	4.98067574567919	11.12115704064555	3.72049318735161
H	5.06487872194150	10.58693229435137	2.76293499353228
H	3.91092850454080	11.23850502450493	3.94570204764876
H	5.43930535231957	10.49138976856959	4.49573106731672
C	5.66222003871399	12.66476579634899	9.46819991109073
C	9.64309103598758	15.13927345077482	10.67320315232317
H	9.84519681346080	14.69089127085037	11.65737530930610
H	8.84160965066768	15.88061359138991	10.80040402168089
H	10.55376248123003	15.66691192501755	10.35354736075178
C	6.68949422504193	11.99321657112807	11.56593699641026
H	6.62979653996971	11.45637160513978	12.51435354984896
C	3.29795730953328	13.43838818978674	9.07797183387764
H	2.94329822647382	13.01882390187613	10.03158462907994
H	2.45401257712670	13.43689569905073	8.37222056761210
H	3.59322193908478	14.48152845169878	9.25984266136189
C	11.09843734446757	14.79718806527791	4.43177993197264
H	11.77380977000142	15.31746544154597	5.12643140798544
H	10.30821825867794	15.50006367406684	4.13207425985624
H	11.67401199651718	14.53110224876593	3.53217437593869
C	6.24891980223639	6.45045061310425	7.44712059079402
H	6.73002816115121	6.09856721945919	6.52193290058645
C	7.68191643155432	11.62751856966366	2.36146967962296

H	6.99537258692480	11.10711189745175	1.69405718272052
C	9.12465401699253	6.19254908551609	6.76242259744371
C	5.07251552292223	7.34312524152147	7.03805447149824
H	4.39059443408745	6.79385904855121	6.37275044169300
H	5.43839875170547	8.23685306844019	6.51328895411722
H	4.48712069664755	7.67783083465768	7.90708904080567
C	4.05528430616246	11.18148496676652	8.18840047386403
H	4.90605168477805	10.60167808340567	7.79975087377990
H	3.25919846996915	11.17831637744928	7.42983181460262
H	3.67272394122853	10.65711313867746	9.07673495098578
C	9.60537156908635	6.07277279519648	4.36318363367750
C	8.59643073383244	7.57678669336068	1.03436042894243
H	7.71099521362828	7.65177056567628	0.40011596312929
C	9.21233955034275	6.79887267065522	5.49548396288111

Complex 3

Sn	4.82811614289247	13.04670206768696	5.13194842630602
Sn	7.73270041073373	11.99617365475425	6.28571414671243
Fe	5.32456491786218	11.49546385286406	6.87039321319989
Al	5.64117942133327	13.53395770231359	8.08708394480162
Br	7.30263321693895	13.99552827996261	4.28665368924706
Br	7.96651432724266	14.19195614938890	8.30744936650938
Br	4.74194631759418	15.41444769225804	6.83919133275791
C	4.87043190722689	15.53060514370226	10.76593496579598
C	4.57617613246720	10.98349340559011	8.75653779148841
C	4.00808543730325	13.57194586865884	12.11608338443646
H	3.82185147489459	14.24804505968254	12.95192978816551
C	4.42404856618264	11.85209074573872	9.96704238115163
C	4.04425309548332	13.76565670438439	3.21766382065801
C	4.01883980200600	12.74299431678162	2.24209012077905
C	3.49778298195396	10.91498376072325	7.81589538146219
C	6.13759822172525	16.05262130635772	11.11276710897333
C	3.59989689335922	16.23971094651106	3.78645325111597
C	6.40202918302150	17.40245549511162	10.84781005593233
H	7.37934872770202	17.81700693750375	11.09363549697166
C	7.17870144818027	15.17899159684705	11.79919358095760
H	7.06853093224418	14.16149598662000	11.38946865911530
C	3.87845432884950	16.36051766257242	10.18958062069002
C	2.74652439760556	10.70002339013491	2.95847431428743
C	5.80328213096965	10.26549276686504	8.52651477411569
C	5.14690858731144	10.48506220749935	2.47102587571728
C	3.85279975489373	11.35950486144889	11.14744925746163
H	3.54254483039647	10.31488188141174	11.21423574691559
C	3.69253561762481	10.17751240229733	6.61576948801751
H	2.91862118043925	10.17601184020163	5.84901103876622
C	3.85774635038120	14.42094471869905	0.49561834725253
H	3.80790137777572	14.68371821472443	-0.56179233793651
C	2.16369687536592	11.58438551514504	8.10211441111258
H	2.23510301617550	12.02475403451921	9.10605995113628
C	3.93435344178497	13.08611971487601	0.88448949202351
H	3.91396019419592	12.28970379884692	0.13929986593618
C	2.69489048632747	9.32057293406744	3.19324943609478
H	1.74862577764412	8.85727554826469	3.47293453981081
C	6.85190342626486	10.20988380798345	9.62560010270006
H	6.73059718116230	11.11958851392600	10.23294861065154
C	3.83155519305654	8.52527609049546	3.05567555075540
H	3.76953217225083	7.45008485399414	3.23198010755221
C	3.98844733070676	11.28517777565215	2.59675639906750
C	6.48643251295459	11.08662601811029	2.08457956461007
H	6.43214898643282	12.16893068952650	2.27287500378591
C	4.54838174144773	17.27112970813854	3.95553225609369
C	3.78462755311637	15.41484609048288	1.46769763804375
H	3.63270271816847	16.45468556886765	1.17595490983263
C	2.04020934207803	17.43819433108516	5.20234853703055
H	1.07386788524224	17.51247202528791	5.69881286484399
C	8.62392120098617	15.63030201356356	11.55820334265217

H	8.83023276378200	15.77705867157041	10.49061606012705
H	9.32119309184594	14.87044614138640	11.93905373130012
H	8.84679521412196	16.57001893428775	12.08616505736297
C	2.48567659744868	15.82006147821139	9.89368785154508
H	2.59871535292590	14.75770336177237	9.62362357697743
C	6.53845912262755	8.99631443740111	10.52163636242512
H	6.64614888041490	8.05924094360813	9.95441850892478
H	5.51310294325688	9.04115877287238	10.91067735066212
H	7.22753125884851	8.96013891929231	11.37760988344558
C	5.92743192441190	17.21372205789935	3.31891470915788
H	6.07778560587808	16.19056523706448	2.94309060962277
C	4.21330863521033	18.37343568843707	4.75395773262654
H	4.94756108465677	19.16496132498813	4.90761218706371
C	8.30478516638367	10.15950116431336	9.15266531610901
H	8.97385050271392	10.04520527841235	10.01736705779785
H	8.60405045064415	11.08981114092559	8.64621880911042
H	8.50037301239878	9.31484761890110	8.47606019918172
C	7.63320410369679	10.52745722958087	2.93535245017444
H	7.89134086336872	9.49588313494221	2.65326562972808
H	7.35508924020858	10.51790478847105	4.00134211921585
H	8.53413017485490	11.14465464137118	2.82128931479390
C	6.90982520822899	15.10819931068235	13.31440213888060
H	6.96577179850730	16.11328839196055	13.75950396526815
H	7.65928378994894	14.47256955839177	13.80984370440356
H	5.91727000759050	14.69629567944346	13.53319658579147
C	1.28846752624124	15.21949085667231	4.24250855947514
H	1.82905491322598	14.26002636058742	4.28313087218489
C	2.97120455540277	18.46223794556063	5.36977741546119
H	2.72806388037326	19.32301298130069	5.99449786576550
C	6.03174115026010	18.17847833829852	2.12576202160300
H	5.86750899958116	19.21711707599831	2.45046238569161
H	5.28811747733822	17.94962308441025	1.35018291721547
H	7.03063877262757	18.11922734219070	1.66863460821863
C	0.43194036447641	11.01920163660802	4.00366398368150
H	-0.37865923487051	11.75360983232005	4.11100671311481
H	0.86949389776651	10.85594132260319	4.99693009910722
H	-0.02626295726716	10.07447252383210	3.67645305840113
C	7.04011229999563	17.50107214798783	4.33943761829529
H	8.02451746059845	17.30527352392532	3.89118057844071
H	6.93836893119741	16.86681734360404	5.22870510077684
H	7.02514254222830	18.55236738774878	4.66345486858112
C	0.23125178827454	15.18789941226038	5.34947934205233
H	0.68923409686039	15.19490438698459	6.34581578543390
H	-0.37647669342795	14.27677779204060	5.25534043274013
H	-0.45516224187923	16.04530044522051	5.28235532331117
C	1.05110828047503	10.52353979673336	8.14610748099736
H	0.09655991984556	10.98044953988920	8.44363120362822
H	1.29181747510252	9.72931942066071	8.86677705160001
H	0.90523007283858	10.04922971101878	7.16443340037049
C	1.77587634044377	16.52349690944225	8.73309996647980
H	1.51494538664333	17.56287866232753	8.98344601285214
H	0.83340808799324	16.00381037833427	8.50623638607022
H	2.39134172275539	16.53428206933656	7.82543434297957
C	0.84283847413693	11.59473168531783	1.59031620024299
H	1.52832893218202	12.05282500553166	0.86668127291954
H	-0.08127188631859	12.19073814055627	1.60761778592872
H	0.59343492918102	10.58318298777972	1.23603574896230
C	1.82166263126281	12.72388722368334	7.14024777728672
H	1.86641751310268	12.40346738437414	6.08866542982420
H	2.50788943163033	13.57241372489090	7.26824656259732
H	0.80103111848857	13.08481969432227	7.32692112188841
C	4.57538961076434	14.07350975995056	10.93614954650079
C	4.81051758443169	13.20409055922513	9.85077081968680
C	5.04499202326276	9.10597201049242	2.69837520965157
H	5.93220745479860	8.47955351586410	2.59771166178223
C	4.18738317524574	17.70271778639309	9.93927388037669
H	3.44282843053013	18.34912359081440	9.47489739710231

C	5.44067390197155	18.21959668898480	10.25793386325838
H	5.67073558463150	19.26439221624912	10.04253558520318
C	1.46891581483878	11.52658606886948	2.99593666133460
H	1.74511299337062	12.55214164698486	3.28120395977259
C	2.33143171744758	16.31642079052573	4.42041361774818
C	3.65830185888821	12.22336435964863	12.22596002240010
H	3.21390601399462	11.85056800694131	13.15011284936646
C	5.94320760595524	9.52700339543207	7.31473522500799
H	6.87631992036531	9.00054079481471	7.11021265190697
C	4.90096158812869	9.48703456412708	6.35844584908646
H	5.03862383989782	8.97733314332998	5.40748487096639
C	6.78102071907619	10.88695118701060	0.58823726353926
H	7.75473711622571	11.32744082464825	0.32886734409788
H	6.01543545822129	11.36193002180029	-0.03900625819437
H	6.81044762964111	9.81603063404645	0.33600027733016
C	3.86001093670556	15.11205911289222	2.83838694580213
C	0.59840667141552	15.30578691584547	2.87008324016270
H	-0.14003049118246	14.49733273300500	2.76090220659895
H	1.31405308825484	15.22687752937089	2.04323943494641
H	0.06930874052201	16.26572341203828	2.77349522027456
C	1.60475554033289	15.88948277198704	11.15572674834529
H	2.03721181273551	15.31821412971788	11.98632835961439
H	0.60217686448489	15.48518473889919	10.94883269548766
H	1.49267089234319	16.93413545741221	11.48332002940433

Complex 4

Sn	8.32799917503265	12.31048004287059	12.91762188861970
Sn	9.09749228493499	15.54601543061703	12.86264691675391
Fe	7.28717710416955	14.44222213990115	12.01556348529167
I	10.37766405384929	11.46807477791320	11.16495859784293
C	9.32026533599952	11.72807007484636	14.74528932893235
H	8.76681824258402	12.08744802253189	15.61898398977271
H	10.31035872079105	12.19290296614025	14.73793162333861
H	9.41301679476203	10.63740093352081	14.77096282679404
C	4.50458706728070	13.34503055580920	13.48193996158717
H	4.84678648036479	12.30661457297221	13.59963875336213
C	4.76111910669875	14.06585500594200	14.80742786027883
H	5.83671173183526	14.09643800627076	15.02733073557487
H	4.25227685424519	13.54198278242115	15.62811947917961
H	4.38705886990291	15.10017257978034	14.79677610986697
C	3.00534302485640	13.30820182994690	13.14195908027288
H	2.61765968017664	14.32634497891024	12.98517095904330
H	2.43273907924546	12.84487372969188	13.95867622862077
H	2.82228897534627	12.73007094351541	12.22606026409305
C	10.77039581691815	16.73077622618600	13.54645854994397
C	6.70904907699767	10.96569021862645	12.31072426423554
C	5.86684351366674	13.10302454798738	11.29936477553335
C	6.59311963688540	13.66036023918056	10.18794762851132
C	6.70618836073354	15.07237447609110	10.10921024607214
H	7.31536791973908	15.52627146935225	9.33123663167121
C	6.11315870129593	15.90679026202623	11.08878146192656
H	6.29970558236132	16.97881969760916	11.05624081431232
C	5.41745139854475	15.34612838868861	12.18945357701639
H	5.04173087889671	16.00268263159151	12.97255932316238
C	5.27828361548611	13.94006758373952	12.32024517688369
C	5.77685431171006	11.62203099669181	11.48751359976803
C	4.69132765193251	10.91771001278253	10.94943488366645
H	3.96620823606501	11.43625898671889	10.31991600697386
C	4.52194019425943	9.56626407680163	11.25275054951632
H	3.67305514909724	9.01750658861615	10.84233032671019
C	5.41332944203503	8.92789463083846	12.11666271172793
H	5.24223589112997	7.88995015707236	12.40696673180370
C	6.51158570287908	9.61611807932468	12.65490654643808

C	7.36773440914923	8.97005085280724	13.69621853458169
C	7.00444423705325	9.14638401553056	15.05071276082265
C	7.81499065161623	8.58705020399593	16.04672290781603
H	7.54814630336910	8.71886459089983	17.09673866010868
C	8.96304804154431	7.87407623938192	15.71434434481999
H	9.59447167294765	7.45578521127019	16.50005084904820
C	9.30695031770789	7.69502084548036	14.37486304471753
H	10.20873889098693	7.13657500138764	14.12523856645170
C	8.51907312263146	8.22767969719672	13.34669051571872
C	11.07476288743269	17.82610113524047	12.71266584515505
C	12.07910748037752	18.72426820528296	13.08602344998996
H	12.31603760173223	19.56853521339494	12.43629462547478
C	12.76402391874128	18.53860074126394	14.29054097819522
H	13.54362930731652	19.24177606514176	14.58669851051137
C	12.44780101195124	17.46220954670288	15.12207212467426
H	12.97601824264327	17.32533533172903	16.06758127671127
C	11.44882151783653	16.54419256190204	14.76189126498254
C	11.10761452371306	15.41222980607865	15.67069160193176
C	9.93677452717631	15.48025582151945	16.46052834921257
C	9.67441705151508	14.43835131841377	17.36159898460894
H	8.77891035450334	14.47790812835808	17.98294384834865
C	10.55038021124886	13.36535600223256	17.48719488177488
H	10.33519138175922	12.56483872515348	18.19632842197416
C	11.69358563429140	13.29801996974187	16.69056101622604
H	12.35676491014533	12.43840742813474	16.78281746943486
C	11.98631545875163	14.30427407452957	15.76489859347276
C	10.26807542266094	17.97790912949810	11.46210583639761
C	10.67448282592936	17.30698508943123	10.28350144812493
C	9.84994274142988	17.37597333098243	9.15408256191756
H	10.14789558972906	16.85953719770979	8.24048650340747
C	8.64994430488830	18.08414408042362	9.18644249037067
H	8.01850979520673	18.12778978676922	8.29744719968575
C	8.25009968113698	18.73104729925961	10.35427315394930
H	7.30721465019257	19.28118202870557	10.36884416895690
C	9.04170748631849	18.68486035523400	11.50977870175611
C	8.58367303414456	19.39264095318191	12.77658412227550
H	9.29946127178315	19.13958691580419	13.57257114969516
C	8.61899089414188	20.91833935919069	12.59387606950160
H	7.92807918107393	21.23750310326988	11.79938764157123
H	9.62700407846515	21.25933428601140	12.31948556628529
H	8.32422894754815	21.42621330756612	13.52382791950889
C	7.19800364197437	18.91522878179832	13.23628554116067
H	6.92304517634079	19.39651251912470	14.18579591069811
H	7.18299835374057	17.82470911710491	13.38940020740876
H	6.41980970831853	19.16301829951586	12.49910495426442
C	11.97701152013600	16.52571104977924	10.23398128209024
H	12.38209225688400	16.50004996257100	11.25597539268890
C	13.00582520967037	17.24487872754288	9.34601504460712
H	12.65269446642803	17.30715543587180	8.30568950524623
H	13.96189936058598	16.70141834808652	9.34684988440856
H	13.19015695054265	18.26891251330145	9.70092820841604
C	11.76243544111301	15.07356955337879	9.78840466552204
H	12.70564322153464	14.51217274794784	9.84192153919206
H	11.39590884768551	15.01455728023723	8.75325929996555
H	11.03397322014680	14.55486765525461	10.42837393400215
C	9.02919947708146	16.70187127284232	16.42976715247885
H	9.24417472177422	17.25494691877098	15.50283881907461
C	7.54001163555528	16.33957182703950	16.40610555302712
H	7.31594964503439	15.67734955165817	15.55699788411110
H	6.92829850563675	17.24690674219130	16.29858778615423
H	7.22179180391153	15.83368108845714	17.32911780080957
C	9.36916739882260	17.63777922976323	17.60200464324492

H	9.18761760450875	17.13744525965480	18.56500171932687
H	8.75230318789905	18.54814783731603	17.56645976285957
H	10.42649913009012	17.93638196233759	17.57098637787931
C	13.17305417971878	14.18561911581966	14.82000623868975
H	13.56243203525508	15.20107613349299	14.64899776339931
C	12.70160054895179	13.64143694597698	13.45936534432425
H	11.91616146307936	14.27294840785246	13.02055103963179
H	12.29161916292243	12.62625831012671	13.55398181675942
H	13.53316930876690	13.60130972635417	12.74092048054073
C	14.32460778350410	13.33594222652168	15.36737191934391
H	15.18744868112156	13.39672930515090	14.68918072799534
H	14.04808786910979	12.27389597011544	15.44213560333197
H	14.64577235590706	13.67682696889653	16.36210054890747
C	5.73365563789113	9.88648168694810	15.44622211098337
H	5.33982393234119	10.37815592076856	14.54574528879814
C	4.66530651673153	8.88953497928180	15.92560623800795
H	3.72582741356473	9.40910756926307	16.16701437511954
H	5.00291668955270	8.35666247207790	16.82717687387621
H	4.45500464767933	8.13861015669575	15.15127402545418
C	5.98232721859772	10.98067645759675	16.49187423595879
H	6.39767158431649	10.56849949570442	17.42284763111292
H	5.04050212425303	11.48868461508576	16.74503361704021
H	6.68426524683689	11.73582849057257	16.11426505306866
C	8.87477228040568	7.97925419596430	11.88905137234823
H	8.57048033899084	8.87534787493292	11.32392193461870
C	8.08678758337613	6.77679060850557	11.33595424619189
H	8.34669521976695	6.60249586730600	10.28114822365623
H	7.00324743814534	6.93750426333914	11.39510259408780
H	8.32891374481056	5.86556338626356	11.90396921748604
C	10.37414535509320	7.76913518975713	11.65242521771836
H	10.96753437665827	8.57038137921351	12.11086542243898
H	10.58442585893147	7.77428701014154	10.57376148871151
H	10.71620879690758	6.80086321341395	12.04956915519682
C	7.22584643977836	12.76915943733639	9.13790492379806
H	7.53393077317637	11.83942111723739	9.64231237477983
C	6.19298794290443	12.41002122861483	8.05602839387109
H	6.64792582748678	11.75955683792521	7.29484963786221
H	5.82720693101006	13.31899661609497	7.55400816745476
H	5.32908580445381	11.88218110596675	8.48008137433126
C	8.47604978728683	13.38958032682090	8.50979987255888
H	9.16851777644446	13.73713746752341	9.28571087456825
H	8.22404135536803	14.23587112180821	7.85196840655067
H	9.00085524798329	12.64033651664376	7.90191044551538

Complex 5

Sn	4.10992248343102	16.18920766252674	22.53277340139142
Sn	4.16611530615351	16.40965722709576	19.60246724239664
Fe	4.21330190566006	15.26269584193483	24.81337622143832
P	4.12041083090612	13.14253263834638	24.29947396147196
C	4.31606937466243	18.36365886287845	23.19680598270913
C	4.42822974413538	18.47496903928478	24.60225818003469
C	4.57981143293112	19.71556836925383	25.23195575134451
H	4.66860152322051	19.76063544358650	26.31943313738538
C	4.24451855912158	19.57967745251137	20.95569503399571
C	4.40178685080978	17.23983801215421	25.44101955346448
C	4.51066591389266	20.79948008077681	23.08483661762518
H	4.53646097987564	21.70593686132876	22.47577873747837
C	4.36407597655299	19.55615661485933	22.44372859727897
C	3.13970488480729	16.71477641994525	25.88075141094006
C	2.96510109007535	19.55107096035641	20.36193744036234
C	2.68583178701696	12.21854500666089	25.01866499391971

H	1.75001434246094	12.61692626870211	24.60768070144056
H	2.66813368723895	12.36069299252033	26.10692692183905
H	2.74733761178709	11.14219078211814	24.79833225858564
C	1.60315144889970	14.51423844303757	18.64394313437090
C	2.84904789602248	13.67574689066929	18.61596838427789
C	0.74044030281402	14.53276217667368	19.76490414550329
C	2.85981405442305	19.66146296506713	18.96656591812502
H	1.87475785864677	19.64072678273261	18.49714033935278
C	4.08678154736919	14.25210179765519	18.96643169498164
C	5.40785824656858	19.69264660592856	20.15559337749040
C	6.58608995084814	14.27256415098265	18.83435609997285
C	6.91894313744155	15.32994900857719	17.93813967733958
C	5.25892160339330	19.80610433952315	18.76692559244650
H	6.14140727964035	19.91095634125250	18.13617917669220
C	-0.77431197157270	16.00453523326577	18.56277972592170
H	-1.70142147017930	16.57922451414225	18.53171266135332
C	4.61911454600260	20.88476861751890	24.47125241725715
H	4.73311170109088	21.85540134398505	24.95612349246579
C	-0.44561000592394	15.27178862819340	19.70155426108395
H	-1.11430576985358	15.29240057871536	20.56206409591168
C	5.27831975323347	13.55756074594863	18.66518698898496
C	1.28658072590779	15.28242009524720	17.49594725339647
C	3.12941553931090	15.49260191162564	26.60391980283695
H	2.18380110876250	15.04815159292834	26.91032301370514
C	5.49419156472219	12.15861955208237	25.05872845872987
H	5.45523497620778	11.10366458439729	24.74983203409204
H	5.40597967986564	12.20950925760440	26.15155422869986
H	6.46021484578262	12.58856171019899	24.77451057083066
C	1.85137692531390	17.47098711964060	25.60904090191875
H	2.02571142174405	18.07939694611980	24.70840078977414
C	1.70761488803533	19.42215851832749	21.20615230507485
H	2.02528108796507	19.25841381772403	22.24566996746579
C	2.80289482918247	12.37778849657089	18.08627509941821
H	1.84040966328618	11.94231466780398	17.80865975569584
C	7.48677653310559	13.91636660901104	19.86344616438639
C	4.34202592238356	14.81440728977397	26.87805458249924
H	4.31162349660279	13.84725434443246	27.38193942207864
C	5.58070267537808	15.31834697897807	26.40998841653331
H	6.48868975426558	14.73727988613249	26.56435362722570
C	8.66930038288627	14.65095164429218	20.02545275929232
H	9.36064159724455	14.38404975357808	20.82572471971477
C	3.99581529733699	19.80021248987112	18.17478207843954
H	3.90100245092736	19.88788566481334	17.09154177821762
C	5.63065120277265	16.52907553767268	25.66970783887774
C	0.09496821324289	16.01972197296918	17.47691918812926
H	-0.15644315124979	16.60930926635485	16.59372260474450
C	6.78798669653644	19.66568652608183	20.79856524741263
H	6.70620400098323	20.20648538611662	21.75510039675992
C	1.10162150342433	13.75476903299302	21.01456345934072
H	2.20204737618520	13.74071068055827	21.05026377352962
C	5.21790678838652	12.26332508741057	18.12912268686965
H	6.14212152665519	11.73137995652532	17.89429700213610
C	2.18740847051295	15.29779430193554	16.27199582630152
H	3.11808726365287	14.77930497950850	16.54080544411637
C	0.66019935305324	16.55093000606958	25.33068980958356
H	0.92585192754482	15.80148313623980	24.57550970906171
H	-0.19364829809789	17.13544689828613	24.95980472849390
H	0.32535016134763	16.02490849115423	26.23754087439703
C	6.08081646754591	15.60660688938799	16.69553332480914
H	5.02762669147282	15.42869216561009	16.95690015057313
C	4.07545927244964	12.47652036885210	22.57936343705942
H	4.63015935823413	13.12542279443349	21.89285445574232

H	3.03395167592950	12.44733065781585	22.24275026728538
H	4.48249549180177	11.45707573234425	22.53279156186397
C	8.11025866842899	16.03866590895538	18.14110028114780
H	8.36884195297522	16.85480261626538	17.46764980112444
C	0.60511165785343	14.42057741331883	22.29808724448384
H	0.88693044304292	15.48122565934909	22.33297028839040
H	1.05864494090450	13.93652372138980	23.17315783773058
H	-0.48732763610826	14.34609120299431	22.40647473668992
C	7.24349486301178	12.70646047914114	20.74713743073064
H	6.19906639914478	12.39448953639153	20.60524088496249
C	6.95230084838116	17.09060875829340	25.17657501189008
H	6.71066610050421	17.80608488970309	24.37708856609617
C	1.55593296892321	18.42703226382687	26.77606463254451
H	1.41625987088445	17.86341516820469	27.71136149823396
H	0.63876456551115	19.00291914138058	26.58330395892244
H	2.37961719977987	19.13741538759610	26.92671680845623
C	6.44180136886597	14.60129850496287	15.58735201611888
H	5.81679065093972	14.77104380200819	14.69781208181657
H	6.28282594671123	13.56838939042762	15.92401531571297
H	7.49670139587947	14.71042252550379	15.29350803362606
C	8.97597683703766	15.71292575219432	19.18244384111728
H	9.89341128835795	16.28471674133424	19.32959213607780
C	0.88444724160263	20.71938002351157	21.16933907104433
H	1.48058466478780	21.57263799597631	21.52226886071051
H	-0.00510997593110	20.63148300412289	21.81069257566482
H	0.54509620434450	20.94400622147804	20.14685817355899
C	2.55513622321433	16.72743386981166	15.85267416182688
H	1.67258053644150	17.29373927357052	15.52014545369391
H	3.26942873048123	16.70837495481403	15.01712956923502
H	3.01572743642841	17.27500793910928	16.68854620923658
C	3.98352175616007	11.66388969798117	17.86851006120168
H	3.94349132492874	10.65548966034572	17.45406761058399
C	6.18929013754272	17.04589750716979	16.18701562675948
H	7.19313697612641	17.27243384744440	15.79805471473073
H	5.95258028415896	17.76444036190874	16.98407337688936
H	5.47975203767822	17.20651914663438	15.36438420987515
C	1.54849848684140	14.51604839543224	15.11320057146516
H	1.34582177052063	13.47643300823303	15.40626563767708
H	2.21608941277555	14.50317379270627	14.23892499683418
H	0.59501166027549	14.97323903686562	14.80882998688268
C	0.86827881082238	18.21195100459029	20.78265695337774
H	0.50767703667831	18.29839315662793	19.74828219150414
H	-0.00855842667404	18.09323049212838	21.43582782243514
H	1.45732714799519	17.28373582451022	20.84424389817371
C	7.87930967585222	16.03116160697025	24.57763376704849
H	8.78173915941233	16.50564703608632	24.16614704262500
H	7.36813533285512	15.49315971181708	23.76921250925469
H	8.20704014584507	15.29344731583618	25.32565668510281
C	7.87181734772612	20.35672211337578	19.96414982809138
H	8.10159693586312	19.79424329311585	19.04633993833406
H	8.80336447950378	20.42168847254569	20.54423965406707
H	7.57691806138904	21.37570286123301	19.67529982497868
C	7.63703056220105	17.86954245077263	26.31135466507470
H	7.88227447927812	17.20261302950173	27.15220561178279
H	6.98453871670084	18.66788616012973	26.69098745753745
H	8.57049940421895	18.33059548297349	25.95671689452858
C	7.45071907625738	13.00581603765926	22.23311265910060
H	7.26915091276863	12.09935361718042	22.82590649154061
H	6.76541704608618	13.79164899283980	22.58259876732069
H	8.47763583296742	13.33531477556617	22.44570679011661
C	8.14201132943219	11.53871959707678	20.30118780855372
H	9.20430892102265	11.79480567810879	20.43138942448752

H	7.98669756671394	11.29446939143713	19.24143898003269
H	7.93208592330185	10.63786740718956	20.89702143283449
C	0.60618665902114	12.30115390039725	20.93264956653927
H	-0.48898021738440	12.27448209326161	20.82829260581458
H	0.87104390654659	11.74529509569003	21.84563311377618
H	1.04614764388470	11.77487392628416	20.07599720628438
C	7.21324058968562	18.22487272172867	21.13333079677126
H	6.51882418294564	17.75740251824676	21.84462767126247
H	8.21636006790349	18.21132233540448	21.58569678857869
H	7.24617969756493	17.60186979916577	20.22739103356788

Complex 8

Sn	-2.84478359970493	12.60634243949793	8.81727640193991
Sn	-5.71136182834340	13.74845444610356	9.25289100362905
Fe	-6.39321532598300	11.71276171657648	11.06233913173638
P	-4.49412887558463	12.55557068160116	11.73834221063179
P	-3.62340398104057	14.24878492666745	10.77972059122360
P	-4.41410643799926	10.50847589914551	11.03114181076437
P	-5.16748147040232	11.07489543335740	9.09874096231439
C	-3.78514138233641	14.14139244634817	4.67399966474207
H	-4.10713064512426	13.66002092744134	3.74883132277440
C	-5.87793116467781	11.94983984153050	14.03200454280168
C	-3.34377342621487	15.46385370079392	4.65869289940083
H	-3.33097907833555	16.02939001707803	3.72588587966970
C	-6.95258579762798	11.72296801239325	13.00939468554848
C	-6.03846807223241	11.74764874894427	15.40506835503368
H	-6.98410308892474	11.36569813376442	15.78993655238815
C	-1.68355242796583	11.36925632583798	5.67469245033051
H	-1.57368094163627	12.38429419461110	6.08661971626447
C	-4.16998407676287	11.95722000668379	5.82050704202909
C	-2.33976201313268	16.00233950244337	8.26299808102622
C	-4.64586872062385	12.46508218177255	13.55620854530452
C	-3.35170154289807	13.99159059657773	7.07597295442422
C	-5.53067685686630	11.57665568817951	5.80225936667553
C	-3.78392502298834	13.40147961649315	5.86477909402024
C	-4.99732234160783	12.05353355295737	16.28684095286531
H	-5.12912712048428	11.88706259407055	17.35668554352358
C	-5.85177453937929	10.21442116007769	5.76220855935903
H	-6.89850786435949	9.90870622632556	5.75837978487869
C	-1.02021006039425	15.70424288536626	8.68163984451513
C	-3.11074317736196	16.96031415270856	8.96227507539531
C	-3.80092080248330	12.59321232897966	15.81043281219086
H	-2.99950859911964	12.85353454905345	16.50340481461904
C	-3.61290532642235	12.81958025188323	14.44008411650660
C	-2.89465839864195	15.32948030661824	7.04742307235473
C	-2.89054364387662	16.04698949044130	5.84051073599329
H	-2.50083358916473	17.06625900537513	5.83366543427974
C	-3.14999820712433	10.97421861996463	5.79873685711289
C	-4.52971744694357	17.30320956802680	8.53361078653507
H	-4.90619472283997	16.45040278911037	7.94474317496307
C	-1.36556708967867	12.70064289999641	13.30528604942497
C	-8.27167506617534	12.72227306101091	11.17610533256538
H	-8.65383793445190	13.59679908109695	10.65271112230182
C	-7.52104262017449	12.89424512485763	12.36891011323392
C	-6.63825384242825	12.61882949438490	5.82493069331856
H	-6.20358387825904	13.53940516263786	6.24909584492575
C	-6.46840251396390	7.96808322287492	12.31675873384974
H	-7.39154284581684	7.57215953434840	11.86835655085333
H	-6.01992148741271	7.15739832882462	12.90777752388172
H	-5.77051300257946	8.22775421276942	11.50943555249191
C	-1.46732396584086	11.18518112543410	13.24011373053144

H	-2.53627601813966	10.92136169512575	13.25059932420433
C	-3.51578853119602	9.62176408327261	5.78748390368215
H	-2.74095817596966	8.85457684963988	5.78439308172303
C	-1.29469590835174	11.44937880304490	4.18731227272122
H	-1.40616939767794	10.46633161999020	3.70512785025817
H	-0.24853963788589	11.77147048139496	4.07510788641308
H	-1.93344475957035	12.16505543235249	3.65245850016766
C	-6.74748184452109	9.17087086984887	13.22219925426208
H	-5.80898398558180	9.43958861505164	13.73223012722543
C	-8.45032617509644	11.43939197435496	10.60487729801686
H	-8.93981851030554	11.33777887259754	9.63721670221610
C	-7.96155101437340	10.29603905245393	11.27591246579786
H	-8.07625360168935	9.31806887399363	10.81314748506153
C	-4.55634950675438	18.54727381981855	7.62840788779401
H	-4.14499910065003	19.41755673184693	8.16210295746728
H	-5.58834766204616	18.78564645267822	7.32984672116704
H	-3.96445595401685	18.39642744678328	6.71753924762257
C	-4.85538855638232	9.24235086999673	5.76578679348196
H	-5.12338085586121	8.18472392064761	5.75929763319258
C	-7.23244374775948	10.41020639528018	12.49340455442653
C	-2.37932966918252	13.47193448061458	13.91532711893182
C	-2.54314265005855	17.61065735647560	10.06558077305846
H	-3.13137550865800	18.34194985656629	10.62042243944689
C	-0.72541686102153	10.45244733857023	6.44323740042692
H	-1.01742309253254	10.36758717811060	7.49988964780579
H	0.29531839941870	10.85950773960930	6.40446915011397
H	-0.68984149408777	9.44100701896088	6.01244403896108
C	-7.32803429577960	14.26994471238066	12.98704003839143
H	-6.27362637328867	14.35229851792630	13.29259599552242
C	-2.28101878873359	14.87887396251662	13.98574673042413
C	-0.87377817531826	10.59527125992578	11.95694735082596
H	-1.28404580301835	11.09043093826663	11.06426074987034
H	-1.11170921467754	9.52444613172544	11.88790746243631
H	0.22169248998815	10.69277411328332	11.92785321039868
C	-0.23968946615770	13.36112436018008	12.79963147895915
H	0.55153584428756	12.78487794300377	12.32019812689789
C	-7.78900937250930	8.79441624095686	14.29313461023661
H	-8.74093114549461	8.51218561360304	13.81867728159274
H	-7.99288851416905	9.62690533609414	14.97879595450152
H	-7.43704106108139	7.93864465769585	14.88681955904058
C	-5.47491479518968	17.50342645565232	9.72490861413817
H	-5.38464530748024	16.67832973685177	10.44380571215487
H	-6.51789922959850	17.55023150830933	9.37953531143884
H	-5.26520019421377	18.44107293293933	10.26116154592174
C	-0.49318788065311	16.37665664419557	9.78924067063603
H	0.52035953230637	16.15291231372189	10.12137669441307
C	-7.12636429760236	12.94429222369754	4.40273629454376
H	-6.30964295321634	13.32055421291182	3.77398215643641
H	-7.91747886781142	13.70877408402179	4.42950254637076
H	-7.53637334327707	12.04331230397272	3.92157196029006
C	-3.40805489317875	15.72907511725206	14.55473154156441
H	-4.25205901172022	15.05759837850739	14.77149050224913
C	-0.14919659803750	14.72413431329004	7.90809815054056
H	-0.82122231426478	14.03719554129007	7.37040916543761
C	-1.24469281907664	17.32582166297731	10.47563032726779
H	-0.81908917446522	17.84108568460312	11.33766447623744
C	-7.61743575352238	15.43203301473966	12.03938865036877
H	-8.68805384248333	15.51108076694964	11.79788247964678
H	-7.05808319496008	15.33522193076784	11.09835662637546
H	-7.30984934840752	16.37766909600045	12.50529129066813
C	-7.82658833709493	12.20922985041575	6.70722312885619
H	-8.41575423199184	11.40444591212808	6.24261982882690

H	-8.49843563099029	13.06652322208259	6.85886849315570
H	-7.49135589517498	11.85206834816315	7.69181505185854
C	0.67157652964637	15.46639887922760	6.83953544739149
H	1.34824552752283	16.19553696320896	7.31018295809762
H	0.01327828111376	16.00816524202253	6.14685563557388
H	1.27884472796188	14.75939266769371	6.25469774374181
C	-0.83127084188476	10.55428821170855	14.49128473656455
H	0.24068772176974	10.79734324793077	14.54540299418213
H	-0.93540584314891	9.45922740306554	14.46965003355042
H	-1.30924389073286	10.92654204834248	15.40836291462832
C	-1.13032539884898	15.49585024693507	13.48381471820174
H	-1.04134083887783	16.58185640803458	13.52869208005048
C	-8.19496954385420	14.37888495902648	14.25379559411491
H	-8.04848885412307	15.35614924137166	14.73573153825476
H	-7.93988023703331	13.59824590754687	14.98152620838826
H	-9.26099415099720	14.27796193600956	14.00035382548763
C	0.75092061996697	13.87126902943844	8.81124429094366
H	1.22517480696910	13.07177548046573	8.22445853604337
H	0.17295882235685	13.40471831231629	9.62209716194798
H	1.55616338028858	14.46748030290002	9.26543816889312
C	-0.11645917882591	14.74528474326040	12.89690149620072
H	0.76916618799494	15.24181407622655	12.49890475264835
C	-3.90657156759237	16.76837719141565	13.53967858016765
H	-3.13309874446113	17.51687514685467	13.31400510869913
H	-4.78397794651090	17.30062292535842	13.93656504468892
H	-4.18371192718079	16.28396218635527	12.59265563072665
C	-2.99450787777633	16.38852181536355	15.87945100482530
H	-2.70232948860222	15.63383805859220	16.62353023068894
H	-3.82313527700579	16.98076021067665	16.29558275068796
H	-2.13664305573355	17.06160407353077	15.73240245332201

References

(1) Pu, L.; Phillips, A. D.; Richards, A. F.; Stender, M.; Simons, R. S.; Olmstead, M. M.; Power, P. P. Germanium and Tin Analogues of Alkynes and Their Reduction Products. *J. Am. Chem. Soc.* **2003,** *125*, 11626-11636.
(2) Brennessel, W. W.; Jilek, R. E.; Ellis, J. E. Bis(1,2,3,4-η^4-Anthracene)Ferrate(1-): A Paramagnetic Homoleptic Polyarene Transition-Metal Anion. *Angew. Chem., Int. Ed.* **2007** *46*, 6132-6136.
(3) Jonas, K.; Schieferstein, L.; Krüger, C.; Tsay, Y. H. Tetrakis(Ethylene)Irondilithium and Bis(η^4-1,5-Cyclooctadiene)Irondilithium. *Angew. Chem., Int. Ed.* **1979,** *18*, 550-551.
(4) Budzelaar, P. H. M. *gNMR for Windows*, 5.0.6; IvorySoft: 2006.
(5) Scheinost, A. C.; Claussner, J.; Exner, J.; Feig, M.; Findeisen, S.; Hennig, C.; Kvashnina, K. O.; Naudet, D.; Prieur, D.; Rossberg, A.; Schmidt, M.; Qiu, C.; Colomp, P.; Cohen, C.; Dettona, E.; Dyadkin, V.; Stumpf, T. ROBL-II at ESRF: A Synchrotron Toolbox for Actinide Research. *J. Synchrotron Rad.* **2021,** *28*, 333-349.
(6) Sheldrick, G. M. Shelxt - Integrated Space-Group and Crystal-Structure Determination. *Acta Cryst. A* **2015,** *71*, 3-8.
(7) Dolomanov, O. V.; Bourhis, L. J.; Gildea, R. J.; Howard, J. A. K.; Puschmann, H. Olex2: A Complete Structure Solution, Refinement and Analysis Program. *J. Appl. Crystallogr.* **2009,** *42*, 339-341.
(8) (a) Sheldrick, G. M. Crystal Structure Refinement with Shelxl. *Acta Cryst. C* **2015,** *71*, 3-8.
(9) Bourhis, L. J.; Dolomanov, O. V.; Gildea, R. J.; Howard, J. A.; Puschmann, H. The Anatomy of a Comprehensive Constrained, Restrained Refinement Program for the Modern Computing Environment - Olex2 Dissected. *Acta Cryst. A* **2015,** *71*, 59-75.
(10) (a) *Sadabs*, BRUKER AXS GmbH: Karlsruhe, Germany, 2016. (b) *Crysalispro, Scale3 Abspack*, Rigaku Oxford Diffraction: 2019.
(11) Clark, R. C.; Reid, J. S. The Analytical Calculation of Absorption in Multifaceted Crystals. *Acta Cryst. A* **1995,** *51*, 887-897.
(12) *Crysalispro Software System*, Rigaku Oxford Diffraction: 2023.
(13) (a) Neese, F. The Orca Program System. *WIREs Computat. Mol. Sci.* **2011,** *2*, 73-78. (b) Neese, F. Software Update: The Orca Program System—Version 5.0. *WIREs Computat. Mol. Sci.* **2022,** 12:e1606.
(14) (a) Becke, A. D. Density-Functional Exchange-Energy Approximation with Correct Asymptotic Behavior. *Phys. Rev. A* **1988**, *38*, 3098-3100. (b) Becke, A. D. Density-Functional Thermochemistry. III. The Role of Exact Exchange. *J. Chem. Phys.* **1993,** *98*, 5648-5652. (c) Becke, A. D. A New Mixing of Hartree–Fock and Local Density-Functional Theories. *J. Chem. Phys.* **1993,** *98*, 1372-1377. (d) Lee, C.; Yang, W.; Parr, R. G. Development of the Colle-Salvetti Correlation-Energy Formula into a Functional of the Electron Density. *Phys. Rev. B* **1988,** *37*, 785-789. (e) Orio, M.; Pantazis, D. A.; Neese, F. Density Functional Theory. *Photosynth. Res.* **2009,** *102*, 443-453. (f) Lin, Y. S.; Li, G. D.; Mao, S. P.; Chai, J. D. Long-Range Corrected Hybrid Density Functionals with Improved Dispersion Corrections. *J. Chem. Theory Comput.* **2013,** *9*, 263-272.
(15) (a) Schäfer, A.; Horn, H.; Ahlrichs, R. Fully Optimized Contracted Gaussian Basis Sets for Atoms Li to Kr. *J. Chem. Phys.* **1992,** *97*, 2571-2577. (b) Weigend, F.; Ahlrichs, R. Balanced Basis Sets of Split Valence, Triple Zeta Valence and Quadruple Zeta Valence Quality for H to Rn: Design and Assessment of Accuracy. *Phys. Chem. Chem. Phys.* **2005,** *7*, 3297-3305.

(16) Weigend, F. Accurate Coulomb-Fitting Basis Sets for H to Rn. *Phys. Chem. Chem. Phys.* **2006,** *8*, 1057-1065.
(17) (a) Metz, B.; Stoll, H.; Dolg, M. Small-Core Multiconfiguration-Dirac–Hartree–Fock-Adjusted Pseudopotentials for Post-*d* Main Group Elements: Application to PbH and PbO. *J. Chem. Phys.* **2000,** *113*, 2563-2569. (b) Peterson, K. A.; Figgen, D.; Goll, E.; Stoll, H.; Dolg, M. Systematically Convergent Basis Sets with Relativistic Pseudopotentials. II. Small-Core Pseudopotentials and Correlation Consistent Basis Sets for the Post-*d* Group 16–18 Elements. *J. Chem. Phys.* **2003,** *119*, 11113-11123.
(18) Eichkorn, K.; Treutler, O.; Öhm, H.; Häser, M.; Ahlrichs, R. Auxiliary Basis Sets to Approximate Coulomb Potentials. *Chem. Phys. Lett.* **1995,** *242*, 652-660.
(19) (a) Grimme, S.; Ehrlich, S.; Goerigk, L. Effect of the Damping Function in Dispersion Corrected Density Functional Theory. *J. Comput. Chem.* **2011,** *32*, 1456-1465. (b) Grimme, S.; Antony, J.; Ehrlich, S.; Krieg, H. A Consistent and Accurate Ab Initio Parametrization of Density Functional Dispersion Correction (Dft-D) for the 94 Elements H-Pu. *J. Chem. Phys.* **2010,** *132*, 154104.
(20) Knizia, G. Intrinsic Atomic Orbitals: An Unbiased Bridge between Quantum Theory and Chemical Concepts. *J. Chem. Theory Comput.* **2013,** *9*, 4834-4843.
(21) Chemcraft - Graphical Software for Visualization of Quantum Chemistry Computations. https://www.chemcraftprog.com.
(22) Neese, F.; Wennmohs, F.; Hansen, A.; Becker, U. Efficient, Approximate and Parallel Hartree–Fock and Hybrid DFT Calculations. A 'Chain-of-Spheres' Algorithm for the Hartree–Fock Exchange. *Chem. Phys.* **2009,** *356*, 98-109.
(23) (a) Klamt, A.; Schüürmann, G. Cosmo: A New Approach to Dielectric Screening in Solvents with Explicit Expressions for the Screening Energy and Its Gradient. *J. Chem. Soc., Perkin Trans. 2* **1993**, 799-805. (b) Barone, V.; Cossi, M. Quantum Calculation of Molecular Energies and Energy Gradients in Solution by a Conductor Solvent Model. *J. Phys. Chem. A* **1998,** *102*, 1995-2001.
(24) Glendening, E. D.; Landis, C. R.; Weinhold, F. Nbo 6.0: Natural Bond Orbital Analysis Program. *J. Comput. Chem.* **2013,** *34*, 1429-1437.
(25) (a) Mitoraj, M. P.; Michalak, A.; Ziegler, T. A Combined Charge and Energy Decomposition Scheme for Bond Analysis. *J. Chem. Theory Comput.* **2009,** *5*, 962-975. (b) Zhao, L.; von Hopffgarten, M.; Andrada, D. M.; Frenking, G. Energy Decomposition Analysis. *WIREs Computat. Mol. Sci.* **2018,** 8:e1345. (c) Savin, A.; Nesper, R.; Wengert, S.; Fässler, T. F. ELF: The Electron Localization Function. *Angew. Chem., Int. Ed.* **2003,** *36*, 1808-1832. (d) Lu, T.; Chen, Q. Independent Gradient Model Based on Hirshfeld Partition: A New Method for Visual Study of Interactions in Chemical Systems. *J. Comput. Chem.* **2022,** *43*, 539-555. (e) Klein, J.; Khartabil, H.; Boisson, J. C.; Contreras-Garcia, J.; Piquemal, J. P.; Henon, E. New Way for Probing Bond Strength. *J. Phys. Chem. A* **2020,** *124*, 1850-1860. (f) Fradera, X.; Austen, M. A.; Bader, R. F. W. The Lewis Model and Beyond. *J. Phys. Chem. A* **1998,** *103*, 304-314.
(26) (a) Lu, T.; Chen, F. Multiwfn: A Multifunctional Wavefunction Analyzer. *J. Comput. Chem.* **2012,** *33*, 580-592. (b) Lu, T. A Comprehensive Electron Wavefunction Analysis Toolbox for Chemists, Multiwfn. *J. Chem. Phys.* **2024,** *161*, 082503.
(27) (a) Caldeweyher, E.; Bannwarth, C.; Grimme, S. Extension of the D3 Dispersion Coefficient Model. *J. Chem. Phys.* **2017,** *147*, 034112. (b) Caldeweyher, E.; Ehlert, S.; Hansen, A.; Neugebauer, H.; Spicher, S.; Bannwarth, C.; Grimme, S. **2019**, 10.26434/chemrxiv.7430216.v2.
(28) Bursch, M.; Mewes, J. M.; Hansen, A.; Grimme, S. Best-Practice Dft Protocols for Basic Molecular Computational Chemistry. *Angew. Chem., Int. Ed.* **2022,** *61*, e202205735.
(29) Grimme, S.; Hansen, A.; Ehlert, S.; Mewes, J. M. r2SCAN-3c: A "Swiss Army Knife" Composite Electronic-Structure Method. *J. Chem. Phys.* **2021,** *154*, 064103.
(30) Jerabek, P.; Schwerdtfeger, P.; Frenking, G. Dative and Electron-Sharing Bonding in Transition Metal Compounds. *J. Comput. Chem.* **2019,** *40*, 247-264.
(31) Stoychev, G. L.; Auer, A. A.; Izsak, R.; Neese, F. Self-Consistent Field Calculation of Nuclear Magnetic Resonance Chemical Shielding Constants Using Gauge-Including Atomic Orbitals and Approximate Two-Electron Integrals. *J. Chem. Theory Comput.* **2018,** *14*, 619-637.
(32) Tao, J.; Perdew, J. P.; Staroverov, V. N.; Scuseria, G. E. Climbing the Density Functional Ladder: Nonempirical Meta-Generalized Gradient Approximation Designed for Molecules and Solids. *Phys. Rev. Lett.* **2003,** *91*, 146401.
(33) Rolfes, J. D.; Neese, F.; Pantazis, D. A. All-Electron Scalar Relativistic Basis Sets for the Elements Rb-Xe. *J. Comput. Chem.* **2020,** *41*, 1842-1849.
(34) Stuckrath, J. B.; Gasevic, T.; Bursch, M.; Grimme, S. Benchmark Study on the Calculation of ^{119}Sn NMR Chemical Shifts. *Inorg. Chem.* **2022,** *61*, 3903-3917.
